# Identification and expression analysis of chemosensory receptor genes in an aphid endoparasitoid *Aphidius gifuensis*

**DOI:** 10.1038/s41598-017-03988-z

**Published:** 2017-06-21

**Authors:** Zhi-Wei Kang, Hong-Gang Tian, Fang-Hua Liu, Xiang Liu, Xiang-Feng Jing, Tong-Xian Liu

**Affiliations:** 1 State Key Laboratory of Crop Stress Biology for the Arid Areas, and Key Laboratory of Northwest Loess Plateau Crop Pest Management of Ministry of Agriculture, Northwest A&F University, Yangling, Shaanxi 712100 China; 2 State Key Laboratory of Integrated Management of Pest and Rodents, Institute of Zoology, Chinese Academy of Sciences, Beijing, 100101 China; 3 Entomology Department, College of Plant Protection, Yunnan Agricultural University, Kunming, 650201 China

## Abstract

Olfaction and gustation play critical roles during the host-location search process of insects. Several chemosensory receptor genes are thought to be involved in providing specificity to the olfactory sensory neuron responses. The aphid endoparasitoid, *Aphidius gifuensis*, has been used as a biological control agent against a variety of aphid species; this parasitoid is able to detect its target host(s) effectively during the parasitic process. To understand the mechanism of host detection in *A*. *gifuensis*, we assembled specific antennal transcriptomes of each sex through next generation sequencing technology to identify the major chemosensory receptor genes. Using a bioinformatics screen, we identified 100 olfactory receptors candidates (62 odorant receptors, 15 gustatory receptors, and 23 ionotropic receptors) from the sex-specific antennal transcriptome. In addition, combining with the demonstrated functions of chemosensory genes in other insects, the sex-, tissue-, and host-specific expression profile of chemosensory genes potentially revealed the candidate physiological functions. The identification and expression profile of chemosensory receptor genes in *A*. *gifuensis* provide valuable information for understanding and investigating the intraspecific or interspecific chemical communications in the solitary parasitic wasps.

## Introduction

In recent years, the extensive use of pesticide-based pest management has led to drastic effects on our ecosystem and environment^1, 2^. Therefore, integrated pest management (IPM) has gained great attention as a strategy to protect crops from pest losses^3, 4^. Biological control is a key agro-system service and a pillar of IPM^5, 6^. Because of the key advantage of their capacity to both kill and reproduce at the expense of their hosts, parasitoids have been widely used in biological control against insect pests^7, 8^.

Aphids are key insect pests that are responsible for major agricultural losses, particularly as they are vectors of various plant viruses^9^. Aphids can be attacked by a wide variety of natural enemies, including several endoparasitoids. Different parasitic wasps usually have distinct hosts, and they can locate their target hosts accurately and efficiently^6, 9^. The success of parasitoids in locating their hosts in a complex environment depends mainly on the accurate recognition of a series of several chemical molecules. In most insects, the chemosensory system is involved in foraging, oviposition site selection, mate choice, and social communication (among social insects)^10–15^. It is likely that the semiochemicals from foods, hosts, mates, or partners are received by insect chemoreceptors at the membrane surface of chemosensory neurons such as olfactory receptor neurons (ORNs) and gustatory receptor neurons (GRNs)^16–18^. Chemoreceptors include three large, distinct families: odorant receptors (ORs), gustatory receptors (GRs), and ionotropic receptors (IRs)^19^.

Insect ORs were the first chemoreceptor family to be discovered in *Drosophila melanogaster* genome^20^. Until date, insect ORs have been identified in many species, including *Apis mellifera* ^21^, *Macrocentrus cingulum* ^10^, *Conogethes punctiferalis* ^22^, and *Bombyx mori* ^23^, with a high degree of divergence, both within and across species. These receptors are ligand-gated ion channels, composed of seven novel transmembrane domains with an inverted membrane topology, compared with mammalian ORs^24–26^. ORs are expressed in ORNs and can receive a variety of volatile chemicals, including pheromones and general odorants^14, 19^. The function of an insect OR depends on the presence of a non-ligand binding odorant receptor co-receptor (Orco), which functions as a ligand-gated ion channel^27–29^. In contrast to ORs, Orco is highly conserved across insect species.

After the annotation of ORs in *D*. *melanogaster* genome, GRs, a common ancestor to ORs and composed of seven transmembrane domains, were discovered in *D*. *melanogaster* ^30^. In insect, GRs are also conserved in their sequence and structure. They are highly expressed in the GRNs in taste organs^31, 32^. For instance, GRs have been shown to play a critical role in coordinating insect feeding behaviors. GRs located on the dendrites of taste sensilla recognize the taste stimuli from the environment, especially in foods^32, 33^. In addition, GRs are also involved in the detection of carbon dioxide^34^. Based on the functional research data, GRs have been classified into four clades: CO_2_, GR43a-like, sugar, and bitter^19, 35^.

A large number of ORNs express neither ORs nor GRs, but they express IRs, which are also ligand-gated ion channels, but with three transmembrane domains^36–38^. IRs have been identified across Protostomia and are regarded as an ancient family of chemosensory receptors. IRs in insects can be classified into two types: the “antennal IRs,” which are conserved across insect orders with chemosensory function, and the “divergent IRs,” which are species-specific and are assigned a tentative role in taste^37, 38^. Meanwhile, two IRs, IR8a and IR25a, appear to act as co-receptors with the function of turning IRs sensory cilia targeting and IR-based sensory channels^37–40^.

*A*. *gifuensis* has been selected as a potential biological-control agent for the green peach aphid *Myzus persicae* Sulzer, one of the most common pests of several crops in China and Japan; it has already been successfully used to control *M*. *persicae* on tobacco in Yunnan and many other regions of China^41–43^. During the predation and parasitism, natural enemies utilize herbivore-induced volatiles (HIPVs), green leaf volatiles (GLVs), or the body volatiles such as aphid alarm pheromone E-beta-farnesene (EBF) to locate its hosts^44–46^. For example, *A*. *gifuensis* is able to discriminate the healthy, mechanically damaged or infested by its original aphid^44^. Furthermore, both female and male of *A*. *gifuensis* represented a positive electroantennogram (EAG) response to EBF and several plant volatiles, such as linalool, cis-3-hexen-1-ol, (E)-2-hexenal^45^. And a lot of natural enemies such as *Aphidius ervi*, *Aphidius uzbekistanicus*, *Adalia bipunctata* show attractant behavior to EBF^47^. All of these results revealed that natural enemies including *A*. *gifuensis* have evolved a comprehensive chemosensory system to enhance their parasitism efficiently.

However, the previous research on *A*. *gifuensis* only focused on its ecological behavior and anatomy. The potential molecular mechanism involved in the ecological process is lacking. Until now, the chemosensory receptors of parasitoid wasps have been only characterized in *Microplitis mediator* ^48^, *Nasonia vitripennis* ^49^, *Macrocentrus cingulum* ^10^, and *Chouioia cunea* ^15^. Therefore, we analyzed the chemosensory receptors of *A*. *gifuensis* based on sex-specific antennal transcriptomes through next-generation sequencing technology. The comprehensive identification and expression profile of chemosensory receptor genes in *A*. *gifuensis* provide valuable information for understanding the intraspecific or interspecific chemical communications, which is crucial for potential genetic manipulation of their sensitivity to chemical cues from hosts, plants, and themselves in biological control systems.

## Results

### Transcriptome assembly summary

The male and female *A*. *gifuensis* antennal transcriptomes were generated using Illumina Hiseq2000. Collectively, there were 38,848 transcripts, and the longest transcript was 13,876 bp in length. We identified a total of 19,074 components, each of which contained at least one annotated gene. The N50 transcript length was 1,980 bp and the total length of the assembled transcriptome was about 45.75 Mbp (Table S1).

### Functional annotation

**Figure 1:**
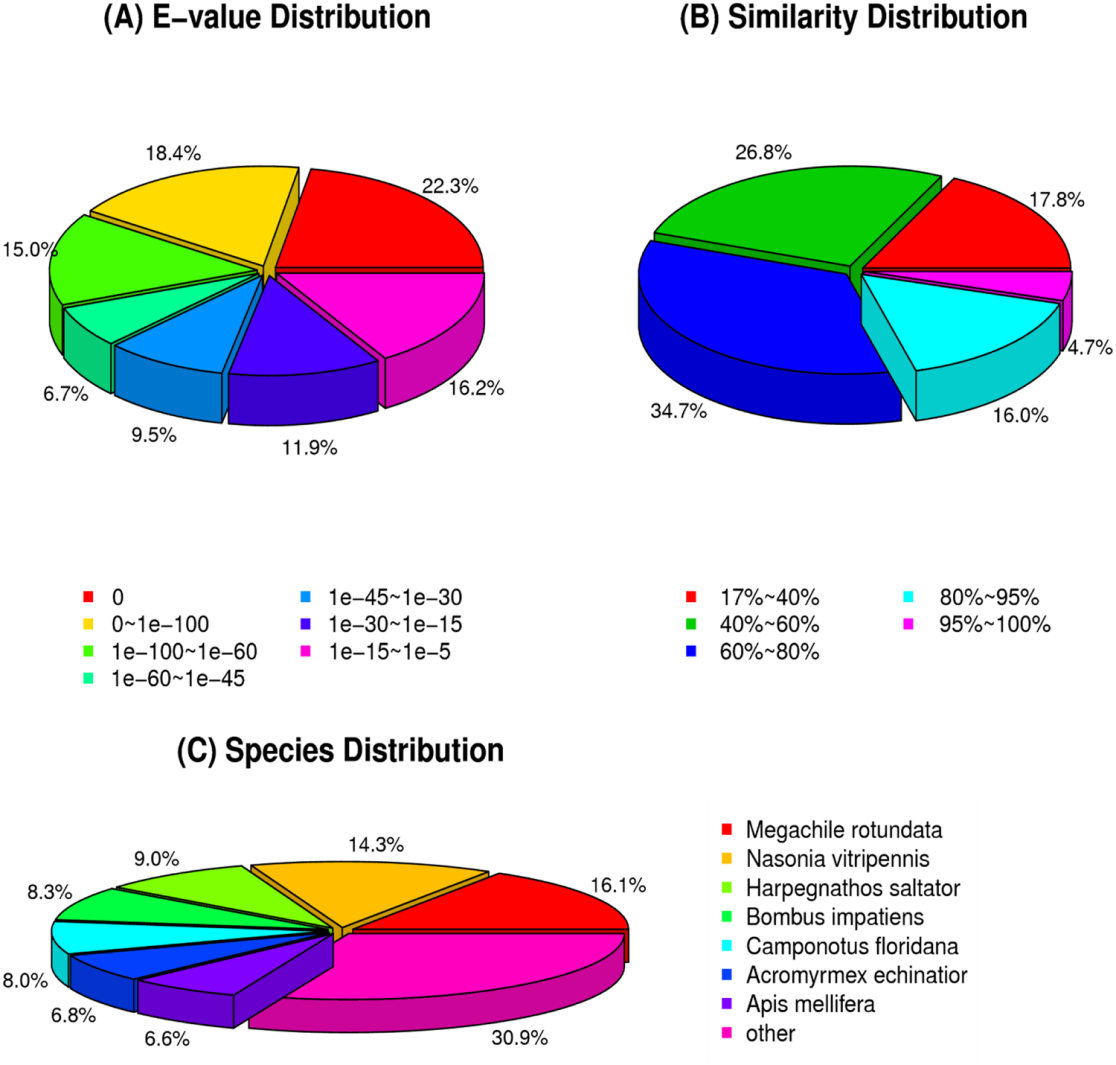
Homology analyses results. The BLASTx annotations of *Aphidius gifuensis* antenna transcripts (**A**) E-value distribution, (**B**) Similarity distribution, and (**C**) Species distribution.

Functional annotations for the assembled database of *A*. *gifuensis* transcriptome were generated through diverse protein datasets. A total of 29,302 unigenes were annotated: 26,969 (92.0%) in NR_Annotation, 21,411 (7.30%) in Nt_Annotation, 11,086 (37.8%) in COG_Annotation, 22,259 (78.0%) in Swiss-prot_Annotation, 12,552 (42.8%) in GO_Annotation, and 20,319 (69.3%) in KEGG_Annotation (Table S2). From the database in NR_Annotation, 16,830 (62.4%) had a strong match with an e-value less than 1e^−45^ (Fig. 1A). For the database in NR_Annotation, 1,278 (4.74%) showed a strong similarity (95–100%) to known proteins (Fig. 1B). Approximately 70% sequences matched to a hymenopteran sequence (Fig. 1C).

**Figure 2:**
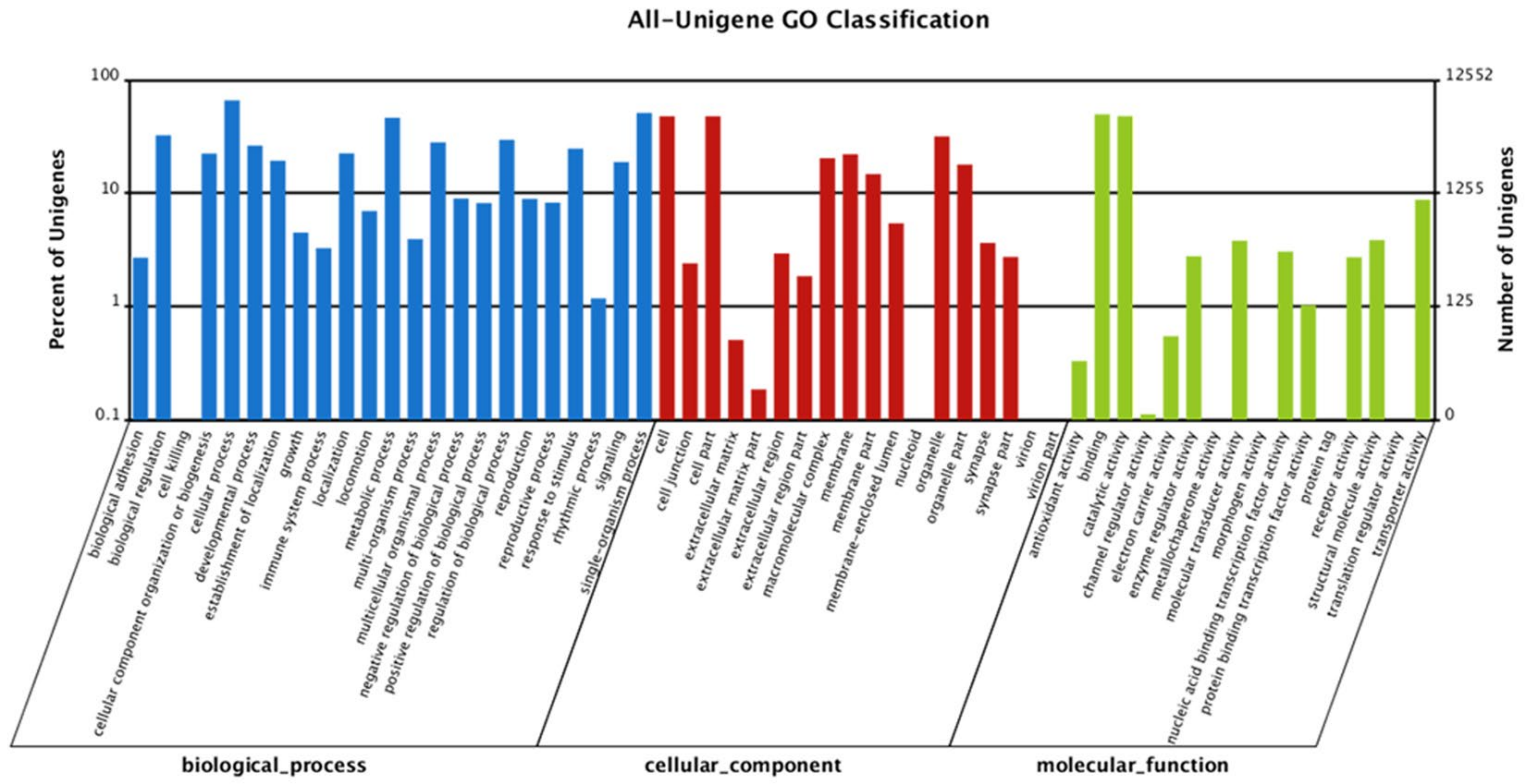
Functional annotation of *Aphidius gifuensis* antenna transcripts based on gene ontology (GO) categorization. GO analysis was performed at the level of two or three main categories (cellular component, molecular function, and biological process).

All the annotated unigenes were classified into three groups: biological process, cellular components, and molecular functions. In the biological process, the most represented biological processes were cellular process (8,294 antennae unigenes) and single-organism process (6,410 antennae unigenes). In the cellular components, the genes expressed in the antennae were mostly cell part- (6,000 antennae unigenes) and organelle-related (3,974 antennae unigenes). In the molecular functions, binding (6,226 antennae unigenes) and catalytic activity (6,014 antennae unigenes) were the highly expressed categories in antennae (Fig. 2). In total, 11,086 of the 29,302 unigenes with non-redundant database hits were grouped into 25 COG categories (Figure S1).

### Identification of chemosensory receptors

#### Odorant receptors

**Figure 3:**
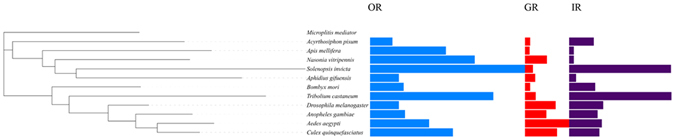
The number of chemosensory genes in different insect species. The digits by the histogram bars represent the numbers of chemosensory receptor genes in different subfamilies. A phylogenetic tree showing the phylogenetic relationships between these species is illustrated on the left. The data are obtained from the current study for *Microplitis mediator*, *Acyrthosiphon pisum*, *Apis mellifera*, *Nasonia vitripennis*, *Solenopsis invicta*, *Bombyx mori*, *Tribolium castaneum*, *Drosophila melanogaster*, *Anopheles gambiae*, *Aedes aegypti*, and *Culex quinquefasciatus*.

Sixty-two candidate ORs were identified (Table 1 and Fig. 3). Only one of the transcripts was an incomplete fragment, whereas all the other transcripts represented a full-length gene, containing complete open reading frames (ORF). The transcript name, length, best Blast P, e-value, and identity are presented in Table 1.

**Table 1:** Candidate odorant receptor transcripts identified in adult male and female *A*. *gifuensis* antennal transcriptomes.

Gene name	Unigene reference	Length(bp)	ORF(aa)	Status	Blast P hit	E-value	% Identify
Orco1	Unigene8357_All	1957	478	Complete	ref|XP_011296908.1| PREDICTED: odorant receptor coreceptor [*Fopius arisanus*]	0	88
Or2	CL2957.Contig2_All	1370	393	Complete	ref|XP_014297094.1| PREDICTED: odorant receptor 22a-like [*Microplitis demolitor*]	7e-79	33
Or3	CL14.Contig3_All	1386	383	Complete	gb|AKO89999.1| odorant receptor 35 [*Microplitis mediator*]	2e-91	38
Or4	CL394.Contig1_All	1305	394	Complete	gb|AKO90003.1| odorant receptor 39 [*Microplitis mediator*]	4e-87	40
Or5	CL1083.Contig3_All	1454	399	Complete	ref|XP_014298630.1| PREDICTED: odorant receptor 13a-like [*Microplitis demolitor*]	2e-99	37
Or6	CL1128.Contig6_All	1590	408	Complete	gb|AGG17944.1| olfactory receptor 11 [*Microplitis mediator*]	2e-46	31
Or7	CL3043.Contig1_All	1350	425	Complete	gb|AGG17945.1| olfactory receptor 12 [*Microplitis mediator*]	1e-81	36
Or8	CL4268.Contig1_All	1462	433	Complete	gb|AKO89984.1| odorant receptor 20 [*Microplitis mediator*]	8e-109	50
Or9	CL2112.Contig4_All	1489	431	Complete	ref|XP_011305064.1| PREDICTED: odorant receptor 2a-like [*Fopius arisanus*]	2e-73	38
Or10	Unigene8467_All	1666	431	Complete	ref|XP_015127536.1| PREDICTED: odorant receptor Or1-like isoform X2 [*Diachasma alloeum*]	0	58
Or11	CL2112.Contig2_All	1742	435	Complete	ref|XP_015121344.1| PREDICTED: odorant receptor 13a-like [*Diachasma alloeum*]	2e-87	36
Or12	CL1077.Contig5_All	1619	406	Complete	ref|XP_015112584.1|PREDICTED: odorant receptor 67c-like [*Diachasma alloeum*]	0	61
Or13	CL1464.Contig3_All	1188	303	Complete	ref|XP_008548428.1|PREDICTED: odorant receptor 13a [*Microplitis demolitor*]	4e-65	37
Or14	Unigene4468_All	1046	317	Complete	ref|XP_011308322.1|PREDICTED: odorant receptor Or1-like [*Fopius arisanus*]	4e-152	65
Or15	CL2275.Contig1_All	1486	365	Complete	ref|XP_015108891.1|PREDICTED: odorant receptor 10a-like [*Diachasma alloeum*]	3e-76	39
Or16	CL2679.Contig1_All	1119	329	Complete	gb|AKO89992.1|odorant receptor 28 [*Microplitis mediator*]	1e-160	71
Or17	Unigene5776_All	1395	399	Complete	ref|XP_008546680.1|PREDICTED: odorant receptor 13a-like [*Microplitis demolitor*]	1e-144	53
Or18	CL763.Contig2_All	1675	408	Complete	ref|XP_011300122.1|PREDICTED: odorant receptor 24a-like [*Fopius arisanus*]	3e-87	42
Or19	CL561.Contig3_All	1318	405	Complete	ref|XP_014295516.1|PREDICTED: odorant receptor 47a-like, partial [*Microplitis demolitor*]	1e-99	40
Or20	Unigene15618_All	1735	352	Complete	ref|XP_014295516.1|PREDICTED: odorant receptor 47a-like, partial [*Microplitis demolitor*]	3e-106	50
Or21	Unigene78_All	1392	397	Complete	ref|XP_011302983.1|PREDICTED: odorant receptor 13a-like [*Fopius arisanus*]	1e-69	31
Or22	CL382.Contig6_All	1474	344	Complete	gb|AKO90003.1|odorant receptor 39 [*Microplitis mediator*]	1e-170	66
Or23	Unigene20005_All	1389	389	Complete	gb|AKO89986.1|odorant receptor 22 [*Microplitis mediator*]	2e-145	51
Or24	CL1274.Contig1_All	1536	398	Complete	ref|NP_001177576.1|odorant receptor 204 [*Nasonia vitripennis*]	2e-55	31
Or25	CL3629.Contig4_All	1359	395	Complete	gb|AKO89985.1|odorant receptor 21 [*Microplitis mediator*]	1e-67	33
Or26	CL3629.Contig3_All	1365	394	Complete	ref|XP_014298630.1|PREDICTED: odorant receptor 13a-like [*Microplitis demolitor*]	3e-95	37
Or27	CL3527.Contig1_All	1435	345	Complete	gb|AKO89996.1|odorant receptor 32 [*Microplitis mediator*]	1e-170	68
Or28	Unigene8353_All	1317	380	Complete	ref|XP_015182294.1|PREDICTED: odorant receptor 43a-like [Polistes dominula]	1e-91	38
Or29	Unigene8366_All	1437	380	Complete	gb|AKO89987.1|odorant receptor 23 [*Microplitis mediator*]	1e-142	52
Or30	CL602.Contig7_All	1807	381	Complete	ref|XP_015120217.1|PREDICTED: odorant receptor 46a, isoform A-like [*Diachasma alloeum*]	2e-88	41
Or31	CL443.Contig11_All	1256	332	Complete	ref|XP_015115473.1|PREDICTED: odorant receptor Or1-like [*Diachasma alloeum*]	1e-67	37
Or32	Unigene11297_All	1453	384	Complete	ref|XP_011308185.1|PREDICTED: odorant receptor Or1 [*Fopius arisanus*]	3e-89	37
Or33	Unigene20726_All	1344	385	Complete	gb|AKO89982.1|odorant receptor 18 [*Microplitis mediator*]	7e-138	53
Or34	Unigene13329_All	1299	383	Complete	ref|XP_015120217.1|PREDICTED: odorant receptor 46a, isoform A-like [*Diachasma alloeum*]	1e-97	43
Or35	Unigene14444_All	1203	359	Complete	ref|XP_011308185.1|PREDICTED: odorant receptor Or1 [*Fopius arisanus*]	2e-154	59
Or36	CL602.Contig10_All	1471	376	Complete	ref|XP_015120217.1|PREDICTED: odorant receptor 46a, isoform A-like [*Diachasma alloeum*]	1e-87	41
Or37	CL602.Contig3_All	1546	375	Complete	ref|XP_011315403.1|PREDICTED: odorant receptor 67a-like [*Fopius arisanus*]	1e-80	35
Or38	CL1553.Contig4_All	1943	385	Complete	gb|AKO89982.1|odorant receptor 18 [*Microplitis mediator*]	8e-147	55
Or39	CL2797.Contig2_All	1199	320	Complete	gb|AKO89982.1|odorant receptor 18 [*Microplitis mediator*]	1e-93	45
Or40	CL2797.Contig1_All	1305	386	Complete	gb|AKO89982.1|odorant receptor 18 [*Microplitis mediator*]	9e-107	44
Or41	CL443.Contig13_All	1254	381	Complete	ref|XP_011308185.1|PREDICTED: odorant receptor Or1 [*Fopius arisanus*]	4e-91	38
Or42	CL2797.Contig3_All	1481	386	Complete	ref|NP_001164395.1|odorant receptor 82 [*Nasonia vitripennis*]	2e-66	33
Or43	CL443.Contig9_All	1173	348	Complete	ref|XP_015110532.1|PREDICTED: putative odorant receptor 92a [*Diachasma alloeum*]	2e-75	59
Or44	CL443.Contig7_All	1380	383	Complete	gb|AKO90002.1|odorant receptor 38 [*Microplitis mediator*]	4e-64	34
Or45	CL815.Contig2_All	1314	407	3′Lost	ref|NP_001177545.1|odorant receptor 143 [*Nasonia vitripennis*]	4e-46	31
Or46	CL255.Contig5_All	1298	393	Complete	gb|AKO89985.1|odorant receptor 21 [*Microplitis mediator*]	6e-71	34
Or47	CL2275.Contig4_All	1505	389	Complete	gb|AKO90003.1|odorant receptor 39 [*Microplitis mediator*]	3e-59	33
Or48	CL3267.Contig3_All	1255	394	Complete	gb|AKO90009.1| odorant receptor 45 [*Microplitis mediator*]	1e-72	35
Or49	Unigene20980_All	1352	387	Complete	ref|XP_011301745.1| PREDICTED: putative odorant receptor 85d [*Fopius arisanus*]	1e-78	39
Or50	CL255.Contig6_All	1417	392	Complete	ref|XP_011301745.1| PREDICTED: putative odorant receptor 85d [*Fopius arisanus*]	7e-90	42
Or51	CL1421.Contig1_All	1501	396	Complete	gb|AKO89985.1|odorant receptor 21 [*Microplitis mediator*]	3e-72	34
Or52	CL2275.Contig7_All	1467	390	Complete	ref|XP_015108891.1|PREDICTED: odorant receptor 10a-like [*Diachasma alloeum*]	1e-87	39
Or53	CL292.Contig2_All	1317	394	Complete	gb|AKO90007.1|odorant receptor 43 [*Microplitis mediator*]	1e-101	42
Or54	CL382.Contig8_All	1556	354	Complete	gb|AKO90003.1|odorant receptor 39 [*Microplitis mediator*]	1e-147	61
Or55	CL1083.Contig2_All	1331	358	Complete	gb|AKO89986.1|odorant receptor 22 [*Microplitis mediator*]	4e-73	38
Or56	CL1435.Contig2_All	1200	328	Complete	gb|AKO90004.1|odorant receptor 40 [*Microplitis mediator*]	6e-34	37
Or57	CL1435.Contig3_All	1437	419	Complete	ref|NP_001229918.1|odorant receptor 115 [*Apis mellifera*]	2e-53	32
Or58	Unigene23100_All	1187	334	Complete	ref|NP_001177605.1|odorant receptor 264 [*Nasonia vitripennis*]	9e-45	30
Or59	CL1525.Contig2_All	1546	392	Complete	ref|XP_011307733.1|PREDICTED: odorant receptor 13a-like [*Fopius arisanus*]	5e-90	38
Or60	Unigene11611_All	1246	393	Complete	ref|XP_011307733.1|PREDICTED: odorant receptor 13a-like [*Fopius arisanus*]	2e-137	50
Or61	CL4022.Contig3_All	1411	391	Complete	ref|XP_011307733.1|PREDICTED: odorant receptor 13a-like [*Fopius arisanus*]	3e-67	33
Or62	CL1525.Contig1_All	1744	379	Complete	ref|XP_011307733.1|PREDICTED: odorant receptor 13a-like [*Fopius arisanus*]	9e-81	36

**Figure 4:**
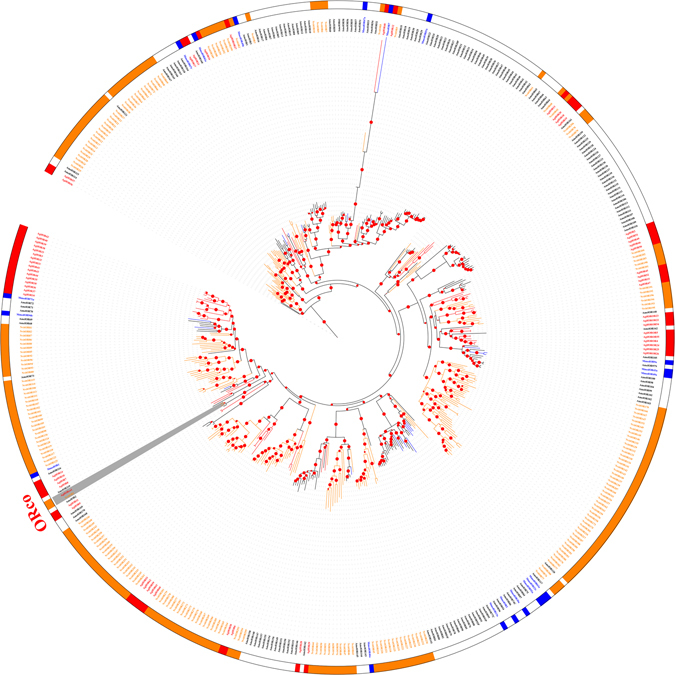
Maximum likelihood phylogenetic tree of odorant receptors (ORs). Included are ORs from *Aphidius gifuensis* (Agif), *Microplitis mediator* (Mmed), *Apis mellifera* (Amel), and *Nasonia vitripennis* (Nvit).

The odorant co-receptor in *A*. *gifuensis* was identified as having an intact open reading frame with seven transmembrane domains. With the exception of Orco, only 14 of the 62 ORs showed more than 50% identity with known ORs in the NCBI database (Table 1). The phylogenetic analysis of *A*. *gifuensis* ORs is presented in Fig. 4, which includes the identified ORs from *D*. *melanogaster*, *A*. *mellifera*, *N*. *vitripennis*, and *M*. *mediator*. The amino acid sequences for all used ORs are listed in Table S5. In the phylogenetic tree, 16* A*. *gifuensis* ORs (AgifORs) (OR3, OR30, OR31, OR32, OR33, OR34, OR35, OR36, OR37, OR38, OR39, OR40, OR41, OR42, OR43, and OR44) clustered in a species-specific subgroup, while the other AgifORs grouped with the ORs of other species. The expression of the sex-specific AgifORs with the transcriptome data-based heat map is shown in Figure S2. Orco1, OR4, OR9, OR17, OR18, OR19, OR24, OR25, OR26, OR27, OR28, OR29, OR33, OR39, and OR49 were highly expressed in both female and male antennae.

#### Gustatory receptors

**Figure 5:**
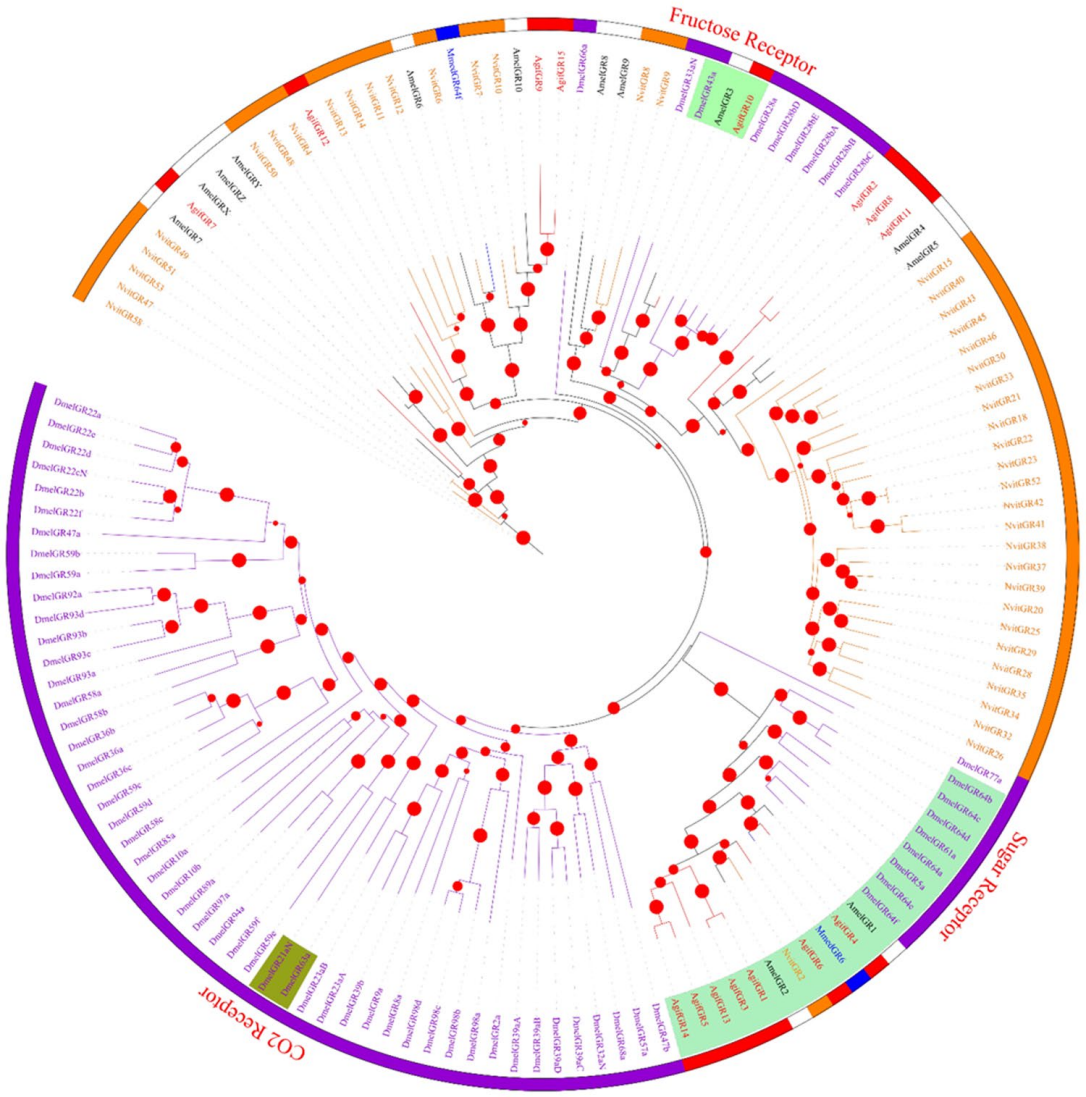
Maximum likelihood phylogenetic tree of gustatory receptors (GRs). Included are ORs from *Aphidius gifuensis* (Agif), *Microplitis mediator* (Mmed), *Apis mellifera* (Amel), *Drosophila melanogaster* (Dmel), and *Nasonia vitripennis* (Nvit).

We identified 15 candidate GRs in the *A*. *gifuensis* antennal transcriptomes (Table 2 and Fig. 3). All these candidate GRs were identified with an intact open reading frame. A phylogenetic tree was constructed with sequences from *A*. *gifuensis*, *N*. *vitripennis*, *A*. *mellifera*, and *D*. *melanogaster* (Fig. 5). Five GRs (AgifGR1, AgifGR3, AgifGR4, AgifGR5, and AgifGR6) were found in a clade with sugar receptors, which included GRs identified from *N*. *vitripennis*, *A*. *mellifera*, and *D*. *melanogaster*. The sex-specific expression of GRs can be seen in the phylogeny of all *A*. *gifuensis* GRs with the transcriptome data-based heat map (Figure S3). The expression profiles of these GRs were diverse.

**Table 2:** Candidate gustatory receptor transcripts identified in adult male and female *A*. *gifuensis* antennal transcriptomes.

Gene name	Unigene reference	Length (bp)	ORF(aa)	Status	Blast P hit	E-value	% Identify
GR1	CL1114.Contig2_All	1896	458	Complete	ref|XP_012173599.1|PREDICTED: gustatory receptor for sugar taste 64f-like [Bombus terrestris]	4e-73	34
GR2	CL1079.Contig2_All	1107	227	Complete	ref|XP_008551044.1|PREDICTED: putative gustatory receptor 28b [*Microplitis demolitor*]	6e-14	31
GR3	CL1114.Contig3_All	1790	369	Complete	ref|XP_003696536.2|PREDICTED: gustatory receptor for sugar taste 64f-like [Apis florea]	2e-56	33
GR4	Unigene11493_All	1316	412	Complete	gb|AKO90019.1|gustatory receptor 6 [*Microplitis mediator*]	0	69
GR5	CL1237.Contig6_All	1174	284	Complete	ref|XP_011304457.1|PREDICTED: gustatory receptor for sugar taste 64f-like [*Fopius arisanus*]	7e-57	40
GR6	CL2663.Contig1_All	1736	468	Complete	ref|XP_011647783.1|PREDICTED: gustatory receptor for sugar taste 64a-like [*Pogonomyrmex barbatus*]	4e-150	53
GR7	Unigene1265_All	713	200	3′,5′lost	gb|ALG36126.1|gustatory receptor 2 [Sclerodermus sp. MQW-2015]	1e-24	32
GR8	Unigene19630_All	805	234	Complete	ref|XP_011305454.1|PREDICTED: putative gustatory receptor 28b [*Fopius arisanus*]	2e-21	32
GR9	Unigene13996_All	572	190	5′lost	ref|NP_001177436.1|gustatory receptor 10 [*Nasonia vitripennis*]	3e-29	32
GR10	CL2554.Contig2_All	429	121	5′lost	ref|XP_016768876.1|PREDICTED: gustatory receptor for sugar taste 43a [*Apis mellifera*]	7e-47	65
GR11	Unigene20529_All	473	117	3′,5′lost	ref|XP_011161650.1|PREDICTED: putative gustatory receptor 28b [Solenopsis invicta]	2e-21	41
GR12	CL2232.Contig1_All	728	235	3′,5′lost	ref|XP_011314696.1|PREDICTED: gustatory receptor 68a-like [*Fopius arisanus*]	3e-18	32
GR13	Unigene13645_All	1476	453	Complete	gb|KOC62035.1|Putative gustatory receptor 64 f, partial [Habropoda laboriosa]	3e-80	38
GR14	Unigene13358_All	2101	224	5′lost	ref|XP_003705354.1|PREDICTED: gustatory receptor for sugar taste 64f-like isoform X2 [Megachile rotundata]	2e-55	49
GR15	Unigene11289_All	679	224	3′,5′lost	ref|NP_001177436.1|gustatory receptor 10 [*Nasonia vitripennis*]	2e-45	38

#### Ionotropic receptors

**Figure 6:**
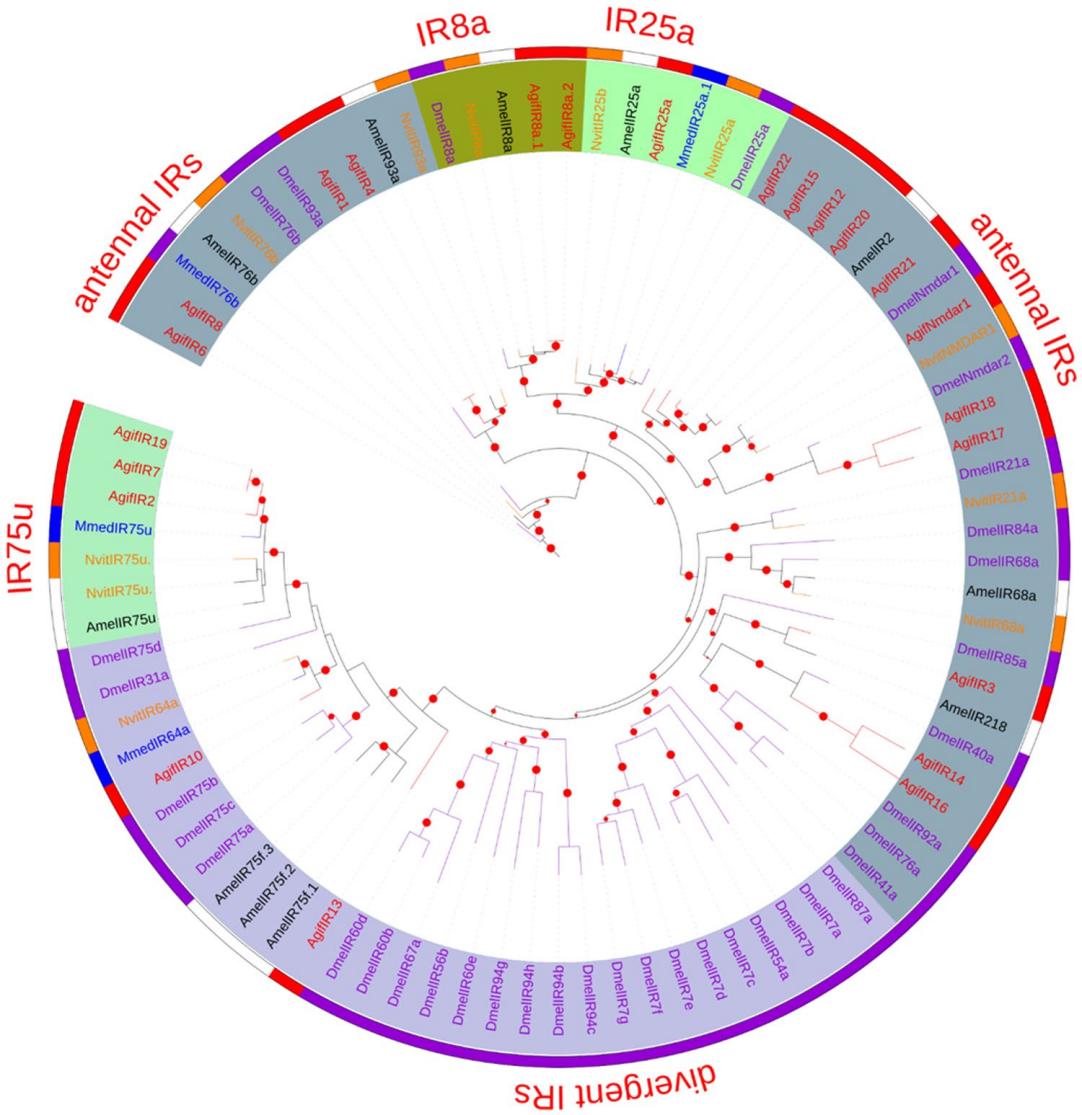
Maximum likelihood phylogenetic tree of ionotropic receptors (IRs). Included are ORs from *Aphidius gifuensis* (Agif), *Microplitis mediator* (Mmed), *Apis mellifera* (Amel), *Drosophila melanogaster* (Dmel), and *Nasonia vitripennis* (Nvit).

The identified candidate IRs in the *A*. *gifuensis* antennal transcriptome are listed in Table 3, with the best blast results from the NCBI database. In the phylogenetic tree, all IRs were classified into five clades, including antennal IRs (IR1, IR4), IR8a (IR5, IR9), IR25a, IR75u (IR2, IR7), and divergent IRs (Fig. 6). The most highly expressed IR transcripts in both male and female antennae were IR8a.1, IR8a.2, IR25s, and Nmdar1 (Figure S4). IR5, IR7, IR6, and IR9 showed significant sex-specific expression patterns.

**Table 3:** Candidate ionotropic receptor transcripts identified in adult male and female *A*. *gifuensis* antennal transcriptomes.

Gene name	Unigene reference	Length(bp)	ORF(aa)	Status	Blast P hit	E-value	% Identify
IR1	CL528.Contig10_All	3299	912	Complete	ref|XP_015125979.1| PREDICTED: glutamate receptor 4-like [*Diachasma alloeum*]	0	52
IR2	CL359.Contig5_All	2760	659	Complete	ref|XP_014299192.1|PREDICTED: glutamate receptor 2-like [*Microplitis demolitor*]	0	59
IR3	CL1108.Contig2_All	1871	611	Complete	gb|EFN83705.1| Glutamate receptor [*Harpegnathos saltator*]	0	47
IR4	CL528.Contig22_All	1533	384	Complete	gb|AID59308.1|ionotropic receptor 2 [Macrocentrus cingulum]	2e-94	47
IR8a.1 (IR5)	CL75.Contig3_All	4416	705	Complete	ref|XP_015126260.1|PREDICTED: glutamate receptor ionotropic, kainate 2 [*Diachasma alloeum*]	0	75
IR6	CL2667.Contig1_All	1881	565	Complete	gb|AKO90021.1|ionotropic receptor 76b [*Microplitis mediator*]	5e-139	40
IR7	Unigene15620_All	2779	664	Complete	gb|AKO90020.1|ionotropic receptor 75 u [*Microplitis mediator*]	0	57
IR8	CL2667.Contig4_All	1907	501	Complete	ref|XP_011301130.1|PREDICTED: glutamate receptor ionotropic, delta-2 [*Fopius arisanus*]	6e-134	44
IR8a.2 (IR9)	CL75.Contig2_All	4608	843	Complete	gb|EFN81309.1|Glutamate receptor, ionotropic kainate 5 [*Harpegnathos saltator*]	0	67
IR10	Unigene16757_All	2201	651	Complete	gb|AKO90024.1| ionotropic receptor 64a [*Microplitis mediator*]	0	46
IR25a (IR11)	CL1853.Contig2_All	3486	934	Complete	gb|AKO90023.1| ionotropic receptor 25a.1 [*Microplitis mediator*]	0	65
IR12	CL1603.Contig2_Al	1916	629	5′lost	ref|XP_011303607.1|PREDICTED: glutamate receptor ionotropic, kainate 2 isoform X4 [*Fopius arisanus*]	0	88
IR13	Unigene13567	1022	340	3′,5′lost	gb|EFN82107.1|Glutamate receptor delta-1 subunit [*Harpegnathos saltator*]	4e-135	55
IR14	Unigene16966_All	836	278	3′,5′lost	ref|XP_014298782.1|PREDICTED: glutamate receptor 1 [*Microplitis demolitor*]	8e-179	90
IR15	Unigene18475_All	731	243	3′,5′lost	gb|ALD51345.1|ionotropic glutamate receptor 4, partial [Locusta migratoria]	2e-154	88
IR16	Unigene14926	655	218	3′,5′lost	ref|XP_016905576.1| PREDICTED: glutamate receptor ionotropic, kainate 1, partial [*Apis cerana*]	8e-139	88
IR17	Unigene9783_All	575	186	3′,5′los	ref|XP_014299377.1|PREDICTED: glutamate receptor 1 isoform X1 [*Microplitis demolitor*]	3e-99	80
IR18	Unigene18816_All	768	205	3′,5′los	gb|EGI62135.1| Glutamate receptor 1 [*Acromyrmex echinatior*]	7e-80	61
IR19	Unigene15621_All	747	146	5′los	gb|EGI59906.1|Putative glutamate receptor [*Acromyrmex echinatior*]	8e-45	51
IR20	CL1603.Contig1_All	414	137	3′,5′los	gb|EFN89307.1|Glutamate receptor, ionotropic kainate 2 [*Harpegnathos saltator*]	2e-90	96
IR21	Unigene1605_All	373	122	3′,5′los	ref|XP_014297236.1|PREDICTED: glutamate receptor ionotropic, kainate 2 isoform X1 [*Microplitis demolitor*]	8e-72	91
IR22	Unigene12803_All	361	120	3′,5′los	ref|XP_014298782.1|PREDICTED: glutamate receptor 1 [*Microplitis demolitor*]	4e-64	83
Nmdar1	Unigene19690_All	2298	732	complete	ref|NP_001011573.1|NMDA receptor 1 [*Apis mellifera*]	0	79

### Tissue- and host-specific expression profile of candidate *A*. *gifuens*i*s* chemosensory receptors

**Figure 7:**
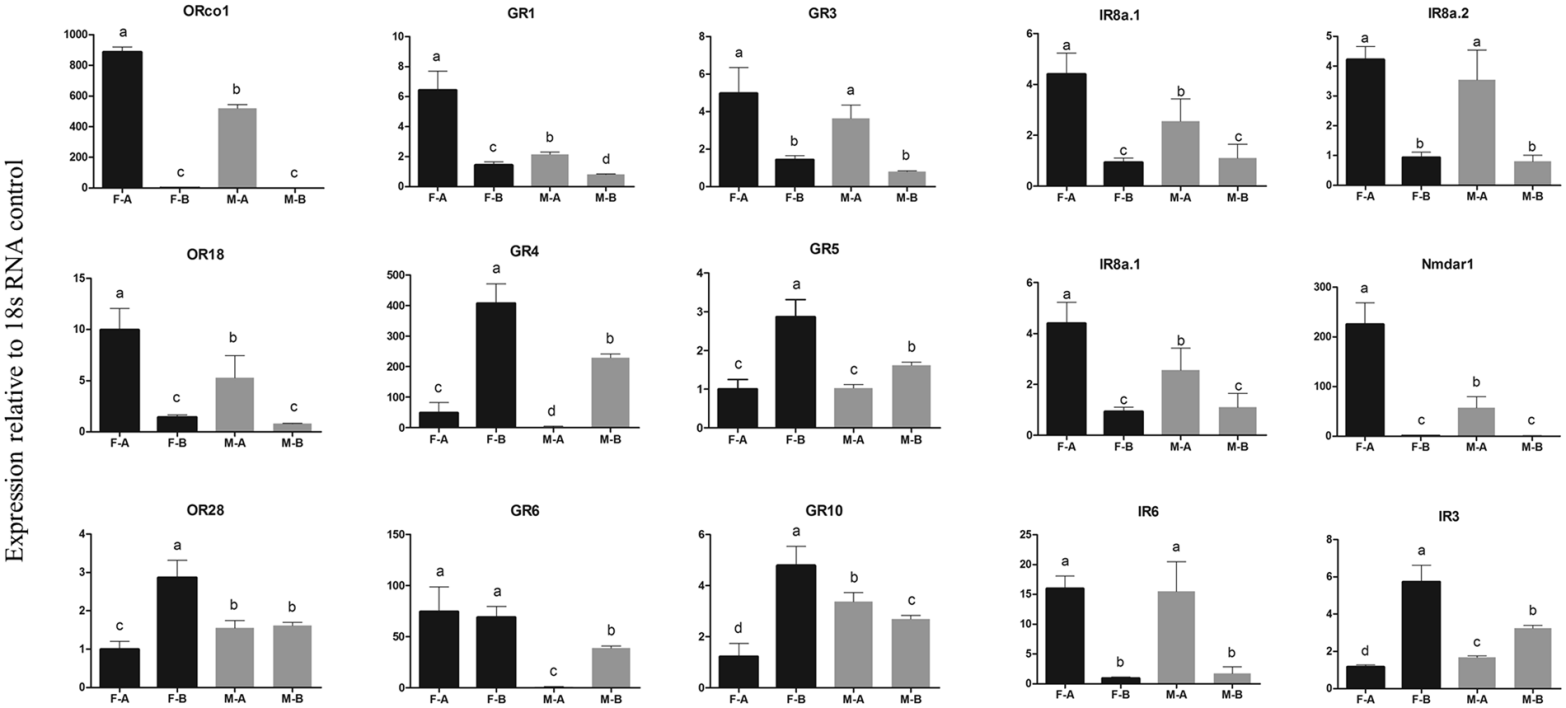
The tissue-specific transcript abundances of *Aphidius gifuensis* chemosensory receptor genes. FA: female antennae, MA: male antennae, FB: female body, MB: male body. Each bar graph in Fig. 7 represent a relative expression patterns of a chemoreceptor gene individually without any comparisons across panel. The error bars represents standard errors and the small letters above each bar indicate significant differences in transcript abundances (*p* < 0.05).

**Figure 8:**
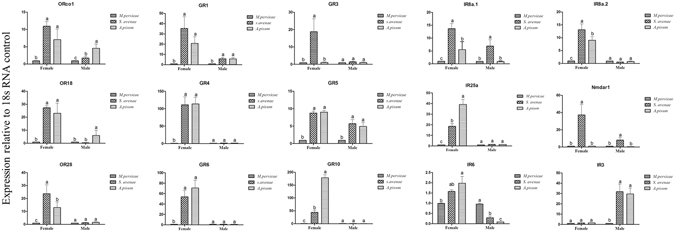
The host-specific transcript abundances of *Aphidius gifuensis* chemosensory receptor genes. *M*. *persicae*: *A*. *gifuensis* reared on the green peach aphid, *Myzus persicae*, for at least 1 year; *S*. *avenae*: *A*. *gifuensis* reared on the English grain aphid, *Sitobion avenae*, for at least 1 year; *A*. *pisum*: the *A*. *gifuensis* reared on the pea aphid *Acyrthosiphon pisum*, for at least 1 year. Each bar graph in Fig. 8 represent a relative expression patterns of a chemoreceptor gene individually without any comparisons across panel. The error bars represents standard errors and the small letters above each bar indicate significant differences in transcript abundances (*p* < 0.05).

In order to evaluate the heat map results of the chemosensory receptors and define the expression pattern of the identified genes, the expression profile of 3 ORs, 6 GRs, and 8 IRs in different tissues and hosts were analyzed using qRT-PCR, considering their sex-specificity (Figs 7 and 8). Furthermore, the tissue- and host specific will help us to have an initial functional prediction of these chemosensory genes.

All these selected target genes were successfully detected. Out of these, OR18, OR28, GR1, GR3, GR5, GR10, IR8a.1, IR8a.2, IR3, and IR6 showed a ubiquitous expression pattern in female and male tissues. Orco1, IR25a, and Nmdar1 were found to be significantly expressed in antennae, especially in females. On the contrary, GR4, GR5, and IR3 were highly expressed in the body. GR6 and GR10 showed a sex- and tissue-specific expression profile.

The English grain aphid, *Sitobin avenae*, pea aphid, *Acyrthosiphon pisum*, green peach aphid, *Myzus persicae* are the common natural pest aphids of *A*. *gifuensis* in China. The expression patterns of *A*. *gifuensis* reared on different aphid species were measured by RT-qPCR. Orco1, OR18, OR28, GR1, GR4, GR5, GR6, GR10, IR8a.1, IR8a.2, IR25a, and Nmdar1 were highly expressed in the female *A*. *gifuensis* reared on *Sitobion avenae* and *A*. *pisum*, compared with the *A*. *gifuensis* reared on *M*. *persicae*. Meanwhile, there were no significant differences in the expressions of OR28, GR3, GR4, GR6, GR10, IR8a.2, IR25a, and IR3 in the males. Compared with the *A*. *gifuensis* reared on *M*. *persicae* and *A*. *pisum*, GR3 and Nmdar1 were highly expressed in *S*. *avenae*-reared *A*. *gifuensis*.

## Discussion

In this study, we identified 62 ORs, 15 GRs, and 23 IRs in the antennal transcriptomes of *A*. *gifuensis*. Since *A*. *gifuensis* is a key biological control agent, the identified chemosensory receptors represent a valuable genomic resource at the molecular level, for aphid-plant-parasitoid interactions.

In our transcriptome, 62 ORs were identified including one odorant co-receptor. The number of identified ORs in *A*. *gifuensis* is less than that in *A*. *mellifera* and *N*. *vitripennis*, which have a total of 170, 68, and 301 ORs, respectively^21, 49^. There could be several reasons for this difference. As OR expression is amenable to modulation by scent conditioning, and the laboratory-reared *A*. *gifuensis* have had no opportunity of exposure to the diverse variety of volatiles emitted from different plants and animals, some of the olfactory genes might not be well expressed. For example, we found that Orco1, OR18, and OR28 were highly expressed in the *S*. *avenae*- and *A*. *pisum*-reared female *A*. *gifuensis*, compared with the *A*. *gifuensis* reared on *M*. *persicae*. In addition, the physiological condition of the parasitoids can also affect the expression of their chemosensory receptor genes. A previous study reported that after blood feeding, the expression of OR1 in *Anopheles gambiae* was significantly decreased^50^. This revealed that maybe the expression of some chemosensory genes were too low to be detected by transcriptome under specific physiological conditions. Meanwhile, the sequenced tissue is another restricting factor. For example, in *M*. *cingulum*, McinGR2 and McinIR7e3 are specifically expressed in other tissues, such as legs, head with mouth parts and body tissues^10^. As shown in Table S4, the number of chemosensory receptors identified based on the transcriptome is lower than that identified based on the genome. Overall, the low number of ORs identified in *A*. *gifuensis* antennal transcriptome might result from the species difference, rearing conditions, sequenced tissue, sequencing depth, and other factors.

The OR27 was highly expressed in the whole body, indicating that it not only reacts with host odors but also plays other roles in the non-olfactory organs of *A*. *gifuensis*. For example, in the migratory locust, *Locusta migratoria* L., 11 conventional ORs, which are perceived as contacting pheromones, are highly expressed in non-olfactory tissues such as wings and legs^17^.

It has been found that Orco was responsible for adopting of the correct structure by OR, and worked as a selective ion channel during olfactory signal transduction^19, 51^. In *Dendroctonus armandi*, the silencing of Orco led to EAG declining to 11 major volatiles of its host^52^. In *Aedes albopictus*, the Orco gene was found to be crucial for transmitting olfactory signals and conventional ORs that contribute directly to odorant recognition^29^. RNA interference and behavioral assays in *Locusta migratoria* L. indicated that OR-based signaling pathways mediate their attraction to aggregation pheromones^17^. In ant, ORs are candidate CHCs receptors and orco co-receptor antagonist blocks CHC detection, which are the main social communication cues in ant colonies^13, 53^. Similar to this, CHCs have been demonstrated to be involved in the discrimination of aphid species by parasitoids and the regulation of parasitism strategy^54^. For example, *A*. *gifuensis* performed different on its original host aphids and the other aphid species^55^. It laid more eggs in the new introduced aphid species than its original host aphid to improve the success rate of parasitism. In our results, ORco1, OR18 and OR28 highly expressed in *S*. *avenae* and *A*. *pisum* reared parasitoids, whereas the expression of OR28 in *A*. *pisum* reared parasitoids is lower than that in *S*. *avenae* reared parasitoids. We hypothesize that the different expression profiles among different clones might be mainly resulted from the different information cues in different aphid species. Furthermore, in *Nasonia*, female CHC profiles can be perceived as sexual cues to attract males^56^. All of these results revealed that ORs in *A*. *gifuensis* might be not only involved in its intraspecific or interspecific chemical communications.

In natural ecology, parasitoids are obligate consumers of plant-derived foods, including carbohydrate - rich solutions such as nectar and homopteran honeydew, which has been demonstrated to be an information chemical of aphid parasitoids to locate their host aphids^47, 57, 58^. For example, the honeydew is mainly containing amino acids and several carbohydrates including sucrose, glucose, trehalose, erlose, fructose, maltose and maltotriose^58, 59^. In previous work, HarmGR4 expressed in female antennae was sensitive to D-fructose in *Helicoverpa armigera* ^33^. And the behavioral and electrophysiological experiments have also found the antennae to be involved in the perception of D-fructose. In addition, DmelGr64a expressed in GRNs was found to be required for the behavioral responses to glucose, sucrose, and maltose in *D*. *melanogaster* ^60^. More interestingly, taste receptors such as GR43a, GR64a, GR32a and GR28a expressed in *Drosophila* wing respond to sweet and bitter stimuli such as glucose and denatonium^61^. In the present work, the homology genes involved in sugars perception have been identified including GR1, GR3, GR4, GR5, GR6, GR13, and GR14 and one GR (GR10) was classified as fructose receptor. Meanwhile, GR1 and GR3 were expressed predominantly in the antenna, whereas GR4 and GR5 highly expressed in body. When reared on different aphid species, the diverse expression patterns were shown. GR4, GR5, GR6 and GR10 expressed highly in *S*.*avenae* and *A*. *pisum* reared female parasitoids whereas no difference was found in that of male except GR5. The different expression patterns between male and female might related with their different food types. The male parasitoid wasps mainly feed on pollen and nectar, whereas the female parasitoid wasps can also consume honeydew and the body fluid of the aphids^57, 62, 63^. All these results suggest that sugar related GRs in *A*. *gifuensis* might be involved in the discrimination of the honeydew and nutrition quality of pest aphids by antenna and ovipositor contact. And the further researches about the functions of GR1 and GR3 in the perception of sugars and behaviors regulations during the feeding and parasitism are needed done.

The number of identified IRs in this study is greater than those for other species. For example, there are 12 IRs in *A*. *mellifera* ^21^, 12 in *N*. *vitripennis* ^49^, 11 in *M*. *mediator* ^48^, and 13 in *M*. *cingulum* ^10^. Similar to *N*. *vitripennis* (two candidate IR25a orthologs), two candidate IR8a orthologs in *A*. *gifuensis* were identified, with 75% and 67% amino acid sequence identity. However, gene duplication for IR25a has not been detected. As the gene duplication of IR25a might be unique to some of the hymenopteran species including *N*. *vitripennis* and *M*. *mediator*, further research on the loss of IR25a duplication is needed. As with the relatively high antennal expression of the OR co-receptor 1 (Orco1), the most highly expressed IR transcripts in both male and female antennae were the putative IR co-receptors, IR8a, IR25a and IR76b, in addition to IR21a, which along with IR25a, seems to be involved in the detection of small changes of temperature^38, 64, 65^. In *D*. *melanogaster*, IR25a is expressed in different populations of sensory neurons, including those in the antenna and labellum and acts as a co-receptor with different odour-sensing IRs^38^. In this work, the highly expressed IR25a in antennae indicated that it might have a similar function in the antenna of *A*. *gifuensis*. Besides these IR co-receptors, another conserved IR has been identified is IR41, which along with IR64a and IR76b are considered to play vita roles in amine sensing^66^. Furthermore, IR25a, IR93a and IR40 of *D*. *melanogaster* have been demonstrated to participate the humidity preference behavior regulation mechanism^67^. All of these results revealed that IRs in *D*. *melanogaster* with a diverse roles in the interactions between *D*. *melanogaster* and environment. In this work, the homology genes of IR40a and IR76b were identified and named as AgifIR14, AgifIR16, AigIR6 and AgifIR8. However, due to the lacking investigation of IRs in the other insects, we only hypothesized that IRs in *A*. *gifuensis* might be involved into the similar functions with their homology genes in *D*. *melanogaster*.

In conclusion, the main purpose of this work was to identify the chemosensory receptors in *A*. *gifuensis*. And RT-qPCR of some selected genes were done to reveal an initial functional predication, which were supported by the functional investigation of their homology genes in other insects. Our results not only lay a solid foundation on the further investigation about the functions of these identified genes in *A*. *gifuensis* such as the CHCs discrimination, odor and sugar perceptions but also provide valuable information for understanding and investigating the intraspecific or interspecific chemical communications in the solitary parasitic wasps.

## Materials and Methods

### Insects rearing

*A*. *gifuensis* were collected from the pea aphid, *Acyrthosiphon pisum* Harris, which were reared on alfalfa. A laboratory colony was established and maintained at 21 °C with a 16 h light: 8 h dark photoperiod on *A*. *pisum* that were reared on broad bean (*Vicia faba* L., var. ‘Jingxuancandou’, Jinnong, Taigu, Shanxi, China).

For providing a host-specific experience, the *A*. *gifuensis* were reared on *A*. *pisum*, the green peach aphid *Myzus persicae*, and the English grain aphid *Sitobion avenae*, for at least one year.

### RNA sequencing

Antennae of *A*. *gifuensis* were cut from newly emerged adult male or female wasps (1–2 days old) respectively, and were frozen in liquid nitrogen. This collection of antennae without any other tissues was immediately stored at −80 °C for further analysis. Total RNA was extracted from four hundred antennae of each sex for each replicate using TRIzol reagent (Takara Bio, Tokyo, Japan), as per manufacturer’s instructions. And there were three biological replicates for each sex. The RNA integrity was verified by 1% agarose gel electrophoresis and the quantity was assessed using a Nanodrop ND-2000 spectrophotometer. Synthesis of cDNA and Illumina library generation was completed at Beijing Genomics Institute (BGI) (Shenzhen, Shenzhen, Guangdong, China), using Illumina HiSeq^TM^2000 sequencing.

### De novo Assembly and Gene Annotation

Transcriptome de novo assembly was carried out using a short reads assembling program—Trinity, which combines three independent software modules: Inchworm, Chrysalis, and Butterfly, to overcome the quality and polymorphism issues. In order to get comprehensive information about the genes, we aligned the unigenes larger than 150 bp to Nr, Nt, KEGG, Swiss-Prot, and COG databases, with e-value < 10^−5^. With Nr annotation, we used the Blast2GO program to get GO annotation of Unigenes. Next, the WEGO software was used to perform GO functional classification for all unigenes.

The unigene expression levels were calculated by fragments per kb per million reads (FPKM) method, using the formula, FPKM (A) = 10^3^ (10^6^ C)/NL. FPKM (A) was set as the expression level of Unigene A, and C was the number of fragments that uniquely aligned to Unigene A, N was the total number of fragments that uniquely aligned to all Unigenes, and L was the base number in the CDS of Unigene A. The FPKM method is able to eliminate the influence of different gene length and sequencing level on the calculation of gene expression. Therefore, the calculated gene expression can be directly used for comparing the differences in gene expression across samples.

### Phylogenetic analysis of candidate chemosensory receptors

Amino acid sequences of the candidate ORs, GRs, or IRs were aligned using MAFFT, with FFT-NS-I iterative refinement method with JTT200 scoring matrix, unalignlevel 0.3, “leave gappy regions” set, and other default parameters. Bioedit Sequence Alignment Editor 7.1.3.0 (Ibis Pharmaceuticals, Inc., Carlsbad, CA, USA) was used for further manual editing. Phylogenetic trees were subsequently constructed by the Maximum likelihood (ML) method using PhyML3.1, based on the best-fit model LG + G estimated by ProtTest2.4. SH-like approximate likelihood ratio (aLRT-SH) supports were used to evaluate the reliability of internal branches. The trees were further edited using the ITOL tool. The identity scores of alignment were extracted using BioEdit software, and the heat map was constructed by ITOL based on a three-color scale. Phylogenetic trees were based on hymenopteran data sets. The OR data set contained 62 amino acid sequences from *A*. *gifuensis*, together with *N*. *vitripennis* (67), *M*. *mediator* (51), and *A*. *mellifera* (68). The GR dataset contained 6 amino acid sequences from *A*. *gifuensis*, together with sequences from *N*. *vitripennis* (67), *A*. *mellifera* (68), and *D*. *melanogaster* (95). The IR data set contained 9 *A*. *gifuensis* amino acid sequences, along with *M*. *mediator* (51), *N*. *vitripennis* (67), *A*. *mellifera* (68), and *D*. *melanogaster* (95) IR sequences. All amino acid sequences for the chemosensory receptors used in this study are shown in Table S5.

### Chemosensory receptors in different insect species

The species phylogenetic tree was constructed based on the alignment results of cytochrome oxidase subunit I (COI) genes from different species, using Mega 6. The trees were further edited using the ITOL tool with the number of identified chemosensory receptors. The number of identified chemosensory genes in all insects is shown in Table S4. The number of chemosensory-related genes was collected from published papers. The GenBank numbers of COI are *Microplitis mediator* (GenBank ID: KJ459149.1), *Acyrthosiphon pisum* (GenBank ID: AB506720.1), *Apis mellifera* (GenBank ID: AY114465.1), *Nasonia vitripennis* (GenBank ID: EU746554.1), *Solenopsis invicta* (GenBank ID: JN808838.1), *Bombyx mori* (GenBank ID: EU141360.1), *Tribolium castaneum* (GenBank ID: KJ003352.1), *Drosophila melanogaster* (GenBank ID: KJ767244.1), *Anopheles gambiae* (GenBank ID: DQ465336.1), *Aedes aegypti* (GenBank ID: GQ165783.1), *Aphidius gifuensis* (GenBank ID: GU097658.1), and *Culex quinquefasciatus* (GenBank ID: GQ165766.1)

### Expression analysis

Heatmap plots were generated for the binary logarithm of raw FPKM-plus 1 values. For each plot, the minimum value was set to the number type, with a value of zero, and displayed as yellow, the midpoint was set to percentile type, with a value of 100, and displayed as blue, and the maximum was set to the highest value type, and displayed as red. These plots were made and edited using ITOL tool.

Quantitative reverse transcription PCR was performed to validate the expression of candidate chemosensory receptors in *A*. *gifuensis*. The collection of antennae and body tissues without antennae of each sex were collected respectively (antennae: 400 of each sex; body tissues: 20 of each sex) and were frozen in liquid nitrogen. For the host aphid specific expression analysis, *A*. *gifuensis* reared on different aphids were collected (whole body and 20 of each sex) and frozen in liquid nitrogen. Total RNA of *A*. *gifuensis* was extracted using TRIzol reagent (Takara Bio, Tokyo, Japan), as per manufacturer’s instructions. The temple RNA was treated using Dnase I and incubated at 42 °C for 2 min to remove the genomic DNA. Next, the cDNA was synthesized from total RNA using Transcriptor First Strand cDNA Synthesis Kit (Roche Diagnostics, Mannheim, Germany) according to the standard manufacturer’s protocol. Gene-specific primers were designed by Primer Premier 5 (PREMIER Biosoft International, Palo Alto, CA, USA), and are shown in Table S3. qPCR was conducted in 20 μl reactions containing 50 × SYBR Premix, Ex Taq (10 μL), primer (10 mM), sample cDNA (0.8 μL), and sterilized ultra-pure grade H_2_O (7.6 μL). Cycling conditions were 95 °C for 30 s, 40 cycles of 95 °C for 5 s, and 55 °C for 30 s. Each sample had three technical replicates and three biological replicates. Relative quantification was performed using the Comparative 2^−ΔΔCT^ method. Transcription levels of these receptor genes were normalized by 18 S RNA, and the normalization of each gene was compared with the lowest expression level in different tissues^68^. The expression data among the different tissues and host aphids of each sex were subjected to one-way analysis of variance (ANOVA); means were separated using Duncan’s test at *P * < 0.05.

## Electronic supplementary material

SUPPLEMENTARY INFO
