# Supplementary material for: Identification and expression analysis of chemosensory receptor genes in an aphid endoparasitoid Aphidius gifuensis
Source: Sci Rep. 2017 Jun 21;7:3939. doi: 10.1038/s41598-017-03988-z (PMC5479799; doi:10.1038/s41598-017-03988-z)
Supplement: Supplementary file 1 — SUPPLEMENTARY INFO [file 41598_2017_3988_MOESM1_ESM.pdf]

The identification and expression analysis of chemosensory receptor genes in an aphid endoparasitoid *Aphidius gifuensis*.

Zhi-Wei Kang[1,#], Hong-Gang Tian[1,#], Fang-Hua Liu[1,2], Xiang Liu[1,3], Xiang-Feng Jing[1], Tong-Xian Liu[1,*]

1. State Key Laboratory of Crop Stress Biology for the Arid Areas, and Key Laboratory of Northwest Loess Plateau Crop Pest Management of Ministry of Agriculture, Northwest A&F University, Yangling, Shaanxi, 712100, China

2. State Key Laboratory of Integrated Management of Pest and Rodents, Institute of Zoology, Chinese Academy of Sciences, Beijing, 100101, China

3. Entomology Department, College of Plant Protection, Yunnan Agricultural University, Kunming 650201, China

Email:

Z.-W. Kang: zwkang2010@126.com

F.-H. Liu: liufanghua5@163.com

H.-G. Tian: tianhg@nwsuaf.edu.cn

X. Liu: 15192226343@126.com

X.-F. Jing: jxf_zb@sina.cn

Corresponding author: Tong-Xian Liu; State Key Laboratory of Crop Stress Biology for Arid Areas, Northwest A&F University, Yangling, Shaanxi, China; 86-029-87092663; E-mail: txliu@nwsuaf.edu.cn

Keywords: *Aphidius gifuensis*; chemosensory receptor gene; odorant receptor, gustatory receptor, ionotropic receptor; expression pattern

Short title: chemosensory receptor genes of *Aphidius gifuensis*

Footnotes

# These authors contributed equally to this work.

*To whom correspondence should be addressed. E-mail：T.-X.L. (E-mail: txliu@nwsuaf.edu.cn)

Author Contributions Z.-W.K., H.-G.T. and T.-X.L. conceived and designed the experiments. Z.-W.K., F.-H.L. and X.L. performed the experiments. Z.-W.K., F.-H.L. and X.L. analyzed the data. Z.-W.K., F.-H.L., X.L., H.-G.T. and T.-X.L. contributed reagents/materials/analysis tools. Z.-W.K., H.-G.T., X.-F.J. and T.-X.L. wrote the paper.

The authors declare no conflict of interest.

The authors declare no competing financial interests.

Summary of supplementary material

Supporting Tables

Table S1 page

Table S2 page

Table S3 page

Table S4 page

Table S5 page

Supporting Figures

Figure S1 page

Figure S2 page

Figure S3 page

Figure S4 page

Legends of Supplemental Tables

Table S1. Assembly summary of the A. gifuensis antennal transcriptome.

Table S2. Summary of A. gifuensis antennal transcriptome annotation.

Table S3. The primer used in this study.

Table S4. The summary of the identified chemosensory receptors in insects.

Table S5. The amino acid sequences of chemosensory receptors used in the present study.

Table S1

Complete Assembly		Protein-Coding Components	
		All Isoforms	Representative Isoforms
Total size	45,760M	41,541M	21,980M
Number of components	28,461	19,074	
Number of transcripts	38,848	28,390	19,074
Longest transcript	13,876bp	13,876bp	13,274bp
N50 transcript length	1,980bp	2,162bp	1,688bp
GC content	31.12%	31.54%	30.87%

Table S2

Database	Numbers of Unigenes	Percentage
NR_Annotation	26,969	92.0%
Nt_Annotation	21,411	73.0%
COG_annotation	11,086	37.8%
Swiss-Prot_Annotation	22,259	78.0%
GO_Annotation	12,552	42.8%
KEGG _Annotation	20,319	69.3%
All databases_Annotation	29,302	

Table S3

Type	Gene name	Primers
Odorant receptor	ORco1	F: ACAAAGAATCAAGGACTTGTGTCCG
		R: ACCAGTCCAACAAAGTGTTGACAGT
	OR18	F: AACAGGCTACAGTAAAATGGAACGA
		R: CATGCAAACACTTAAAGTGTCGACA
	OR28	F: TGCTCAATTTTTTAACTCTCTGGCA
		R: CAATGCACCACCATATGACAAACT
Gustatory receptor	GR1	F: CAGATTCTTTTCATTTTCCACCAAG
		R: ATCCACATTTGTGAACGGCAA
	GR3	F: GGACGGTGTCAACAATCCAATT
		R: CTCCATTTTCATTGTCAGCTCTGA
	GR4	F: ATTCACGTAAAATGATAAACACCGG
		R: CTTATGGCAATTTCACGTTCAACTT
	GR5	F: TTTACGATAATCGCTGTTCTTGAGC
		R: TTGACGAGATCACTTAGAATCGCAT
	GR6	F: ATTACAAATCAGCAAAAGTCCTCGA
		R: TGCAGCACTTATACCTTCCTGAAGA
	GR10	F: GGTTGTTCAACCATGCTATGCAAC
		R: CGTAACAGCACCAGCCATAGATGTA
Ionotropic receptor	Ir8a.1	F: AGAAAGTTTTCAGAAAGTGTGCAGC
		R: CCGTTCTGAAGAGGATTTCCATTTT
	Ir8a.2	F: CACCATAAGTAAACCATGCACATTG
		R: AGAAAGTTTTCAGAAAGTGTGCAGC
	IR25a	F: GCAACAGTCAATACCAAAAGGACCC
		R: CTTCATGTGGCTCTGCACCATTTA
	Nmdar1	F: ATCAGTGTTGGAATTTGAGCCAGG
		R: CAATCCAAACATATCCAGCACCAG
	IR6	F: GACGGGAATTGATTTAATGCTTATG
		R: GCAACAAACCAATGACACCCTTTT
	IR3	F: TTCCATTTTCACTTTGTTGCAATGC
		R: TGCAGTTGACGACCTTGAAGATCTA

Table S4

Species	Odorant receptor	Ionotropic receptor	Gustatory receptor
Bombyx mori	72	11	56
Chilo suppressalis	47	20	NA
Helicoverpa assulta	64	19	NA
Dendrolimus houi	33	10	NA
Dendrolimus kikuchii	33	9	NA
Plutella xylostell	53	16	NA
Manduca sexta	47	6	NA
Helicoverpa armigera	60	19	NA
Spodoptera littoralis	47	17	NA
Sesamia inferens (Walker)	39	3	NA
Agrotis ipsilon	35	14	NA
Ostrinia furnacalis (Guenée)	56	NA	NA
Sesamia nonagrioides	13	15	NA
Grapholita molesta	48	24	4
Spodoptera exigua	10	6	6
Spodoptera litura	26	9	NA
Cnaphalocrocis medinalis	29	15	NA
Heliconius melpomene	68	NA	72
Locusta migratoria	142	32	75
Pediculus humanus	10	12	8
Colaphellus bowringi	43	9	10
Anomala corpulenta Motschulsky	43	5	8
Tribolium castaneum	265	23	220
Leptinotarsa decemlineata	37	10	NA
Monochamus alternatus	10	8	2
Dastarcus helophoroides	10	8	2
Tenebrio molitor	20	6	NA

Ips typographus	43	7	6
Dendroctonus ponderosae	49	15	2
Dendroctonus valens	22	3	4
Apis mellifera	163	10	10
Bombus terrestris	166	21	25
Apis cerana	119	10	10
Linepithema humile	337	97	32
Camponotus floridanus	352	46	31
Harpegnathos saltator	247	17	23
Pogonomyrmex barbatus	344	61	24
Atta vollenweideri	70	7	2
Atta cephalotes	215	21	25
Solenopsis invicta	333	17	219
Microplitis mediator	60	6	2
Nasonia vitripennis	225	47	10
Acyrthosiphon pisum	48	11	53
Euglossa dilemma	86	4	2
Euglossa viridissima	85	5	4
Nilaparvata lugens	50	NA	10
Drosophila melanogaster	62	66	73
Drosophila simulans	63	67	62
Drosophila sechellia	57	56	53
Drosophila yakuba	65	65	63
Drosophila erecta	62	63	54
Drosophila ananassae	68	62	64
Drosophila pseudoobscura	67	58	52
Drosophila willistoni	71	60	68
Drosophila mojavensis	62	56	50
Drosophila virilis	56	54	50
Drosophila grimshawi	66	62	66
Drosophila ananassae	64	46	62

Anopheles gambiae	75	46	61
Culex quinquefasciatus	178	69	65
Aedes aegypti	127	95	70
Calliphora stygia (Fabricius)	50	22	21
Bactrocera dorsalis	23	11	6
Toxorhynchites mboinensis	87	38	21
Mayetiola destructor (Say)	122	39	28
Macrocentrus cingulum	79	13	20
Cydia pomonella	58	21	20
Conogethes punctiferalis	46	7	NA
Chouioia cunea	16	5	17

78

Table S5

Odorant receptor:

>AgifORco1
MMKTKNQGLVSDLMPNIRVMQMSGHFMFNYYSEGKKFLHKIWCCINLFLVVLQFAFCGI
NLIMESDDVDDLTANTITILFFLHSIVKIIYFAARSKLFYRTLAIWNNPNSHPLFAESNARYH
SIALTKNRRLLFFIGAVTVLSTLCWTGITFVGESVKRRIDPDTNETMIIPIPRLMVRSFYPFD
ASHGFLHIAMLAFQFYWLFICMVDANSLDVLFCSWLLFACEQLQHLKAIMKPLMELSATL
DTVVPNSSELFKAGSADHLRDTTGTAPPATPANGDNMLDLDLRGIYSNRQDFTATFRQTA
GMQYNGGVGPNGLTKKQEMLVRSAIKYWVERHKHVVRLVTAIGDAYGVALLLHMLVTTI
TLTLLAYQATKVNGINVYAATTIGYLLYALGQVFLFCIFGNRLIEESSSVMEAAYSCHWYD
GSEEAKTFVQIVCQQCQKAMSISGAKFFTVSLDLFASVLGAVVTYFMVLVQLK
>AgifOR2
MVFFDNKNWLLTKVLLSVVGAWPFQSQQTRIVLGFSSILIILSLLIPEIFGLIKERQNSSTVIE
CATIMMLHVAMLVYLTHVLLNHKKMKQLLMEIEQFWNSTLLKNEICILEKLTLANRKFTH
SYIWFVLVSTVLFASLPIIPKIMDIIVPLNESRPTIFIYQSEYFIDPIKYENLIFIHSYIGTMYPTI
VLVGYDSLYSNLAQHSSCMFEIISNHVKNTQLADKNIYGDDNSLSQYDYDAIFYIDCIQRH
RVAQKFTSTIKSVYNIPLFFMLFLNMIAVSLAGCQVLLTSDQPSQAMRFFMNAACALIHLY
FLSWVGQKVIDSSSKLYQSIYQSEWYQANNTTQKFLIIFMMACREPCNLTAGKLWILSSEN
FTVVLKTAVSCFTLFRSTQ
>AgifOR3
MQLLQESFFILKCTGLWKPASCYGWRSWLYNLYIFIIRFNLWMFVITQSLALVLTSNSIEDF
TNILFLLLSVLVVCGKINNMLSGRKLMDKLINSFNKYPFKPETDDEINIQNRFNRINRINTIL
YWAINGITAAVWCIRILLDLKPTYILPYGGWFPYDHTKPIIYWFTAVSQLMAPVMAANMT
GGYDSFFCGLMMQLCAQVEILKHRFNNKLDNLEKKNLINLNPSVHKNIEKKIFSDIIEHHI
AILRMAYDVNSFFSKIVFLQYSLSSIVLVASVYILAGLKPGSLQFTSGLIYLLCMFYQIALLC
TSGHEISLAFEELGDSIYNSNWLNLTSNKSQKNMIIMMSRMLRPFVIKSGHFVTLNMDSLK
YLVKLSYSIYNLL
>AgifOROR4
MDTSTPSYLAINKLLASLSGVWPHQPIVTKNIVFIIMLILTISQSYFQIYGAILARSNVDLFLE
TIPNILVDFSVAAKVVNCFFNSKKMKKLLITLEKDWIKFNSEGEIQILNEHGVRARKLTLTY
FSVICGTITPFMVIPLVPIIYNNFAPANGTLPKQMLYAQYDYLFNLQVSYYPVLIHSYIATFAF
INDIIAIDTMCMVFVQHGCALFSIIGYNLEQIEINSTIKNDINHSRFNDKAYHQMSECIRRHN
HALKYAKLIEDAHNVSYFFQIGIDMICFSFTGFQIVDKLDTAPDVSIRFAAFTSTQLAGLFLL
SYPAQQLFDYSVKIYDDIYNAGWYKTSLRTRKLFIIMSMRSLIPCQITAGKFYVLDLANFG
KIVKTSLSYITVLISMK
>AgifOROR5
MKKATSMETPQYLRFNKNLLACAGLWPHQQKLSKIIFFIILLFLIISQTAFQIIGAILARENV
DLFMEAIPAILGGIAIAVKIMNFYLNGHKMKKLLVLIQNDWIEYKNGPESLILKKYAKFSN
KLTLMYFGIIWSTMTPFLTIPLVPLIFNIFVSQNNSIQRQHLYAQYEYVFDSDKNYYPVLIHS
YIGSFAFINAVVAIDAMITMYIEHACALFSIIGYNLQVMQNNHDVDVNLNPHEINDGAFKN
VINCIKKHDRVLQYVKLLDNANSISYFFQLGIDIICLSFTGFQIATKIDNSPDAAMRFAAYST

SLLVVLYFLSYPGQQLIDHSLKICHNIYKANWYCTSLRTRKLLIFMAMRSSIPCQITAGKLY
VMDITSFKLVVKTSISYVMVLLSVQE
>AgifOROR6
MDNSNNWIKFEKLLNLEKYILQIFGLRSMKTLFLNKPHAKNIYETIYFSIPLTIMIGLIFLNIL
YVLSVWKDEVDSAINMISTIFNQFVVIPKGIIIWFYQEELYNILNNLHEKWNTSFTRTEIRD
QILKRAKKITKFRNIYVFSFAILTITYMYPPIRNSLIYFNQNTNNSLDYSQTMWPTEVYPIKI
DTFFKYLCISTIEICQAFFYIIYCHVGDIFYIQIFTHISIQFQVLANDVITTVETKKNGNSKTLL
IELISIVMRHQELYELIDKMNNQLYSPVIFVTFIIGSINICLNIFEFQRAIDKGNYYDASRNVIL
TVSICFGIIIYCVQAQIICESAELIPEAIYNSQWSNIDKNSILTFQIIMSRCQQKLTCGAYGLVN
IDHEQITQVCRRTASFFVFLDSIQ
>AgifOROR7
MRTSYSNAAVHRRIADASILGMKFSGFWNFSNNPNTIEKLFNLIYKYIMKLIIYTFATTLVA
DLISNTDDLKIFIDDGCFFAGIFVIIFKLTTFGIYENNINELIEKIFKPIDYLKNLNDKDVTTLI
NENIFFERCTAYGFASLAVCLLITMIFFGNHKDGELPIRAKYPFNSTEGINYTTAFTIQATGV
TIGLTGIMAMDTIVLGLCRWTKFQFDVLYSNFQHCGDDIANSKKQQQKKLINNDKTGHN
NSEIFKNFVAFNSNENHHLGFSERFKICIKNHQRLIGIIESVNNLFGLNMFVQICGSSFIICFA
GYQALLGSNGMRDLMKFSCYCGTGFSQLFIWSYFGNNLLCQGDLLSDGQWFSGWEKQN
SRDLKNLLTIPMIRTRKPLELKAMNFFTMSIETFMMILRASYSIFTLLRTMEMK
>AgifOROR8
MVKQFLHRKILNMQIDGLRLCGFWSLNPTLPAYVKILYYVYNKVVLSFVLIFIGTLTADLII
NFNDLLIVTDDGCYLAGMMVILFKIYIFNDKQEKINQLVEKVYQPMDIIAKSSDPGVRSRL
RISNFLEQLLFYFFIGLAIFLAFALLVLVPRQDGELPIRSAFPFNTKISPKHEIGYFLQSAAICY
GLFLIVCMDSMVTEMGRWINFRMKILTSNFRMCDSKQHDRGAFYSTDATFEFLKNTSIYQI
TDEHQKIRSFIPIKLDEVNNFEDSYQLRFKTCLQNHQDIIHAVDDLNAIFGSSMLMQLFASF
SMICLTGFQAVLGSTSKTSLMKFAVYLGAAFSQLANWCWIGNEISYESTILFESQWLSGWE
KQISDPSIRTLMTIPLIRTKKPLQLKAGKFYTMSMTTFTGILRNSYQFFALLTTATDAQRQ
>AgifOROR9
MVEFKEHIKKEDLFKFETYLSRLFSILCFMKISPSYSELSFIKKLCSTCFVIISLGCATSGCILQ
IVSFKDVRDMPSFSQRVIELCFIGLGTYKWYHCILKFDSLKKIIELLNNCHQVGYLINNNDK
EYKIYHQEMLDAKKASEYFMIVWFPFTMLGVVQWCLTPLTSDFYINKDIDRNNKNMTLL
RSYPNGATFPIEIDTLTKYLLMFLFQSLGILFSAIGMSGCDVILISIIMYICAHLRYLNNCLIDE
NNIQLILKSKNPSEKLKEKLKNCVIFHDQILKVHENYLSYSNGAMFAQCIENIIALCLVSLQ
ASTMHYSPGIDFIVAFTSMIEYSMGIFIELFAFCYYGTKLEELGHEVSDSFFSSNWEKCNDK
IQIKIQNILKLGIMRSQKPIIITGGPFYIFNLETFKKLAYMSISNSVVLRQLQTTD
>AgifOROR10
MTSHEYKPINAFKINLNIFTAEGIWPAGL
KNKNLRYPYAVYRLLSPSIWFGSFFIFQLIQVIIVINDMAKLMDSMYLMVTYFAILSKFMCF
ILYRKRIEKLFNTLDESIFIPRQKKHFDYINEAMRIIRRDTIFFLSSGISATIFWTFYPLFDKHQ
EEKQLAYVMWCPIDVSTSPIFELVYTYQVIAITYNTMFNTMADTTICGLFRMMSGHLEILA
QDYSELFDDNFISPGDIVDKKNNINKGLSNYKNDNEDIDEDKYLIVKKPNVTHEKLSYRVA
ACVEYAQAIEKFVNEVNDVFQGGILTQFIASCLIICATAFALTMVPVASVQFVSLLQYQGCM
IIQIFIYCWRGNELTLKFSELSNAIFKCNWQLCNGHFRRSMDIIMSRSQKPMILVVAGLFSLS
VDTFIGIIKSAYSFFMFLKEVQTTAAED
>AgifOROR11

MVEFKEHIKKEDLFKFETYITRLFSILCFMKISPSYSEISFIKKLCSTCFVIISLGCSTSGCILQI
MSFRNVRDMPSFSQRVIELCFMGLGTYKWYHCILKFDALKEIIKLLNNCHQVGYLINNND
KEYKSYHQEMLDAKKASEYFMIVWFPFTILGVVQWCLTPLTSDFYINKDIDRNNKNISLLR
SYPNGATFPIEIDTLTKYLLMFLFQSLGILFSAIGISGCDVILISLIMYICAHLRYLNNCLIDEN
NIQLILKSKNPSEKLKEKLKHCVIYHDQILKVHENYLSYSNGAMFAQCIENIIALCLVSLQA
STMHYSPGIDFIVAFTSMIEYSMGISIELFAFCYYGTKLEELGHEVSDSFFFSNWDKLKIDK
KCQDIQDKIQNIIKLGIMRAQKPIIITGGPFYILNLETFKTLAYMSISNAIVLRQMADG
>AgifOROR12
MKNKSQSIPASARKNIRYSTELSRWVLTILGIWSNKNNKNIINQILSIVLIFMCYFIICFSLVP
CGLYTFISEKDIHLKMKAIGPLSFCVMALSKYFLLVTRKKDIYNCFKHIYHDWRQVQLIND
ENIMLENAGTGRFITIICAAFMYGGGFFYNTIMPFAVGSFVTADNKTIKPITYPVYDPLFSA
QETPNYQIVFTLQWVSGFIMYSITIGACNLAAVFAFHACGQLKIVISRLDNFVDRNHNYDD
VLKNKMGVIIELHLRTIRFILRVEDILNELNLIEFLGCTFNICMLVYYFMSEFEKAETISTITY
CVLLVSFTFNIFIFCYIGEMLTQQGKKLGLAAYNNMNWYELTTKNSRGLILIMAMSNCPCS
LSAGKMVNLSYASFASVIKTAMTYLNIIRTTVL
>AgifOROR13
MFKIVNNAINDWQSVLNHESKNIMKKFAKKARKVFFYQMGFSFIIINMMILDSLPVSHDN
LNLTADSQLLNSFPMKTTYYFGYIELSWYKYTALYFLQIIQLIFCVIGNVGNDCYFFGMAM
HLSGQFVTLQLQTKDFETEINKNDNCTNYIENFVEKHQHLIDIGNCLKDSFNSILIVELVNN
ACSILMMGIQMILNIQSGDNVMVADAIVIILVVIMQLFLYCHAGEELKHYFESLRFALCECS
WYNMPNYNVKNILFIMMRTSKTFNLTAGSIYHINMDTLTKILKAMGSYFSVLQALFIK
>AgifOROR14
MVASTFLLMTNSVHAYKICVIIKNQKRIQLLFDTISSELFTKNFDKYERIFTWYAWSGIKHH
IAYQSFGTMAVFCWGFVPISNAITGNERQLPMDGWYPYDTKLSPAFELTCGHQAVAVIIAC
CHNIGMDTLINGFINVACCQLEIIKQNILSIDDGDKNVHQEINQSVQHSVVVMKFIDEVQSI
FGTVIMYQLIANCLIICLTAFHVVQMTVYIPAEIFGMMMYMCCMTYQIFIFCWNGNELTIQ
SETLAFTAITSNWWKFDKKYKRSLGILITRSHKPMIFKAGPLINLSLQTFMKVLRMSYSFFT
LLQSSTNPH
>AgifOROR15
MRCMARTEFISSIHCSFFIVGWDGNNYDTRTWVYGSFMEDFLPCIPTQLLITAVAAKYVVF
AIYKKTFQDTIDKMRVDFEFFGNTPARKILKEYADKGRTRTIFYGCFLFMILTTFVVPAFLP
VVLDKFRPLNETRIRLKLLDTDYGVDPDEYFVLITIHGYIASTFLVLMLWSVDSFIMIMVQ
HCCSLFVIVGFTLKKLDQGNKKHTEKFENDVLINALQVHQQAIEFADFIEASLKNMYGIVII
ENMLAISITGLETIKQMDQTDQAMRFAAFAFGQMVHLFFNSLPGQELVDHSTGLFDLVYD
CQWESLSIKSKKKILFILMRSAKPKSLTAGGFYELNLQHCGAVLKTSMSYLTVLSSMRQ
>AgifOROR16
MAFTQILVRIAMLKKYNRKLGDIINKAIKDYDYKLYKTTEEKKVFIDYIKRAKLFCKLLCIF
VAMTASSYYVKPITSPPPQPPPSSTINDDDFIINNLTKSFILPYRFYLFHQVNDFKMYVITYV
SQFPFVFVSGFGQTAADCLMVSLVFHICGKLAVLSIKISTINPNINFCKKQLNEIIIEHNRLLK
MGQDIEEAFSETLLIHLVGATSLVCILGYQLLTNYAKGQNADLATFLIFIFLVFLILYAHCVV
GESLITESNKVCEAYYACQWYNMPHDTSRGIILCMIRSQKPLGLSAGKFKTLCLSTLTEVL
KTAMGYLSVLRSFLAV
>AgifOROR17
MNISEDDDNKKVEEIQEEFEHLNDLLDWNRKFLIFGGIWPLENTKFRAAIFTAYMTFHLFT

EYSGVVEVVGNFEAMVLGIIESSMQSMVLSKLFVFRHSKNLRVLISAMKEDFQENNYNDL
NEKKIYLKYNYYSKLYFKLSVPYVLTIAALYYARPLLTSLILGTFGVNDSLILPFQTKLFFPI
DNTQTYFYVYIWYCPMVYLLACHNAFICILITIILHICGQLAVVEYRIKNLKYDHEKDHNQ
MIFKTLVQRHQRSIWMAKTFDTSFNVVLLIDLIGSTVLIGLLSYSIVTESETLETSFVYIYIC
AIIATLFLLFGYCYVGECLVENSTRVHEVYYECEWHKMSLKFQKSLSFCLMETEKPLTMTA
GKIFVFSLSGYAYVIKSAMGYVSMLRNVSM
>AgifOROR18
MYEAKGNKEIKQATVKWNEDTIYALGLYKRVTTLVGIWPTNNRILTGIQIGVYITFHLSSVF
IFFQKFLEYGNCGTTMEIVDTLSVCMASVQICIKSFIAWFKNHEFRHVVDSAVMDWSCVE
EEQSHSVMLVYARKGRVLLMCQMAVGFSTAFPLMLDRFPKIVEVIDEVKNESIFVRNIPLL
PRCWISLTMSEYSYLAYWTLVVIFIWTLSIATVCCNVFVYGFGLYVYTQFEILSTSVNIINGN
ENISEQRRLIKMFAKRHNHLLNIANDLENGSTFIILSEVTVIIFVGCIAGVMLLIGLRDHDSQ
TIIAMVIRLTLVMMQLFIYNFMGECLSNQTEKLQTAIYNCPWYELSSRTAKDMKLIMIRSN
NPFRLTAGKLSDMNLISFKSVVKSMFSFFSVLRLMLQN
>AgifOROR19
MDLQNKNKETSIKWNKDAIYALGLYRRLSTILGIWPSENRISSNIRIGTLIILQLTTVSIFIERII
VSGNCGTIQEIVDTIVISISSTQISLKCLLICCQHRKFKRIISSALEDWSSIIDQQSRKIMLKFG
HVGRVLFTCQVITGVATSSSLIINELPKLVELPDENNSSITLLRTIPLAPSCWVSTTMSSYLYY
TYWTSVALYIFYVGMATVSCNLFVYGLGLHVFVQFELLYLSLDTVFENHSHFQQKKIFKK
FVQRHDEILEIANDLEDASTGVILSEVAAIIVVGCVSGVMLLLAQHKGDSETVSAMAVRLV
LVFIQLYIYSYMGEKLSTQADKIQDAIYNCPWYTFSPLVARDLKFIMVRSNYPFYITAGKFL
PMNLMSFKAVVKSMFSFFSVLRLMLQN
>AgifOROR20
MSMSLIILLLIKGNCGAITEFVDALSLIICGLTSVIKVVIPRINYKKMSAIIQSAVNDWQKIDH
HDKLHFKTMQHFSNIGRVVFIIQMSGAYIVGFPLIVMKLPFVVNYFHLNKDESLLLINKTIL
TTSSNVGQVPIGPTCWIPTNMSPSIYFLYFISQSIQLFFVCSASIGGDTYFFGIGLHIWGQLRI
LNNSLDKFNKALLKEKILLNEFIKRHVHLLTLIKYFEDIYRYIILAQVGTDVLLTCISGIVLL
MTVQYGDPIVIGGLVIRIYLVYVQLFMYCFIGEKLTEESNKLEISIYHDIPWYNMDKKIVNN
IKFIMMRINYSYKLTAGKMYTMNYANFIGIIKAMASL
>AgifOROR21
MTWSDKEQKSFEKVSL
VISRVEWQLKVAGSWPFEKTLWSNIKFIIIVLFYVTHLILAYGDLIMVFSNIEKAVENFSSTGI
QTLIFFRIITLKLNEKLHNLFAATIEDISENNYKDYHEKRLFIKYHIFSYKYFMIGTWFASGS
AFCWYIKEIPGYIILRFNNETAILPPPFRIFIPFDVTSINRILIVYICETPVIVLSHLIILTSILYSLI
VSNICAQLSLLSHRMKINLSDKNEYNLTLKRELFFKRHVDKHCQLLSMVRETNNVYDIQL
FFELAVLTILLALLIHSESESINKADYTGVFFIGTFICVLLMIIFITCYLGECLHDEVNNLRNT
YWYCLSYKLTIQDRKNILICMAIVDKPLHVTAGGFYIYSLNSFLMIIKSSMAYVSILRQME
>AgifOROR22
MFAALVADIDIFLESVPTVLADVVCYLKYFNFFYNGSKMKELLITMEKDWKKYTSPEECQ
ILDKWARFGRKVTIYYAGALYGTLVPMGLVPLVPVVLDVVAPINGSYPRHLMFQQIEFLFD
YEKYFFPLLIHGYFGTMAYLTIIIAIDTIFMVYIQHACAKFEILGLFLDRVANDDDAIKNRHC
SKFDEIDYNDMVNCIATHNHAIDFSNLIEEANYVSFLFIIGINMIMMTASALVAAFKIDNPDI
AGKFVAFTLGEVFHLFYSSWQGQLLLEHSESIFNQVYQAKWNNASVRTQRLIIPLLMRSA
QPCKLTAGKMLTMSLTSFTTVVKTSFSYLTVFTSMRA

>AgifOROR23
MEHYYKVNEFLLSSIGQWPYQKKYSKLFFYGSTVFLLVTQGFLQTGGLIAARCEPAIFLEA
LPPVMISLMCIVKYINFIYNADKMKDLLDAIKSEWNSLTEEKEIEIFKNWAYDSRKSTLMY
AGAVYGSLVPLMISPIIPFFIDLIPDNSNDTIPRLTMFHVEYFIDVDKYYYPLLIHSYFGTMAY
ITVVVAIDTMFMVYVQYACSIFVILGHRLENLVRTDILMANANPNLIDDEPYRKIIKCIIKHS
DAIEYAHLIESANSVSFLIQLGINMICISFTGLQSALKVDQPHEAFKFASFTIGQTFHLFFESW
PPQRLADESERINEYTMRAPWYNTSLRCRKLLQLIIMRSRFPCQLTAGNFYILNLQNFSAV
VRSCMSMFTLLSSTQ
>AgifOROR24
MESSIPSYLKINKALLGMFGLWPHQNIFTKILLSFILIFFTSTQTIFQIWGALLAREEWELFLE
TIPNILAGFSIFAKMGNFFFNNHRMKKLLEQLEDDWRELKSSAEKEILNEYGVSARKFTLT
YLGIMWGTMTPFLVVPAVPLVINIFLPNNETLPRQMLFVQYEYLFDLKKYYYPVIIHSWIGT
CIFMNIVVALDTTCMVYVEHAAALFKCVGHNLNEIENTDEIKFDVNPKLSDDKAHKEIGQ
CMKLHNRVLKYSELIEDAHYISYFFQLGFMMICMSFTGFQIATKIHTAPDVSIRFAAMSSSQ
LAIMLFQGFIQGQQLFDSSAQVYHDIYNSKWYNTSMRTRKIQNLMSIRASKPCQLTAGRF
YILSYENYGIVVKTAMSYVMVLLSMEEED
>AgifOROR25
MKSSTIPLYLRINKFLLIISGIWPHQEFVSKILFTFFMLFFTGTQSLFQISGALFARENAAMFL
ETIPNILVDFSVGAKIANFFFNKNNMKKLMVLLQDDWILFDNKSEIEILKKYGAYARKFTL
TYLAVVYGTMMPFMIIPIAPIMINIFISDNSSLPKQLLFVQYEYLFDIKKYYYPVMLHSYLG
TCAFINVIVALDTTCMVYVQHCCALFRVLGYNLSKIDNETSHNFAFKPTKYYDKSHEEMI
KCMELHQRILDYAKLIEDSHYISYFFQLGINMICISFTGFQIATKFSIAPDVSIRFAAFTSTQL
AFLFLQSFPGQQLSDSSSQVFQDVYSAKWYKTSMRTRKLFLLMSMKSAKPCYLTALHFY
KLGYENYGMVVKTSISYVMVLLSME
>AgifOROR26
MKSSTIPLYLRINKFLLIISGIWPHQEFVSKILFTFFMLFFTGTQSLFQISGALFARENAAMFL
ETIPNILVDFSVGAKIANFFFNKNNMKKLMVLLQDDWILFDNKSEIEILKKYGAYARKFTL
TYLAVMYGTMLPFMVIPVVPIFINTFMKNSSLPKQLLFVQYEYLFDMKKYYYPVMLHSYF
GTCAFINVVVALDTTCMVYVQHCCALFRVLGYNLSKIENETNDNYAFNPKKYHDKSHEE
MIRCMELHKRILDYAKLIEDSHYISYFFQLGINMICISFTGFQIATNLNTAPDVSIRFAAFTST
QLAFLFLQSFPGQQLSDSSSQVFQDVYSAKWYKTSMRTRKLFFLMSMRSAKPCILTAGRF
YVLGLANFGTVVRTSISYVMVLLSME
>AgifOROR27
MLFLLAPHL
IYTYFVAEDLKRLIKIIAAQFFNSLALIKFWTMIINKKQLRWCLNELEDSWRDIASDEDRKI
MIKNAKIGRIFTVAYLSLSYGGALPYHIIMPLVAPRIIMPDNSTLIPLPYPSEYIFWVPRDSPG
YEMLFVGQILISSIILSTNCGIYSLIATYIMHACCLFEIVQRHLKYILESSVEGQFNDNLAAVI
DKHNQAIYYAGLLEKSLNIVFLSEMAGCTVIICFLEYGVLLEWGDGDVLGLTTYFMLLTSI
FVNVFIISYIGERLKEMSLKIGECTYDLDWYNLPKKMALDLMVIMVRANQPSTLSAGKLF
DLSLVGFADVVKTSAAYLNFIRAMV
>AgifOROR28
MGFKITPENALNFAKGRVWIISSWPPSTSGGNKWKMIWFNVRWWISFVFLLLLFFPLLNG
VYVFRNDSLVMIKAVTLSAANLQAIIKMAISRYQWKNLKSLHTDIDDFLKNSTPKEREFIQ
FYVNKGSFFHLFFTVTTWLVCLALYIEPIVFNQPFPTDAFYPFNVDNIILHNVLYLQQALSIF

IIASALIVDSQVAILIWFACARFAIIGERFEHIKNEDELKDCIQSHQNVLRYSDNVRKAVRFIA
VGTITTTTIGVICGLLTVISKQPVSVKIRVAAIIGCAATELLIYSWPADDLIQISQVIGDKIYRS
EWLEKSHKLRNMVQFVIHRTQKPVYISVPGIIPNLSLQYYTNFLSSTFSYFTTLRIVLAKEE
TSYEQ
>AgifOROR29
MKFKATPEAAITYIKFSVIILFTWPPGLGASKRTQFLFEVGWFISWLISILLVIPLGYAAYDQ
RKNTLNFTKSVCLAISCAQVVAKMLIAMCQRNRFQILINEMENFVKNSKNNDRKILVNYV
KRVSVPMIVFNFLSISACVGVICGPLVLDQPFPTEAKYPFSVDKHPVFDLIFLHQAIAGFQC
GSIGAIDCQVALLLWYMVARLDMLTFQLKNINKLSDLKNCVRTHQYLLWYIKELVQCARY
FVLTSIIMTTLSIIFGGIHIIGEQPLTVKLQFIIIDCGFSYLLYHSAWPAENLIRASERIGSILYEI
NWTKNSQEFNKIMMIIIQRSQKPTTITIAGFVPRLSLSYYATFLSKTFSFFTTLRIILTKMEAQ
DDHQ
>AgifOROR30
MSVFSYNIMIFRLLGMWYPEDNNSAWKKIFYIFYTTLIVSIYYTNAISQIIILFGSLDDAKEF
SGASFITITRILVCYKMYNILSKRKYIIHIINTLETGSFKARDSVELSIQTKFSRKIKLLIITYG
ALCATTLTALLYVSILVDIPQRKLAFEAWLPVNTSIPIFYWIVFIYQHFSGYCGTSISVSFDTV
LVGSMILLCAQLNILKYRLRHGHNNNEIENKSEKKYLIDCIEHHKAIYELAELSNEAFSNAI
FLQYFASLIVLCVSSFQLSQEKPFTKKFNDLILYLICQLLQVFMFCYWATQVQVESENIITGV
YDCEWLSLSITTKKNLILTMVRALKPIQYSSGYVVNLSLTEFTNLLRMSYSIYSILQQSNSR
TR
>AgifOROR31
MLKYTILLLTCVGLWQPTDWAKGWKTYLYTIYTFFVFLVVYSSTFSEFIYFAISNKGPIEVA
RQSFMLLTMICDCIKAATIVMNKCAIFDLIMLLEKDPYQPEINNKNKIMKEYDDKINKRSIA
YILMFAITGSSCMTAYSIKNIPKRAFITNIWVPYNHTTPIGYWFTYTCQITAHLYGASINAAS
DTLVMSIMIQICCQFSILQHRFEKLPDVMSNIKKYDDEKSIILETMKLSECVEHHLQIYQWA
ERCNQIFSGIIFLQYSISLNVLCVSVFTLSKIEYNNLNFVGLVIYLCCMLTQIFMLCYTGSQV
TTELIKLSYSAFNFLNRSSE
>AgifOROR32
MLKYTILLLTCVGLWQPTDWAKGWKTYLYTIYTFFVFLVVYSSTFSEFIYFAISNKGPIEVA
RQSFMLLTMICDCIKAATIVMNKCAIFDLIMLLEKDPYQPEINNKNKIMKEYDDKINKRSIA
YILMFAITGSSCMTAYSIKNIPKRAFITNIWVPYNHTTPIGYWFTYTCQITAHLYGASINAAS
DTLVMSIMIQICCQFSILQHRFKILPDVMSNIKKYDDEKSIILETMKLSECVEHHLQIYQWS
KRCNNIFSGIIFLQYSISLNVLCVSVFTLSKIEYNNLNFVGLVIYLCCMLTQIFMLCYTGSQV
TTESIQVGDAIYDMDWGQFESSTKKHLMMIMKRTMHPVVFTSGHFVTLSIDSFTVLIKLS
YSAFNFLNRSSE
>AgifOROR33
MDFLPGSFIIFRIVGLWRTSHNNIYIYRLRTVLTLFLLYTFVGCSIIGIAMKHDDMERVASDC
FLMISVLGCCGKSVNVLICRNKILNMIDILQNDPCLPRDDIETDIQKKFDTFIWKSTIIYGILT
EITAGMLTFGTLLLDLPEGELPFNTWLPYDHSHGFIYKFAYGQQIISVMTSANIAIAFGTLVP
ALILQVCAKLNILKHRFTNITNYIYKQQLNNNDLSFEEKLNREKELIADFVRCHLTIIKLAE
TINSTFNLMIFLQCLISATVLCTSVYKLVTVPLFSSEFSSIVLYLACMLSEIYMLCAAGNEVIL
VSGSISDAIYCADWTKLNISTIKSLVLIMNRAMHPVTFTSGYIVILSYDTFKSLLKISYSTYN
VLQQKG
>AgifOROR34

MSILSYNVMIFRLLGLWYPENSITTRKLFFYKLYTGLLVVTLYTFTLSQLIKMFTSLDNPDD
FSKASFMSITMTATCFKVYDMLSKKKLLARLINTLDNGSFKSRNSIESSIQSNFFNKIKLLIF
LYGTLNESTATTITLASIIRDAPQRALAYKAWLPFDESKPTFYWIAFIHQHFGGYCAASMSV
AFDTMVVGLMISVCAQLRVLKYRIHHGYNTVTYEQKKNYENDYLVDCIQHHNAIFQFAE
LSNYIFSKAIFFQYFASSIVLCVSTFSLSQEKTFSKEFNNLIMYLACMLSQIYVLCNWSTNV
HIESTSIATGVYESDWPSLLIGSRKNLVIMMIRTLRPIQYSSGYIVNLSLTAFANLLKTSYSAY
NILQKSKSRTE
>AgifOROR35
MIILVYTNTLSQIIDLFVSFKNLKQFINNAFILLSTIGAGVKAAHLIWQRKTIINLMKIIQSHP
CIPQDEDEQIVQQKFNNKIKRMNFAYICLFVCTITTLTIASFFRDIPMKQLYYHAWIPFDYSS
KFRFWMVYVHQVIAHGFDASLHAAYDTLAPAMMIQTCAQFELLNLRFEKLPQSLKKKIH
KFEGNNNVMINSQHLKREEAKKIGECVSHHIQIIEFSKKNNSIFGTSIFLQYSVSSLVLCMS
VLRLSQLNTFSPDLASVFLYLISMISQVFIPCFAGNQLTVQSSQICDAIYSMDWTTLTISTQK
SLVLIMSRSQKPVQFISGRIIPLSMNSFNNVIRISYSIFNVLHGSSAI
>AgifOROR36
MSVFSYNIMIFRLLGMWYPEDNNSAWKKIFYIFYTTLIVSIYYTNAISQIIILFGSLDDAKEF
SGASFITITRILVCYKMYNILSKRKYIIHIINTLETGSFKARDSVELSIQTKFSRKIKLLIITYG
ALCATTLTALLYVSILVDIPQRKLAFEAWLPVNTSIPIFYWIVFIYQHFSGYCGGSMIVAFDT
MLVGLMISICAQLKILKYRICHGHNTFTEKNYLIDCIEHHKAIYEFAEMSNKIFSFSIFFQYF
ASSIILCASTFKLSQEEPFTEKFNNLLLYLIIMLLQIFMLCYWATQVQVESENIITGVYKCDW
PSFSIETKKSLLLTMVRARIPIQYSSGYVVNLSLAEFTNLLKMSYSIYNILQQSNTNSR
>AgifOROR37
MSMLSYNIMIFRLLGLWYPEENNFTWKRLFYKFHTTFLVSMFYTLTISEVISLCGSLDPSEF
LDASFLSITRVLICWKIYNILSKSKYIIHMINTLETGSFKARNLVESSIQIQFSQKIELIIILYGG
LIEITTTTIIYGSIVTDIPQHKLAYKAWLPFDISKPTFYWIAFIHQHFSGYCGGSMIVAFDTML
VGLMISICAQLKILKYRICHGHNTFTEKNYLIDCIEHHKAIYEFAEMSNKIFSFSIFFQYFASS
IILCASTFKLSQEEPFTEKFNNLLLYLIIMLLQIFMLCYWATQVQVESENIITGVYKCDWPSF
SIETKKSLLLTMVRARIPIQYSSGYVVNLSLAEFTNLLKMSYSIYNILQQSNTNSR
>AgifOROR38
MSSGSFVVFQIVGLWRSPNIKSSIYNNFYRLRTFFSVFLLYSFVGCSIIGIAIKHDDVKTVTN
DCFVMLSVLACCGKSINILKCRKTILNIIEIMQNDPCSPRNEHEIDIQKKCDSFIWINTVIYGI
LTEVTAVMLTFGTLFIDLPEGELPFNTWLPYNHSHGFTYKFAHGQQIISIMTSANIAIAYDTL
VPAMILQVCAKLNILKHRFSNFTKMINNQINKSSTSIDKLKKEKQLIADYVKCHLIIFKLAK
TINNTFSIVVFLQCLISTLVLCVSIYNLASVELFSSEFTNIILYLACMLSEIFILCAAGNEVTLV
SQSISDSIYETDWTDLNTSTIKSLVLIMSRTMKPIIFKSGYVIELSLDSFKSLVKISYSTYNVL
QQTS
>AgifOROR39
MSRDCFLPFAVLGCFFKSLNIVGSHDTITYIIQTFMNDPCLPRNSKEILIQKKFDKFIWRYTV
AYGVLTETTAIGLTIGTLWLDLGPGVLPFPAWLPYKHEDGFWYWFAYYYQLLAAIISANFA
IGYDTFITSLMIQIVSKFKILQHRFENFYEINKTDNKYDNIFKNEKAFLFDCVKFHEILFKLS
NIINETFDFVIFMQCSLSVCVICVTVLNIDKYQTFSQEWTTVILYLCVILSEIFVLCAAGNEV
SFVSSQLGEAVYSMNWTDLDKSTIQSLILIMNRSSHPIVFTSKNIVTLSLDSFKTLIKISYSTY
NVLHRT
>AgifOROR40

MDDVMNFLPACFFVFYIIGCWKSKNYTTSISICFYWLRTIIVIFLLFTFTVSSLLGVAMSKKN
IQEMSNDCFLPFAVLGSFCKSLNIVGSHDTITYIIQTFMNDPCLPRNSEEILIQKTFDKFIWQC
TVAYGIMVEVTSIGLTFGTLLLDLGPGVLPFPAWLPYKHVDGFSYWFAYSHQLLAGINAAT
FAIGYDTLIPSLMIQIVSKFKILQHRFENFYAINKTDNKYDNILKNEKSFISDCVKFHEILFKL
SEIINENFDFVIFMQCSLSVCLICVGVLNIDKYQTFSQEWTAAIFYLGCMLSQIFILCAAGNE
VTLVSSQLGEAVYSMNWTYLDTSTIKSLILIMNRSLHPIVFTSKNIVTLSLDSFTNLIKISYST
YNVLHRT
>AgifOROR41
MLKYTILLLTYCGLWQPSDWIPGSWKTYLYTIYTYLMILLLYTITFTEFLYLVTSTAEIEEIA
NNSFLLLSMIGVCVKAATVIINKCVIDDMNILLGKDPFRPQTIAEKRIQNKYNDIINKSTFIY
MSMIEVTSFVMVFSISIKNIPNRVLSCNAWVPYDYTTSPISFWLTYVLQLTVHAYGASINAA
FDTLIPSLMYQICCQFSILQHRFKILPDVIRNMKEFDEKRTIKLNECVEHHLKIYEWSKRCN
NIFGGVIFLQYSISLNVLCVSVFTLSKIEYNNLNFVGLVIYLCCMLTQIFMLCYTGSQVTTES
IQVGDAIYDMDWGQFESSTKKHLMMIMKRTMHPVVFTSGHFVTLSIDSFTVLIKLSYSAF
NFLNRSSE
>AgifOROR42
MDDVMNFLPACFFVFYIIGCWKSKNYTTSISICFYWLRTIIVIFLLFTFTVSSLLGVAMSKKN
IQEMSNDCFLPFAVLGSFCKSLNIVGSHDTITYIIQTFMNDPCLPRNSKEILIQKKFDKFIWR
YTVAYGVLTETTVIGLTFGSFWLDLGPGELPFPAWLPFKHEDGFWYWFAYFYQMLAGINS
ATFAIGYDTLIPSLMIQIVSKFKILQHRFENFYAINKTDNKYDNILKNEKSFISDCVKFHEILF
KLSEIINETFDFVIFIQCLLSIFLICLTVLNIDKYQTFSQEWTAAILYFGCMLSQIFILCAAGNE
VTLVSSQLGVAVYSMNWTNLDTSTIKSLILIMNRSLHPIVFTSKNIVTLSLDSFKTLIKISYST
YNVLHRT
>AgifOROR43
MILLLYTITFTEFLYLVTSTAEIEEIANNSFLLLSMIGVCVKAATVIINKCVIDDMNILLGKDP
FRPQTIAEKRIQNKYNDIINKSTFIYMSMIEVTSFVMVFSISIKNIPNRVLSCNAWVPYDYTT
SPISFWLTYVLQLTVHAYGASINAAFDTLIPSLMYQICCQFSILQHRFEKLPDVMSNIKKYD
DEKSIILETMKLSECVEHHLQIYQWAERCNQIFSGIIFLQYSISSVVLCVSVYLLTKIEYGNP
EFLPVVMYTMCMVIEIFILCYSGNQVTIESINVGHAIYDMDWTQFELSTKKNLMMIMNRT
LHPVIFTGCHFVKLSIDSFTSLIKLSYSAFNLLQSG
>AgifOROR44
MLKYTILLLTYCGLWQPSDWIPGSWKTYLYTIYTYSVFLTVVYSIALLELLYLVTSTDEIEEIA
SNSFVSFSMISASAKALTIFMNKSVIFDMIILLEKDSYQSKIDCKNIILKKYNEMINKSSLSYI
IMVEVTMIVLVVTFSIINIPNRVFTTNILVPFGEITPFRFWFIYIFQNIAHAYATSIHGAFDTLLP
SMIYQICCQFSILQNRLKIIPDIIHNIKKFDQTKKIILEKNKLSECVEHHLQIYEWAKKCNNIF
GAIIFLQYSISSVVLCVSVYLLTKIEYGNPEFLPVVMYTMCMVIEIFILCYSGNQVTIESINV
GHAIYDMDWTQFELSTKKNLMMIMNRTLHPVIFTGCHFVKLSIDSFTSLIKLSYSAFNLLQ
SG
>AgifOROR45
MLIFETGYYRLNKIGLMICGAWPRQSKIVAYTIRVIIMLLFISSFIPQAVYSYNFWGNFNEIVP
VSTTQAALLLIALKFLFLVGQKNMVHRVINQIQDDWKIFSKIPEIKKIMNHHATDAYNYTL
FYMYFLIVLGSSYVGFVFIAPVMDIISPLNYTREMTPLYKTNYGVDSEKYFFSIYAHGFIITT
IDVICIWTSDTFIIMMVQHCCTLFSIVGILLQQLNAKNCNYNENHERKIITQAIIVHKYAIEF
AEFIEKSVTKLYGFVFLLTMIFMSITGFYVVLIMEENGNEGMRMVAFTIGQAIHLFFSTLPS

QSLINSSENVFYSVYNCQWNELSIKSQNSLCIMLIRSSKSTGLTASKFYPINLENFGNIIKTSISYFTVLKSM

>AgifOROR46

MSILDSPYFSLNKRVLKFYGQWPYQSKLQNRCHRIVVFLAVSSILLPKLIKMIESRHDIDEFILCMPMVFLHIEGLLNLTAWCLNGDKIENLINRIQNDWEILKTDKERQILVEYWKRGRSSISYYAIPMATVLIIFLISPGLPKVLDIIAPLNESRPHELPYETEYFVDQDDYYLYILIHAYMTVPVSLGLTVLFNSVISSWIHHAAGMLAIVAYQLETIHDIPESDERFSQFEKNQSIQKRLAYSVKLHNNAIEFIETVESSASTVLFIVIIGSTLILTVTGTVAVIKLNHPNESSRYMSFSLATLGHLYYISYVGNQIIIASESINRACYASEWYTLPIKYQKILIPIMARTNRPCTITAGKLYVLSMDNFSELIKKAMSLFTFINSTTR

>AgifOROR47

MTVFETGYYKINRVSLCLCGVWPEQNSFQAYIARFLLWGGMATIMIPEYVCLYKAWVYGSFMEDFLPCIPTQLLITAVAAKYVVFAIYKKTFQDTIDKMRVDFEFFGNTPARKILKEYADKGRTRTIFYGCFLFMILTTFVVPAFLPVVLDKFRPLNETRIRLKLLDTDYGVDPDEYFVLITIHGYIASTFLVLMLWSVDSFIMIMVQHCCSLFVIVGFTLKKLDQGNKKHTEKFENDVLINALQVHQQAIEFADFIEASLKNMYGIVIIENMLAISITGLETIKQMDQTDQAMRFAAFAFGQMVHLFFNSLPGQELVDHSTGLFDLVYDCQWESLSIKSKKKILFILMRSAKPKSLTAGGFYELNLQHCGAVLKTSMSYLTVLSSMRQ

>AgifOROR48

MEFFDNPNWIMTKYLLSVLGGWPLQQATIGRTILRTVIVLLVISSLLPQLFIVFTRLNDIDTVIESSAIAFVHFISITNAIYCFLNTETEKKLLIQIKNLWESRLSAIERGYLEVYGNAGKKITHIYAISIYTTGMITLSTTIAPKLLDIILPLNETRPTVFLYPAEYFIDEEKYAMFILIHSWILTPIQSTIIVGFDTLYAHYVQHLCSWYLIVGHRMKNISTALDKINKNNNLFDENKRIILSFEKCINEHIKALEFTSNIQSIYGTPLFFIIGFNMIQISMTGCHTISKIDDPPEAFRYGAFTIAQFVHLFFLSWPGQKIIDHSGELFEAIYETRWYDLPGKTKQLMILIMIKCQTPCVIKAGKIIEVSLESFAIILKTSISYFTVFTSMQ

>AgifOROR49

MSILESPDFLLSKRILKLYGQWPYSSGVINYIKRIYCFVAVISILIPKLIKIYESRHDVDAFILCMPMVAVHIEAIINLTSWCTSGEEIKYLIETIKRDWDILKSGDEIAILDGHWKRGRSNINNYAYPMVGITLVFMTSPAIPLVCDIIAPLNESRPRVFLYETEYFVDQDEYYFHILIHEYMTVPLSIALTVYYNTVLGSWIYHATGMLAIVSYQLENMHVLSEGDKAFKEHEKNQNIYERLTYCIKLHSSTIEFIEIIESSNSLALFLVILICTLIFVITGVVSIVRMDHRYVGFSIGVLGTLFFLSFIGNEVIKASEAVHIASYHNEWYNFPISYQVMLMKIMMRSEKPCTISTGKILDLSMENFSELLKKAMSLFTFINSTR

>AgifOROR50

MSILESPYFSLNKRVLKFYGQWPYQSKLQNRCHRIVVFLAVSSILLPKLIKMIESRNDIDEFILCMPMVVLHIEAMLNLSSWCFNDDNMYKLMDKIQNDWESLKTDKEREILDEHWKRGRSTINNYAIPMATVLIIFLISPGLPKVLDIIAPLNESRPHELPYETEYFVDQDDYYLYILIHAYMTVPVSLGLTVLFNSVISSWIHHAAGMLAIVAYQLETIHDIPESDEQFSQFEKNQSIQKRLAHYIKLHNDAIEFIETVESSSSTVLFIVVIGSTLVVTVTGTAAVIKMNHINESSRYMSFSLGTLGHLYYMSYVGNQVIKASESINTACYASEWYTLPIKYQKILIPIMVRTNRPCTITAGKLYVLSMENFSELLKKAMSLFTFINSTR

>AgifOROR51

MFVIMEVWESVYYRTNKRALQIYGQWPFYSKFRTRCHRIIIYTILLSILTPKMIKFIESFGDI

DETIECLPMIATHFVSQTKFVAWTFTSSEMKKLIIMMEKQWKEITSIEDRKLLDYYAKRGQ
VTTRLYAIALFGVMGVYLATAGLPKLLDKINPLNETRPKIYLYKTEYFVDQDDYYKEILIH
AYLTVPLSVGVIIYFDNMLACYISFANGMFAIVSKHLKNIHVLSETDQMKSTIDKHKSIFNR
LKYCIKIHSSTIEFIELAESSTTLALLFVAGLNTVVITVTGIVSVMKIDKPSEASRYMAFTLG
GIFHLYYISFLGNQLIQGSASVHRACYDNEWYTLPINYQKLLIPIMMRSKKPCQMTAGKLY
IMSMDSFSDVLKKAMSLFTVLNSAR
>AgifOROR52
MTIFEAGYYKINRMGLCLCGIWPEQNLFLAYIARFFLWGGIISSMVPEYVYLNKVWTNGS
FLEDVVPCIPTQLVIILVATKFIVFARDKETFQNAIDKMRYDCEFYMDLPAGKILREYGEKG
RTRTIFYGLFLLTILTTFLVPAFLPVVLDKLMPLNETRIRFSLFDTDYGVDPDEYFVLITIHGY
IVSVFLIFMLWSVDSFIMIMVQHCCSLFMIVGFTLKKLDRTNKKTFTKKFENNVILSAIQIH
RQAIEFADFIEASLKNMYGIVIIENMLAISITGLETIKQMDQTDQAMRFAAFAFGQMVHLFF
NSLPGQELVDHSTGLFDLVYDCQWESLSIKSKKKILFILMRSAKPKSLTAGGFYELNLQHC
GAVLKTSMSYLTVLSSMRQ
>AgifOROR53
MDEKKKLQAYVAFSEMTKKLMLVAGLWPVANPTLFYSIKPLIALSIALCSAIVITNFTIVNI
KNIAIMTKGLSLATSFYTTSLKIICFALNRKDAMVLHNTLANYYIVYLNNENLKKLVLTGF
SGIRRLSTTFSTLVFLSILTYAVKPMISILIQLLHNDGNKIVLMLAMPAKYPWNSIDNGQLFY
WINYLFECSAAVCLFVVTCSADSLFSFYVFQMSGHLRVMSCQLNFHEENINHHIIIRECVIK
YGDLIKCRDILQKIYGPIILWMIISSALVICALIFQMAELEKVTVGEVLLFTAYSGSKLLQTF
MYAWSGSVLTSESENFLDAVYQSNWVGKNRKRFQTSILILLTQKPIVLIACKYFAVSIDMFV
MVLNTSLSYFFLLQTMAEKQN
>AgifOROR54
MDTVATSYHKINRKFLKSLGIWPYQSSLSKKFFILLTLFLTITHGYLQTAGMFAALVADIDIF
LESVPTVLADVVCYLKYFNFFYNGSKMKELLITMEKDWKKYTSPEECQILDKWARFGRK
VTIYYAGALYGTLVPMGLVPLVPVVLDVVAPINGSYPRHLMFQQIEFLFDYEKYFFPLLIHG
YFGTMAYLTIIIAIDTIFMVYIQHACAKFEILGFSNLIEEANYVSFLFIIGINMIMMTASALVA
AFKIDNPDIAGKFVAFTLGEVFHLFYSSWQGQLLLEHSESIFNQVYQAKWNNASVRTQRLI
IPLLMRSAQPCKLTAGKMLTMSLTSFTTVVKTSFSYLTVFTSMRA
>AgifOROR55
MKKATSMETPQ
YLRFNKNLLACAGLWPHQQKLSKIIFFIILLFLIISQTAFQIIGAILARENVDLFMEAIPAILGG
IAIAVKIMNFYLNGHKMKKLLVLIQNDWIEYKNGPESLILKKYAKFSNKLTLMYFGIIWST
MTPFLTIPLVPLIFNIFVSQNNSIQRQHLYAQYEYVFDSDKNYYPVLIHSYIGSFAFINAVVAI
DAMITMYIEHACALFSIIGYVKLLDNANSISYFFQLGIDIICLSFTGFQIATKIDNSPDAAMRF
AAYSTSLLVVLYFLSYPGQQLIDHSLKICHNIYKANWYCTSLRTRKLLIFMAMRSSIPCQIT
AGKLYVMDITSFKLVVKTSISYVMVLLSVQE
>AgifOROR56
MSRNNIANLKSSYR
WTKLNFDIVGSWPSSSSNIGQFRAFINSVLITTLILIPRFSALYYIKNYLDGVVFNASGNLIYI
VSIIKMFVIFKNQKVLAKILDDILSEWNNSLENSNYHVVEKYANISKNISILSLLISWTVCV
MGCLAQRYSNIEIDDSVERHPRLAEDMFWSKLKKIVKRHHELIQLSVTLEKCFNKVLLLQ
LLTCCLTFASQGYLMIGKLIRGDMTFFQTEFAVAYTTYTVVHFYFLCYAGESLIQTSMKIGF
AAYEAEWYDLSTNEGKLLMFVTLNSMRSLKITTGKFAILSYELFITVIRTSLSYLSVLLAAK

DSKS
>AgifOROR57
MNSVSFLLAITLSAPMSRNNIANLKSSYRWTKLNFDIVGSWPSSSSNIGQFRAFINSVLITTL
ILIPRFSALYYIKNYLDGVVFNASGNLIYIVSIIKMFVIFKNQKVLAKILDDILSEWNNSLEN
SNYHVVEKYANISKNISILSLLISWTVCVMGCLAQRYSNIEIDDSVERHPRLAEDMFWVSY
FPYDIHATKIIFALHWTLQAYASVVGATIYVAFDCYCCFLILHLTGQLTILQIDLRNIHLKYK
SKNYNTNNLQSKLKKIVKRHHELIQLSVTLEKCFNKVLLLQLLTCCLTFASQGYLMIGKLI
RGDMTFFQTEFAVAYTTYTVVHFYFLCYAGESLIQTSMKIGFAAYEAEWYDLSTNEGKLL
MFVTLNSMRSLKITTGKFAILSYELFITVIRTSLSYLSVLLAAKDSKS
>AgifOROR58
MNILYTISVWQDDVDSAITMLSIVFTVMVTITKGIIIWFYQEELYNILDNLHEKWNKSWTRI
EIRDQILETVVRVTKFRNIYVVALIGLFITYTYPPIGNVIIYLYKNTNESMDYSQTMWEAEV
YPIEIDTFLKYFYLSTMDIYQSVVYIICCYVGDIFYVQIFTHISIQFRVLANDITTTVNTNKNG
DSKKLSMELKHIVMRHQELYKLFDIMNDGLYSSVIFTSFVIGSINMCLNIFEFQKAVDEGD
YYAASRFLFLTVTIFSGIILYCVQAEIICASAEFIPEAIYNSQWSNIDKNSILTLQIIMTRCQRK
LTCGAYGLVDIHHEQITQVC
>AgifOROR59
MAQKKQLKNIAKCRKRLKLVTMALGIWPQKNPNTNFYCIQSYIVVVYLVIVYSAVLNFM
YQNRHSFTIVLKGASLSLSFSIVTLKVVVFIYRRKKLREVEDIMEEILKSQLRKSDELVTTM
LGPLDYFAKHISFGIFFCGYAIGTVLYSFPIISLVKQHINNVHPKKYTTPYPTIYPYEIVGGST
LWWAHFAFESILSLIFCSIGTSTDNLFGYYCSHIMSQFRALNYEMENIKLDDQLKVNITDIVI
KHHKLINCCDLLMETYGEIIIGLLITTALILCCLVFQISQMTQITIGQVIWFTVYICYKLSQAL
IYAWAGEKVIEESEKFRMAIYTCSWETQYRRDLGKYLIIMMSQRPVSFTACGIVSVDAKLF
TSILNAALSYFFLLQSLNE
>AgifOROR60
MKRQAELQSFKSYSSGIRILLLFMGLWPVENSNVFYKLIPYFLGSVLLIVTSASMNFAIHYH
YNLMIALKGVSISLSYLATTVKVICYVIHRKKLMKLEKTLHELLSEQENNKNNDELIKTAL
TPVFNFRRLSVSLSFCSFSVVTTYFLTPVISMIKQYKNNIRPIKYLLPYPTLYPYAIEGGSLLW
IIHFIIETYACFTLFTITASVDTSFAFYSSRVIGQFRVLSNNIRSLKFKGNYEKRIQGYIVHHRK
LITCCELLQDIHGPIVLAILLTTALIMCSLTFQISQMETISIKQTILFVVYIAVKLAQTWVYAW
SGEMIAAESEDFRKAVYECGWETSDNKTVKHCILFMLMQKPLILQACNYTQISAQLFVAIL
NTTVSYFFLLQTINDD
>AgifOROR61
MKEQEDKLKEVKKKVRIFRLVMSGVGIWPYENPTIYYRLLACLVAFLLLQTFCGCINFIVA
NSDNLMIMLNGMGLSLSISCVMLKGGSLIYFRKDNIRMNKKLEELIDKQMKGPEDVCNV
MLEPMVVFARKIFYLIYCLGFGMVLVQWLRPTVAMTKQYMHGYNITYRRPFPSVYPWKI
EPGSDLWKFHFFVDSITTWFYFSIGISTDNNFTHQAAQIIGQFSALNYEISNLKVEDVNNDK
IKDFVERHQDLMDLCQTLGDCNGLIVLVLTLSAAIILCTLSFQCSKMETITVGELTWLLTYI
FYKLLQIFLFAWSGEIIKKKSEELRDNVYGIAWTDKADPKVNHAVRFMMHQRPVVLQAL
GIKPISAELFSGVVNTSMSYFFLLRTISE
>AgifOROR62
MAQKKQLKNIAKCRKRLKLVTMALGIWPQKNPNTNFYCIQSYIVVVYLVIVYSAVLNFM
YQNRHSFTIVLKGASLSLSFSIVTLKVVVFIYRRKKLREVEDIMEEILKSQLRKSDELVTTM
LGPLDYFAKHISFGIFFCGYAIGTVLYSFPIISLVKQHINNVHPKKYTTPYPTIYPYEIVGGST

LWWAHFAFESILSFVFCSIGTCTDNLFGYYCSHIMSQFRALNYEMENIKLDDQLKNNIKDI
VIRHHKLINCCDLLMETYGEIIIGLLITTALILCCLVFQISQMTQITIGQVIWFTVYICYKLSQ
ALIYAWAGEKVIEESEKFRMAIYTCSWETQYRRDLGKYLIIMMSQRPVSFTACGIVSVDAK
LFTSVSEL

>AmelOR1
MENTTNYRNIHYKSDAEYTVHVAKTLLTLIGIWPRRNTFIDNVKFYVQIGIVFFLMCFLLL
PHVIYTYFDCENLTKYMKVIAAQIFSLLAIIKFWTIIINREEIRFWLMEMEIQYRDVECEEDR
LVMMNTAKIGRFFTIVYLSLSYTGALPYHIILPLISERIVKEDNTTQIPLPYLSDYVFFVIEDS
PIYEMTFVLQIFISSIILSTNCGTYSLIASITMHCCGLFEVTNRKIKTLCKWNNRDLHDRVIDI
VQSHLKAIEYSARVGESLSIVFLSEMLGCTIIICFLEFGVIMELEDHKTLSTVTYFVLMTSIF
VNVFIISFIGDRLKQESERIRETSYFIPWYDFPTEVAKNIKTIILRASRPSSLSGAKILELSLQA
FCDVCKTSAAYFNFLRAMTV
>AmelOR2
MMKFKQQGLIADLMPNINLMKATGHFMFNYYTDSSTKHIHKIYCIVHLVLILMQFGFCGI
NLMMESEDVDDLTANTITMLFFTHSVVKLVYFAVRSKLFYRTLGIWNNPNSHPLFAESNAR
YHQIAVKKMRILLLAVIGTTVLSAISWTTITFIGDSVKKVIDPVTNETTYVEIPRLMVRSWY
PYDPSHGMAHILTLIFQFYWLIFCMADANLLDVLFCSWLLFACEQIQHLKNIMKPLMEFSA
TLDTVVPNSGELFKAGSAEQPKEQEPLPPVTPPQGENMLDMDLRGIYSNRTDFTTTFRPTA
GMTFNGGVGPNGLTKKQEMLVRSAIKYWVERHKHIVRLVTAIGDAYGVALLLHMLTTTIT
LTLLAYQATKIHAVDTYAASVVGYLLYSLGQVFMLCIFGNRLIEESSSVMEAAYSCHWYD
GSEEAKTFVQIVCQQCQKAMSISGAKFFTVSLDLFASVLGAMVTYFMVLVQLK
>AmelOR3
MSVKTARNIRDYHNIHYRSDAEYTVRVAKILLTMVGIWPRRNTFSNNVKFYVQTTIVFFL
MCFLLLPHVIYTYFDCENLTKYMKVIAAQVFSLLAIIKIWTILINRNEIRFCLMEMEVQYRD
VECEEDRLVMMNTAKIGRIFTIVYLFLGYGGALPYHVILPLISERIVKADNSTQIPLPYLSDY
VFFVIEDSPTYEITFVVQMFTSFLIMSLNYGIYSLIASITMHCCGLFEVTNRRIETILKNRDLR
GRIADIIQSHLKAIEYSALVGKSLSIVFLSEMLGCTIIICFLEFGVIVEWEDHKTFSMVTYFVL
VTSMFVNVFILSFIGDRLKQESERIGQTSYFLPWYEFPTEIAKNIRIIILRASRPSSLSGAKML
DLSLRVFCDVFKTSAAYLNFLRTMTV
>AmelOR4
METKHTEKDLKQAFYVQTFLKIIGAWPIAIESSLGSKIQKWFIISFYLFLQICIVAPCILDVFL
KEKNGSRRINLFMLLISTLNQVFKYVITLNRANELRIAIHEIKKDWLTATPEDRFIFVMNSRI
GQRIMLIMAFIMYISGLGYRMVLPLLKGKIVLPNNVTIRLLPCPTYFTFFNELVSPYYEMIF
MLQLLARFFIYTVLNSTVGISLMLSLHMCSLLKILTRKMADLTDGSIISEKIMQQRIVDIIEY
QTRIKRFLSNTELITQYFCFYDIGCSTCLICFIGYSIIVEWENHNIASTVIYFSGLVTCTLMIYI
ICYIGQLLLDESNNLAQTCITLNWYRFPKKKARYLILMIIMSNYPIKLTAAKVVDVSLTTFT
DVMKAAVGYLNMLREVI
>AmelOR5
METKHTEKDLKQAFYAQSFLKIVGVWPIPIGSPLSSKIRNWFITFFSLFLQICIVGPCILVMFL
KEKNGKRKINLFKLLTNTLNQLFKYIITLNRANELAIAMNEIKNDWLTATSEDRWIFTANSK
MGQKVMLIVAVTVYSSGLGYRMLLPILKGKIVLPNNVTIRLLPCPTYFTFFNELVSPYYEMI
FMLQLLAGFFSYTVLNGTVGISLMLSLHMCSLLKILTRKMANLTDRSITSENIIQEKIVEIVE
YQTKIKRFLGNAELITEYFCFYDIGCNMCLMCFIGYSAILEWENHNIAAIVVHFMLLGTCIF

IIYIVCYIGQLLLDESNNLAQQCITLSWYHFPTRKARCLILMIIMSNYPVKLTAAKVVDVSL
TTFTDVMKAAMGYLNMLREVI
>AmelOR6
METKEKDLKQAFYAQPFLKIIGAWPILIESSLSSKIQKWFIISFSISLQMCIVVPCILVMFLKE
KNGRRKINLFMLLTNILNQVFKYVITLNRANELRIAIHEIKKDWLTATPEDRFIFVTNSRIGQ
RIMLIIAVITYSSGLGYRMVLPLLKGKIVLANNVTIRLLPCPTYFTFFNELVSPYYEMIFMLQ
ILAGVFVYTVLSGTIGISLMLSLHMCSLLKILRRKMIDLADGSITSENTMQKRIVDIVEYQT
KIKRFLGNTELITQYFCFYEISCNTCLICFIGYCIILEWENSNVVAIVVHFMLLGTCILVTYIV
CYIGQLLIDESNNLARTCITLNWYHFPTRKARCLILIIIMSNYPVKLTAAKVVDVSLTTFTD
VMKAAMGYLNMLREVI
>AmelOR7
METKEKDLKQAFYAQPFLKIIGAWPIVIESSLSSKIRKWFIISFSISLQMCIVVPCILVMFLKE
KNGRRKINLFMLLTNILNQVFKYVITLNRANELRIAIHEIKKDWLTATPEDRFIFVTNSRIGQ
RIMLIIAVIMYSSGLGYRMVLPLLKGKIVLPNNVTIRLLPCPTYFTFFNELVSPYYEIIFMLQI
LAGFFIYTVLSGTIGISLMLSLHMCSLLKILRRKMIDLADGSITSENTMQKRIVDIVEYQTKI
KRFLGNTELITQYFCFYEISCNTCLICFIGYCIILEWENRNVIAIVVHFMLLGTCIFVTYIVCY
IGQLLLDESNNLARTCITLNWYHFPTRKARCLILIIIMSNYPVKLTAAKVVDVSLTTFTDVM
KAAMGYLNMLREVT
>AmelOR8
MVQIRNAREGINHTFWFAYPLSRMLGYWPLNVPSSAFSKILNSFTIFFSYLLPLIVLIPGLLY
VFLKERNGRRKVKMLMPHINSIAQMTKYTIILRRTKELGKLLDEIKKDWSTATQENRRIFS
ERASIEHKLTMIVAITIYGGGFLYRAILPLSKGRIVLPNNVTIRLLPCPGYFGSLDEQVTPNY
EIIFTLQVLGGFVTHTAVCGIKSACLMVCMHMCGLLRILTNKLTDLTNDNDERVVQEKIVH
IVEYQTRIKEFLNHVDQFVPYVYLIEIFVGVLITCILGYCIIVEWEDSDAMAIIAYVALQTTC
VFGTFSICYVGQLLVDESESVRQACKTLKWYRLPTKKARSLILLIIMSNYPIKVTAGRLVD
VSLVTFTSIIKSAVGYMNILQQVT
>AmelOR9
MARIRNAREGINHTLWFAYPLSKMVGCWPLNIPSSTFSKIFNAFIIFISYLLSLIVLVPGLLYL
FLKEKNGRRKIKMLMPLMSTIAQMTKYTILLRRMKEFNKLLDEIKKDWSTATQENRQIFS
AKASIEHKLTTVIAITIYGGGIFYRMILPLSKGRIVLPNNVTIRLLPCPGYFGSLNVQITPNYE
IIFTLQILGGFVIYTALCGVKSSCLMLCMHMCGLLRILTNKVMELTSDKDEKVVQEKIVYI
VQYQTRIKEFYNYVDQFVPYVYFIEMIVGVLITCVLGYCIIVEWEDSDAMAIIAYVVLQVT
CVFGTFSICYAGQLLVDESENVRQACNTLKWYRLPTKKARSLILLIIMSNYPLKVTAGRIV
DVSLVTFTSIIKSAVGYMNILQQIT
>AmelOR10
MVQIRNAKEGLRHTFWFAYPFSRMLGHWPLSVSSSAFSKILNSFIIFISYLLQMIVVIPSLLY
VILKEKNPKKKIKLLMPHLNSIVQMIKYTILLRQMKLIDKLLDEIKKDWSIATEENRRIFSRT
ASVEHKLTSIIAITIYSGGFFYRMILPFSKNKIVSNNMTIRLLPCPGYFGSLDEQVSPNYEIIFI
LQVFGGFVIYTAVCSTKSICLMLCMHMCGLLRILTNKVMELTNDNDERVVQEKIVHIVEY
QMKIKEFLKQIDQFVPTIYLFEVFIQVLIMCIIGYCIIMEWKESNGMGLITYVIVQMTCLIGS
FSVCYVGQLLIDESENIRQAFIALKWYQLPVKKSRSLILLIIISNYPIKVTAGKIIDLSLVTFITI
IKTAVSYMNMLQQIT
>AmelOR11
MVQIRNAKEGLKHTFWFAYPFSRTLGYWPLVSPSAFTKFFNSFTIFTLYFLELIVLIPGLLYV

LQVKNPRTKIKLLMPHLNSIAQMAKYTIILQRAKEFSKLLDEIKKDWLLATEENRQIFSERA
SIEHKLTTVIVVTMYGGGFFYRTILPLSKGKILLPNNMTVRLLPCPSYFGSLNEQATPNYEII
FTLQVLGGFIIYTVLCGTKSACLMLCLHMCGLLKILTNKVMDLTNDSDEQVVQEKIVHIVE
YQTRIKEFLNQLDQFVPAIYLIEVVIQVLIICIIGYCIIMEWEDSNAMAMVIYVVFQVTCVIG
TFSVCYVGQLLLDESENIRQAYNTLNWYRLPVKKARSLILLILMSHYPIKVTAGRIMDLSL
VTFTSIIKSAVGYMNMLRTVT
>AmelOR12
MARIRNAKDGIRHTFWFAYPFSRMLGYWPLSVSSSAFAKISNYFIIFLSYLLTLIFMVPGLLY
IFLKVKNGRSRIKLLMSHINGIVQMAKYTILLRKTKEIAKLLDEIKKDWMTASEENRQIFST
RASIEHKLTMVVVVTMYGGGFFYRAILPLSKGKIVLSNNVTIRLLPCPGYFGFLDEQVSPN
YEIIFTLQVLGGFVIYTAVCGTKSICLMLCLHMCGLLKILTNKVMELTNDKDEKVVQEKIA
HIVDYQTRIIEFLNDLNQFVPSVYFFEIILEVLIICIIGYCLITEWEDNNTMATVIFVIFQITCFI
GTFAVCYAGQLLVDESENVRQACSTLNWYRLPVKKARSLILLILMSNYPIKVTAGRIVDVS
LVTFTSIIKNSVGYMNILQQVT
>AmelOR13
MGQPYSLKLVYPLLKILGAWPKSSPSSVLSTILKCCLISICYLIQLMVLIPGILYIFLKEANLG
GKIKMFVPHMNGITQVSKYTILLRQIKEFNIILKEVKRDYSLATDKNMWIFTTRAYIGHKM
MIAIAIAMYSSGVGYRMILPFLKGRILLPDNTTVRLLPCPGYYMFLNEQVTPNYEIIFTIQV
LGGFLNYTTLCGTTGITTMLCLHMCSLLEILINKMNDLTCQSDECEIIVRKKLADIVEYQM
KIIDFLNHVEQLTSYLYFCEILEYVCGACVIGYCLITEWENSNAAALIVYFILEFLCIFCTLTI
CYIGQLLIDESDKVRQISVTLDWYRLPVNEARGLILVIIMSNYPIKVTAGKIVDISLITFTDIV
KTSVGYLNILRTVA
>AmelOR14
MSRVGKAENGMRHTVWFAYPLLRILGAWPNRVSSSTLSKIFNWYLIFTCYTLQLIVLVPGF
LHVFLKEKNGRKKMKMMIPQVNGYLQLCKYSLVLRWTNKLRVLLNEMKEDWLNTTEE
DQLIFRAKASFGHRVMSMIAIVTYSAGLGYRTILPLSKGRILLPNNTTKRLLPCPGYFVFFN
EQVSPIYEIIFIIQVLGGLLTYTIMCGTIGMCVMFCLHSSSLLRILLNKIYQLTKQLDVNEVV
VHEKIVDIVKYQTKVKGFLKNVEQLTTYLFLLEIMVETSIGCVIGYNVVTEWEDSNAAAM
IIHLMMQVSTISCTFIMCYVGQTLIDEGNNVRRMSITLDWYRFPVKEARNLILVIIMSSYPV
KLTAGKVVDISLATFTDIIKTTVGYLNMLQKVT
>AmelOR15
MSRIGNAEDGMRHTIWFAYMLLGKLGAWPNRATSSTSFSRTRNCILIFMCYSVQLIILIPGL
LHFFLKEKDSRKKVKILIPLINGYLQLCRYSLVLRSANKLCHLLNEMKKDWMNISEEDRLI
FRRKASIGHRLMSVVAIIMYSAGLGYRTFIPLSKGRILLPDNTTIRLLPCPGYYIFFNEQITPN
YEIVFTLQVIGGLLSYTIMCGTTSMCAMLCLHATSLLRILVKKINELTKQPDINESAVHMKI
TDIVRYQTKIKQFLNDVEHITTYLFLLEIIDETGIGCVIGYCAITEWEDSDATAAIIYLLLEAS
VFGVTFTMCYVGQILIDEGNNVRRMSITIDWYRFPAKEARNLILVIIMSSYPVKLTAGKVV
DISLSTYTDIIKATVGYLNMLRKVT
>AmelOR16
MENISGIAKAEEDLKYATRFVKPILATIGAWPISSSTSFFLKALQRLGHIFTYFLFFLIMIPTLA
YVFLKEKNSKVRLKLMGPIINCSMQFFKYTIIIWRRKEIQEGLHAIRHDWIQATEEERLIFRS
KMKIGRRVVLIAAFTMYGGGLCYRTILPLLKGTVITADNITIRPLPCPSYFIIINEQQSPIYEIL
FVLQVMAGMAIYAVISGTCGISALLVLHACSMLRILVNKIKKLVNKSDMSEVTLQRKIMDI
VEYQMKIKRFLKNIETVTEYICLIEMIGGTCLMCLVGYCILMELENTNTMAVVVYITLQISII

FCVFILCYIGQMLVDENYIVSQASSTINWYRLSIKNMRCLILIIAMSNYPMKLKAAKMMEMSLTTFTDVMKMSMGYLNILREVI

>AmelOR17

MENISGIAKAEEDLKYATRFVKPIMGMIGAWPISPSTSFLLKVLQRLRHIFTYFLFFLIMIPTLMYVFLKEKNNKVRLKLMPPIINCSIQCFKYTIILWRRKEIQEGLYAIKHDWIKATEEERLIFRSKAKIGRRVVLVVAFTMYGGGLCYRMILPLLKGTIVTANNTMIRALPCPSYFFILNEQQSPIYEILFVLQIIAGIAIYAVICGFCGIFALLVLHAWSMLRILVNKIKKLVDKSDMSEVVLQRKIMDIVEYQMKIKRFLKNIETITEYICLIEMIGSTCMICLVGYCILMEWENTNTMAIVIYITIQISIIFCIFILCYIGQLLVDENYIVSQASSTINWYRLSIKNMRCLILIIAMSNYPMKLKAAKMMEMSLITFTDIMKVSMGYLNILREII

>AmelOR18

MNAEKLMIEGKPPNANYKNDLSFNVRLNVWTLRTIGTWPRSPDHSWLETLEHVCLNLFCYELLAFILIPCSIYIILEIKDFYNQLKLGSALSFFLMAVMKYCVFIIREDDIRKCVELIENDWKNVRYQEDRKIMLENASFSRRLIVICGTFMYGGVIFYYIALPLTRAKIVEEGGNLTYRRLVYPFPKVLLDARHSPINEICYTIQLLSGFVAHNITVAACGLAALLAIHACGQLQILMSWLEKLVDGRKNDNENLDQRLANIVKQHVRIINFIALTEDLLHEISLIEVVGCTLNICFLGYYSMMEWDSKQPVSGVTYIILLISVTFNIFIFCYIGQLLAEQTVKVGEKSYMIDWHRMPWKKSLAIPLMISMSHSTTKITAGNIIELSISSFGDVIKTSVAYLNMLRTFTT

>AmelOR19

MNMEHFIVEKKSYNASYKNDLFFNVQLNVWTLRTIGTWPKSLDRSWLETIEHVCLCFLNYVLLAFILIPGVMYFLLEMKDFYDQMKLGSALSFFLMAVMKMCVFIIRENDIRKCIECIEDDWKNVKYQEDRKIMLENASFSRRLIVICGAFMYGGVVFYYIALPFTRAKVVEEGGNLTYRRLVYPFPKALLDARRTPANELLYTIQLLSGFVAHNITVAACGLAALLAMHACGQLQILMSWLEKLVDGRENDDENLDQRLVNIVEQHVRIINFITLTEDLLREISLVEVVGCTINICFLGYYSMMEWDTEHLIRGMTYIILLTSVTFNIFIFCYIGELLAEQTVKVGEKFYMIDWYRMPWKKSLAISLIISISRSTTKITAGNIIELSISSFGAIIKTSFAYLNILRTLTS

>AmelOR20

MEKSKDKANQKFYLTDYEYQKNVNLSIQYNRWLLKPMGLWPNSYTSKDYPYWLINIVCYCLISFLFIPCTLYLFLEIEDFYGKLKQFGPLIFCMMAFVKYYYLIFHKTDIRECVERIKWDWRNITYAKDREIMIMYANFGRKLVMVCTFFMYSGFAFYYIAIPISVGRVKTDNLTFVPLVFPFSRFIVDTRYSPTNEIVFSIQLMAGALMHGITSAACSLVATFAVHACGQMQVLMNWLQHLIDGRLDMDERLDGRIADVIRQHVRVLKFLALTEKTLQQISFTEFLGCTLDICLVGYYVIMESKSNDVTSVITYIILLISLTFNIFIFCYIGEIVAEECRKIGEISYMIEWYRLMGNKKLFCILIIAMSNSSIKLTAGNIVNLSISTFTDVVKTAVTYLNVLQKTT

>AmelOR21

MSSVKIDQDYKSNVNLSIKYSRRISKMIGLWPIFDKISTIHKFLRMLYNTICYCLLMFMIVLGWMYIAFEVKNIYDGLKFVSLMSFCMLSITKYHLINIHKDDVRECVKRIEWDWKNISYSEDREIMLMNANFGKRLIIVTTTVTYSGFVFFYIAIPMKIGKIPAPDANISFIPTMFPFPKYIADVRYSPINEIVFFFQFMCGFLVHGVTSSACSLAAIFTVHACGQIQVMMIWLEHLIEGRLDMCYSVDQRIAKIVSQHVRILKFLSLIEKILQQVSYMEFLECTVNVCLLGYCAIIEWESNHLTEVVTYVIILITIIFNIFVFCYIGELLADQSRKIGEVTYMIEWYRLSGKKKLCCVLIIAMSNSSMKLTAGNLIELSMSTFSDVVKTSFAFLNVLRTLT

>AmelOR22

MEKSKINSISCIQTNHDYKRNVNLSIQWSRWILKPIGLWPNSSTISTTGKYLYRLINVICYSL

ISFLSIPCSLYVILEVEDIYNRIKLFGPLSFCVMAFLKYHLLILHKDNISECIKRIEWDWKNIT
YSKDIEIMITNANFGRRLVIICTFFMYSGFAFYYIAVPISVGKILAEDDNITFIPLVFPFSRFIID
TRYSFINEIVFSIQLIAGALMHTITTAACSLAAIFAVHACGQMQVLSNWLKHLINGRSDMY
NNVDSRIASIVSQHVRILKFLALTEKALQQVSFVEFLGCMLNICLLGYYVITEWSSSHLTSA
ITFFILLISLTFNIFIFCYIGELVAEQCKKIGEISYMVDWYRLEGNKKLCFVLIIAMSNSSIKLT
AGNMVELCLTTFSDIVKTAVAFLNVLRTLTI
>AmelOR23
MSSVKINQDIKNNINFSIKYSRLILKMIGLWPIFDKSSTIHKYLQWLYNVICYSLIMFIIISGW
IYISLEVENIYDRLKFVSLMSFCMLSITKYHLINIHKDDVRECVKRIEWDWKNISYSEDREI
MLMNANFGKRLIIVTTTVTYSGFVFFYIAVPMKIGKIPAPDANISFIPTMFPFPKYIADVRYS
PINEIVFLAQFICGFLLHGITSSVCSLAAILTVHACGQIQVMMVWLKHLIDGRLDMCNSIDQ
RIATIVNQHVRILKFLSLIEKILQQVSYMEFLECTMNVCLLGYCAIMEWESNHLTEVITYLI
LLITIIFNIFIFCYIGELLANQSRNIGEVTYMIEWYQLFGKKKLCCVLIIAMSNSSTKLTAGNL
IELSMSTFSDVIKTSFAFLNVLRTLT
>AmelOR24
MSYTKTDHDYKRNVNLSIQWSRWILKPIGLWPNSSTISTTGKYLYRLINVICYSLISFLSIPC
SLYVILEVEDIYNRIKLFGPLSFCVMAFLKYHLLILHKDNISECIKRIEWDWKNITYSKDREI
MITNANFGRRLVVICTFFMYSGFAFYYIAVPISVGKIPAEDDNITFIPLVFPFSRFIIDTRYSFT
NEIVFCIQLVAGVLLHTITTAACSLAAIFAVHACGQMQVLSSWLKHLINGRSDMYNNVDSR
IASIVNQHVRILKFLALTEKALQQVSFVEFLGCMLDICLLGYYVIMEWSSSHLTSAITFFILL
ISLTFNIFIFCYIGELVAEQCKKVGEISYMVDWYRLEGNKKLCFVLIIAMSNSSIKLTAGNM
VELCLTTFSDIVKTAVAFLNVLRTLT
>AmelOR25
MEKQQYVIAQDDGKKANLSIQWNRWLLTPIGAWPNLRKSRIGKCYSLLISIICYGLIGFML
VSCSMFLMVEIKKVYNRIKMIGPLSFFLMTFMKYYLLLLHENDIREGIECIEWDWKNMKH
QEDRNIMIEYANYGRKLVLICTFFMYSAFAFYYLVLPFSVGKIEDGNLTFIQLPFPSSSLIADI
RYSPYNEIVLSVQILTGVVMHAITSAACSIAAVFAVHACGQMQVLMNWLDHLVDGRSDM
SKAIDDRIANIVIQHDRILKFLALTEKALQQISFVEFLGCTANMCLLGYYLIVEWNPKEIILS
VTYVALIISITFNIFIFCYIGDGVAEQCQKVGEMAYMIEWYRLTGKKKLCCILIIAMSNSSVK
FTAGNMVELSIYTFSDVVKTSVAFLNMFRALT
>AmelOR26
MMNQLNEQSVLMPVSYARDYEYSIQVNRWLLKPIGAWPNLTKATRTEKLLVKLLNFICHS
LIIFTVMPCIMYIFYEDESLKTRMKAIGPTSHWLMGELNYCCLLMRAKEIVYCIEHIKYDW
KTVRRARDRELMIKNAKLGRFIACIAALCMHSGIMSYTVITGFKKITFQIGNDSYSMYRLP
CPFYTNLLDVRFSPMNEIVFALQLLSGFISTSVTVGACGLAAVLAMHACGQFNVVMIRSD
KLVKDNNEKKQDEQTLHKKLGFIVEHHLRTLSLVWYMEKVMNMICLVELVGCTMNMCI
LKYYFLTEKSKTILGIYAIVYASMVFNIFIFCYIAEIVTEQGKKVGEKFYMTEWYQLPHKTA
LGLVLIISRSSMVIKITAGKLIQISIATFAAVFKASFAYLNMIRTIAM
>AmelOR27
MMNQTAITEEIKTNSDYSLQLNRWFLKPIGAWPLFSTTTKFEKTVSLILNIICYAIVILCATPS
LMQIILAEESFYLKLKTLGPVSHWFVSTVNYTALLMKSKDIRYCFEHMEADWQTIKRMED
QQTMLKNAKFGRYVAASCAIFMQGGILCFCFVTILTTETIQVGNETRVLHVLPCAVYKKLV
NVEENSINIFMLCFQFVAAAIANSSTVGIFSLAAVLAAHAYGQLSVVMVWITEFVNQSRNQ
KKTDDFKEIGIIVERHLRVLNFITYLENIMNRIYFLELFRCTMIICIVGYYILTEWAEKNVQN

LTTYFMMLLSICFNIFIICYIGEILTEQCMKIGEVVYMTDWYYLPDKTILNLILIILRSTVVVQITAGKLFNMSIYTFGDVLKTAFAYLNLLRQMT

>AmelOR28P

MSNRSVAIKTDPDTNSDYCLQLNRWFLKPIGAWPSFPSTTKHERIISFLLNVSCYSSLLFTLIPCLLHMLLEDESFYLKMKVLGSLAHWFVGTMNYTTLLLRGKEIRLCVEHIRTDWQTVTREEDQQVMLKNAKFGRYVAAVSAAILQSGVNCXCCMTISRTELIQIGNETRIVHVLPCAVYRKLIDVTHSPNSELIIASQFLSGFIVNSSTAGIFSLAAILGAHACGQLSVVMTWITEFVNKSKKREKMIFREIGLIVEHHLRTLNFISCIEETINRIIFLEVFRCCLHICCLGYYILMEWSDYDKRSMIIYFMLFVSVCFNIFIICYIGEILAEESMKVGEVVYMTDWYYLPDKTILDLTLIIARSSVVVQITAGKLIHMSIQTFTDVIKTGFAYLNLLRQVT

>AmelOR29

MKNQQVVITQDDYKRKTNLSIQWNRWLLTPIGAWPNLRKSRIGKCYSLLISIICYSLIGFMLVSCSIFLMVEINNIYNKLKMVGPLSFFVMTIMKYYFLLFHENDIREGIERIEWDWKNVKHQEDRNIMITYANYGRKLAFICFFFMLCAFIFYFLIQPFGGGKIVDGNLTFIQLPFPISILIADVRDSPYNEIMLSIQILTGIVMNAIRSAICSVAAVFAIHACGQMQVLMNWLNHLVEGRSDMSKKIDDRIANIVIQHDRILKFLALTERALQQISFVEFLGCTANMCLLGYYLIVEWNPKELIVSFTYIAIIASITFNIFIFCYIGELVAEQTEKVGEVAYMIEWYRIRGKKKLCCVLIIAMSNSSIKFTAGNMVELSIYTFSDVVKTSVAFLNMLRALT

>AmelOR30

MEKNRSIIGHDDYERNVNLSIRWNRFLLKSLGTWPNLRESRIGKCYSVLIGIVCYGLISFMLTSSNMFLVVEVKDTYNRIKMIGPLSFFAMTLIKYYFLTFHEENIRKGIEHIEWDWKNVKHEEDKRIMIEYANYGKKLALISIFFVYSAFVFYYFVVPISVGKIRDENLTFIPLPFPSSKLIADMRQSPANEILFSVQVLSGVIIHAITATAVSIAAVFAVHACGQMQMLMNWLECLVDGRSDMNKIVDKRIAKIVVQHDRILKFLALTERALQQISFVEFLGCTMNMCLLGYYLIVEWNPKEISLSLTYISLLISFTFNIFIFCYIGDLVAEQCQKVGEMTYMIEWYRLTGKKKLCCVLIIAMSNSSIKFTAGNMVELSIYTFSDVVKTSVAFLNMLRALT

>AmelOR31

MTSKSVISEESFDSLCDYSLQLNRWLLKPIGAWPSSSSSSKLERIVSFFLIVLCYGFILFTVIPSLFHIVLEDENLHMKLKVFGPLSHWFIGGINYTTLLLQNKEIQYCVEHMQTDWKIVNRAKDQQVMMKYAKIGRYIAALCAIFMQTGVLTYCVVTAFSTRIIEIGNETRIVHMLPCPVYKELISIDTSPTNEIVLISQFVSGFIVNSIAVGAISIGAVFTAHACGQLTIIKRWIREYINRSKDNNKNVVINEIGEIVEYHLRILNFIEGIEDVLNRFCFMELFKSTLDISMLGYYILTEWADHDIRNLTTYFMILTSMSFNIFIICYIGDILMEQCRKVGEVLYMTNWYYLPYKDILDLILIISRSNAVIKITAGKLTNMSIYTFGNVMKTTFTYFNLLRHVT

>AmelOR32

MIDKFASIQQTNNNLSNYSIQLNRWFLKPIGAWPPSPSTTKLEKIISIVLIICCYSSICFTVIPCLLHVMLEDESFRDKLKVLGPLSHWFIGAINYTTLLLRSKEIRYCIEHMQRDWRIVTRTEDQQIMMKHAKIGRYIAVFSAAFMQGGVLSNCAVTAFSTQTIEIGNVTKTIHMIPCTAYKKLIAVDTSPTNEIVIASQFLSGFIVNSSAVGAVSIAAVFAAHACGQLSLLMVWIREFVDHSKKIHDKNIGLNKIGKIVRHHLRTLSFVTGIENVMSGICFMELFKCTVNICMLGYYILTAWSVHDIQNMVVFLVILLSMIFNIFIICYIGDILTEQCKMIGEAVYMTNWYYLPGKDILNLVQIILRSSMVIKITAGKLVHMSIYTFGNVMKTAFAYLNLLRQMT

>AmelOR33

MMTSKSVPIEQDNHSLSNYSVQLNRWFLKSIGTWPLSPSTTKLEKTISFLLIICCYCFICFTV

IPCLLHIILGDDSFREKLKVLGPLSHWFIGGINYTTLLLRKKEIRYCIKHVQRDWRIVTRME
DQQVMIKHAKIGRYISMMCAAFMQGGVLSYCAVTAFSTQTIEIGNETRIVHMIPCIVYKKL
IATDTSPTNEIVIASQFVSGFIVNSSAVGAVSIAAVFAAHACGQINLLMAWIRQLVNHSNVN
NKNVGLDKISNIVRHHLRILSFITGIENVMSGICFMELFKCTMNICMLGYYVLTAWIDNDM
RNLIVCSVILFSMIFNIFIICYIGDILTEQCKMIGEAVYMTNWYYLPGKDILDLIQIILRSSMVI
KITAGKLVHMSIYTFGNVMKTAFTYLNLLRQLT
>AmelOR34
MMIDKFVPIEQDNHSLSNHSVQLNRWLLKSIGAWPSFSSTTKLEKIISFVLIICCYCFICFTVI
PCLLHVILEDDSFHEKLKVLGPLSHWLVGGINYTTLLLRNKEIRYCIEHMQRDWEIVTKTE
DQQVMIKHAKIGRYITMFCAAFMQGGVLSYCAVTAFSTQTIEIGNETRIVHMIPCVVYKKL
IASDTSPTNEIVIASQFVSGFIVNSSAVGAVSIAAVFTAHACGQVSLLMAWIRQFVDHSNIQD
KNIVLNDIGEIIRHHLKILSFITGIENVMSGICFMELFKCTVNICMLGYYILTAWTGHDIQSLI
VFSVILFSMIFNIFIICYIGDVLTEQCKMIGEAVYMTNWYYLPGKDILNLIQIILRSSMVIKIT
AGKLVHMSIYTFGNVMKTAFTYLNLLRQMT
>AmelOR35
MLVLKDSSSVSYSKDWIYSVQINRWLLKAIGIWPLSLCVTTTEKIHSVILTLISIFLIGFLLVP
CTLCTLLDKTGDLDTKIKMIGPFSFCIMAAIKYYVLLSRGSHIGKCIEDIRVDWFRVSSHNC
LEDRKIMMENARIGRSLAIFCAGFMYSGGFFYTTVMPLCTKRTEIIDNEIVRSQAFPIYRGL
LDPRTSPSFEIVQLMQCLAGFVIYSVTVGSCSLAAVFVMHACGQFQILVTKLRRLIDGLKE
DKDMENIVHEQRLGNIVEHHLHILGFISQIEELLNEICFVEFIGCTLNICFLGYFLLKEWEQS
ETIGILTYCILLISFIFNIFILCYIGEILSEECKSIGLSAYMIDWHRLPGKKALSLILISAASNSST
KLTAGKLVELSLSSFCSVLKSSLAYLSLLRTLTT
>AmelOR36
MTDDISAIQKKFGSLNEYSIQVNRWLSKTIGVWPLPSSTSKFEKITTRILILFCWTIAVLDTT
SGLLHFVLVKEDIIIKLKSLAPISYILGGGLNYAVLLLRKNDIRYCIDRIEADWKVITRMADR
QVMLKNAKIGRIISCCIVGFMQLGTFCFCTILGVFKRTIKIGNDSMEIYVLPSPTYKIPVDTN
PGHDIVLGFQYVAAYITSATVISAFSFATVFACHASGQLTIMIIWIEEFINRSQKENKNRIDEIS
VIIEHHMRILSFLERAEHLLSPICFMEMFKNILSICLFSYCILAEWSEHNIRILSTYILAVINITL
NTFLICYIGEVLTERCKEIGNMVYMTNWYRLPKKDILNLIMIITRSSVEYKMTAGKIIDMSV
ITFGNIIKTVFGYLNILRQVTML
>AmelOR37
MMADDIATVQKEFENLNEYSIQFNKWFSKTIGVWPLPSSTSKFEKIMTRILILFCWIIALFD
AISGLLHFVLVKEDIIIKLKSLAPISYIFGGGLNYAVLLLRKDDILYCIEHMETDWKTITRMT
DRQIMLKNAKIGRIISCCILAFMQVSAVCFCTVLGVFKRTIKIGNESMEIYVLPSPIYKIPVD
TNPGHDIVLGFQYLAAYITSATVVSAFSFATVFACHASGQLTIMIIWIKEFINRPQKENKNRI
DEISVIIEHHMRILSFLERAEHLLSPICFMEMFKNILSICLFSYCILAEWSEHNIRILGTYIFAVI
NITLNTFLICYIGEVLTERCKKIGNMVYMTNWYRLPKKDILNLIMIITRSSVEYKITAGKIID
MSVITFGNIIKTVFGYLNILRQTTML
>AmelOR38
MMADDIATVQKEFNNLNEYSIQFNKWFSKTIGVWPLPSSTSKFEKIVTRILIIVCSIITLFVII
PSMLHFILVKEDIISKLKSLGPISYCFGGGLNYAVLLLRKNDIRYCIDHIETDWKVITRMTDR
QVMLKNAKIGRIISCCIVGFLQIGTFCFCTILGVFKRTIKIGNNSMEIYVLPSPAYKIPVDTNP
GHDIVLCFQYLAAYITSATVVSAFSFAIVFACHASGQLTIMIIWIEEFINRPQEENKNVHIDKI
SVIIKHHMRILSFLERAEHLLSPICFMEMFKNILSICLFSYCILAEWSEHNIRILGTYIITVINIT

LNTFLICYIGEVLTERCKEIGDMVYMTNWYRLPKKDILNLIMIITRSSVEYKMTAGKIIDMS
VITFGNIIKTVFGYLNILRQTTML
>AmelOR39
MMADDIATVQKEFDNLNEYSIQFNKWFAKTIGVWPLPSSTSKLEKIMTRILILFCWITTLFV
TISSLLHFTLVKEDIIIKLKSLAPISYCFGGGLNYAVLLYRKSDILYCIEHMEVDWKAITKTA
DRQIMFKNAKIGRIISCCIAAFVQISAVCFCTVLGVFKRTIKIGNESMEIHVLPSPTYKIPVDT
NPGYGIILGLQFLTGYIMSATVVIAFSFATVFACHTIGQLTIMVTWIEEFINRPQEENKNVHI
DKISVIIKHHMRILSFLERAEHLLSPICFMEMFKNILSICMFSYCILAEWSEHDIRILTTYTFA
VMNLIFSTFLICYIGEILTERCKEIGNMVYMTNWYQLHDKDILNLIMIIVRSSVEYKMTAGK
IMDMSVITFGNIIKTVFGYLNILRQTTML
>AmelOR40
MADDITAIQKKFGSLNEYSIQLNRWLSKTIGVWPLPSSTTKFEKIMTKILIFLCWIIALFVITS
SLLHFTLVKEDIISKLKTLGPISYCFGGGLNYAVLLLRKDDIRYCIDHIETDWKAITRTGDRQ
VMFKNAKIGRIISGCIASFMQVSTICFGIVFGVFKQKIKIGNESMEIHVLPFPTYKIPVDTNL
EHSIVLGFQFLTGCIMSATVVIAFSLATVFACHAAGQLTIMVTWIKEFVNRPQEENKNMRV
NEISVIIEHHLRILSFLGRTEHLLSPICFMEMFKNVLSICMLSYCILVEWSGRDIRALSAYTFS
VMNIALSTFLICYIGEVLTEKCKEIGNMVYMTNWYRLSDKDILNLIMIITRSSVEYKMTAG
KIIDMSVITFGNIIKTIFAYLNILRQMTIL
>AmelOR41
MADDIVAIQKKFGSLNEYSIQVNRWLSKTIGVWPFTSTTSKFEKIMTKILIIVCSIIALFVTVP
SMLHFILVKEDIITKLKMTGPIIYCIGGGLNYAILLFLRDDIRYCIEHIEADWKTITRTGDRQV
MFKNAKIGRIISGCIGSFLQFSTISYCTVFGVFKQTIKIGNESMEIHVLPFPTYKIPVDTNLEH
GIVLGFQYLTACIMTATIIIAFSLATVFACHAVGQLTIMVTWIEEFVNRPQEEKKNMRINEIS
VIIEHHLRILSFLERTEHLLNPIYFMEMFKNILTTCMLSYCILVEWSGHDIKVLSAYSFTITNII
LSLFLICYISEVLNEKCKEIGNIVYMTNWYRLSDKDILNLIMIIIRSSVEYKMTAGKIIDMSVI
TFSNIIKTIFAYLNILRQVTIL
>AmelOR42
MADDIVAIQKKFGSLNEYSIQVNRWLSKTIGVWPFTSTTSKFEKIMTKILIIVCSIIALFVTIP
SMLHFILVKEDIITKLKMTGPIIYCIGGGLNYAILLFLRDDIRYCIEHIEADWKTITRTGDRQV
MFKNAKIGRIISGCIGSFLQFSTISYCTVFGVFKQTIKIGNESMEIHVLPFPTYKIPVDTNLEH
GIVLGFQYLTACIMTATIIIAFSLATVFACHAVGQLTIMVTWIEEFVNRPQEEKKNMRINEIS
VIIEHHLRILSFLERTEHLLNPIYFMEMFKNILTTCMLSYCILVEWSGHDIKVLSAYSFTITNII
LSLFLICYISEVLNEKCKEIGNIVYMTNWYRLSDKDILNLIMIIIRSSVEYKMTAGKIIDMSVI
TFSNIIKTIFAYLNILRQVTIL
>AmelOR43
MMADDIAAIQKKFGSLNEYSIQLNRWFSKTIGVWPLPSSTSKLEKIMTKILIFLCWIIALFVI
ISSLLYFALVKEDIISKLKTLGPISYCFGGGLNYAVLLLRKNDIRYCIDHIETDWKAITRTGDR
QVMFKNAKIGRIISGCVAGFLQLSTISFCTVFGVFKRRIKIGNESMEIYVLPFPTYKIPVDTN
PGHNIVLGFQFLAAYIMSATVVIAFSLATVFACHAIGQLTIMITWIEEFVNRPQEENKNMRV
NEISVIIEHHLRILSFLGRTEHLLSPICFMEMFKNILSICMLSYCILAEWYGRDVRVLGAYAF
SVTCITLNTFLICYIGEVLSEKCKKISNMIYMTNWYRLSEKDILNLIMIMIRSGMEYKMTAG
KIINMSVVTFGNIIKTILAYLNILRQMTIL
>AmelOR44
MADDIVAIQKKFGSLNEYSIQVNRWLSKTIGLWPLTSTTSKFEKIMTKILILLCWIIALFVTT

LSLLHFILVKEDIITKLKMIGPISYCVGGGLNYAVLLFLRDDIRYCIDHIETDWNAITRTQDRQVMLKNAKIGRIISGCIAGFMQLDSICFCTVLGVFKQTIKVGNESIRVYILPYPTYKVPVDTNPGHSILLLLQFLTTCIMSTTVVIAFSLATVFAYHAVGQLTIMVTWIEEFVNRPQEEKKNMRIDEISVIIEHHLRILSFLGRIEHLLSPICFMEMFKNILSICMISYCILAEWSGRDVRALSTYAFCVTCIILNTFLICYIGEILSEKCKKISDMIYMTNWYQLSDKDILNLIMIMIRSGVEYKMTAGKIVNMSVITFGNIIKTIFTYLNILFQMTML

>AmelOR45

MADDIAAIQKKFGSLNEYSIQLNRWLSKTIGVWPLSSSSSKFEKIMTKILIFLCCIIALFVIIPSLLHFTLVKEDIISKLKTLGPIGYCFGGGLNYAILLLRKNDIRYCIEHMKADWKAITRTDDQQIMLKNAKIGRIISCCFAAFMQFSTVIFCAVFGVFKRTIKISNESMEIYVLPFPTYKIPVDVNPGHNIVLGFQFLAGYITTGTVIIAFSFATVFACHAVGQLTIMITWIEEFVNRPQEENKNVRVEEISVIIEHHLRILSFLERTEHLLSPICFMEMFKNILTICMLSYCILAEWSGHDIRALSAYASAVMNISLGTFLICYVGEILTEKCKEIGNMVYMTNWYRLPKKDILNLIMIITRCSMEYKMSAGKMIDMSVITFGNIVKTIFAYLNILRQMTIL

>AmelOR46

MSDDLVEVEKKFGSLNEYSIQFNRWILKPIGAWPISLCTTRNEKIISKILIIVCWSLSLFTLIPGLLHFILEKEDTYLKLKTIGPLSHWVIGGFNYAVLLLRKNDILHCIEHIRVDWNIITKKQDQQVMLKYAKIGRYIAAFCTAFLQGGVLCTCIALGAFKTTIKNGNETIEIYSLLCPAYKLPVQTNPTHDIILGTQLLSAFITSSSAAGAFSLAAVFASHALGQLNIMVAWINEFVNRPIDLNNVYVNKISIIVEHHLRILSFITHIEHLMNPICFMEMFKCMVGMCMPSYYILAEWSEHNVQNLAVYVMIIISMTCNIFLICYIGEILTEQCKKIGEIIYMTNWYELSNKDIFNLMMIISRSSISVNMSAGKLIDMSVLTFGNTVKSVFVYLNMLRQMTMI

>AmelOR47

MADDIVAIQKKFGSLNEYSIQLNRWFSKTIGVWPLPSSSSKLEKIITKILIFLYWIIVLFIIITSLLHFILVKEDIVSKLKSLGPISYCFGGGLNYAVLLLRKNDIRYCIDHIEADWKVITRMGDRQVMLKNAKIGRIISCCIVCFMQIGTLCFCTILGVFKRTIKIGNDSMEIYVLPFPTYKIPVDTNPGHAIILGLQYLTSFIMSATVVIAFSLATVFACHAIGQLTIMISWIQEFVNQPQKQNKNIRIDEISVIIEHHLRILSFLERTEHLLSPICFMEMFKNILSICMFSYCILAEWSEHDVRVLGIYAFAVICITLNTFLICYIGEVLTERCKEIRNMVYMTNWYRLPDKDILNLIMIITRSGVEYKMTAGKIIDISVITFGNIIKTVFAYLNILRQMTIL

>AmelOR48

MADDIVAVQKKFGSLNEYSIQLNRWLSKMIGVWPLPSFTSKFEKIMTKILIFFYWIILLFIILASSLHFLFVKEDIVSKLKTLGPISYCFGGGFNYAVLLLRKNDIRYCIEHIETDWKIIKRMEDQQVMLKSAKIGRIISGCIAGFMHIGTFCFCIVLGVLKRTIKIGNDSMEMYVLPFPTYKIPVDTNPGHGIILSLQYLTSYTSSATVVIAFSLATVFAYHAIGQLTIMISWIQEFVNQPQKQNKNIRIDEISIIIEHHLRILSFLERTEHLLSPICFMEMFKNILSICMFSYCILAEWSERDIRVLGIYTFAVMNVILSTFLICYIGEVLTERCKEIGNMVYMTNWYHLPDKDIFNLIMIIVRSGVEYKMTAGKIIDISVITFGNIVKTVFVYLNILRQMTIL

>AmelOR49

MADDLAKIEKKFGDLSEYSIQFNRWILKPIGAWPASFYKSRIEKIVSKILIVICWISSLFTLIPGVLHFFLEKEDIYVKLKILGPLTHWLVGGFNYAVLLLRKDDIYYCIKQICADWNIITKKQDQQVMLKNAKIGRYVAVFCTVFLQGGVFCTCLALGAFKKTIKVDNETVNIYNLPCPAYNMPVDTNPTHDIILGTQLLSAFICSSSTAGAFSFVAICASHALGQLNLMVIWINEYVNRPKKLNNNAYINKIGIIVEHHLRILSFIARVEHVMSPICFMEMIKCMVGICMPIYYILMEWSEHNIQNLT

VYVMIIISMTYNIFLVCYIGEIITEECKKIGDIVYMTNWYELSDKNILNLMMIITRSSMNINM
TAGKMTNMSVLTFGKIVKSIFAYLNVLRQITMI
>AmelOR50
MTNDINVAKQRSDNLSEYSIKLSRWYLKPLGAWPASSSTTKMERIISQILIVICWCIILFTVIP
GILYILFVKQDIYVKLKIFGPLSHWCIDGFNYAILLLRKNDILHCIEHLRADWKLITRTQDQ
QVMLRNAKMGRYIAAFCAIFMQVIIFFTCFILGIFKRSIHIDNKTVELYNLPCPAYKIPFDTD
PTIHDIMLGTQFLSAFVVSSSASASFTLATIFTCHVLGQLNIMMIWINEFVDRLQRKENKDN
HINKIGVIVEHHLRILSLIARIERITCPIYFMELFKCMMGMCMPSYYFLAEWSERNIQNLTIY
VMVALSMSFNILLVCCIGEILREQCKKVGDMVYMTNWYQLPDKDILNLIMIISRSSVEVKI
TAGKIITMSIYTFGNIVKTVFAYLNMLRQITMM
>AmelOR51
MRSTNNIDNLPLNDRYESDIQYTFQFCHWILKPLGIYYFIYNQANKFEKILSIILILICFFIIQF
VIVPFGYYILFYEKDMNTKIKFLGPLTFCLSALFKYSYLGIKSSELGHCIKHVEKDWKMLQ
NEDHRVIMSRYVIMGRNLITLCAAFMYTGGLSYHTIMPLLSKRKVENFTIRPLTYPGYEAF
LNIQKSPTYEIIYCMHCIYVIVVGNITMAAYSLTTIFITHACGQIKIQMLRLENLKNEKKVLE
TGIESHLAVVVKNHVEILRFAKNVETTLRELFLVEVIVSTLLMCLLEYYCMVEWETSDSAA
ILTYVILLFSFTFNILIFCYVGELLLGQGSEIATALYEIEWYNLPGRKARDIILLLVISKYPPKL
TAGKIFILSMNTFSVVLKSSVVYLNMLRTITEL
>AmelOR52
MFDRSYNNSQLKNIHYENDIHYTLQMCQWLLKPIGVWPFVYDRTSRFEQLISIILMATCFS
SLLFIILPSGHHIFFVEKDMHLKVKLLGPVGFCLSSTIKYCYLGVKGVFFEQCIKHVKNDW
KMVQDPSYRIIMLKYATISRKLIIMCAVFLYTGGMSYHTVMQFLSKEKDNNNTFKPLTYLG
YDPFFDTQSSPIYEIVFCMHCFAAMIMYSVTTVAYSLAAIFVTHICGQIQIQATRLQNLVEN
KDKKNNCDPFALIVHDHVEILRFSKNVEEALREICLAEIIESTIIMCLLEYYCMTEWQNND
AIAILTYFTLLISFTFNIFIFCYIGEILSEQCSQIGTISYEINWYKLPAKKAHDLILLISISQYPPK
LTAGKIIDLSFNTFSSVVKTSVIYLNLLRTVTD
>AmelOR53
MHDRSHDNINGQLKNSHYKSDIHYTLQMCQWLLKPIGVWPLIYNQTSRFEQLISIILMGTC
FSSLLFIILPSGHHILFVEKNLHMKVKAFGPAGFCLSSTIKYCYLGLKGSSFERCIEHMRKD
WMMVQDPNHRTIMLKYATISRKLITMCAVFLYTGGMSYHTIMQFLSKGKNKDNYTIRPLP
YIGYDPFFDTQSSPTYEIVYCIHCFTAMIMYSISTVAYSLTTIFVTHICGQIQVQIARLQDLVE
SKEKRKYKDCDPFALIVHDHVEVLRFSNNIEEALREICFTEIIECTIDMCMLEYYCIMEWSV
GDTITLLTFFTLLISFTFNIFIFCYIGEILTEQCSQIGTVSYEIDWYKLSPKEAYDLILLISISQHP
PKLTAGKIIELSLNTFSTVAKTSVVYLNLLRTVTDW
>AmelOR54
MHDRSYHDIESQLKNSYYKSDIHYTLQMCQWLLKPIGVWPLISNQTNKFEQLVSIILMITC
FSSLLFIILPSGHHYFFVEKNLNMKVKALGPVSFCVSSTIKYCYLALKGSSFERCIEHMKRD
WMMVQDPNHRTIMLKYATISRRLITICAVFLYSGGMSYHTVMQFLSKGKNNYTIRPLPYIG
YDPFFDTQSSPTYEIVYCIHCFTAMIMYSISTVAYSLAAIFVTHICGQIQIQIARLQKLVECKE
RKKYESCNLFALIVHDHVEILRFSNNIEEALKEICFTEIIECTLNMCMLEYYCLIEWSAGDTI
TFLTFFTLLTSFTFNIFIFCYIGEILTEQCSQIGTVSYEIDWYKLSPKEAYDLILLISISQYPPKLT
AGKIIELSFNTFSSVAKTSVVYLNLLRTVTDW
>AmelOR55
MHFSVRNLINKPRNPNYEKDITYVMKHNKWVLISIGIWPTVLKNIGKFLPKIVIGINNLMC

FFILIQSALHIILEQKDTLLRLKFFGLIFFSFMSLMKYWALTIRKPEIEHCIQQVQSDWKQVK
MENDRELMLKYGIIGRNLTIYSILFMYISGIMYISFMQYAMRLQINNDNQTNKVLIFPAYSN
SIQKSPIYEITYGIQCICGYVLDSVTSGACGLAALFVTHACGQIDVVISRLDDLVAGQFYKK
NSNPNIQVIKIIKHHIKILKFSAVVEKVLQEVFFLEFISSTFVICLLEYYCITDWEQNNIISLTS
YALLLISLTFNMFLLCYIGDLLIHKSGNIGVAVFMIDWYHLPAKTIQNLILIMAMSNSPAKLS
VGRIVDLSLSTFGNVLKTTFVYLNFLQTAVMQ
>AmelOR56
MYLSIQNPINEPRNPNYEKDIAYVTKYNKWVLTCIGIWPIILKNINKILPKIVIGINNLLCSFI
LIQSALHIIYEEKDVLLRLKILGLIFFSFISLMKYWALTIHKPEIKYCIEQVQSDWKQVEMEN
DRELMLKYGILGRNLTIYSILFMYMGSITYMSITQYAMGLQFNEHNQTIRVLIYPTYGYNIQ
KSPIYEIIYGVQFMCGYVVDTITSGACGLAALFVTHACGQIDIITSRLDDIVAGQFYSKNLN
PDIRLMGIIKHHIRILKFSAVVETILQEVFFLEFIGSTFVICLLEYYCIADWEQKNIISLTSYVL
LLISLTFNMFLLCYIGDLLIQKSSNIGVAVFMIDWFHLPTKTIQNLILIMAMSNTPAKLTVGRI
VDLSLSTFGNVLKTTFVYLNFLQTAVMQ
>AmelOR57
MHVSVRDPINELRNPNYEKDIAYVTKHNKWVLASIGIWPTVLKNIGKILPKIVIGFNNLLC
FFTLTQSALHIILEQKDTLLRLKFLGLIFFSFMSMMKYWALMIRKPEIEHCIEQVQLDWKQ
VEIENDRELMLKYGIIGRNLTIYSILFMYLSGIIYVSIMQYAMGSQINEHNQTIKMLIYPAYG
GYNIQKSPTYEIIYGVQCICEYVFDTIASGACGLAALFVTHACGQIDVIMSRLDDIVAGQYK
KNSNANIRLMEIIKHHTRILKFSAVVETVLQEVFFLEFVSSTFVICLLEYYCITDWEQKNIISL
TSYILLLISMTFNMFLLCYIGDLLIEKSGNVGVAVFMIDWYHLPTKTIQNLILIMAMSNTPA
KLSVGRILDLSLSTFGNVLKTTFVYLNFLQTAVM
>AmelOR58
MHLFVRDQTNQPRNLNYEKDIVYVTKHNKWILNSIGIWPTVLKGIDEYLPKIAIALSNLVL
SFTVIQCVLHILLEQKDPILRLKILGLTFFSFISLMKYWVLTMRKPKIKLCIEQIQHDWKQVE
FERDRKLMLKYGIIGRNLSMYSIVFMYSGGIIYHTVMHYKLGSYVDEYNRTIKLLIYPTYS
RLYDVQKSPVYELVYILQCICGYMFDAVTVGACGLAALFATHICGQIDIVMAKLEDLVDG
KFSKENSNPNIRLIEIIEHHIKILRFSAMVETVLQEVCFLEFIGSTFVICLLEYYCITDWQQNN
TIGLTTYSLLLISLVFNIFLLCYIGNLLIEKSSNIGIVCCMIDWYQLPIKTIQGLILMIAMSNSP
AKISAAGIADLSLSTFGSVLKTSFAYLNFIRTTIM
>AmelOR59
MHPITLNESDCKARNLKYKEDIAYVTKHNKWILKSIGIWPSIFKDVSKFLPKIMFGLCNFV
LFFAIIPCILYIVIEENDTMIRFKLFGLLSFCLVALIKYWTLLYRKSRIKNCVEQIWIDWEQVE
LYEDREMMLKYGQMGRNLMIICAMFTYTGGTIFHTILQYKVGTFIDEYNRTIKPVIYPTYN
GLFNVQRSPIYEFVYILHCMCGYVMHSVTAGACGLTALFATHACGQIDIVIARLNDLIHGK
YSKEKINLNARFTKIIEHHLRILRFSATVQEVLQELCFLECIGSTFLICLLEYYCITDWELNN
TISLTTYIILLISLIFNIFILCYIGELLMEKSSNIGLSCFMIDWYYLPSKTIRGLILMIAISSNPTK
ISAGGIVDLSLSTFGNVLKTSFAYLNFFRTTIM
>AmelOR60
MHLTILNESDYRARNLKYKEDIAYVTKHSKWILKSIGIWPIILKDVAKFLPKIVIGISNFVLL
FAIIPCILYIILEEKNNLIKLKLFGLLMFCSIALMKHWALAYRKPKIKNCIEQIQNDWEQVKL
YEDREMMLKYGQVGRNLTIICAVFMYTGGIIYHTILQYEIGTFIDEYNHTIKPVIYPTYSGL
FNVQKSPIYELIYVLHCTCGYVMYSITAGACGLAALFVTHACGQIDIVIARLNDLVHAKYG
KGKFNLNARLIKIVEHHLQILRFSATVQVILQEVCFLEFIGSIFLICLLEYYCITDWELKNTIS

LTTYIILLISLTFNIFILCYIGELLMEKSSSIGLSCFMIDWYHLPVKTIQGLILIIAISNSPTKISA
GGIVDLSLFTFANILKTSFVYLNFIRAAIM
>AmelOR61
MHLTTLNKNNCKVRNLKYKEDIAYITKHNKWILKSIGIWPSVLKSVSRFLPKIMFGFNNFV
LLFSVIPCILYIVYEEKNIMIKFKLVGLLSFSLIALIKYWTLTYRKPRIKDCIEQIWIDWEQVE
LHEDRKVMLKYGQIGRNLTIICAVFIYTGGSIFHTILQYKIGTFIDEHNRTIKPVVYPTYNAL
FDVQKSPIYELVYLLHSICGYIMYSVTAGSCGLTALFATHACGQIDIVIARLNDLIHGKYTK
NTFNLNTRLVKIVKHHLRILRFSESIEMALQELCFLECIGSTFLICLLEYYCITDWELSNTISL
TTYTMLLISLTFNIFILCYIGERLMEKSSSIGLSCFMIDWFQLPTKTIHDLILIIAMSNNPIKIS
AGSIVDLSLYTFGGVLKTSLVYLSFLRTTIM
>AmelOR62
MGKRKESIDERIRNFMVQKMVLKIIGIWPTNGERSFFGRWIFAVTTQIGIIYILSLEIYRHCL
DIDDTMDAFVMDLSAVISLAKLFILRLNSKHAWVLINSVVEDWSAVHDSRHEYIMTEYLK
KGRIVSLMILYLGYASGFSFIVKALPFGDILPFQMFQNSRNSSMNPDIPLKLNYFLASYCVF
GSLPLLHHVCVLLLQGIFIFVNAVAHCGNDGLFFSLTMHLCGQFEILKTRIAKIEFVDRRKI
GPLVKRHCQLAVLVNDLEQTFNMIIFVQLLMSALLICVEGFVFLVCLSTKDNIGALKSMVL
MVTLLIQLYLYAYAGDALESRTEEIAQAAFHSFWYQSRGRTARDLILIICRGNSSYHVTAGK
FVFMNIFTFKEILKSSASYLSVLKVMMDT
>AmelOR63
MLKKMKTTSNKDFAYAMTPLKFLAWPVGTWPLQVFNTFSIIRATFSTFLLLLMLTILQVEL
YLDSSNPEYNLDALILINAGILAVTKVICFHVRSLGLVSNFTSAVKDYKELNSEENRVIVRR
HAYMGRAACISLIFCSYVGCTLFMIVPIVAGDKEEVINVTEESAMKYPVPFENTLILINMPE
NMYFLIFIVEYLMLLLTTTGNLGSDSLFFSIVFHLCGQVEILRLEYNKLSNENERTTKHITLL
IKRHIYLLKLGDMLNKTISSILIVQLSSSCMLICTTGFEFILALSIGNIVMIVKTFAVICVLLIQ
LFAYSYVGEYLKTQTEGLGNSIYFCTWYDMPKNVSHNITFIIMRAQHPVLLTAGKFFVINM
ETYMSILRISMSYLSVLRVMVNS
>AmelOR64
MKTTSNKDFAYAMTPLKFLSWPLGTWPLQVFNTFSIIRAMFSTFLVLLMLAILQVELYLDR
SNAENNLDALVLINGGILAVAKVMCFHIRPLGLISNFTSAVKDYNELNSEENRVIVRRHAY
MGRVACASLIFCSYVGSTLFMTVPMLAGDEEEVINVTEESAIKYPMPSENTLTLINMPEKM
YFVIFIVEYLMLLLTSTGNLGSDSLFFGIAFHLCGQVEILRLEYNKLSNENERATKDIILLTK
RHIYLLKLSDMLNETISSILIVQLFSSCVLICTTGFEFILALNIGNIVMTIKTFIVMCVLLIQLF
AYSYVGEYLKTQTEDLSNSVYFCTWYDMPKNVTQNIIFIIMRAQHPVFLTAGKFFVVNME
TYMSILKTSMSYLSVLRVMVNS
>AmelOR65
MKTTSNKDFTYAMTLLKFLSWPVGTWPFQVYDTFSLTRTIFSISLLLLMIIIVQVELYLDRT
NAENNLDALLLINCGILAVGKVMSFRVRSTGLVFNFTSAVKDYNESNDEENRMIMRRHAY
MGRVACTSLISCSYVCSTLFITVPMLAGDEIQVINATEENAIKYPIPSKNALEIINMPDNLYF
VVFIVEYMMLLFTSIGNLGSDSVFFGIVFHLCGQVEVLKREYSKLFNKNEKITEHFILLIKR
HIYLLNLSKMLNETISSILIIQLFSSCVLICTTGFQFILALSIGNIVLTIKILIIMCVLLIQLFAYS
YVGEYLKTQTESVGNSVYFCTWYDMPKNVSKDIIFIIMKAQRPVLLRAGKIFVVNMETYI
SILKTSMSYLSVLRVMVNS
>AmelOR66
MKTTLNKEFAYAMTPLKFLSWPVGTWPFQVYDIFSLTRTIFSISLLLLMIAIVQVELYLDRT

DAENNLDALLLINCGILAVAKVMCFRIRPVGLVSNFSSAIKDYNELNSEENRVIVRRHAYM
GRVACASLIFCSYAGSTLFMTVPMLAGDEEEVINVTVESAMKYPIPSKNILAIINMPENMYF
VVFIIEYIMLLLTSTGNLGSDSLFFGIAFHLCGQVEILRLKYNKLSNENERTMKHISLLTRRH
IYLLKLSDMLNETISSILVIQLFSSCVLICTTGFEFILALSIGNIVMMIRICIAMCVLLIQLFAYS
YVGEYLKTQTESLGNSVYFCTWYEMPKNVSQNITFIIMRAQHPFLLTAGKFFVVNMETYM
SILKTSMSYLSVLRVMVN
>AmelOR67
MKTTSNKDFTYAMIPLKFLSWPVGTWPFQVHEIFSISRTIFSISLLLLMVVILQVELYLDRSN
AENNLDALLLINCGILAVAKVMCFRIRPIGLVSNFSSAIKDYNELNSEENRVIMRRHAYMSR
VACASLISCSFIASTLFMTVPMLTGDKKDIINVTEKSIIKYPIPSKNALAIINMPENLSFMVFI
VEYMMLLFTSTGNLGSDSLFFGIVFHLCGQVEILKLKYNKLSNTNERTMEHIILLTKRHIYL
LNLSKMLNETVSSILVIQLFSSCVLICTTGFQLILTLTFGNVVLTIKILAEISILLIQLFAYSYVG
EYLKTQTEGIGNSVYFCTWYDMPKNVSKDIIFIIMKSQRPVLLTAGKFFVINMETYMSILKT
SMSYLSVLRVMVNS
>AmelOR68
MTILQPIFNILTICGCRMPSSCRTSYKRMLYILYATFVLLLLYSFCISQFLNVIINVRTADELCN
SFYMFIASLLSCCKIVALLMNHKAIKIFRRKLEEEPCKPTNTKEVTIQKSFDKNIGSITIYYT
VMVEFTVFCMIVSSLVTDFRNQRLAYEAWLPFNCSAPNYYYYYIAYVHQIIALIGTSLLNV
ACDVTICGLFVHMYSQQEILKHRLKESVNVENRLNIGKIVYFHNYLYGYAFMVQEKFKKII
GIQLLSSTLVVCFILYKLANTSLISTKFLEFVLYLACMMTQIFVYCWYGNQLKLKSVEVVD
TIFELDWISLDNRSKKDLINIMRRAMNPIELTCAYIFTIDLRTFVTILKMSYSTYNFLQRTKV
N
>AmelOR69
MQLLRTIYHLLTSCACWRPPFLSPLKNFAYTVYYCYVILLIYGATFCQFVDLLLIVETEDEF
CDNFYLTLAIFISCHKMYSMLVNRENIILVNRMLESEPFQPETEEEMDMRDKCDKQARLN
AIYYAILVELSVMSLSFGGLLKAESHKLPYRMWLPYNYTSLSAHIFIYTQQVVSLIVSAMIH
VACDSFIWALLMHICSQIEIFNCRLRKIKHEKNEVTKLCIHYHNLIYRLATTINEQFKMVIFV
QFTVSTLTICVNLYILMGTQITFERIMQLAIYSSCMLTQIYIFCWYGNEVKLKSLDISNMIFE
LDWPDLDNTTKRDLLMIMMRASYPIEMTSVHVITMNLDSFVILLKTSYSAYNLLQSNRE
>AmelOR70
MQALQWTRFLLSVCGCMPPTSWKSSFKKSLYNIYTCVIWLLILSLVSTQILDIIINVKNKNE
FIENFYITLVVFVTSCKMTIILRYRKNILSLMDDLQHEPFSPMTHEENEIRTKFNKMNERTSI
CYTILVLVSATWIFVRSFFTDFKKRKLTFRAWLPYDYSELLPFALSYAHQATTSMFCSCQNI
SCDTLFAGFLVQIYCQFEILEERLKNVQQDESNYSAKQCVKHYHQIYKFSRTLNEKFKVIL
FLQFCAIAFILCFNLYRMTTITMIPKLLEASLYLIRVLVQILYYCWFSNEVKLKSLEVPGMIF
KSDWTSWDDKTKKIFLIIMTRATQPFEFTSGYLVTLNLEFFVALIKASYSVFNLLQRTK
>AmelOR71
MRILRWTFLLFALCGCFPPSSWTTRLKRYLYKIYAVFSFVALNSFLLSQILDMVYNVKGTD
DFSDNFSVTVVVFVTCFKLITILTRRENILLLCNTLKQEPLSPINTEEFEIFLKFEKLTDWNTL
GYFILLMSSSLCILMGSLLANFKIRKLAFRTWLPYDYSTASAFLLAFAYQVVVATVCTFACV
ASDTLYSGLLIHISCQFEILEHRLKNIGSDKNYTMKQCVRHHNHIYKYGEMVNDAFQSIMF
FQFCTSLSMICFNFYRIMQIEMDSRYVGTILYMVCSLMQIFYYCWFSNEVKLKSLELSDMI
FRSNWTSLNNNVQRAILLVMRRSMKPIEFTSIYIVSVNLDSFMTLLKSSYSAFSVLQQSRES
>AmelOR72

MHLLRWTFKLFVATGYFLSPKIKSPRKRFLYNVYTVVVTLFLLSFLLTLIMQIVFNVRTADE
LSENFGITITVFTTICKFINLLFRRGIIISLLDLLQKEPFLPMDIEEIKIHTKYNKLIEKVSIFYT
LQNVSCLVALIGATLITDFKKKKLTFEAWIPFNYTASWFLFSLTFIHQCGCAVVTSFGISIFDT
LFAGLLLQVCCQLDTLVYRLQNIKEDAIQSLKYCARQHELIYRFTELMNKLFSSILCLQFLI
SAVAICFSVYRVIYTKTDSQFAGAIIFVFSALIQIFYFCWHGDIAKYKSLEIPDMIFNSNWPN
LSNEAKKILLIIMARSLTPVEVVSAHIIPLNLESFKRLIKATYSAYNMLQQTK
>AmelOR73
MHKLSLSFALLTYGGYWRPTKWPASSYKYHLYNIYSAFMIFLLYFITFCTCVDSLISKNLKT
MSEKFSLCISVFGVSLKVANLFLQRGKIINIMNSLTKENSIPRDEQEEIIQRRNDNYARKVTI
YCEILNESAVFFATVGQYKRFINTRTLPVSDWIPYDLSSTELYIISLLYQTVGLLICANASVG
NETLIAGLMIQAGVQFEIFCHRAQNLPSLVLTVTRNSNVFAETVNTVFQYMIFLQFTISSVV
LCLSIYKFSTVDPLSMNFVWSGFYLCCMLMQVYLYCWFGNEVTLKSNKVSDAIYEMDW
TILPSNVMKDLLLVIARSKKPVKITSGQIFILSTESFMKIMKISYSSFNILKNSTMK
>AmelOR74C
MQGEGYTDVSLKVSQFLLKSAGIWVIGNDAEERQRKFAVFYTLAALIYGIYVNAVDIYHN
LDNLAHCVFLTCNMMCILLGLFKCFVISFFRIEFSRIVSYAQKHFWRLDYDYDEKILFGEC
QKFCRLWIIVVSMISQSSLAFYIITPIYENIGKNKSERILPFKMWVDLPLSVTPYYEIMFVIQL
LAVEQIGIAYVCSDFFLCILNLHALYQFRMMQQELSKIWSAIEQQTTSVAAYTRGCHVALK
KCIRRHQSLIEFCNKLEQVFTFPILSHVVVFSLLMCFDTYEILLADIPTLKRLIFLCHMVASFI
HIIFFTYICHGLMEESGNVGLATYSGWWTTLPMNETGRMLRKDIRIIMMKSMRPCYLSRS
GFFPMSLETSTA
>AmelOR75
MRRRGSKDVSIIWTSFLMKIVGLWLATDRNEQRQRDFALIYTVGTLFISICIAFRDIYYSWG
NFSNSVFICCNILYVAIVLLKISVLYAHREEFFNLIAFTQKNFWRLYDDPQELLIITGCKKLC
NFSIVLIIFCAQGTCAGYMVTPLIENIGKNESDRALPFNLWIDFPVGLSPYFELLFILQILCVY
HVATCYICFDNLLCIVNLHVAGQFRILQHRLKNLGNAIRDETGLPRYEKCCYERLKDCVVQ
HQTLIEYCKRLEDIFTVMVLGQVMFLAVVICLVGFQLFLADTSASKKASLVLNLGGTFFQL
LIFTYSCDNLIRQSVNVGNAVFSGPWVNLPMSKAGILVRKNLIIVIMRSQKICCLTAGKFFP
VSLETSTAVLSTAISYFTLLKQSSLENM
>AmelOR76
MKSKEVRDLSITVTAFYMKIAGFWTSTNYVEERRRNVTMSYTLFAILFAATTEARDLYFS
WGNFSDSIYVACNIITVSLVLIKLLTSFIYNEELLGIIRYAKTNFWHSNYDTCEKSIMNKCQR
TCNYLVFVFTFFAQGTVLGFILRPILVNRGKNESDRILPFNMWLELPLSITPYFEVMFFVQV
VFVYHVCVCYHCFDSLLCILNLHTASQFRILQHRFANTCNEKRGKRDEDEESALSFYEYSK
LKAYIRQHQALIEYCKKLEQVFNSIVFGQVLLFSLLMCLDGYLILMEETPFGRRVTFTFHIT
GCMCQLLMFTYSCDCLIRDSMDIADAAYNCSWSFLPMDKYGKMIRRDLMFVITRSRTPC
CLTACGFFAVSLETYTKVLSTAISIFTILKRYEKEFKSDSS
>AmelOR77C
MNGLLNGGDASMTMTAAFMKLVGLWTAKNRREQRARKFALIYTVAAMLFALWIEFTDF
YYSFGDFSTCLFNTCNIIYITMPLLKIFVIVLNKKDFFHLIFYTEKHFYKDNYDEHEQRIFTN
CRRQCIIFVCFLTFSTKGTLVCYIVSPLVENIGKNQSERALPFNMWVNLPLSTSPYYEIIFTIQ
VLSLYHIGVGYFCFDNLLCVLNLQLAGQFQILQYKMANIVDLLKEKNEKRIINTSYFAKKC
YEAFKKCIREHQALIAYCEKLEKVFSLIILCQVLTFSLIICLDGYQIIL
>AmelOR78

MQSENQLDVSITLSTFFLRNIGLWMSDDPGEQRRMRILLVYTVWILLLGMIINGRDLYFTFL
YNGDILYALTNNVTMVMGLIKIYIILLYKGKFLNLIVHMQQNFWNVNYDYHEKEILDDCR
KTCIFFVSSLTTIGICAMLSYLMTPFAIRSGNNESERMLPFNMWLDMPLSKTPYYEITFLIQ
AMCVYYIGISNFCFDTVFCIMAVHLAGQFRILQYRFTKLCDTDNQICKKNLILEEQMQKFH
EKFKKYVRRHQALIDYHQKLENVYTTIMLSQVLLFSVLICLFGYQVLLATASLARRSIFIFL
LMGAMFLLFMFTFSCNGVMEQSDNVAVGTYSALWTVMPMEKFGRMLRKDLIMVIMRSR
RVCCLTANRFFPISLETYTKILSTAVSYFTLLSKHVDNS
>AmelOR79
MQAEYRLDISINLSTFLLKNVGVWMSHDPGEQRRMRMLLVCTVWMLLLGIVINTRDLYF
TMLYNGDILYVVTNNITLIISLVKICNIIIYKGKFLNLIVDMQENFWNVDYDYHEKEILDDC
KKICIFFISSVTTIGICAIISYLMTPFVAQSGSNESERMLPFNVWITFPVTRTPYYEIIFFIQAICL
YYIGISSFCFDNIFCIMAVHLAGQFRILRYRLTKLCEQEIYEKDSTLTKQMHKFYEQFKECV
RHHQALIDYHQNLENVYTIITLGQVLVFSVLICLFGYQVFVATASFARRSIFVFMLNGSMFL
LFMVTYSCNGVTEHSDNVAIGAYSALWTIVPMDKFGRMLRKDLIMVIKRSRRVCCLTANG
FFPVSLETYTKILSTAVSYFTLLNNRIENANGL
>AmelOR80
MQTESQLDISINLSTFFLKNVGIWMSDNPNEQRRIKMLFLYTIWNLLFGTVVNSRDLYFTL
LYDGDILYVTTNNITMIMGMVKICIILIYKKKFLNLIVYMQQNFWNVNYDHREKQILDDC
RKTCIFFVSCVTIMAICAMICYIMIPFIAQSGSNESERMLPFNMWINLPISRTPYYQITFLIQA
TCVYYVGISYFCFDNIFCIMAVHLAGQFRILRYRFTKLCDMEYGIKENSQSILSKQMHKFY
EKFRKCVQHHQALIDFYQNLENVYTMITFGQVLVFSVLICLFGYQVLVATISFARRFIFVFM
LNGSMFLLFMVTYSCNGVIEHSDNVAVGAYSALWTIMPMDKFGKILRKDLIIVIRRSRRVC
CLTANGFFPVSLETYTKILSTALSYFTLLSNRIENSS
>AmelOR81
MQTESQVDISMNLSTFFLKNVGVWISDNPSEQRWRNMLLGYTTWILLSGIIINGRDLYFTL
LYNGDILYATTNNITMIMGLVKICIILMYKKKFLNLIVYMQQNFWNLNYDHCEKRILDDCR
KTCTFFVSSVTSMAICAMICYLMIPFIVQSGKNESERMLPFNMWINLPVSRTPYYEIIFFIQA
MCVYYVGISTFCFDNIFCIMAVHLAGQFRILRYRFTKLCDVEYENSQSILSKQMQKFYEKF
KKCVQHHQTLIDFYQNLENVYTTITLEQVLVFSVLICLFGYQVLVATASFARRFIFVFLLNG
SIFLLFMVTYSCNGVIEHSDNVAIGAYSALWTIMPMDKFGKIFRKDLMIVIRRSRRVCCLTA
NGFFPVSLETYTKILSTALSYFTLLSNRVENA
>AmelOR82
MQTESQVDISMNLSTFFLKNVGVWMSDNPNEQRQIKMLLINTTWILLSGIVINGRDLYFTL
LYHGDILYSITNNITMIMALIKISIIIIYKGKFLNLIACMQQNFWKVNYDYREKEILNDCRKT
CIFFVSCLTTMVICAMISYLIIPFIAKGNNESERMLPFNMWINLPLSKTPYYEIMFLIQAMCV
YYIGVASFCFDNIFCIMAVHLAGQFRILQYRLTKLYDVECIEMHKKDSILANRVPKFYEKFR
KCVQHHQALIDFYQNLENVYTRIAFGEMLVYSILICLFGYQVLVATASFARRSIFVFLLNGS
TFLLFMVTYSCNGVIEHSDNVAIGAYSALWTIVPMDKFGRMLRKDLIMVITRSRRVCCLTA
NGFFPVSLESYTKILSTALSYFTLLSNRVETANDT
>AmelOR83F
MQTDNQLDISISLSTFFLKNVGVWMPDNSDEQRRMKMLFLYTIWMLFCGTIISTRDLYFTL
LYNGDILYAMTNTITTIMALIKICIILTYKGKFLNLIVYMQQNFWNVDYDCQEKEILDDCRK
TCIFFISSVTTIGMCTVMSYLTTPVITQSGSNESERMFPFNIWINLPITQRTPYYQIIFFVQGVS
VYYIGISYFCFDNIFCIMAVHLAGQFRILRYRLMTLCDTEPETREKDSRSTFAKQVYKFYEQ

FKKCVRYHQALIDYYQNLENVYTIITLGQVLVFSVLICLFGYQVFVAAASTARRFIFVFLLS
GSMFLLFMFTYSCNDVMEHSDNVAIGAYSALWTILPMDKFGRMLRNDLIMVIKRSRRVC
YLTANGFFPVSLETYTKILSTAVSYFTLLNNRVENA
>AmelOR84
MRSTRDISIIWTSFLMKIVGLWLAADRDEQRRRDFALIYTVGALFIIVCIGFRDIYFTWGNF
SDSVYISCNNLYLMIVVLKVGVLYAHKMEFFDLVTFTRNNFWRSYPDPEEELILAECKRIC
TIFVVVISFCAQGTCTGYMITPIIANVGRNESDRELPFNLWVDLPVGLSPYFEILFTVQILCV
YHVGVCYICFDNLLCIVNLHVAGQFRILQHRLRNLNVAVTGDRESYRANVCHAKLRSCVI
RHQTLTKYCKQLENIFTIIVLGQVLFLALVICLVGFQLFLMDTPASRKVSLTLNFAGTLCQL
LMFTYSCDDLIRESVNVGNAVFSGPWAELPMDKVGRVVRKNLIIVVARSHRVCCLTAGKF
FPVSLETSTAVLSTAMSYFTLLR
>AmelOR85
MSSNKVGGDLSITVMTFYMKIVGFWIASNYVEERRRNLTISYTFFAIFFAMATEARDLYFS
WGNFGDSILIICNLVTVILVLFKISISLMYRNKLHKIIQYAKTNFWNLKYDLHDEQIIINTCK
RYSTFFVCIFTFFSQGTVFSFVIRSLKENIGKNETERIHPFNLWLDESWYMTPYFEMVFIIEIL
SLYHVGVCYLYFDNFMCIINLHIAGQFRILQHRFSNVCNEMCEKCCYQLSRKSPYLSICKY
AKLKIYIRQHQTLIEYCRKLEMVSNFIIFGQVLLFSLLICLDGYLILMEDTSNMSRLIFTFHLI
SCMCQLLMFTYSCDCLIRDSTNIANATYNSLWSFMPMDKYGKMLRKDLILVIMRSKSPCY
LTALGFFPVSLETYISILSTAISYFTLLRNRAEQTIMDA
>AmelOR86
MHATPYSDVSIVVSQFLLKLTGVWMTVNDGEKRRRRIAMAYTFVIQVYGLYLNIGDIYHS
WDDLSHCIFLTCNTLCIVLTMFKFSILFIRRTEFKNLILFARKNFWHLDYDRHETILFTKCRK
FCTLWTLTVFSFTQASLTFYIITPICANIGKNKSERILPFKMWVDFPLSETPYYEIMFVIQLLT
VQQIGIAYTCNDNFLCVLNMHVVCQFRILQHRLTKLWSIIDERADKFNYASKCYEALKECI
RQHQSLIEFCDKLEHVYTLPIFGHVVVFSLLMCFDTYEIFLANVPVSMRLIFFFHMVGSFIH
IIFFTYICGGLIEESSNIGLATYSGWWTVLPMDEAGRMLREDVKVMIMKSMRPCHLSAGG
FFPVSLETSTALMSSTLSYFTLMRESSKDK
>AmelOR87
MGAKAVAKVVAHSKLVARHSNKDFALSMTAFLMKIVGLWLAKNEQEQRKRRLTLMYTVI
AILFGVWVQFRDFYYSWPNFGNCAYTACNILCLIMVLLKLFVLFVHRKEFIDLLVYTHENF
WHTNYTNNELLLLQNCKRISMLCITLINVCAQGTIVSYVLTPIVENIGRNHSDRVLPFNMW
VDLPTLFLSPYYEILFVLQVLSLYHVGVCYICFDNLLCLMNLHAATQFRILQHRLSDLGSG
WDTRRSFNKIDRETSWSSCMENCYATFKLCVKQHQDRITYCHRLNDIFTIIVLGHILVFSLL
MCLVGFQVLMANSPPTRRLIFVFHITGSLCQLLLFTYSCDSLIQESTNVGSAVYSGPWICLP
MNRIGRTLRRDLRMVIIRSRKPCCLTASRFFPVSLETCTTVLSTAMSYFTLMRQSFAN
>AmelOR88
MNGRNVRNLSITVTAFYMKVAGFWVANNYAEKRRRNVAMFVTIFFAFMGISIEGRDLYFA
WGDFEDSIFAGCNVITIVLVLLKIFVLYINNEELLNVVNYAKTNFWRESNYEPHEKKIIDDY
RRLCSFLVCSFTFFAQGTVVCFVITPVFVNNGKNESDRIHPFNMWFDRSLSLSPYYEIIYTIQ
VLSAYEIGICYHCFDNLLFVINLYTAGQFRILRYRFENICGKNDDKNYYKVSKSSKYCINEY
KSFKTCVQQHQALIEYCKKLEDVFSIIVLAQVLLFSLLICLDGYLVLMEDTSRAKRVIFTFH
LMGCMCQLLMFTYSCDCLMHDSMSVANAAYNSLWPCLPMDKYGKSLRKDLTFVIMRSR
SPCCLTACGFFPVSLETYTGILSVAVSWFTSLKKYEKKLLQYACIANQSLLKEEESKIIFYHQ
NI

>AmelOR89
MQREVELDVSVNLAAFFLKNVGLWASNDPGHERRRKVILVYTMWCVTLSSVVIIRDVYF
TWFYNGDILYVVTNALSMMMITVKVCVIVVHKEEFINLIVYMQENFWNDNYHDLREREI
LENCKRTCAFFVSLVTAIGICAILSYLATPLIVQTASNNSERMLPFNMWLKLPLSESPYYEL
MFVYQIMTFYFIGISYFCFDNIFCIMTVHLAGQFQILRHRFDRLCNAEDRIAEKGAHAREF
YDRFKARVPYHQALIDYCEKIENVSPTIILEPVMVFSVIICLFGYRILWANAPSTRRSIFIFLLI
GAMSLLFMFTFSCNCVTEHSENIAIGAYSALWTAMPMDKFGKMLRNDLIMVIKRSRRVCC
LTANGFFPVSLETYTTILSTAVSYFTLLRNNVEKANEA
>AmelOR90
MEKELDISVNLSSFFLRSIGLWIGDGSTNERRRKGMLAYTIWCTFFSTIISSRDLYFTWIYNG
DILYALTNYMSVMMILLKICVIVVHKSEFINLILYMQRYFWNVNYDSREKEILNGCKKTCA
FFVSTVTFIGICAILSYLTTPFTARIGNNESERILPFNMWVNLPLSQTPYYELLFLIQIITLYYI
GICYFCFDNVFCIMAIHLTGQFRILGYRFAKLCNIEHEMREKDTVLSKHVHTCYEKFKEYV
RYHQALINFYTKLENVYTMIILGQVIVFSALICLFCYQVLLANAPSARRSIFIFLLIGAMSLL
FMFTYSCDGVIEQSDNVAVGAYSALWTIMPMDKFGRMLRNDLIMVIERSRRVCCLTANGF
FPVSLETYTTILSTAVSYFTLLRNNMENDKDD
>AmelOR91
MSFLESDVSVSLTSIFMKLVGLWMAADQYEQRLRNISVTYNLVAILFALYLQTTDIYYSWG
NFSACLFSVSNTLSLILPLLKIFILLSNKEDFFRLIVYMQRNFLQGNYDDHERKIVFGCKRK
CTFFICFFTFFTMATIVSYIAGPIIGNIGKNESDRVLPFNMWINLPLSMTPYFEITFTLQVLSL
YQIGVSYFCFDNFLCIMNLHLAGQFKVLQYRISTIADRVIEKEEKKEKLIIDSLYFSNKCYTT
FKKYIRQHQALIAYCRKLEVVFNWIVLEQVLMFSLLICLDGYQILMANGDIKTRLTFSFHIL
ACLCQLLMFSYSCDCIIRESVSVATAAYGGPWTLLPMTISGRMMRKDLIIVIMRASIPCCLS
GKGYFIVSLETYTSVLSTAASYFTLLRNNIESDN
>AmelOR92P
MNFLENDVAVSLTSIFMKFVGIWMXQYQQRMRNIMVAYNVIAIFFALWIQTMDMYHSWG
NIRACLFSTSNTLSLILPLLKIFILLCHKQDFFRLVLYMKRNFLXNYDDHERKIVIGCNQKCT
FFICFFTFLTIATTASYMVIPLIVNIGKNESDRVLPFNMWVNLPLSMTPYFEISFVLZVLSLYQ
IAVSYFCFDNFLCIMNFHVAGQFKVLQHRISTIADLTIKTEEKKEKLIIDSLHFSNKCYTTFK
KYIRQHQTLIAYCRKIEVVFNWIVLEQVLMFSLLICLDGYQILMADEDIKTRSIFSFHILSCL
CQLLMFSYSCDCILRESVSVATAAYEGPWTLPPMTISGRMMRKDLIVVIMRASIPCCLSGK
GYFIVSLETYTSVLSTAASYFTLLRNNIESEIMKHD
>AmelOR93P
MNFLESDVSXIFMKLVGIWMAGNGZEXITLFTAIIFSGYNLZMYFILGDFSACLFFISIILSSI
MLLLKIIILFSHREDFFHLILYMKRNFLXNYDDHERKMIGCNZKCTSSSVSSRFSRWRPLLL
TSSVRLLVKNIGKNESDRILPFNMWVNLLLNITSYFEITYTLQXFSLSLYHIGVSYFCFDNFL
CIMNLHVAGQFQVLQYRISNIIYXRFNEKKEKLIVDSXATKCYAIFKKXQHQALIYLIYCRK
LEEVFNLIVLEQVLMFSLLICLDGYQILMADGDVKTRLIFSFHILGFLCQLLMFSYSCDCIIR
WTLXPLLSMTSSRRMIRKDLILVIMRSNVPCYLTGRGFFIVSLEMYXVLSTAAXFTLLKQR
TEATS
>AmelOR94
MTLKYRGDVSFSLATFFLRVVGFWLASSRLEEWFGNATVMYSIITIIFSMWVQMRGLYFS
WGDFGVCTFIVCNSLGLVMDLLKILVVFVHKKKFLGLIAYMQKNFWHLDYDQRENSIIAD
ARQLCVYFVCVFSFFSQSTVFSYMFMPMISNIGKNESDRILIFNMWLDLPLSMSPYFEIIYV

IQALCLYQVGICYLCVDNMFCIMCLHLASQFRILQYRLANVSNVEDEEGVEENMNSSNRC
YAILKNCIRYHQALIQFSITLEEIFTIITLGQVLIFSTLICFVGYQVLLVNMTLSWRISFLCFLIT
NMCQLWMFTYSCDCMTRESVNVASAVYCIPWTRIPMDKFGKMIRKDLQFIVVRSRRACC
LTGCGFFDISLETYTKIMSTAMSYFTILKQRIVEVENT
>AmelOR95
MKNDDFSINLSSIFIKLMGIWMANDQSEKYVRNVTILYSIIALLFGLWLQITDMYYSWGDF
SECIFSMCNMLSIAAPLLKLITLVVHREDFFYLILYLQRKFLHGDYNDYERNIVLNCKRKCT
FFTCSLTFTTLATVVSYVINPLVANIGRNESDRVLPFNIWIDLPLTITPYYEITFVLEVISLYHI
GVSYFCFDNFLCIMNLHVAGQFQVLQYRISNIIDSIDKEKKEKLIMDSCYFASKYYAIFKKC
IRQHQALIAYCRKLEEVFNLIVLEQVLMFSLLICLDGYLVLMADTSTTTRLIFGLHITVCLC
QLLMFTYSCDCIIRESLSVATAANRGPWPMIPMTTSGRMMKKDLILVIMRSGTPCCLTGRG
FFVVSLETYTNVLSTAASYFTLLKQHSEAHS
>AmelOR96
MNLKYRKDLAFTVASFYLRVVGFWLTTSRLEEWFRIGVVGYTILAITFSAWVQIRGLYFN
WGDFSACTYIACDGLGLVMDFFKIFSLFIYEKKFLGLMVYMQKNFWHYNYDEKEDLIVK
DTKRITAYFVCILTFSSLSSIFTYMFRPLLTNIGRNETDRILIFNMYLDLPLSISPYYEIAYTIQI
AALNQAGSCYFCFDNIFCILCLNVACQFRILQYRIANVPILKMKGNPDANKNSSDECYKAF
KNYVQQHQALLDFCETLEEIFTIIVIGQILMFSILFCFLGYQVILADLTPSYRISFISYLFAGM
CQLWMFTYSCDCITQESAKIASAAYASPWINLPMDKFGKMLRQDLQIVVMRSRRACCLTA
CGFFPISLETYTKIMSTTMSYFTLLKQRTVDT
>AmelOR97N
KNGKYKSTVFDISYYKTFKKYLKFLGQYPNQSRWNKEFNTNVMICSLISFLIPGLSRVYISI
VEKNLNALMEIIPIVFATISCAIKLLNHRINKKNFDKLFDLMSKEWEMKNDRNQTCILDEFT
KQGNKFAEIYKNVLLSALLLFLLLPLFPSFLDIVFPLNETRQQFQIFKMKYFVNEDEYFYPI
YFHSVWSSFVIIMITVTIDSLYMLIIHHASGLFAMCGYQIAKATECNDRHNENELFRQCVM
THNKAYKFFEIMNKSSRNSYFLQISLTIIGISIIAVQIVMYLHKPEEAFRISLFLIAAQFHLFIIT
LTGQVIADQSSKLSNNMYCTTWYRMPPNVQKIFHIIQIKSSKPCKLTAGGILELNLENFGIA
LKTCMSYFTIFLSLQD
>AmelOR98
MDMFQKTGKEYSNIFDIPYYKVLKKYLQFLGQDPYQECKYRNIITIIMLISMIAIFIPTTFEI
YVSIHDKNTDAVMECLPNLCASLSSVVKILNVHFNRENFNKLLEFVVKEWDELKLNELHI
LEEITIQGSKIAHLYRNTLLSFLILFLLVPMYFPILDMIDALNQTRSRQQLLRVNYMVFNAD
DYFFYVYLQLAWGAIVIVMIVITVDSLYIIIIHHVCGLFAVCSYEIQKTVKDLTVFTDIEKCS
YKELKNCVIKHKKAIKFYNILNNSSQLSYLLQIGINIMGISTTAFQLAVNLDTRPQEAIRNAV
FCGANQFHLFVLSLPGQILLDHCAELSNTIYCSMWYKLPVKIQKMFNIMLMRSKKSCALT
VYGLYELNMENFGTTFKACISYFTMMLSLK
>AmelOR99
MDIFQKIEKQHNEIYDIPYYKMMEKYIRFLGQDPRQKDEFRNIIVFILIISIASIVIPTTLELYI
SLRNKDVDGVIECIPHFIASSISAVKLLNLHFNRQNYNILFHFVTKKWQQLKSTYELNALD
ETIMQGKKMAQLYRNTLFSFLILFLVVPLVSPILDIVHPLNQTRSRQQLLRVNYIVFDIDDYF
FYVYLQLAWGSIIVVLTIIAADWFYILIIHFNSGLFAVCGVQVLEATMNSNLISKDAFSENSS
YEKFRTCVIMHNEVIEFYNILNENCQYSYLIQVGLNMLGMSTTAVQTVINLDRPDVAIRSA
VFFGADQFHLFLLSLPGQILLDHCADFANAIYDSTWYGTSLEIQKMLYMMQIRSKKLCALT
AGGLYDMNIENFGITFKTCMSYFTMIMSFK

>AmelOR100P
VHIFQIYEEQCLDIFEIPYYKSLKKWLILSGLYPPKNIIIILVAISIISVTLPLMFAIYTSLHAKNI
DAMFECLPSLGVCIVAMFKLQNIYNNSENFKKLFTFVAKQWYQLKLNNEIRILEEIIMQGN
KMAQIYKNTLLLSMTIFFFVPLIFPILDIVYPLNETRPRQQLYRVNYFIFNHEDYFFYVYFQL
VWSSFVCVIVIIIFDWLYILIIHHNSGMFAVCGYQIQKIFAEKVFSNIHIYEQFKNCLIVHSEAI
QFFSILDESSRNTYLFLVGTNIMATSISAVQVVLNLDKLEVAIKSAVFLIAAQFHLFILSIPGQI
LLNHYSNLKNNIFMSSWYNMPIEVQKMFYVMQIRCKKPCSLTACGLYEMNMENFGTALK
TCMSYITMILSLK
>AmelOR101
MDIFQKTKKQHNEIYDIPYYKMMEKYIRFLGQDPRQKNEFRNIIVFILVISIASILIPTTLELY
ISLRNKDMDGVIECIPHFIASSISAVKLLNLHFNRQNYNILFHFVIKKWQQLKSIYELNALD
ETIMQGKRMAKLYRNTLFSFLILFLLVPLVSPILDIVHPLNQTRSRQQLLRVNYIIFDTDDYF
FYIYLQLAWGSIIVVLTIIAADWFYILIIHFNSGLFAVCGVQVLEATMNSNLVSKDAFSENSS
YEKFRTCVIMHNEVIEFYNILNENCQYSYLIQVGLNMLGMSTTAVQTVINLDRPDVAIRSA
VFFGANQFHLFLLSLPGQILLDHCADFANAIYDTTWYGTSLEIQKMLYMMQIRSKKLCALT
AGGLYDMNIENFGITFKTCMSYFTMIMSLK
>AmelOR102
MNIFQKTRRQCPDIFDIPYYKMVEKYFQLLGQDPRLKNEFRNFIVTVVVISISGNIVPTSIEL
YTSLCDKNMDAVIEGLPHFIAATISAVKILNVYFYRENFDKLFQFVTNEWNKLKLNNELHI
LDKTIIRGNRTAHLYRSALLIALVLFLLIPLISPMLDVFLPLNETRPRQQLLKVNYLVFNDDD
YFFYVYLQLAWGSIIVVVTSVAVDSLLILIIHHCSGLFTVCGYQVQKVISNAKSFNGTVLNN
YTYEQIKNCVIMHDEAIQFYNILNESNRNSYLIQVGLNMLAISATAVQAVVNLDRPEEAIRS
AVFCGANQFHLFVLSLPGQVLLDHCSEFSNNIYSCIWYRAPVRIQKVLYIMQIRSKKLCTLS
AGGLYEMNIENFGITFKTCMSYFTMIMSLK
>AmelOR103
MDIFQKTERQYPEIFDIPYYKMVEKYFQLLGQDPRLKNEFRNFIVTVVVISISGNIVPTSIEL
YTSLCDKNMDAVIEGLPHFIAATISAVKILNIYFYRENFDKLFQFVASEWDKLKLNNELHIL
DNTIIQGNKMAQLYRSALLTALILFLLIPLLSPILDIVLPLNETRPRQQLLKVNYLFFNDDNY
FFYVYLQLAWGSIMVVVTIVAVDSLLILIIHHCSGLFTVCGYQVQQVTGNAKSLNKIVSNN
YTYEQIRNCVITHDEAIQFYNILNESNRNSYLIQVGLNMLAISATAVQAVVNLDRPEEAIRS
AVFCGANQFHLFVLSLPGQVLLDHCSEFSNNIYNCIWYRVPVRIQKVLYVMQIRSKKLCTL
SAGGLYEMNIENFGTTFKTCMSYFTMIMSLK
>AmelOR104
MDVFQKTRNKCINIFDIPYYKLLEKYMKFLGQDPRQRDGFRNIIVIVMVASISGILIPTSLEL
YTSLRDKNMDAVIECLPHLIAAATSVVKLLNIHFNRENFKKLFEFITKEWEKFELNNQFHV
LEEITIKGSKMAQLYRNTLLSFMVLFLLVPLIFPFLDIVHPLNETRPRQQLFRVNYLIFNHND
YFFYIYLQLAWGSIIVVMIIVTVDSLYMIIIHHSSGMFAMCGYKVQEATKYQNLFNDRIISE
NYTYEQLKNCITIHNKALQFYNILNESSRNSYLIQVGLNMMGISVTAVQTVVNLDRPEEAI
RTAVFLGAEQFHLFVISLPGQVLLDHCTELANNIYSSTWYRIPVKIQKVLHMMQIRSKKPC
SLTAGGLYEMNMENFGITFKTCMSYFTMLMSLKK
>AmelOR105
MSMLQKSNEQEYNAFDIAYYKTLKLYLTICGINPYQNNSISIIIIIMIISVCMSFLCPTSIQLWE
AISNKDFDNIIQNIPQVITVIASMIKILNIYSNKMQFKNLFYSLAQDWKLLESKEELIMLDKF
TQYGSKLALLYRRTLLTFLVIFLFLPLCNPILDVILPLNETRSRQNIFNVNYIILDNYEYFYIV

YMHLSCSAVIIVIIIISVDSLYISIIYHACGLFAACGYQIQKLTKVHTIEKNGPNISNIDYEEFK
QCVIMHYKCLQLYDVLEKCCRNLYLIQMGLNIMIISVTCVEVVVFLDRPKEAIRAIIYVIAQ
QFHLYAISLPGETLLNQSSKLADKIYDSEWYKIPMKVQKVLHIMQIRSNKPCILTAAGLYE
MKIESFGITIKTCMSYFMMFLSLRE
>AmelOR106
MDVRLEERYLKINKIYSIIVGMWPNQKRKTIPRIFVELIAILAHLTQGGNMVLFFSLTLAMD
QIPFLIAAILLMIKYNNFIINEQKFKELFVSILNDWQKKKTHEEEMILEKYADKSLFFILIYV
VNAYFCTVLFLILPLTPILLDIFIPLNESRPRVQMYPAYYYIENEADYYYPILIFSIVSLLTAMC
VYIATDTTLVYYVVQHACGLLTLAGYRFRNSLNDLYSMRKDSKMDEKIYRRMCYAIKTHK
RALAYLTKIEDFYSMNIFAQVGASILCLTVTLMKIATIKWSMETNQYYGFVIAQVVHIFFLT
AQGQFVIDSHDNVYRDMYEPYWYNVQYKIQAMFVLILRRNLNPPLLTAGGLMQLNLNTF
AQVVKTSVSYFTVLKSV
>AmelOR107
MDQQAMEELYLKDNKFFGQLVGVWPDQGKFMKFLMRFIILIVMIIAFIAQISRVAVFYSVD
VLSDQIPYIDLGFALMLKQYNYILNEKKLRELLHNIISDRLVKRSKEEEEIFEIYFKRAMFFC
SFYEVSIYSCGFMFLSMPSIPLIMNVIMPLNESRSRELVYPSYYFVDEQKYYYLITGHMLAV
CLGHVFVYIACDINLIHVVHHGCALLTISGYHFKHAMDNVDLCNEKYSDELMEKTYAKV
SQSIDAHKKAVEYVNKIDACHIHYFFILLGMIIVTFTGTFIKLTSMEIGGRFFTFCTFTIGQLT
HLLFLMVMGQFLIDSNEEVFKTIYDARWYYGSSKTQSLYLLVLRKCLNPPKLTGGGLIALN
LDSFVKVLKTSFSYYTVFRSS
>AmelOR108
MNKQAIEDQYLRINKFFGQLVGVWPYQERFTKFCIRLTIFAIIILTLTTQIYQVIVFCTLDALS
NQLPYLNALFILLFKQYNYILNEDKLRDLLNDIIFDRLMVRSKKELEILNMYSRRATTLCIF
YEVIVIFSAIMFIMIPTIPPILNIIMPLNESRDREFIYPTYFFIDEEKYYYPILTYMATVILIVSSV
YLACDTNLVQIVHHGCALLAISGYHFKHAVDDMKFSNGNYIDLLMDETYKKVKQSIKAH
KTAVEYVDKIDACHIYYFLLIIGMIVLAFTGTFLKLSTMEIEIRFFTFCGYTVAQLTHLFFLTI
MGQFLINANDEIFNTIYEAHWYNGSSRTQSLYVLVLRKCLNPPTLTGGGLIVLNLDSFVQIL
KLSFSYYTVFRS
>AmelOR109
MDERAIEDQYLKINKFFGQLVGVWPYQKKFFKTCIRFITFTIMIFSLATQISRVIVFYSLDVL
SDQLPYINAGIVTLFKQYNYILNEDKLRELLHDIVSDRLIERSKEELEILEMYSRRTTALCAL
YKVMVYSCAFMFLVIPTIPPILNIVAPLNVSRSREFIYPTYYFVDEQKYYYPILTHMIAVILVL
SSVYLACDTNLVQIVHHGCALLAISGYHFKHAVDDVKFCDGYYIDASMDETYVKIRQSIK
AHKTAVQYVDKIDACHIHYFLLVIGMIVLAFTGTFLKLSTMEVGIRFFTFCAYTIAQLIHLFF
LTIMGQFLINANEETFKTIYEADWYNGSSKMQSLYVLVLRKCLSPPKLTGGGFVALNLDSF
VQILKASFSYYTVFRS
>AmelOR110F
MDKQTIENQYLKINKFFGQLVGVWPFQERFIKTCMRFIVSVIMLLDLATQISRVIVFYSFDV
FSDQIPYLNAAIICLFKEYNYVLNENKLRELLNDIISDRLIRRSKKELEILELYSRKATTLCIL
YKVMVYSCAFMFLVIPTIPPILNIVAPLNVSRSREFIYPTYYFVDEQKYYYPILMHMIAAILV
LSSIYLACDTNLVQVVHHGCALLAISGYHFKHAVDDVKICDENYITLMDETYTRVRHSIKA
HRTAVEYVDKIDACHIYYFLLTIGMIVLTFTGTFVKLSTMEMGIRFFTFCAYIAAQLTHLFFL
TIMGQFLINANEEIFRTIYEARWYNGSSKTQSLYVLVLRKCLTFPKLTGGGLIILNLNSFVQI
LKASFSYYTVFRS

>AmelOR111
MDKRVIEDQYLKINKFFGQLVGVWPYQQRFIKFCIRFITSVIVVLTLAAQISRVIIFYSIDVLS
DQLPYLDVGFVLLFKQYNYILNEDKLRELLNEIISDRLIKRSKEELEILEIYLKRARVLSTVY
EVSIFFCGFMFLLIPSIPPILNIISPLNESRGRELIYPSYYFVDEEKYYYPILMHMIAVALILTSV
YVACDTYLVYIVHHGCALLAISGYRFKHAVDDIKLCGGDCIDPLTDETYTKVRQSIKAHK
MAVEYVDKIDACHIHYFLLIIGMIVLAFTGTFVKLSSMEVNVRFFTFCAFTVGQLTHLFFLT
IMGQFLINANEEIFKTIYEARWYNGSSRTQSLYILVLRKCLSPPKLTGGGLVALNLDSFLQIL
KASFSYYTVFRS
>AmelOR112
MDARTVEKNFLKVNKIFGLITGVWPYQNYRSKMAERFISVTVMMSGFVTQFAYLVLNPT
MDKIATNLPYSIASFGTFVKMGNYFLDETKLTTILNHIFEDWATIKSKEEYEIMYKYSRRGL
FITISYFLHIGVTETFMLILPMVPPILDIIVPLNVSRKRVFLYPAYFWLDDEKYYVLLLGHMII
TLLMICFIFCACDTNYVYAVQHACGLLAIAKYRFKNVCKNLKEDHAIPLEEIKYKSICESIK
AHQHALKYLKLIENSYHTYLFVSMGLLIMAISVSLLEVANGKNGSRELVQATFLFAQLFHT
FILTVQGQFVINELQDVYESIYESPWYTFSPRIRSLYVLSLRSCLNFPTLTAGGLIVLNLQSFA
EIIKAAVSYYTVMQTT
>AmelOR113
MDSDILEKRFLKITKRFAKLSGIWPDQNKYLKYISWIIIYVISIPSIVVQIARIVHISTANVIVE
QSGIATAIFLSLLKEANYILNATKVKSLFNDMYMDWRMDRPKKEFEIMSTYAQRGSFLAM
FYFINAYCCSLLFLQVPWTARLLYMIKSQNTSPPMLYVIPGYYFVDDDRDYYYFIQLHMSL
SIIMVANVYVAYDTCYMVFVQHVCGLLAVAGYRFKHAIDDSASKNSEEKIKETCKKIRSSI
QGHEGAIRYLKKIEDTHVNLLFISLGLIIMCFSITLLKVVTMDYCLDFYKYSSFLIVQLMHL
CYVMIQGQFVIDSCNEIYYSIYEASWYNINPKIQALYILALRRSLTPPRLTAGGLIELNMQSF
SEVIKLSISYYTVLRST
>AmelOR114
MKFIGIWPEERKWNQASNYLVLIPFLMILCFICAPQTINLTIISNDFNLVIENLSMGNITITLS
LLKTIAFWINGKPLKSLLNCMANDWIKVTSKTEQETMARIASITRNTIIKSTVMCHTVVAF
YVFLRYISMKYNENKLLFRAYFPYDTTVSPNYELTILGQFVAALYAATSYTAVDTFVAMLIL
HVCGQLSGIKNELSRLPTYDKKDLKRRLKEIVQKHEYVNRFAETIENCFNVMLLIQILGCT
VQLCFQCFQAIMSFGGGEAQEYLFFQLMFLLVYVFYVMLQLYLYCYVGERLSVESMEIVN
AAYNTEWYTLPTNITKMLIIVMCRAKSPLTVTAGRFCSFTLQLFSEVLKTSMRYLSVLYAV
KDKIKR
>AmelOR115
MDFAMGWNRFNLTLLGVYPEPRKMSRNSRLMSSLIFWFTTLVTFTFICAPQTANLILKSTS
LDEVIENLSINIPIVFALIKQIVLRYYKKALTELLGEMLADWSGPIGDQDRETMLRNARLSR
AISIVCSTLTYFMLLAFVSLQVWSNAENASETDLGGLLHPATFPYETSKSPNYEITWLGQL
MGTVLTAICYSCFDTFLAVLVLHLCGQLTVLGTALEDLVNATRRNDYKTFEQRLSSIVNRH
NHLSRFAVIVEDCFNITLLVQTLICTAMFCLTGYRMITSVDREDEADVPIVGIIFFIIHVIYTM
LHLFIYCYVGETLLGQSTGIGLSTYHCNWYDLPSRRAVLLMIVIRRANVSFQITAGKFSPFS
LEFFNAVLKTSAGYLSVLLAMKDRLVEGK
>AmelOR116
MTNHLEKQIKLKKINSNKHLQNNLSIIYYIGLWPDRVKYKYLYNLYTICSLIFLVGIIIVSEII
YIIINWGKIEIMMTGLTILMTNSTYAAKVIYIICRYERIKNLVDITNSEIFNRDNDKYKHIISY
YNWQGIFHHIAYQGFASICIFSYSCIPLQSAFSGKSKQLPIAGWYPYNVTSTPIFEIACLHQV

LVILINCINNIAIDTLITGFIIITCCQLTILKKCIARNNNINIEKSPSKIYNKFYENLKHCVKHSII
IFDFTKQIQDIFGIIIFFQLFVNCIIVCLAAFNLSQIKNYITPEFFGSLLYICCMIYQIFIYCWHG
NELYLHSMKICLSAYKNNWWNNNKNFNYALLIIMIRTQIPLIIIVGKVMELSLQNFLLILRTS
YSIFTLLKTFTT
>AmelOR117
MKKPFNKSIDYYILPNKIFCSIAGMWPIDEKSSIFSKIFAYVRLIFGLIIVNSFFIPQIIIIVMNW
KNIKIIAGIGCVLTTITQVLFKMIYLIARREKTYSLYYKIRNLWNSSNDSKERPYEEFAYWAR
IFSIIFYSSCMCNVFTFSIAAAIDYFKFEYNANNTENNRHLPFIVWYGTDISASPSFEIVFFYQ
IISSSICASVISGLDTSLMTIILHVSGQFKLINIWINNIGIEINCNPNYMRKLKVDLIKCIRHHQ
QLIHVVNNVNNLFTPIIFIQLLTSGIEICLSGYAVLDNNSANADLLKFISYFISMGIQLLLWC
WPGEILIQESQEIGHVIYLNIPWYNLPPIYQKYLYFMIVRSQQYCRITALTFQTLSICTLSNVF
NTSVSYFTLLRQMQQ
>AmelOR118
MINRPLEYSLRIFGIWPDSPYPKLKIITWIIILPTFLVFQYWYCITHIKLGLIDLLDGLSLTLSN
TLVFIKLIVIWFHKRTFYEILMSMKEDLNNNKHSATENKRIIMDKSMLSSRISNFLISYFAITF
FLYSGVALVIFDEDQGKFLVRMEFPFIATISPRYEIILITQFIFESFIVYGAATSIALIAALILYVG
SQIDLFCQNLTFHSYKKRESQDTIKDIIVRHQKIIQLSKNIETIFTYISLCQFVSNMLVICFISF
VLTVSLHTEQTIVLIMKCLPYYIAVNCEAFILCYTGEYITSKSENINKAVYNFLWYNLKPRD
VRIMLMIILRSQKQLTLTAGKFICLSLEAFANMLKASASYVSVLYARY
>AmelOR119
MHTQRDTSEITYSHDTATSRKLFYLLVVVGGQMAHASGHEWMKSIRTLTSIKINYLKYSG
LGEIDSSCSRILKYAYFVYKVWMLVSMCILAITVFADIYTNMDNLSITTDDGCIFAGIFVVIF
KAMNLQIQLESVKKIIDKYHTRNKVMFFGFCVIGACLGFALLCFTPMENGLPIRAKYPLNT
TVSPWHEISFFVETCAVSGGLLGIIVMDSMTTFKCSLITMLLDALSVNFENCNGETKRTICN
RHGKEERNDNNNRFLDRYKKCVQFHQRLVVISRDYNKIYSLSMLVQMISSTSIICLTGFQA
VVVGGQSSNIMKYGIYLSAAMSQLFYICWLGNELGYASSTLDKNQWFSGWCNERLTGIG
QVFTLSTVFTRKSIILRASVFYVLSLETFIAIIKRSYSFFTLLNNMDLTDH
>AmelOR120
MSNQTNTMNIRNYIFINQLVLKFVGFYPINILRYVICISCIMFIVIPQIIMIYINWNDLNIVME
TGSTLLTILLAALKSIVWIFNRKKLEFFIEFMLTDYWKIIETNVFEYLQEYAIYAKNITKGYF
FSMCNALLFFFSLPIIETLTKNENLNNFTIKNFPFAASYPITFYKFPFYEIAYISQILATSICCLM
MLAIDSLIATALLHTCGHFTVLKENLKNLDTYIYDLTKTNLKTNSKYINKNLYEIKTQIIYII
KHHQLVLWFCDNMEKNFHLILFLQAITSSLIICFVGFQISIALTERSKFLESFSHLIVSLFQLL
LFCFPGDILIRQSFNISIAAYSMQWYQLPTFIKDEICMIILRSQRPSFITAGKLYIMHLENFTAI
LSTAFSYFMMLQSFNTEA
>AmelOR121
MHTSESKKYSKDYEWAVRLNRFSLNVICLWPVEEQNMRKQSWTKLHIMTCFMLITFVCTI
PCLCALKQCNNLMEVTDNLAYSIPLIITTIKFIVVSSKKKVLSLIVNMVAKDWAKLKTDHE
KDIMIRRARIARIINIFGYILICILIWLLMILPRFGITIRYVTNETDAKKLFPLPSYYIFDVSETP
YFEIMYALQSISLLIAAFCYAGVDNFFGILILHICGQLTNLRFQLANIKESEASNFILIAIVKD
HIRLIRVIELLKMFVEQIINLIITIFKYFFKCLKMYIEEEQFSLFRIIYLICNFTNTFLQTFLYFM
AGQMLVTQSEEVHNAAYECEWVSLKYTKAKSLIIIMARSKKPLYLTAGKLFPVTMLTFCNI
LKISLSYISFLLTIL
>AmelOR122

MNMDVFDKQYRIYRIILKIVGLWPYDKSIYVWIQRICLSMYFLIGVIFQIILLVKSEITLRNYI
VTLSAIFPLLLFFIRYIYYITMFPYVEILFDNIRTEENLLQDTTEIQIQTKYLDISSHIIYIFCCM
TFAFIVAAIIFLVNPVILDLRNPLNESRIFYFDLLFLLDDQSAYIKIFLILNFMLNILFGLLSITS
TESFTNIFSYYICRQFNIVNYRIRKIIEDLSTRNLSKIDLKIKDIHRVVDIHCHAIELLYKALIT
MDNRIEIFGSTLIVIYHLMIAFYNNHCGQLIIDSNLGIFNELFASTWYRIPLKAQKLLLFMIL
RSSMGCEICLSGLFTPSYAGLTSMMSSSFSYCTVIYSIQ
>AmelOR123
MNVFDNQYRTYRIILKIVGLWPYDNSIYVRIQRICVLIYFLIGILIQIFSLVKSEISLRNCIVTF
STTFPIVLFCLRYIYCLTLFSYAKVLFDDICIEEHLLQDTTEIQIQTKYLDISSHIIYIFCWLSFI
CVASTWIFILNPVILDVIMPLNKFRLHYSVIFLSNDRRKFIDIFLVLNSIIIFTFGLLSLICSELF
TNIVSYYICRQFHIVCYRIRKIITDLSMPNLPKTDLKLRDIHRVVDIHCHAIELLYKALVTTD
NRMEILGSTLIVIYHVMMALYNNHYGQLIINSNHGIFNELCASTWYRIPLKAQKLLLFMIL
RSSMGCEICLSGLFTPSYAGLTSMMSSSFSYCAVIYSIQ
>AmelOR124C
MNVFDNQYRTYRIILKIVGLWPYDNSIYVRIQRICVLIYFLIGVLVQIFSFVKSEISLRNCIVT
FSTTFPTLLFCLRYIYCLTLFSYAKLLFDDICTEEHLLQDTTEIQIQTKYLDISSHIIYIFC
>AmelOR125
MNMDVFDKQYRIYRIILKIVGLWPYDKSIYVWIQRICLSMYFLIGVIFQIIVLVKSEITLRNY
IVTLSAIFPLLLFFIRYIYYITMFPYAKLLFDDIRTEEYLLEDETEIQIQTRYLDISSHIIYIFCCM
TFAFIAAAIIFLVNLIILDLRNSLNEFRFYFDLLFFFDDQSAYIKIFLILNFMLNTLFGLLSITST
ESLTNIFSYYVCRQFNIVNYRIRKIIEDLSTPNLSKIDLKIKDIHRVVDIHCHAIELLYKALTA
MDDRMEILGSTLIVIYHLMIAFYNNHCGQLIIDSNLGIFNELFASTWYRIPLKAQKLLLFMI
LRSSMDCELRLSGLFTPSYAGLTSMMSSSFSYCTVIYSIQ
>AmelOR126
MNVFDNQYRTYRIILKIVGLWPYDNSIYVRIQRICVLIYFLIGVLVQIFSFVKSEISLRNCIVT
FSMTFPTVLFCLRYIYCLTLFSYAKLLFDDICTEEHLLQDTTEIQIQTKYLDISSHIIYIFCWLS
FICAAASCILILNPVILDVIMPLNKFRLHYSLIFLSNDRRKCIDIFLVLNSIIIFTFGLLSLICSEL
LTNIFSYYICRQFHIVCYRIRKIITDLSTPNLPKTDLKLRDIHRVVDIHCHAIELLYKALITMD
NRMEILGSILIVIYHLMMALYNNHYGQLIINSNHGIFNELCASTWYRIPLKAQKLLLFMILR
SSMGCEICLSGLFTPSYAGLTSMMSSSFSYCAVIYSIQ
>AmelOR127
MNVFDNQYRTYRIILKIVGLWPYDNSIYVRIQRICVLIYFLIGILIQIFSFVKSEISLRNCIVTF
STTFPTLLFCLRYIYCLTLFSYAKLLFDDICTEEHLLQDTTEIQIQTKYLDISSHIIYIFCWLSFI
CAAASCIFIVNPVILDVIMPLNKFRLHYSVIFLSNDRRKCIDIFLVLNSIIIFIFGLLSLICSELF
TNIVSYYICRQFHIVSYRIRKIITNLSMSNLPQIDLKLRDIHRVVDIHCHAIELLYNALITMDN
RVEIFCSTIVVTYHLMTALYNNHYGQLIINSNHDIFNELCASTWYRIPLKAQKLLLFMILRS
SMGCEICLSGLFTPSYAGLTSMMSSSFSYCAVIYSIQ
>AmelOR128
MNVFDNQYRTYRTVLKIVGLWPYDNSIYVRIQRICVLIYFLIVVLVQIFSLVKSEISLRNCIV
TFSTTFPTLLFCLRYIYCLTLFSYAELLFDNVHTEEHLLEDTTEIQIQTKYLDISSHIIDIFCW
MSFICVASTCIFMLNPVILDVIMPLNKFRLHFSLIFLSNDRRTYIDIFMVLNLIIILIFGLLSIVC
SESLTNIFSYYIYRQFDIVSYRIQKIIADLSMPNLPKTDLKFRDIHRVVDIHCHAIELLYKALI
TMDNRMEIFGCILVVAYHLMIAFYSNYCGQLIIDSNLGIFNELYASTWYRIPLKAQKLLLLM
MLRSTVGCELHLSGLFTPSYAGFTSMMSSSFSYCAVIYSIQ

>AmelOR129F
MNVFDNQYRTYRIILKIIGLWPYDNSIYVWIYRLCLLIYFLVVVLVQIFSLAKSEISLRNCIV
TLSTTFPTLLYCLRYIYCLTLFSYTELLFDNIRTEEHILQDMTEIQIQTKYLDISSHIIDIFCWL
SFICVAATWIFILNPVTLDVIMPLNKSRIHFSLIFLSNDRRTYIDIFMVLNLIIILIFGLLSLICSE
SLTNIFSYYVCRQFDIVSYRMRKIIVNLSMPNLPKTDLKLRDIHRVVDIHCRTIELLYNALII
MNNRMEIFGSALMVMYHLMIAFYNNHCGQLIIDSNFGIFKELYASTWYRIPLKAQKLLLF
MMFKSSVGCELRLCGLFTASYAGFTSMMSSSFSYCAVIYSIQ
>AmelOR130
MNVFDNQYRIYRIILKIIGLWPYDNSIYVWIQRLCLLSYFFANIIFQIVSLLRSEITLQNSILIL
SITCPLVLFLLRYIGSIACFPTIKIVFKHIRTEENIVQDSIESQIRMKLIDDSHHIINIFFWMTYT
TIVIFIIYVSYPIILDFMIPLNESRTHFIYYITTFSHNQSIYLDILDFNFMFTGIFGLLSVACSESI
TGIYSYYICILLKIVSYRIQKIIMYLAMFKLSPKQIDSKLIELYRVVDIHNQTIELLVNATLIKK
NQLEMLFCFTLVAIHLVIIFLNNYNGQIVMNSSQELFDELYNSMWYFMPLKAQKILLLIML
QSTTKHAFNILGLFTPCYAGFSTMLSSSFSYFTLMYSIQ
>AmelOR131
MDVFDKYYHTYRIVLKIIGLWPYNNSVYVWIQRLCISALFLGNIIFQILSLIRSEITLRNCILI
LSTTCPLIIILLRYISFIIFFPMVKLLFHHICVEENAVQDLIEIQIRMKYIGNSRHMIEILLRVTF
LTITLFSIFLLYFVTMDFIMPLNEFHRHILLYVTLFSVNRTIYFYILYLNFLFVITFGLLSLICTE
SIVGLYSYHTGMLFKIISYRIRKIITYLTMFNVSSKQIDSKLAELHRVVDIHNQAIGLVVNAI
TIKKDQLEILITLIIFANHLMIMFLCNYNGQILINSNEEFFHELYIPVWYFVPLKVQKILLLIMI
RSSMACIFHIFGVFIPCYVGFTTMLSTSFSYFTLIYSIQ
>AmelOR132
MDVFDKYYHSYRTVLKIIGLWPYNNSIYVWIQRLLLLTLFLGNIIFQIMSLLRSEITLRNCILI
LSTTCPLIIISLRYICFILFFPMIKYLFHHMRMEENIVQDSIETRIRTKCINDSCHMIDIFLWMI
YAIFAFCIILLLCPIILDFIMPLNESRIYIAHYITIFSDKRIIYVDILCLNYMFLMIFVVLSIMSTE
SILGLYSYHTSMLFKIISYRIQKIITYLTIVNLSSKQIDSKLAELYHVVDIHNQAIQLLENAIIV
TKDHLEILICLMLFVKQLMIMFLCNYNGQILIDNSEELFDELYFSIWYFVPLKVQKILLLIM
TRSSTTCMFHILGVFVPCYTGFTTMLSTSFSYFTLMYSIQ
>AmelOR133N
MEENIIQDSIEAQIRTKYISDSRHMIEILLWMAYATITLYSILGLCPIIFIILLNESPIRMLHYVT
LLSVNGTIYFYILCLDFLFIIIFGLLSMICTETIVGIYIYHTSILFKIISHRIQKIIAYLNMFNLLS
NQIESKLAELYCVVDIHNQAIQLLVNAITIKKDQLEILISLIIFVNHLVIMFLCNHTAQILINN
NEEFFHELYISVWYSVPLKVQKILLLIMIRSSMACIFHICGVFVPCHAGFTTMLSTSFSYFTL
MYSIQ
>AmelOR134
MDIFDKHYYSYRTVLKIIGLWPYNNSIYVWIQRLWISALFLGNIIFQIVLLLRSKITVRNCILI
LSTTCPLIIISLRYICFILFFPMIKYLFHHMRMEENIIQDSIEAQIRMKYIGDSRHMIEIFLWMA
YANITLYSILGLYLIIFIMPLNESPIRMLHYVTLFSVNGTIYFYILCLDFLFVIIFGFLSIICTETII
GIYIYHTGVLFKIISHRIQKIITYLTIIDLSSKQIDSKLAELYRVVDIHNQAIQLLVNAITIKKD
QLEILISLIIFTNQLVFIFLCNHTAQILINNSEEFFYELYISVWYFVPLKIQKILLLIMIRSSTAC
MFHIFGVFVSCHAGFTTMLSTSFSYFTLMYSIQ
>AmelOR135
MDVFDKQYHSYRTVMKIVGLWPYNNSIYIWIQRLLLLTFFLGNVIFQIVSLLKSEITLRNCI
LILSITCPFIIVSLRYVCFIVFFPTIKLLFHHMRVEENIVQDLIEIQIRTKYINDSCHIIDIFFWVA

CTNITLSSISLLYFITLNFIMPLNEFRIIHYITLFSVNRTMYFNILCLDFIFVVIFALLSVICTESII
GLYSYHISVLFKIINHRIQKIITYLTIVNLSSKQIETKLAELYRVVDMHNQAIELLVNAIIIKKD
QLEISISFIFFVNQLIIMFLCNHSGQILIDNSQKLFNELYISIWYFVPLKVQKILLLIMIRSSTRC
MFHILDIFTPCYAGFSKMLSTSFSYFTLIYSMQ
>AmelOR136
MNVFDKHYHTYRTLMKIVGLWPYNNSIYVWIQRLWFLMFFFGNIIFQIMSLLTSAITLQNC
VLIFSTTCPLIIVLFRYIGLILFFPTIKLLFHHMCMEEAMIQDSIEAQIRRKYIDDSCYMIDIFF
WMTYVGIALCSILLLCPITLDFIMPLNESRTRIVHYVTIFSDKSIIYMDILCLNYMLLAILVIL
SATCTESILGLYSYHTSIMFKIIGHRIQKIVKYLTMFNLSSKQIDSKLAELYRIVDIHNQAIEL
LLNAIVIKKDELEILISFIFFTTQLVITFLNNNCNQILIDNSQELFIELYISMWYFVPLKVQKIL
LLIMIRSSTACMINILGVFTPCYIGFSKMLSTSFSYFTLMHSIQ
>AmelOR137C
MDIFDKRYCTYRTMLKIVGLWPYNNSIYVWIQRLWLLIFFLGNIIFQVVSLSSEITLRNCILI
LSLIFPLTIILVRYVSCVIFFSMIKLLFHHMRMGGNIIQDSTEIKIRKKYINDSCHMMNIFFWII
YGIAALSIIFILYPMTLDFIMPLNRTRIRIIHYITIFPYNRTMYLDILSLNFMFVGIFGSLSLACT
ESIFGLYCFHASILFKIIIYRIQKIVTYLTMFNLSSKQIDTKLTELYRAVDIHNQTIGL
>AmelOR138N
VVSLSSEITLRNCILILSLIFPLTIILVRYISCIIFFSMIKLLFHHMRMERNMIQDSTEIKIRKKYI
NDSCHMINIFFZIIYGIAVLSIIFILYPMTLDFIMPLNKTRIHIIHYITIFPYNRTIYLDILSLNFM
FVGIFGSLSLACTESIFGLYCFHANILFKIISYRIQKIVTYLTMFNLSSKQIDMKLTELYRAVDI
HNQAIGLLVNAIIVKKDQLEMLISFMILMAQLIITFLCNYNNQILIDNSQELLDELYISAWYF
VPLKVQKILLLIMIRSSTSCTFHILGVFIPCYTGFSKILSTSFSYFTMIYSIQ
>AmelOR139P
MDIFDKHQSYHSYRTIMKIIGLWPYNNSIYVYIQKLZLLIFFLGQIIFQXDAVIAFFSISLLYPI
ILDFINSLNESRTRIIHYFTIFFHSRIIYIDILCLNYIFLAIISLLSIICIESMIGLYTIVTTSLFFKIIG
YRIQKIITXLTIFNLSSKQINSKLVELYHVVDFHNQVIELLVNMILIRKDQLEIFMFFIFLVSQ
MMIMFICNYSSQILIDNSQELLYDLYISMWYFVPLKVZKILLLIMIQSSITYMISILGVFILCHI
GFSTMLNTSFSYFTLIXSTQ
>AmelOR140N
LRGLYSVWGVNYDAVIECMPPIISIFQSASMYFNGIFNTKKIKNILLFIKNDHKYYINRPENII
LQKYDLQGKKITFYYILYVYTTLFVYLLLPTIPLIIDFITSSNHSQKRNFLFELDYGMDKQQ
YFYYISIHSYIGTAIVANLIASCDTMYMLYAQHAYALFAIVSYELKTIHILNTNNLINVTDHH
LLEKYKNITLLSKDEKKVYRKLFICIKNHQNAIKYSNLLESLFTKSILVQLFFNVLCLSITGV
ETVIKLGNLSEMMRFGSFTFAQAVHIFFLCLPGQRLLNHSEELHVSACEVTWYIFPKKYQN
LYKFLLARSLIFSKLTAFKVTTLSMQTFLAIIQTAMSYFTVLLSTT
>AmelOR141
MIDEKTKREFDKTIDLNLFLLKLCGIVPCGDGFARNILAWLAFSCLTIYSISYVHEFITNTTN
LTTALESVAMIISIVGGHARYTILLWFRDICQTMLNVCEIFWSNLKPHEKKIVQSYTRKTTR
LTRWYLASCVLTIAFYAFLVLFGSLFDQSKDFEHMRNDSSLVPSEAGNILESMSKRHLPYAF
FLDVQKTPWYEIVYAVQLIGMFNVGFTCVGVDTVGALFILIICGYFDTIQSRIENLHSFDTS
LSSSLLNILSRKITTAKMSDIKTEASNSVQMRNLRMCVIHHQLLLNRFCEDIEHLTSGMFFI
QVIASTYNISLVGFKLLEDTPDKFKYITQLIILIIQLFLCNWPADLLLSKSIDISRATYSMPWY
GYSYNLQKITNILMIRSQKAVRLTAGKFIGLSLETFASMISTAASFFTMVRSMN
>AmelOR142

MKNRLTPEKAILFTKLSVALTCSWPPSPLATKAQHLFFNALWCIAFLTSVMLFLPLLAAIYV
YRKHPVILGKTVSLTAAVAQVTIKMIICRLQQKRFQMLYSEMENFCKQATNEEKIILQRYV
DRYKYFHSFYILWSFLTTIFVICGPLYTVQTFPTHAIYPFSVRRHLYKGLIFFHQSLVGFQVSS
GMAIDTQIALLLRYATARFEILGIQFNNAKSDGEFDACIKKHDELLRYSREIRQSIKFLILAT
NGTTVIAVIFGSLNLIANQPLILKALYAIVVFSASVELFMYAWPADSLMHMTMKMATKVY
NMDWYGKDIRTQRKILFIILRSQKYESFGINGIVPALSLSYYGKYLYTSLSYFNALRIMVED
TVN
>AmelOR143
MNIRQILYILELIGTFTCTWPINPNISKRRIIFRNIFWIFSILNVILLMTSLMLAVVYFRNDILM
SLKTASEMAALLEVVLDLILCKWNNSEFQVLIEEVKSFVEMANEYEIKILQGYVNRYKKF
FSTVSMGYISTAISFSLMPLFSAQKLPADGWLPFSTEPFGIYCIIYFNHVYCILQTAFCIFVDF
TIVILFSFPAAKLDVLRSKLRHVNNYDTLVSCIKEHQKIIGFVEDTKATVETLLFKTNVTMG
STVMCGAFPLLNNQSLAAISQFLPLVLSGILHLYVIAWPADDLRESSVQFSNSISDIQWLGQ
SNKMKSCVIFMMMRSQKAFLIRMSNLLPPLSLEYCSNFITTVSSYFMAMRTMIES
>AmelOR144
MGMLNMDIRQVLHILELTGTFTCTWPINPKDSKKYIIIRNILWTFTILNVIFLTISLMLAIFHF
RSNIPKSMKTASEMAALLEVVLDLVLCKWNNSELQVLIEEVKSFLEMASEYEIKILQGYIN
RYKKFFSTVSMGYILPASSFILMPLFSAQELPAEGWLPFSIEPLGIYCVVYVNHIYCILQTSF
CIFVDFTIVILFSFPAAKLDVLRSKLRHVNNYDMLVSCIKEHQKILGFVEDTNATVETLLFK
TNVTMGSTVICGAFPLLNNQSLDVVTQFLPLVLSGMLHLFVISWPADDLRESSIQFAESIND
IQWLGQSKKMKSCVIFMMIRSQKLFLIRMSSLLPPLSLEYCSNFVTTVSSYFMAMRTMIES
>AmelOR145
MGMLNMNIRQVFYILELTGTFTCAWPINPNDSKTYIIIRNILWIFTILNVIFLAISMIFAIFHFR
SDIPKSMKTASEMAALLEVALDLALFKWNNSELQILIEEVKSFLEIADEYEIKILQGYINRY
KKFFSTVSMGYILPASSFILTPLLSDKELPTEGWLPFSIEPLGIYCAVYVNHVYCILQTLSFCI
FVDFTIVILFSFPAAKLDVLGSKLQNVNNYDMLVSCIKEHQKILGFVENSNATVETLIFKTN
ITMGSIVICGAFPLLNNQSLDVVTQFLPLILTGMLHLFVIAWPADDLRESSIQFAESINDIQW
LGQLKKMKSCVIFMMIRSQKLFLIRMSSLLPPLSLEYCSNFVTTISSYFMAMRTMIES
>AmelOR146
MFRNATPEKAIAFTQFIVSLSCCWPLPSTATKLQTRCFKIIRSLLFLNSLLLFFPLLYFVYVNR
NDNTTFCKAMSLSLAVVQVPLLSSFCITQYDRFQRLIKEMKFCCENANSYERQVFQGYAK
SYATFYGVSAIWFYWCALIVVVGTLFISDPFPTNAEYPFPVHFEPVRSIVFVQQALVGFQCS
AHLCVNIFCALLLLFAAARFEILMNELRAVENIESLIKCIEKYYAIRRYAEEVVNSARYTTLI
TLCICGVESVFGGIIFIGRQPFTVKLQFLTLSATTLLAVFMCAWPADYLMDVSENTMRAVY
ESEWYKRSLKLQKFVLFATIPQTPVILKVRCIIPAFSLNYYCSFITNVLSMFTALRVLMYKD
EN
>AmelOR147C
MLKQVSPEKGIYIIWLSVALSLCWPLPINSTRKQIVCMKILQIGAIISAFMILLPLIYTIYLNL
DNLNIFFKSICLLMGVFQHIVQTITCFIKYDSLQRVVEEMMICIKEMQLNEIMCAYVAKCNI
FYGGTIVLIYTTATVFILGPTFLPITFPWETEYPFQVNYTSRNFIIYMHQFFFTYQCAAHICVS
MFVALLLWFTSARFECLVKELQKTTNIEMLIVCLKKQLLLRRYAEDVVNCIRFIIFYTMAVS
TIVLTLSGIILITTSSLLVKIQFLTICISILLEIYMYAWPADHMYDMSITVLQSVYDSMWYGQT
LNMQKLVLITLIYQKPVTISINVVLPTTYFALLLF
>AmelOR148

MLKYVTPEKGIYIVWLSVALSLCWPLPASSTRKQIVCIKILQIGAIISAFMVLLPLIYAIHLNI
HNLINLFKCICLLICVFQNIIQTIICFIKYDVLQRVVEEMMTCVKEEQLYKVLCIYVKKCNIF
YGGTIVLTYGAATVFVLGPTFLPISFPWETEYPFQINDTSRNIIYIHQFFFTYQCAAHICLSLF
GALLLWFAAARFECLVEELQKITNIDMLIVCFKKLLLLRRYAEEVVSCIRFLVFYAIAVGTF
MLTLSGIIMIINSPILVKIQFIIICMSSLMEIYMYAWPADHMQDASINILRSAYNSIWYEQSLD
MQKDLLIILMYQRPVILSINVLLPELTLRYYCSYVANAFSVFTALRAVVEDK

>AmelOR149CN
PARRVVEEMIICIKEAQQYERKIFCKYIENCNIFYGSSLTITYLVVIIYIMGPIVLPTPFPVDTE
YPFHVNSTIIKIIIYLQQSLLIFQCAGHLCISIFCALLLWFTAARFECLIVELQKITNIGMLIICI
KKQLRLRRYARNVVNSFRFMIVYAIGVSTFALILYGIIMIVKAPLIMKIESVTLSFVLLLQIYI
YAWPADHMKDM

>AmelOR150C
MLKQLTPEKAIHITWISVAITFCWPLPANSTKIQVFMFKTLQIISIINAFILLLPLLYSVYLHFD
DIVIVFKSIALCVGLSQMIIQTAICFVKYNTLQRVIEEMITYVKEAQQYERKIFHKYIKKCYT
FYGCSIICMYLTGLAFIIGPAFSPASFPADAEYPFQINYKSIKVIIYLQQTLVGFQCTAHICLSV
FGALLLWFTAARFECLIVELKKITNISMLIVCIKKQLHIRRYAKKVVIGFRFIILCAIGISTFAL
TLGGVIMIKKAPFIVKVQFITLILTLLTEIYIYTWPANHMKDMSINVSQSIYNITWYKQTLR
MQKDVLTVLMYQQPIILSINCILPELTLHYYCS

>AmelOR151
MLKQLTPEKVIYITWVSVALTLCWPLPANNGKIQVFMFKALQIISIINAFILLLPLLYSVYLH
FDDVIIVSKCVAVSIGLTQVITQTIICFAKYDSLQHVIEEMIICIKAAQQYEEKIFHKYIEKCY
TFYACSITCMYLTATAFIIGPAFSPASFPIDAEYPFQINYTSVKIIIYLQQTLVGFQCAAHVCLS
IFGALLLWFTAARFECLAVELQKITNIGMLIACVKKQLRIRRYAKKVVISFRFIILYAIAVSTF
VLILDGIIMIMKVSLIVKVQFITLSLTMLTEIYIYAWPADYMKDMSTNVSKSVYNITWYKQT
LRMQKDVLNILVYQQPIIFSVNCILPELSLRYYCSYLSNAFSIFTAIRVMIEDDP

>AmelOR152
MLKQIISEKTIQIIWFSVAITFCWPISLNSSKTQVFIFKILQIISIINVFMLLLPLLYSVYLHFNDI
IIVSKSIALSVGLIQVIVQTIICFIKYDSLQHVVEEMIIYVKEAQQYEKKIFHKYIEKCHIFYG
CSIACIYLTATVFVIGPVFSSASFPADAEYPFQVNSTSMKIIIYLQQSLIAFQCAGHACLSIFG
ALLLWFVSARFECLAVELQKTTDIGMLIVCVKKQLHIRRYARRVVISFRFIILCAMGVSIFSL
TLGGIIMITKSPFIVKVQFITLILTLLTEIYMYAWPADHMKDMSINVSKSVYNTIWYEQTLR
MQKNLLNILMYQQPIILSINCILPELSLRYYCSYLSNAFSIFTAIRVIIENNPS

>AmelOR153P
MVKEMIPEKTIHITWLSVALCWPLSVNSGKTQVFIFKMLQIISIVSACMLLLLSSYSIYFXH
GQCRIFKNYHRFIDVAQNIIQTVICFYIIKEMKICIKETQEYEIEIFQKYIAKFKTVWGCNITC
MYLTALAFTIGSVFISTSLSCDAEYPFQLNYTLVFAIRYQSFLSYQCAYACADHXLLWFTAP
RFECLCVELQNVTNINMLIVCXYAKKMINWFRFIIFNAIGLSILVFTLASIILIMISICMYIVV
CSCMYNFINKNYYMYIWPADYMTDKSINVSRKIYDSMXYKQMLKMQKNLLKXLIFQRP
VXIYRLZLLSKLILRYYCLYLSNVFSIFTALHVLEDNI

>AmelOR154
MLKKVTPENVIYIIRLSVAICCCWPRPFNSTKNQIFAFKVLQISTIISAFMVFLPLLYSIYLHN
DNIIHVFKCICLSIGITQLIVQTLICFIKHNSLQRVVEEMVNCVKQAQQSEIEIFYKYIEKCKI
FYGSSIAFSYLAATAFMLGPAILPISFPLEAEYPFHVNESLITIIIYMHQSLVSYQCSANVCVSI
FGALLLWFTVARFECLIEEFQKCSNIDMMIACIKKQLQLKRYAEEIINCFRYIVLYGIAVTTF

ALILCGIILLMNIPLIVKIQFVIICITIMTEVYMYAWPADYVKNMSINISRSVYELSWYEQTIE
MQKNFLNVLVYQKPVIFSISCIVPELSLRYYCSYLSNVFSIFTTLRVLLEDTSA
>AmelOR155C
MLKKATPEKIIDIIRFSVAICFCWPYPLNSSRNQIFGFKVLQISTMVSACIMLLPLLYSIYLHN
DDVIHISKCICISIGVTQLIVQTLVCFIKHNSLQRVVGEMMKCVKEAQQNEIEIFSKYIEKCK
IFYGSSIIFSYLTSTAFMLGPIILPISFPFDAEYPFHVNHSLVTIIIYIHQSLVGYQCSANVCASV
FGALLLWFTVARFECLIVEFQKCTDIDMVIACVKKQVQLRSYAKEVIKCFRYIVLYYITITTF
ALIISCIILLMNVPLIVKMQFIIICVTIMTEIYIYAWPADYVKNMSINISKSVYELSWYEQTLE
MRKYLLNVLIYQKPITFSISCIVPELTLRYYCS
>AmelOR156
MIEQVMLKRVIYITWLSVALCFCWPVSANTSRNQIIVFRFFQIFTIISSCLGSLPMFHSIYLHQ
DDIVIVAKSISIMVVLIQLIVQTTICAIKHDTLQHIIEEMITYMKEAKQYEKKIIQKYVSKCYI
LYGSAIIISYLTTTIFILGPIFLPISLPFYTEFPLSLNNTAVYIIIYFHQCFFAYQCSATVCLSIFGA
LLLWFVVIKFECLIMKIQNISNKDMMVICIKKQLQIRRYAKEIANCFRHIIFYTIIATSFNMIL
AGIILIMNPLLVIKIQFMITCFTALIEVYLYAWPAQYMDDMSKNVSISAYNLKWYEQTSEM
QQNILIMLIFQKPISLSINFLMPKLSLRSYCAYLSNAFSIFTALRVILKDNSI
>AmelOR157
MRRARPEKSVYLVWLSVAMTFCWPLPPDTARKRIVGMKVLLIISIVNGCAVILPMLYWIHL
HLDDIISLFKCICVALCLVQYVAQTIVCLVKYDTLQRVVDEMMGLIEERRMYEILRAYASK
CNTLYGASIASIYVCGTSFIFAPLFLPNPFPFETEYPFHVNTTTRIFIIYASHVLVIFQGTAHMC
LCMFGALLLWFTTARFECLIGELRGVTSVDTLVVCLEKHSRLKRYAEEVVSCIRFLVFHAIL
LGTFVLTLCGIVLIINSPLIVKAQFIIICVCILLEIYLYALPADYMYDMSMNISRSVYDSIWYE
QRLDLQKALLTVLAFQKPIAVSINVLLPELTIRYYCSYVSNALSIFAALRTVVE
>AmelOR158F
MLKQITPEKSIYIIWLSVALSFCWPLHINSTRKQIMYIKILQISAVVNAFMVLLPLIYTIHLN
MHNLINLFQCICLLICIFKHIIQTVICFIKYNALQRVVEEMMICVKEEQLYDILCMYVKKCNI
FYGGTIVLIYGTATVFVLGPIFLPISFPWGTEYPFQVNYTTINVIIYAHQFFLVYQCAAHTCL
SLFGALLLWFATARFECLIKELQKITSIDMLIVCLKKLLFLRRYAEEVVSSIRFLVFYAITISTF
TLTLSGIIMIINCPLFVKMEFITISISLLVQIYIYAWPADYMQDMSINVLRSAYNSIWYEQTLD
MQKTLLIMMAYQKPVTFSINVLLPELTLRYCCSYVSNALSIFTALRAVVEVT
>AmelOR159CN
YRLEHAMDTYKGQNEIEIYYIICTKLIKAIEVYKLAVKFVFFCQIFFFFQGNFNLHFPLLKV
VYMLQQLENIYKLCINLILLIRKFCFLFLITYLGQNIENHSNEVFEKCYDSLWYTAPVATRK
LLLIIMINIMKPCQCKMFGGLFKGNIEGFAQIIRICISYFMSLYSTQ
>AmelOR160
MRRPISSYVELFYDKNVISWSKRLLGLSGLWPDNRNDVRFFLYITYVVIFTWLEIVTLVQNI
HDLEKTLKNITLSFPTILIVLKAVMFRMNMHLVLPLLTVVKRDVNEGLYRSAEERRTVVW
YNVAATLFSTSSALSLFFVPTLFYAKPIIGCLLSKYNNCTLPFELPMKVNNVYEITKLQTYA
LFCVYLIPTSTLLTIGATGADSLLVTLTFHLCSQLSIVAYRMRNVNIEPKIYFPKMKALVERH
TELLRLANILANTFSSLMFVQTLGLIFSLCIVVYQLLMTSESGEDMNTIHFIIYSCAVILLAF
CYCFLGECLINESSEVQMACYFTNWYDLPEQYTRSLIFCIARAQKPLYLTAGKFYVFSLETF
AVIVKASMAYLSVLKSII
>AmelOR161
MGEFRNEEYDQLIKPIMITGKIISIWPLAENSSRITITFRRFHLFCMFFLVIVMSVAVTADVV

HNIDDLDEATECALICTAFYLCVVRLLVYSFHQKDMLYVVNTMKEDWLSSSDQDRLIYAE
KTMFAFRLAKYFITTVAITIVMFMSVPILEIYVIGNSDKVLPFRGYFFINQTVSPIFEFLYLFN
VTAGGFGGSMIAGATSFNLVVIIHGSGKFAVLRRRMEALNGADPNSAAIMGDNVIRHQQAI
KFADTLERIINLLALGQFVISTGLICFAGFQITSMMEDKGRLMKYSTFLNSAILELFMFSFSG
NGLIDESEGIGESAYNSGWIGSRFCRSVQIMMMRSKIPSKITAAKFYSMSLESFSAVLSTSFS
YFTVLTATKNE
>AmelOR162P
MKRLMDIMQEDWKFHARLRNEYEILCEHYAIARKITTSFVAFLLGLTTPFGAMPLLLNIGD
ALGLCNISDDRPLAFRVEYFVDVDKYYYLLLVHSSIGTLGYTVIVLAINSIIIVYVLHECGL
CEILRESNNMICKIFLKDSLDIYTLDKIMNQFIIVQGETRKLCGNGCNGYRITPPQZKGZMV
SKCQGLCTLAQTHNRVSIKLITKFQLILTYLFFFLLIFQISRFLSLRFAKILEDTNTTSYLLQL
GFNMICISFTQFQAIINIEDTPKVLRYVSITIALLCDLLFVSWTGQQLSNSTERIFEYTTNGK
WYQSSISCRKLLAIMLSKSIAPLRLTACKLYTLNLESFTTIAKTSVSYTMVLCSLQ
>AmelOR163
MFKTIITYPVEVCLRLIGVWPYSSYRIMQRIFWTIIMGNSTVFQLWYCISYFKTADLFDLLD
GITLTLSNTVTFFKLIILWFNYRTIHNILTIVFEDWNNRALTDKKKQLMVDNTRLSSRISNFL
FGIYSVTCILYSASIALISDDIDNTNNELILNNKKLLLKMKLPFDFTIFPLYEFVIVAQFVFEC
FVALTAGMLMAFSAALVLHIGSQIDITCQELIEIPRHKGKTSYILKNIIVKHQRILRLSENVK
YLFLYTSLIQFLSNILVICFLGFILVNALGTEQESTIFIKCFPYYIAANCEAFILCYTGEYLMFK
NESIVHAAYDTLWYNLNPRDSRIVLLILIQAQRKLILSAGNFVTLSVQTFASMQKVSASYIS
ILMTIY
>AmelOR164F
MAIKSIINRPVEISLRLIGAWPNSSCQILKYIMWTIVMSIFLIFQYSYCIIHIKTATLIDILDCLSI
TCSNTLLLLKFIIIWFHKRVLFESLIIIAEDWDNCKFEWNMEIMMQKAILSYRIAKLMLIIFIC
SIFMYAVSTFFGPDIGASHSDQKKFLLKMEFPFEATVSPLYEIIITIQLVMQFMFATMAGMF
MTIIATFVLHIASQLDIICDRLSEILDEHKEQELRIRIIKKLIAKHQRTLNLSENIENIFTFISLS
QFFFNILVICFVNFILVTSIGTEQAPTVISKCFPYYIALNFEALILCYTGEYLSSKSENISWIAY
NSNWYELSIYEIRVLLLLIMRSQKPLTLTIGKYMKLSLETFANMLKISASYASVLYALE
>AmelOR165C
MLVLNTLSPSVKFGLHFAGIWPGTPFPYLHKLGWLAAIAALQSYQYRYIVMHYKSDNLM
SIIDNLSIAMPFSLVFIKLIVTWINYGVFCDILSTMEKDCQKYAVIDINNLISKTGQISFYTTTI
VMSSYLVSAAFYITGTLAFQRTNSSISRELLFKMDLPFETNESPNYEFVVTSQLLIHVSAAF
TFGTFSALLLMMVLHIGCQIDILCQNLLDIPHISTSHLKFFIIRYQEIITFAERVEKLFTYIALS
QLVSNTLITCCVGFLIVIAIHEDNGLPLLLKSVLFYMVICLEAFIYCFAGEYLRIK
>AmelOR166C
MTSINTISRSVKYGLYFAASWPGASFSILHKFFWTIIFCTLHISQYSYLIMHYKYDALTEIID
NISICLPHSLVCIKLFTAWTQNTLIRNILLSMEEECQKYAIMDTDNLISKTAYLSYRLTSTIICT
CVASTVCYAIGIFSHQEVNVTSSRELLLKMNLPFDTNKSPIYEFVVIIQYFYQVSAAFVFGV
FAAFLLMIVLHVGCQIDIMCQTLMKTTHRDQKKLKFFIKRHQEIILLAEKIEKFFTYIALSQ
LISNTLITCCLGYLIVITLHLGNNIILIKYIMFYVAVCSEAFIYCFAGEYLSIKSKLIADTAYEFL
WYNMNPNESRLLIPIILRAQRGFTFTFGKFATLSMESFTA
>AmelOR167C
MIPIRSISHPIVIGLRLIGIWPKSSYEIIVRFMWVIIMMCAQIFQYQYIINHIGFDNLADLIDSV
STTLPYSLLCFKLISFWTKREIFENILIGMYHDWTNAFATDFIVEDMIKKTELAYYCSNLILS

IYAIAVFLYVGVFLELSHDHDQENRSNLSPELLIKMDLPFTYDESPIYEYVFIVQFIQLFFIAS
SIAVLDALIITLIFHIGGQIEILHKTLKNISINDEKPESSRIIIKSLIDRHYRIIIGSEYIESLFSYIA
LMQLICNTLIICCIGFLIVVALNSNLKLLIRISFFYIAITLEAFIFSIAGEYLSNKSLSVSISAYES
PWYLLSPKNRGVMILLMVRSQRRLTITAGKFMDLSMQGFAN
>AmelOR168
MNFQNLNRLNALANVVSGNFLPMTNINEKSSVISKIYFVIVWIIQLMYLASCTLGLFNVSW
ERALKDGTVNMVLLLEVIILNVYLHSRKKLLRELIGKLNQILINEDEIFRNVTISTTKMLEK
PSRIYIIVNVISIIVWISSPLIKLFQKDEFYHEDFVMPAVFSNQPFSTGVFISGVFLQLFGGEYL
LFRKISLDLYTMHLNLLITSQYKYLRIKFATILKENGESAKDNDKTIRQEMKLLIRHFETVIE
MTGILKKLLSPNIGILYLNYVFRFCFLSFMFATTSLSEKLTYTIIVSYTTGALIQFYILCYCIQ
DLFEASTSIADDVVYEKWYSYDVRFQRVILMISLANELKCKISNFQNIDLTLPSFMSILNQA
YSICLLFLKTKQD
>AmelOR169
MKMNFQNLNRLNTFVNAVSGNILPITDMKKRLSIVLKIYSILVWTIELSYLAACILGLFNVS
RERALKDSTVNIVISLEVFVLIVYLHNRENLLRELIGKLNCLLIVDDETLRDVTIGTVKPLE
KPLRVYIIASVGSLMIWASLPLAKIFRKSEFYYTDYQVPAVISNEPFPIGVFIGGVALQIFGSA
YTLLRKVSLDLYTMHLILLITAQYKYLRIKFAAILEQETPKDFFYGGIIWQNVPCEYDKMV
KQEMKLLTRHFEIVVEMTVMLKKLLSPNIGILYINYVFRFCFLSFMLATSSGMHFEKCLLV
SYTIGALIQFYILCYCIQQLLEASTTVADDVVHEKWYLHDVKFQHIILMITLANKLKCKLSS
FRNIDLTLPSFMSILNQAYSVCLLFLKARQS
>AmelOR170
MNFQNLNRLNAFANMVSGNFLPMTNINEKLSTILKIYFVVAWIIELIYVAASFLGLFNVSGE
KALKDGTVNIAISFEVIVFNIYLHSRKKLLHKLIGKLNHLLITEDEIFRSVIIDTVKPLEMPLK
IYVIASVASLMIWILSPLIKLFQKDEFYYEDFIMPAVFSKQQPFSNDVFICGIFLQLLGGEDTII
RKISLDIYTIYLCLLITAQYKYLRIKFAIILKEEREITKDHYKNIIWRNDNVRQEMKLVTRHF
ETVIETTTILKKLISPNIGFLYLSYVFRFCFLSFMFAMTTAKYFEKCLLASYTIGALIQFYILC
YCIQRLFEASSSIADDVVYEKWYYYDVRFQRVILMISLSNELKCKISNFQNIDLTLPTFMSIL
NQAYSVCLLFLKARQD
>NvitOR1
MMKMKQQGLVADLLPNIRVMQGVGHFMFNYYSEGKKFPHRIYCIVTLLMLLMQYGMM
AVNLMMESDDVDDLTANTITMLFFLHPIVKMIYFPVRSKIFYKTLAIWNNPNSHPLFAESN
ARFHALAITKMRRLLFCVAGATIFSVISWTGITFVDESVKRIVDPETNETTIIPIPRLMIRTFY
PFNAMSGAGHVFALIYQFYYLIISMAISNSLDVLFCSWLLFACEQLQHLKAIMKPLMELSA
TLDTVVPNSGELFKAGSADHLRDSQGVQPSGNGDNVLDVDLRGIYSNRQDFTATFRPTAG
TTFNGGVGPNGLTKKQEMLVRSAIKYWVERHKHVVRLVTSVGDAYGVALLLHMLTTTIT
LTLLAYQATKVNGVNVYAATVIGYLLYTLGQVFLFCIFGNRLIEESSSVMEAAYSCHWYD
GSEEAKTFVQIVCQQCQKAMSISGAKFFTVSLDLFASVLGAVVTYFMVLVQLK
>NvitOR2
MTSKVSPKLEKAPLAYVNEQYLADTEYVVRVAKTLLMPIGIWPRYGDNSTLSNAIIYIRVC
LIFCLMLFLLTPHFIWTWFKAEDLRKLMKIIAAQVFSSLAVLKFWTLILNKQDIRYCLEIME
NDYRVVESEEDRQIMLKNAKIGRFFTTAYLGLSYGGALPYHIIMPLLQPRVLRSDNTTMIP
LPYPSEYVFFIVEDSPLYEIVFVTQILISSIILSTNTGVYSLIACVVMHCCCLFEVTSNRAEKL
LRGMKYDKSKISPELGKKLSELIDFHVKAIQYAETMENALNIVMLSEMGGCTIIICFLEYGI
LQDLEDREYLGMVTYIMLMTSIFVNVFILSYVGDKVKEQSEAIGFSAYSMQWVDLPNEFI

MKDLKFVMARANQPTRLTAGKLFDLSLQGFCDVAKTSMAYLNFLRTLEIT
>NvitOR3
MAEMKRMEDVFAYYDERMKKPGPSCSNEKFEEDVKYATALNRRIANAIGIWPIFTSTGAR
LGFDICVKTLKNAAVYILLSFLLVPGILHIVVEEGKLKAKILKTGPMILNTMALLKYSVMLF
RKSQIQECLKQLESDWRKAGNDELRALMRRNTAVGHRLSRVCVATFYVGGIFYRLIKTLL
TPIRYTKDGLMIKPLPSPLYKGLFRFNTSASPVYETIFATQMMSGFVVHSTTVTTCSYAVLL
ATHACGQLDIVVYLLKRLIEDDGDNGRLTRVGNEAVDRKLRVIVQLHLKVLRFISSVEDL
MNQICLVEILGGSTILCLTSFYFIVDLQSNDALGLFTYMVMITSLIALLFTYCYVGEIVSDKA
KKVGAKTYMINWYDLPPKKGLCIGLIISVAHSPVQLTAGKMLELSMYNFGCIMKSTAGYL
NLLRTITD
>NvitOR5
MLGEKSHYAVQLNRLFLTPIGVWPIGRDAPLVQRLLKRLAIIGCYLLMSYLLVPTALHTFLE
EPDPAIKLKLIGPMSFHLMAIGKYVSLVGRTEEISACFEHVEEDWKMYSDKNAKPELEMM
KRNAKIGRFLIYLCAAFMYGGGFFYHMIMPLSVGRLVTKQVRAERYLQLVAENASTDNID
VEPVRVLSYPIYGLLAKLDYVTLLLVQFVAGFVLYTITIASCSLAAVFANHVCGQLEIVMSL
LRDFVHDNEDNPRIYALADDAATVERSRSDKFAEIVQRHLRALNFASRVEKNLNAICFVEF
IGCTLNICFLEYYFITEWENQNTVSTMTYCILLISFIFNIFIFCYIGELLTEQSKKIGEVTYAIN
WYTLSGKRAVDLIMIIMIASCYPARITAGKMVNLSLGSFCNVIKTSATYLNLLRTMML
>NvitOR6
METQSTKRIENDRGFNYAVKLTRLLMISCGIWPAKFSTSFQKCLRPILIIICFFIMFFQLIPFCL
FMFLIIKDMRIRLKLLGPLGFSLTSLFKYVVVVIKNREIAKCVQIMVDDWHQLNSTEDRKA
MLINAKTGRVLTMVCMFLMYGGGMPYVTIVPLTKGVTMVGNVSYRHLAYPSYYIFFNPH
VRPIYDVIFATHCICGFTRYTITCAVYSIVIICVMHICSRIAITSSMLQRLADDSDGRLLGTAV
KHHLDILKFATKLENIFKEIFLAEVLGSTYQICLLGYYFITEYEQRAGIATATYLFLFMSFVF
NIFILCYIGQILTEQCESIATTAYTSKWYQLSGREARSIILIVHWNRRRVVLTAGKMLTLSLES
FSSIVKAAGGYLNILRTAVANSN
>NvitOR7
MIESVYKLKMNEDNKQNLERNQGFDYAVQLTRLLLMPCGIWPAKFSSRVQRFLRPFLIVA
CCFVMLFLLVPVCLFMFLIVRDVRIRIKLLGPLGFSLMSLFKYAVVIIRSREIEKCIQNMLDD
WQQVASDEDRDTMFENARTGRVLTMVCMFLMYGGGMPYVTIVPLAKGATMVGNVSYR
ALAYPSYFIFFNPYIRPVYDVVFLTQCLCGFTRYTITCGVYSVVIICVMHICSRITVTSSMLQ
RLADNYDNKLMGTVVKHHLKFLNFAAKLDNIFREIFLVEVMGSTGVICLLGYYFITEYEQ
RESIATITYFFLLMSFVFNIFILCYIGQVLTEKCESIAKAAYTTKWYQLTGKEARSIVFIVSCN
HRPVELTAGKLLKLSLNSFSSIIKAAAGYLNILRTAIVNSS
>NvitOR8
MYSAMDNHDIKDLINAEDFEYAIQILRWLFQPMGIWPLKSAAYPSFLRPISIVISFWSAAFLI
IPGILSVIRVQNDFALRLRLIGPVSFCLVTSFKYFSFLVKNRQFYAYLINVALDWREMKKNN
HNRIIMLRKTQISRFFMTSCSICMYLSGMSYNILLPLTKAPTQVGNVTFKNLPYIGYYIFFD
QYADPYYYVVFVMQCMSSFFCYSTCCGVCCISIQSVLHISGRCDITSIMIKNLNGNCNEKA
LKAVVEFQLQSLKFAREIEKLLNQMFLVEFVGSTFNICLLVYYFMGDFKENDTVGTMTYV
LLFISFTFNIFIFCYLGEHLTEQCASAGAAVYTMDWFRFSAKKSRDLFLIVLFCQRPVVITAG
KMVNFSLLSFASLMKASAAYLNMLYKMG
>NvitOR9
MVKICPDMNSEDVSAIKNAQGYAYAVQLTRWLLLPLGLWPTKSIIYQKILRPVAILLCLFIM

LFVIIPLCLFIFLVVKDLGIRLKLIGPLGFGLMSLFKYVVVIVKQRDVASCFLGMAVDWQEL
SSLSDRKVMLRNAKTGRLLTIICVIFMIFGGMPYITVLPLTKGPIMRGNVSLRPLAYPSYFV
FFNPQIRPIWDYVFVTHCMCGLVRYSVTCGVYSIAILCIMHICSQITITSSMLDRLVENFDN
MLLGKIVTQHLRFLKFASKLEDLFNQICLVEVLGSTCIICFLGYYLITEYEQREPIATVTYFL
LLCSFVFNIFILCYIGEILTEQCESIGTTAYMIRWYHLSGKEARNVVLIIASTQRPVVMTAGK
MVNLSLQSFTNVIKASASYLNMLRTVTANAN
>NvitOR10
MSKQNLISNLIELKSIRNAQDFEYAVQITRWLLQPLGIWPMKSSTFFSSILRSLSIATCTFLLG
FLLVPCCLHMFLIEKDLGVRLKMIGPLSFCLMNIFKYYAVLIKDGQISSCIVDMAGDWHRL
EGSEQRGYMLENAKTARVFTTICALFMYGGGLPYSTILPLTRDAIIVGNDSYRHLAYPSYFI
FFNPHIRPIYDLVFFAHCLCGFVMYSVTCGVCSIAILCIMHICSQCSITSATLRSLTSDVDEKT
FGKIVTQHLRSLKFASKLEKILNDMCLVELIGCTFNICMLGYYFITEFEQSETVGTITYSLLL
ISLTFNIFIFCYIGDLLTEQCENIGEVAYMINWYQFSGKDARNIILIVASTQRPVVLTAGKMV
TLSIRSFCNVIKASVTYLNMLRTLTASES
>NvitOR12
MADKKGYEEAIEATRAVLRAFGVWPNRHKISENWLSRSHFLAPAFLIICFINIPQTLKIIKV
WRNLNEVLDILVTANIPSFVALIKLLCVRYNKKVIGLLLVSMENDWKSLKTLVETRIMWK
NGKLGSLITLVIYTLTCGSYVAYVIMITYINVGGSKQEDVITLNESKKLRPLYMRSYFIYDV
QKTPVYEIIWIFQFVSMGVATFTFMAVDSLFAVLMMHLCGQLINLQERLKNFTNMLGQTK
TRNFSYQLSTIVSRHEQLNRFAKAIENAFNTMFLVQMLLSGMVLCLQGYQIVIILTGRDTV
QIIELLFMVYYTLCFAFSLFVYCYIAEILRIESMEIGNAAYHCDWYDLSAFERRLFILTIIRSK
TPFEITAGKFAAFSLEFYCSILKTSGGYLSVLLAVQDRLAA
>NvitOR13
MTVEIAEDSMERIVALSDDQNVDGYNHAIGPCRFFLRLLGTWPDPYGNVDSWTTSARCL
VITATMFLFATISQTVKMALSYKDLNLVTEILTNCNIPTTIATIKIASIWYYRWVLRDLVRQII
EDWEMSHDRHESAIMWRSAKISRIFSIGCMFMTEGTLLTQCVVGLFRPISYAFKTDLNQSI
EWPLYMKGSFPYDVQSSPNYELSILGQLLSNVFASTSFSSADSFFIVLMFHLIGQLSILKLTI
LDLPSKIENSDDRSKFIDRFAFVHMRHNRLWRFSMAIEESFNTMFLIQMIPCIFALCTQGYQ
LIMIMDADNVSLMELIFMIYFLVLFLFTIFTYCYVTEILRCKSLELSYAVYDCDWTILPAKEA
RILLLLILVRTQHPFEITAGKFASFSLPFYCRILKTSAGYLSMLLAVKKRSEQVASKVVL
>NvitOR14
MSKKSGFDVAVGPSRAFLCFVGVWPNPEGSETTFETIQCIIVTLTMIIFANIAQTVKVFMVW
GNLNSVIEILTTADMPIFVALMKFLVAWYNRKVLKGLVILMMEDWSRSYSSSNLDSMWRT
ARFSRKLSAVCIGLAQGTITAQFIMVVVFDVNNKGEAERTLYMISYFPYDTQVSPNYEITW
LGQCFSNIFAAGAFSAVDAFFAVLVLHLCCQLSILRKELVMLADHHKKQGDNSEEFSRKLA
RIVEKHEYFNSFAKTIEDSFNTMFLSQMIASSLALCLQGYQLVMIITNTEGKLPVFQLIHMI
YFTCCFSFSLFVYCYVAEELRFESTELDYAAYDSDWYNLPPKDTKLLLLLMHRSRKPLEIT
AGKFCAFSLRLYCSILKTSGGYLSMLLAVKDRLVVEAD
>NvitOR15
MAEKEQGFQTAFSVTRFVMRFQGIWPGVDKPRTGFSRFQFIPAALMMVFFINAVQTMELT
RVGGDLNMIIDILTFADIPIFIALVKHVGIAYNNKVLYKLLYLISEDWKEVTKESEKKVMWQ
KARLSRIFTMIEVSLGLGRLFIHTIRMTYAMLHPTSFDPTGKLIRPSYMRGYFIYDSQSTPIY
EITWGCQFVATAFGGCAFASADALFVALVFHLCGQLTNLQTEFREVGKNTSGKKLEFVRSL
ARIIKKHRRICHMADTVEYCFNKIYLVQVSSSSVIFCLHGYSLVTILFDQDDVVVELIVMTF

FTLGFIYSMFVYCYVAECLSTESLALSSAIFDNTWYDLPPKHAKLLLLPLQRTGKPLIVTAG
KFVVFSLNLFSNIIKTSAGYLSMLLALREKL
>NvitOR16
MDDKEGFEVAVKASRTILRVLGIWPNHHERTESWLSRSYFIMPTFILVYFTSFPQTMEIIKV
WGDLNSVLELLTTFDIPNLISLIKILSVWYNKKVLGLLIMAMENDWKSLKTVFELRVMWK
NVKLGRLITLAIYLLTYSTVATYVVMAVYITANAYKQEFILTPDNSTKLRPQYMRAHFAYD
VQKSPVYEIVWIFQCIAMHLAGLSFMAIDSLFSILVLHLCGQLINLQERLKNVTENLTKRH
NLSYQLSRIVMRHEQLDRFAKAIENAFNTMFLAQILLSGVVLCLQGYQIVIILTSRDTVQVT
ELLFMIYFILCIAFSLFIYCYIAEILRTESTEIGNAAYECNWYDLPACETRLFILTMIRSKTPFE
ITAGKFTAFSLQLYCSILKTSGGYLSMLLAVKERLAL
>NvitOR17
MSDKEGFDVAIQTSRTILRFLGVWPDPKRKESWIYSGHFLIPAIVMFYFVNIPQTMMVTKV
WGDLNAVLEVLTTSDIPIGIALFKMLGIWYNRDVLGQLVVSMSEDWKSVKSPEERDVMW
RNARLSRLLSVTIIGLAEGTIVAQFAMVIYFNVLEARQYSLTKDNVTARFRPLYMSAQFFY
DAQKSPNYEIHWLFQCSSTIFAASAFSSVDAFFAVLMLHLCGQLNNLREKLKKLPKQISDK
GGGSFVEKLSEIVTRHDHLDRFGNAIEDAFNVMFLVQMVASSMVLCLQGYQLVMITTAG
DGIPLFELIFMIYFTCCFTFSLFVYCYVAEVLRTESMEVGNAAYESNWYDLPSCETKLLML
VIIRAKKPFKITAGKFAAFSLGLYCSILRSSGGYLSMLLAMKDRLAS
>NvitOR19
MTTKEEGFDVAIGITRFVMRTHGIWPGFSVSKAGIMRYAYLPAALMLLLFVIIPQTVQVIFV
SRDLNAVLNVLTLGNVPVGIALAKLLGVSYKQNVLHQLILSVCEDWKHTTKESELVVMR
LNARKSRMFSIICIVLSEGTAMAYSARMFYAAFSTHTKAQATGIDDCEKPLFFIGKFPFDPQ
SYPNYQITWTLQIIATFLAAGAFSSVDALFVTLVLHLCGQLTNLQAAFSEIGEENAEKGTM
FVSKLSKLIERHRKINVFADIIEYSFNMMFLVQVLSSTLLLCLQGYLFMIILSGQDGLLVEMI
FISYFTICFTFSIFVYCYVAELLQEKSLQLGYAIFYSKWYNLPAKKARLLIISIVRCKRPLEIS
AGKFCIFSLNLFCNIVRTSAGYMSVLLAVKDKIT
>NvitOR20
MARKEEGFDVAVGFSRFFMRLHGIWPGDTSSKFTWARFAFVPPAVIILMFINIPQTVQIFFV
GGDLNAILDILTLANVPLGIALAKILGVSYNHNILRQLIVSVSGDWKHTTKKSELQVMWR
NARISRTFSILFIGLAEVTVLANTARMFYILYSTRSEAESSGIKNYKKPLYYTGKFPYDAQSS
PNFEITWVMQILATILAAGSFMAVDALFVTLVLHLCAQLTNLQTAFRKIGEDKHEKEVDFM
SKLSKLMKRHRKINEFADIIEYSFNMMFLFQVMSSTFLLCLQGYLFVILISSQKVILVELIFM
VYFIICSSCSIFVYCYVAEILREESLQLGNAIFYSKWYNLPANKARLLIIAILRVQKPLELSAG
KFCIFSLNLFCNIVKTSAGYISVLLAVRDKIVQP
>NvitOR21
MKIRKSGYDECVGFTRLIMTIIGTWPGAEYSQHWYARYMFSIPLFFSMFFMIIPQTRMLLH
VKDDLNYIIEILTTADVMIIVACLKLIGVWYNKKDLRYLLNEIEKDWTITEKEEQHVGNAM
WENVKLGKFIMNGYAVLTYGTVVLYAAGMLLLMNSQKIEDFDNENITQSRLMFVRSKFPF
ETQGSPTFEIIWFLQFLAAVMSIAAFTTFDGFFIFSILHVCAQLVNLQCNFRNLISRCRLTKRT
FVQHMRDLVERHIHLQRFTQIIENNFNKVFLMQMIGYSVTLCLQGYQLVISLTENSEQNFIT
IAFILVYTTANILSLFVYCYVAEKLRKESTEIFYAVCAMPWHEVKPEESKMIVNIMYAAKHP
FEITAGKFAVLSFSYFVKVLKTAMGYLSMLLAMKSSHKM
>NvitOR22
MMANNNKLGFDESVGVTRWTMNVIGLWTLDERRDLQTRFRSLLPAFLILFFIVIPQTRKAT

LAHDDLNLMLEILTTADIIEGICLLKIFGLWYNKADLKKLVIQISEDWTHTNNDEQGIMWSNARLSKFVCLFCISSSSGSVLTHAIVFLVTNVGANETRSLFLISQFPFNTQHSPVYEIVCFCQFAGALLSTFIFSSFDGFFVFSILHFSSQLSNLNIRIRSLTEKTSGDKCQFVESLKSVVKHHQHLISYTDIIEYNFNKIFLVQIFATSIVLCLQGYQFVMIISESGTKLLTSLIFILVFTTGNVLSLFMYCYIAEIIRNESQRLLRAVYEMKWYTLPAKDSCLLLIVMCRLKMPVEITVGKFAPFSLEYFASVVKTSVGYLSVLLAVRNKIND

>NvitOR23

MEVKTLVKSDTQISISNNLNGLSGFDHSVKVTRVISRMCGVWPGFEEKKSFTERFFFIVPGMVTFFSITLPQLRRVMIHRKDLSTVLELMTTGIVMELISILKLLAIRYNQSGLRWLLRRMVDDWKIYDKGQYYKIMWVYARHTNTIVTICIALTTGNIAAQIIRQYAIYIIERHYSSANETVIKPTILKSDFYFNEQIEGIYELVVAAQILGGFSVAFSFTAFDGFFVCSIMHVSGQIHKLQMQIEDLVQCYERREGAFSEVLGPIVHRHRDLRGYAAVIEENFNKIFLVQMLVTSVFLCLQGFEFAMVVAEGGTEMVPHLIFIVCFVASNLVSIFTYCFVAEQLRTQSNQLFRSIFQIRWYDLTPKDSRLLIIIMVQTKKPIEITVGKFVPFSLDYFCSVLKTSAGYLSVLLSMKDRL

>NvitOR24

MEESPGFLHAFGICRTCLTMSGLWSDTHFKKSKKFVISVLYAANVFVILTFMNVAQTVKLFLIWGDFDEMSQIISTSDFSVGMLVVKMFVFRSYRKALALLIEFVEKDWLDLKTISEEETMEQNAHTANKIYLTCFFLGNSAVNSYTLLRLGQEMSFLPGPPDKRQPLFDAYFPYDDKRSPAYEITWLMQYAGIALANLAFTGMYCLFVGLMLHLCGQFANLRIKLIEAVSRKEGESEKKSDGAKTFRERLAFIVERHNSLNKYAQVIEKIYHWIFFVEILSSTIQMCSQWFMLVTVISNTQGGLPYLQIGFLLIFTAHSGFHLFACCYAAERLQNESLSIFEAAYSCEWYNLSPQDAKMLLFIMQRTKTPLRVTAGKLCVFGLELFAKILKTAGGYLSILLAMRDRLVIDEEPI

>NvitOR25

MDGKRGFDHAFSLCRINLGTVGLWPNSKNGKGHQEVASLIFFIISLFTIIVFVNLAQTVKLIMIWGDLNHMIDNISTANLPIAVVVFKMLTFRRYKKTLTRLLGIAMDDWCTKKTSREAENMSKNARTARKMSLVCVVLGFGSVNGQLAVRISQELDILPGQTEKRLPMLSSYIPYEYQTSPAYEITWFMQYLGAVLATLVYSGVYCVFVGLVLHLRGQVANLRFMFESVDDPEEDKGKNFRRRLRSLVERHESLNRFAEDIENIFTLMFLAEILSCTIQICLQVFLLVTLMSNDNGGVPILQILFMMVYAMHVGTHVFICCYVADKLRDESLSICDLAYNYEWYRLPARDARLLLFIMLRAERPLEVTAGKFCAFSLRLYAQILKTSGGYLSMLLAVKDRSTNF

>NvitOR26

MDKKRGFDHTFGMCSINLGIVGLWPNSKNTKFQEFRSNVSFVFAIFSVSVFISMSQTAKLIMIWGDLYQMIENISTANLPITVTVFKMLIFRSHKKVLGELLALAIGDWCTKKTEEETANMCANARLAHRISMICVFLAGGTVSIHAVLRTCQELDIMPGPPEKRLPLFSSSYVPYDYKSSPIYQVTWLMQLTGTSCATLVFSGVYCAFVGMVLHLRGQVANLRLKLENICEIREKGEGLVEARRDFRKKLGFIVERHLVLNRFAADLETVFTLMFLAELLSCTIQICLQVFLLVTLLSNIKHGFPILELFFLMVYIMHVGTHVFICCFVADKLREESLLICNSVYNYQWYKLSAQDAKMLIFVMHRGDRPLAMTAGKFCAFSLQLYAQILKTSGGYLSMLLALKDQS

>NvitOR27

METKAVAMTDSRAQVSNYFPDSSGFHKSINITRTISRVCGIWPELEEKKSIAARYYFIVPTIVIFFTMTVPQVRRAVLHRKDLSAVLELMTTGIVMELIALLKLLGIRLNESGLRWLLRRMIDDWKTSNSKERNIMQEYSNLTRFIMTLCITLTIGNVVAQTTKQFAIYFMERYQSMANETVIKPTFLKSDFYFNEQPEGIYEAVVAAQILGGFYVAFAFTACDGFFVFSILHVSGQICNLQLQIEGLVQNHEQRRCSFIKVLAPIVVRHRDLRGYAAVIEENFNKIFLVQMIATSIFLCLQGFEFAMVIT

KSGSEMVPYLMFILCFVASNLVSIFTYCYVAERLREQSENLFRAIFEIRWYDLAPNDSKLLII
IMTQTKTPIEITVGKFVAFSLGYFCSVLKTSAGYLSMLLAVQDRL
>NvitOR28
MDGEKGFLYAFGMCKKSLTLIGLWPKSKSSNYAEAVVVLRFTLTTLLIVSFVNIVQTIKLLA
VWGDLDAMTDIISTANLPIAVAVFKMMVFYKHRKAFEPLLSFVEADWKSYKTDSDMTNM
WSNAQTTRRISMICVILGAGTVNGHLFIRLGQEAKILPGKDGATRLSFVDSYFPYDYSPTPI
YEITWAIQYIGAALATCAYSGIYCLFVALMLHLCGQFSNLRKKLRRVVTNEDDKRKFVEK
LAEIVKRHENLNNFARVIEKIFNLMFLAEILGCTIQFCMQGFFLLTLSSKEGMGLPILHILFM
VIYVLHIGTHLFICCYVSEKLQDESVSIVRAAYNCEWYNLSAKDAMLLVMIMNRAKKPLR
ITAGKFCAFSLSLYAQIFKTSGGYLSMLLAVRDRIT
>NvitOR29
MDDKKGTSFIHAFGLCRINLTVLGIWPTLRSSKRDETAALFRLVLSLTIIILFINTVQTIKLFI
MWGDLDAMTDIISTANLPIGLMVFKTFVFLYHKEALVPLLSFVQTDWSNFKTVSEAANM
WSNALAARKISLLCVVIGWVTVNCHLAIRIGQELRFMSGKNGLTRLPFFDSYFPYDYTPSP
VYEITFVIQYIATMLATFGYSGLYSLFVALMLHLCGQFANLRDRLYTVTQKKAGVTFQQRL
GYIVMRHQCLYNFAQVVEKMFNLMFLAEILGCTIQFCMQGFFLLTLSSKEGMGLPILHIMF
MVVYVAHIGTHLFICCYVAEKLQDESVSIAKAAYECQWYHLSPKDVMLLIMIINRAKDPIE
MTAGKFCTFSLSLYAQIFKNSGGYLSMLLAMRDKIT
>NvitOR31
MDDKDGFEYAFGVCRKELIIFGMWPKPNDTMDHKVFAIFRLVLCIALNFIFINLVQTIQLFI
MWGDLFAMTDIISKASLPIGLVLFKTLVFIYYREALLPLLAYASSDWKKPKSSLEAANMWS
NARTARQLSITCLFIGLSAVNYHMAVRICQELRIIPGKTKVERELYFNAYFPYNYTESPAYEL
TFAMQYFATVLATFSYSGLYGLFVGLMLHLCGQFANLRVKMDKVAKQADSAKFRQNLTAI
IIRHQFLFRFSQIIEKIFNVIFLGEILGCTIQFCLQGFFLCTLSTEDVGLLVMYIFFMVFFIGHIG
SHLFICCYVSERLQDESVSIANAAYKCQWYHLPAKDVMLLVMVINRAKDPIQITAGKFCV
FSLSLLAQIFKTSGGYLSMLLAVRDKIT
>NvitOR35
MSDKIEDAQKKLETREQRLRDFKWALGLNRLSLRLMGVWPGDDEAEGLGRLAILLRVPF
MIAAMFFCLFLPQMGALALVIHELPLVIDNLMTSCAAFTCCIKLYFVWRSKQVLRPVIQSV
SADWLRPKLDWEREAMIREASRARIFTVSGYAVLAGCYTGFAFAPLFGFDIRMISNITDYG
EKHLLVQSYFPYDYSKSPNYEITQVSQLIAGFFIGMSVSVPDNYFGALLFHASAQFEILGAN
LENLVRQDDKALRSRQFNRRFGIFVDRHVHLMTMVTAVEYSFSFVIMAQIFCMSIMVCSL
GFQILGMIEGTTADKPSLLQVLTLLGTLFTLMMHTLVDCFACETLELRSAGIFENVYNSRW
YTVPKQSVAKDVIPMMVVSKNPRKLTAGKIFTLSLATYCSILKSTAMLIAVNRR
>NvitOR36
MERSQKNQLQDFDWALGLNRFSLRLMGIWPADQDESSKSLLTVSRIPLMILVLLCGLFLPQ
MWALALVIEQLPLAIDNLMTSCPAFTSCIKLFFIWRSKTILQPVIDSALQDYLRPKSKSEETA
MQREALRGRLVTIADYSIMASCYVGFIFMPMLGFNVRIINNLTDCDTQRVLLVQSYFPYDY
ARSPAFELTHLLQLAASFFVGMAISIPDDYFCALLFHASAQFEILGLQIESLPIDGSKSGRLL
SGFIERHVHLNRMVSAVERSFEFVIAAQIFCMSIMVCCLGFQVLRMLDSAAEKPTPVQILT
LGGTLFTMLLHTFVDCFASENLAARSSELFFKIYSSRWYSLSWSKMRCLVPMMLVAKTPR
QIRAGKILSMSLATYCSIIKSTAGYISMLIAVSGR
>NvitOR37
MESLEKYRSQEFDWALGINRVSLRLLGIWPADQDESSKSLLTVSRIPLMVLVIFGGLFLPQM

WALALIIEQLPLAIDNLMTSCPAFTSCIKLFFIWRSKTILQPVIESVLQDYLRPKSEWEELTM
RREASKGRLITIADYSLMTICCVGFIILPTLGFHVRIVNNVTDYASYGNRALLVQSYYPYDY
YESPAFELTNLVQLTAAFFVGMTVAIPDDYFCALMFHVSGQFEILGLQIENLMGKDDAKEG
VDWSLLGSFVERHVHLNRMVATLEKSFEFLIAAQILLVTVMVCCMGVQVLRTLNGAGEK
PSPFQILTLSGTVFYLLLHTFVDCFVSESLTSRSSEIFFKIYSCRWCALPWNKVRCLLPMML
AAKTPRQIRAGRIMPLSLATYCSIVKSTTGYISMLVAVSGR
>NvitOR38
MKSNNAHESFFAYLNWAIGLNRLSLRLMGIWPDDSVAETKLFTTILRIPLIISVMMLCIVVP
QMYALILVRNNLLLIIDNFMTSFPTLIGCAKFYFLWRSKEVLRPVVCSVTEDWLRPKSDLE
CQKMRDAAVVARLFTVGGYSLITGSLMGFIIAPLCGLNIRVEQNITDYGRQPLLVQSYYPY
DYSQSPNFEITHSSQIVAACFVAMSLAVPDNYFGALVFHISGQFQLLGLNFEHFIKQNEKIV
GIMAVRDFNKSLGVYVDRHVHLIRMVAIVEKSFNFIILIQIFCLCVMACCLGVRILSAIGNP
NDKTAVIQIINLGATLISLMIFAFSNCYASETLASRSAEIFQQVYSSDWYKIPKRSTCCYLIMI
MIMSKNPQMLSAGKILYLSLSTFCIILKSIAGYLSVLIAQSN
>NvitOR41
MHRMRTRVKRLKVSRSSKTKGGFSYEFRIYKIITWPAGLWPLERDNIFNVLRFLLAASSQM
FIVVAALVEIYRKCGNVADVLDYYALSIAFWLSFVRLVLVRIHLAKIHKICYNARKTWARIK
DPDLVKIMISHAKTGKRFYYLQMSIAFVIVTLYVFNPLILRRYDAANLPMQTVCTFNNADV
LKHTAVYFIETLSFVYLAVGFISIDLLFLGIAMHLCGQLKILQKEFSEIVGKSTSQADCIRYVI
SLSRRFQRVVELTDDIRKTFSEILLVNFVVNLFLITSQSVTLLLALKINNYFLAVKCSQTFPIL
LIEMFLYCYVGELLRHAFDDIPRAIYSSRWYLLPPKIRRGYLLHVMAQASKTFDLTAGKMI
RMNMCTFIQLVRSIVSFFSLLLLMFDK
>NvitOR43
MKCFDSYDKSNFKTDPQHAINFFKKLGRFWTIWPVSANASRFTKVYHECSLWFIIINLFFA
SLTLWMSVCVYHKYPILMAKNLSQLMIISDSFTHLVLYRINRSELQVLVQEVFDFMKNSKQ
NEKYIMRKHYNRFLGHYTILIVLYVIASLAFFCGAFILGKKFPMDASYPFSTDSILVSSIIFTH
QTFSIVQNSVLIMIDLLVITLFWYAGARIKILGYKFKIVDSNEKLKNCIKEHQKIIQYVASIV
KAVRFILYKTISIVAIIIISAGLQLLYYDAKVVISQFSLIIIVACFRIITYSSTIEEMNQLNEDLRW
TVYKSSWFCITSEMKQCLQIFIHRCQIPLVTIDGQLLNIMSLAFFAKLIYSTVSHLTTLRAIIE
RS
>NvitOR44
MAFKISPEKAFTFTKLSVFFTAIWPPNCNDSSFKIKLANIFWIYSIISAMCLLIPMLASVLVY
KDNPMIVSKSICLSCAVIQVIAKAIVCRHHQKKLQFLVKELTHFLKKAKKEERQLIEKYINR
RAIFHMTFTLCCFGSSFFVICGPIFLPFSLPADAVYPFSVNSSPIWEIIYVHQASVGIQASSGM
CVDNLVAYILWYTGVRFESLYYKFKHIKDSKEMLQCIKEHQYLLRYGTTVADTFRYVIFTT
VLSVTAGLAFAGIYLFSPQPIFVKGQFVVVSISVVVNLYVTALPSNNLISMCHKVGDVVYE
SLWVGDSPSIMKHWIFIIQRCQKPVVIAIPGLIKELSLQFYSSVLCSTFSYFSALHVIMTKE
>NvitOR45
MKFLIRPKFFFKTLRLIGDMIAIWPKHIGAKKTMIMFHEVKWWLSFTNATGLLIPLVLGVY
YFRNDSITMTKTLSELTALCEVFINLIQCRLQEKKFQVILYEIENFIENSNEQEESLLQDYLN
RYKTLQLFVGSSFISTAILFSCIPIFTSQLLPADAWYPFSVEYFPIRVFLYITQVLAIFQTGFGIC
VDLTVATMLWYSAVQIELLEKNVHKAVSKAELRECARRHQEIIEFTDNIKKGIKFIILKTNA
TMIIVVICGAFQLIHHEPLEVLLRFTLMVLAGCLRLYVSAKPADDLKENSEQLARTAFQTA
LMQKSTSNSKIGLMLAFRCQKPIVLSVTAVIRAYTLQYYASFLSRTVTYFVNLRAVLDD

>NvitOR46
MRKMKCTFTFSIISVYETLKYFGLLTSIWPIFSKNRYLWVFMKAVYYFILVNYLCVFIPLIL
MTLFNINTSATVTITAVEQMIIVEAVYNLLYTRFYSAQFKSAVKEIEEFFKNSSPKERYILDRY
ATTRTFFNIYIAINYFVAIMSFNFGQLFLKDRPYPLNLWYPFTIKSQVIVVIIYIHQVIVITHTL
ILIVFDLIVQIFLWTLAARFELLQADFKKTASEMDLKCNIQKHQYLIRTTEAVIDFTKYMILK
VFLAVTILVISSTLQILHRGPSTIIVQFFFIMKIASMRAFAYCWAGHSLAEKTGGLARSIYNS
YWINQTQRMKTNVLIVMQRCQKPTVVKISGIISSLSFRFCVNYFYMIYSAFMTLRAVLEV
>NvitOR47
MSSWRMGSIKDIMMNVFMMKVIGVALAIWPLKSAGKRWYYAFLQEMVYRFFHVNFWLL
VIPSLWSIYKKRHNLASVLTSVTQLTIVFEILAIMVLSRRQAARLKTLLTMAYDYVSVADD
KVYPVVYKYVRKAQIIFGIITIAYALILLTYLVQAFIENKPPIYAYYPFDIKSPVVWICVYGN
QLLCTFYAAVVIIMDAMMMFMIFVTSIRLELLQNDFKKVKDYPDLVKCIRTHQDIIWYIKE
VYCIKKYMVLKMLISIAIYIMCEGLQLFALNLSWGMRFQVSLLFGIGLFRVYIYAACSQDLI
SSGLDLGYFVYSSLWYNQSHSVMVAKAFVICRCQKSLGIRVCGITDDLNMKFLANFLYRV
FSYTMTLRAIIKTLR
>NvitOR48
MMINKKVTDKLVTIVSTLLNIFLHIDYLFHSFKILKFFSRLFALYPLNSDCTKLEILYDNFVW
LFIHFHFWVAAAATLVAIYKARSDLSIWLIAFSELIIIIEIIVAMILYRLQRSRLKILMHIFEDFV
KDPDDSKIQLIRRNAKEHIKVFSILALLFIIVVIMYVYRALSTRPYQLLLSGYYPCTSDSLII
WLVVFFHQCILVIYSPSTFASDSIVTVLIFAAIIKLQKIRPRFRNIENYAQLVGCINEHQHIIWF
VQEINYVIRLFVFKSIFCLAALQLGVGVILFMPNISVFTRIQFLLLFTVTIFRIYIYSYCAEILT
KSGLDLGFAVYSSRWYDQRRKMVLAKSIIICRCQKPLLIAINGIIPALGMRYLARFLYLTFSY
IITLQAMTRT
>NvitOR50
MFKKIKPNFTVQKNFNILCNCMKILGTWPVHRRHNKIFTCLNHSLWWFYVVNHMMLLLP
TMQTFYNTTKDIISASYSLIEITGIVESMVILITFKLQGSRIQLLLQIIKNQIVVKKPKPALNN
RNVHASVFAIIAVLYVIVVYMYIHKPATLINKGFIMTTCYAFPTEDIRTKIAIYCNQLIALMH
TSVVLVTDGVAVLFIYTCAIKLKTLEIRLKKAPDWTKLKYDIAEHQTILLVIEETNSLAGVL
VVKTVICFMCYSISAGVQIINQHVVTAQMLHQFIIIAIVYLRIYICAETAEMLLTVNGDMLF
TVYSIAFSTPNIVKVKSLIIMRCQKVPKIYVNGLMAALNRAYLRSISYATFSYFMTIRAIVSK
>NvitOR51
MRIHLNAKIAYQYLRLSATLMATWPLSDATKKYRNVFYNMLWWLYLTNHLIILYLTLNTII
THNKNHLTVFYTWLEISFMTENIIVLVSYKLQETKWKQLLYTSKMTINRTEDNIRLENSDL
YPKVFATLFIFFIVIIISYVNKAETYERGLIMTTRYPFEIKSIGLKLFLNLSQFITLLHASSILIT
DAIVVLLLYTCTIRLKIVEQKFRACKYYRHLKLHIYEHQKTLLLIEDTNLLVSKTVLKSIAS
FMSYSIGGGLVLYNKNTSPLQLVQICLVICVIYLRVYVCAEIAEKMISANESIGFTIYFTKWY
EESAKDINAKNIIIQRCQKLPRIYINGFMQSLNRNYVRMITYATFSYFMTIRKIINKTANCVD
C
>NvitOR53
MQNKFKLRDRTNKFTFMFKSYSGVHLGFLCVNFYMKCLGIYPLPSTVSKFWTRVYNLLW
CFYLSNHLLIIFPTFYAFGSTTQDIAVATFSLMEGLCMIECIVLLIHFKYQRSDFKILLSLVHH
ELNKKKRIITLDNGNVYIIAFVLIAIMYVLIIFNYIQRPETVRYHKLLTTARYPFSTRAATIKII
LSCHQIVVLLHMTIILTSDGLAVLLTLICTVRLKNLETKISNEKRGKLPKRIREHQQILLQVE
ETNLIVRIIVIKTVFCFMVFSISTGLQIFHKFEIIQIFIVMIVFLRFYVSAESADNMATCANNL

GIAVYSTAWYEEKTKIRIAKTIIIQRCQKSPRIFITGFMSELNRKYFLVVAYATYSYFTMIRTLI
SKNK
>NvitOR56
MSVYISPNRSFRVLRFLGTHLRIWPDDNKKWNFKTDVFFWFCVINYVLLLLPLFNALYLN
RKNVVAASNTWIEVSGYAEVLAAFIYSKYKRVQLYVLLCEAEKYLLFKKVTIIKKYANTY
AKIFLLVIVFYLFTVFVYWSIEKPITGYEHLITTAVYPFNIRSHPIKGLIYCNQTFNLVYSSILP
VFDGISVLLIFNCTHRLKILEHKFKLAKTSSDLSECVREHDDVSRTIKETNSIVRFLVFKTVC
SFTSNVIPGGLQILNNVALSQSICQVCIILLVYSRIVLCAECAGNMTDAGEDLLFTVYSTLW
YNEEPKIVSMKIFIIQKCQNIPAIHIKGIMSGLGRKYLLTIMYSTFSYLTTLRTVTSDEKS
>NvitOR58
MTIQSVLRRKVDVLLKAIALNKMCVSPKMILLVIKFAAMYLAIWPLDSSGKHWNTAFDCL
WWFYVVNNVLVIIPTLLAFYSSRRDIIAAMFSWLEILALLEALIILANFRYYRSRMQPILKE
AVDYIGSANSRRQLCLEKRASIITTTFGVIVALYIAGIIIYIYRPAVTEWDGMLTTAYYPASMR
SPFADVFIYITQLTALLHNGVLIVSDAFTVLLLYVCTVRLEVLQKNILRVADYDELKLWIRE
HERVLRLVTDTNMVVRINISKTVISFVGYSVGAGLQIISPTVTIVSFQRFALVIAMNAMRLF
FSATFADDLVNSSNSLINTIYSTIWYKDNRDMKIGKIIIMLRCQKLLRISVGGIMPVLGKPYL
TKILYTSVSYFMTFRAITGN
>NvitOR59
MSRNLYHDAKIKCKMNVITYLLKCLNFLRFMGKIYAVWPLKTDDNIRWRFVYECLWWFY
FLNYLVAASFTLNTCGHASDDITIASFSWLEFVSMVESIIILINYKCYHVTLQLLLTEVEDYL
TLADEKKQWVLKEKASIFAVMMCIITFLYFVLVVLYFTNPAITAWETFLTTSYYPPAIRSPV
MDVFLFSNQLIVMCHTSVIVNLDAMVVLLIYICSVRLKVLAADLESVNDDEELKQRIREH
QHILCLAKKTNIAVRLVVSKTVICFISYTVGAGLQLVNPTATVASLQRFGIVLLINYVRLIMN
ATSADELLTVSRNVGLSIYSTDWYGESKIVTSSKFIVMLRCQKLVRIHVDGVMPALTLTFIT
GIISTSISYYTTLRAVTRQN
>NvitOR60
MSRVLQSDSSSREGKHVWSKDAKFALMLNKFIVWPLGLWPLECDDAFSRFRNFYAVVSQ
VWMIGTQATAAYLGCGDVADTVDFVMMTACALMALSKIVTIRLHMSKVHTVFVSALDD
WLAVDVDKSRDVLIPFAKTGRFVFYLQMVSAYMSNTLIIIGALPFLIPPAANGTWANVSET
LQSRQLPMRTGCMFAGYRDEIYGSLYVYESVMIMITAHGNVGCDVLFFILAMHLCGQIEL
LKTDVLKIGEDEKVPGEWKNKIVECVHRHIRLLGMAKALNKVVSGVLVIQLLLNAGLNL
MLGIRMLIEIKRGSIFNAVRPMIGFNVLMLQLYLLSYASDRLSSQAESILDAVYDSYWYKLP
AKLRRDLYFVTMRANKPIYFMAGHFYAMNIENFMNILKASFSYFSILRIMFQA
>NvitOR61
MGGKSEVDEAFAVYRAFLWAIGVWPLEEKSFSQILRYIVAAVVQVTFLLHTFTEILLNNGK
VSDMVDVFFFSSAAFLTFAKHTYLHLHKDAIRENLRCYLDDWSNTKDEHFLRIMREHVKI
YKYQFHIYNLCGYVGTTLFMCRSILINILAKRQLGPGESYNYQFICQTSYLSQDTLAKYYPI
IMAIQYIQCMYCCTSGACTDCFFFGLVFHLCAQFEILKIKWERLGTKDFGVTAVHDRVKV
NALIARHKELVKLGENLESGFNNTILVQLMISIVLICMSGCSILVAIMRNDHVTMLISTNSIS
FMVTETLIYGYASDYLVTQSESIVQAVYSSSWYDMDSSVKKDIVFVMMRAKIPLHITAGKF
FCVTRNTIVQLLKTSVSYLSVLRLTLEMSHQEGQL
>NvitOR62
MCANIFIGHHQFGLRVVGSWPGKSQLPGFYFAIGIMLFFLIFEILNITEVYHDLEELMDNLV
STIGVVLGLFKFITVRVKRRKLKTVINKIFDDWKTDSQFVSEMMVKNCTRSQLVSKFVIFL

YNSMNFTYFLRTVISHIFDEVQDRKFLAQVTFPIVDGRQTPLYEIIIFFQFITASVCFNSQALV
EGLLATLVLHACSKVDVVRREILNFSTICKTDKNDKKDILKTLRKLSEEHFKFIEFSEDIQDI
FSYVSFFHIFFLTLIQVVSGYMFIDGLERGTKPVNLIHYAILTTSFLVSAGYYCIAGEYLTSQS
EIIFNELYNCYWYEFPSSYKKAICFMLLKARKPVKLTVGKFSTLSLIYLTSIMKTSFSYLSLV
RAVR
>NvitOR64
MSDKIVMRHVRVALQVIGLWPGYTSSVGFVIAITWLLTCLTFQLWHAAVVFSKLDALMGN
LGATMAVATATLKLIAFHVKGRNVKIVIKEILNDWAYENRSSNCEVMVQNTKRAKYLTKW
ITGAYNATVITYLVNAIIAYCSGITEQRLYVLPSKFPSFCKQSPVFEIVCFFQFSAALISTNVQ
VLVEGMLTVLVLHAGTKVFLLQKEIQKLSVICQSKTNNKEVISKSTIALINKHLNFIKFVKE
VKDIYYFISFVHVFTFTFLHVIVGYMFIDTLERGDRSIKLFLYGLFTTRALASTTIYCIAGEY
LMNQSMRIFDELYNSAWYEFDVPNIKAITFMIMKARNATSLTPASFGQLSLFYLTSVIRTSFS
ILSLTRATR
>NvitOR65
MSILSRHVKIGLYAIDAWPGVSSSGLFFLVMAYMTFSLIFQILNTTEMITQLDLLMNNLQTT
MPVILVVLKLSVFRVKCRSARLIIADMLSDWKCINETKERKVMMKNAKIAFYLSSTIAICY
NGLILSYLLKAILAYETENIYDRKYVMQATFPINAKSSPVFEMLCLFQFTVSVFAANGHAIL
EGLLTTSVLHANTKAFGVCQEITKFAKSCEANKSRKNIVEAKRRLIKRHLYFINFAEKIQET
YAYISFFHLFLMTLINCIVGYMFINLTINKDNISALLLCIAYMFTALSAVGSYCIAGEYLMSQ
GSLIFEKLYDCPWYKFKPVDTKTFIIMLMKSRHSVTITAGNFGDLSLVYFTNIIKTSVSYLSL
VRAATN
>NvitOR66
MIPIFNKPLECCLKVAGFWPYDFNMLGPVAITSMLVTTLPFQCWNAFALTENLVVLMDSLS
DIFTEVLIYIKIFILWNHRREIRDLLEEIGKDWSIKSIPTEWENIADYCRIICNIDVIVYASASIL
YYPDLLMSYFGKPVNERHMLFQSYYPFDYRRSPIYEVINIVYFFQGILMIIADSVSKTLFIS
MIFHVSSQIYELRNNLEQYSRHSNDGYENKNFKRLKLVVQQHLKILSLVRRIDHIYSYVAL
FQIVFSSIIICVTGFVIITAMESANIMLLVKFMTFIIAMLAQVSYFCFAGQYLLNKGESIVEMI
NSSFWYNSQCKDVKVLIFVLTNAQKPLTVSGANIFNLSAETFTMIVKTSASYLSVLRAMYT
Q
>NvitOR67
MILLINKPLEYSLKLSGFWPFEFNIIGSLALISTLVTTLPFQCWQAFNFTNDFVLLMDSLSDI
LAEVLIFLKLFAMWKSKSCITIILREIFDEWSTEKIPDEWKTLAYYSRMFCNIDTLVYFSAA
ASYYPDLLMSYFGKPIENRKMLFQSCYPFNYLGSPTYELINLMQMIQAVAMMAADSLSKT
LLVALILHVIANIDLLKNEIRIYSTNIANTCNHTNNKKSTVDLKQVISQHRKILYLVQSIDNA
YSYVSLFQIVFSTIIICVTGFVIVTAMESANIILLFKFILYIIVMLSQAFTFCIAGQYLRNEGESI
IHEIYDCLWYYTEPKEIKSLIFVLKSAQIPLTLGGGKLFELSTNSFTMIVKTSVSYLSVLRAV
CV
>NvitOR68
MKIPIIGIPLEYTLKLAGLWPDQSNILGSIVMGSALVTMIPFQVWDTINVSDNLVMVMDNL
SNILSEVLLYTNFIVLLLNKSYLDDLLREIADDYKNNIVTEKWLKLDQNSRRFCNYDYGM
YLGACCLFYLQFALMYTQMPSEDRIMLLKAYYPFDYKSSPVFEIMCFIQVIQGLLMCSIQA
LSESLLIALVSHVSGHIDLMNKQINVVSKSYDGQNSLTLKLVIKSHLKVLNLVNKIESVYTY
VSLTQVCLSTFIICVTGFVVLTMNSANEIVVMIKYIMLYFTLLWQSFSFCFAGQHLLNKSD
MIPYQVYDALWYKAEATEMKAILFIIKRAQTPLSLSAGKFIALSAQTFTLIIKTSFSYLSVLK

ASYA
>NvitOR69
MKIPMVYWPLEYTLRINGLWPGENNILGSIVTASGMVLILPFQVWDAIKTIDNPILLMDSL
SDIMTEIALYAKLIIMWFNRRYVVDVLKEISNDCNQNDVSQNWTLLNYNARRFCKYDYS
WYISATLLYYIQLVTMYIEVPVDGREMLLKSYYPFDYKSSPTYEIMLFLQIILAMSMAIANA
MTESLFIVLILHACSYVDLLLDEIKIFSDNCNKKVLNITDSNNMRFYVHVILKRHIQLLESV
KKIENIYSNVSLVQMFFSVITICVTGFVMITALESKDIVLLIKFATFIWFLLWQIFSFCFAGQY
LLNKGETITGAMYDSDWYNIESNDVKAISFIIKKTQRPLSVTAGKYIPLSVTSFAAIVKTSFS
YLSVLRASYVE
>NvitOR71
MYDEIFIRPHKISLKLIGAWPGYAKLTGFFLVIGSSSVLLFFALWNTIEVFGNLELLVDNLVN
VIGIIVGFFKLTTLRVKRRNLIFMVDTMFEDWQTSKKTIEELNAMKDHFERSKWLCKSIIM
LYNSLILTFLLKPVRSYMNDSIEGRQYLAPVSFPKFIDAKQSPIYEIVIIGEIGTAFFCINSHAL
VEGLLASTVLHASAKIAAVRQEIIRFSKVCRSQNSNKRLIISATRRLVQVHLSCNEFSETIVD
IFAVISFFSILLMTLAQVFSGYMFIFNIENGGETVQTLHYGFLTIVFLVSSGYFCIAGEHLANQ
SELLTMEIYNCFWSEFRIPEQKAIRFILAQSQRPVRLTLGKFDELNLVYLTKIIKTSFS
>NvitOR72
MDDEIFIRPYQISLKLVGAWPGCAKLSGFFFVTGWSSILLFFALWNTTEVYENLDFLVDNL
VNVIAVVVGLLKLTTLRVKRRTLMTILNKMLEDWQTMKMIEEFKAMTDNFERSKWICKSI
VMLYNSLILTFLLKPAISYMNDSVEHREYLAPVSFPKFMDAKQSPMYEIITAGEIVTTFLCL
NSHALIEGLLASSVLHACSKVDAVRQEIVKFSDVCRTQSGDKMLKLTAIRRLVNVHVNCD
EFSENVENIFTVISFFHISLLTLMQVLSGYMFILNLEEGGEILQTLHHGLIIIVILVSCGYYCIA
GEYLTNQNELLNVEIYNCFWTEFPVPQQKAIKFILAKSQRPVRLTFGKFDQLNLLCLTKIIK
TSFSYLSLVRQVH
>NvitOR76
MSTKKIASSIDSFLWPNRYTLEFLGFWPPEPGTSSISKYFAAFRIVFSILAIGFLFVPEIMMVV
VFWGDITVLTGVGCVSTTLAQLNFKMLYVLARRRRFCRAYRKTRELWSMTDHESELRKG
LEKLAGQAKKYSIAFFFTCFCNNISFTTLSVVVWLNYNAQENKSLLERRLPFDVWFGFDL
QRTPNFELVFVGQSISAIFCCFGIVGLDTAMMALILHVCGHFRVIGARLRAIGQGMHNDVQ
SKNSVEYLHTSPKLAIWQCIQYHQQMIKFAEEVRSLLSPIIFVQLLTSGLEICLSGYAVIVNS
DAGNYGDLVKCTGYFLSVFIQLIIWCWPGQILIQDSSEIGRIVLHDLPWWDMATEQQRQFV
FVIFRTQKECQITALGFQVMSMSKLTDVFNTAGSYLALLRRVYEKETEE
>NvitOR77
MAGRGSSVRIDEYLWPNRYLLELFGTWPTDYDGRTLASQLFVNFRVCFVFVAITGVLVPEI
LMIIVYWGDLDVLTGVGCIATPVSLILFKVAYMIIRRNRFHGVYSNLRRLWLAIDDAEEFEP
LEELARLAKRVTIGFFLSCFSNNVSFTTAAVIDWVNYDETRNDSTPRHLPFDVWFSFDVER
SPNFEIAFGCQVISSLYCCTGIVGIDATMMTFILHICGHFRTIAAKWRAIGSKILDNEKYSKS
GQVMPVKKDINQILRQHSEMLRIAEEVRRLLAPIIFMQLLTSGLGICLSVYAVTMNGSKGA
DLFKFIVFFVSIFVGLIIWCWPGQLLMQDSAALGDVVCYELPWHLLGVAEQRNLAFFIMR
AQKECQITALGFQVLSMNKFTEIFNSAGSYFALLRTIHEKQLEAQ
>NvitOR78
MARSFASFDEYTFLNRWGLTFLGIWKSDAEARGGPLRRFLHRLHVTILFTLLMLLLLPQW
MDMYVLWGNIDANAETFVLNVFTITALLKLWCFLSARQIFEVPENARAATEARVYFLKFEI
KQVIDTMKENWRRTMSGDEPGRKTHREILLDMAGKARDYTKRYGLLMYSTATMYFVSP

FVGMQRDNVRIRKYPFFGWYYFDRFSNLYYGICYASQVIIGIVVGTSNYAMDSIFLVAIYHT
CARLQMLQHDLKKIGEDRENRSPEEIVQLIRLHQREIRDAKRLTKIFNGSSLQQLLVSCVIIC
IIGFKLIIALNDGGFEFLVYVAFMFVALLQIFLYCRPGDELIVQSTAVGYAAYQSHWTSLEAE
SIRKIMFMILRSQTSLKMTAGNFYVLSLPNFTMILRMSMSFLSLLRAMYRKSDGFG
>NvitOR79
MRIGARRASRMESTTEASGIMREYDDCIFLNRLGLTMVGIWPLEHNASRLRIVLRRIHLGA
IYVLMLSVVIPQWFDIYCLWGNIDANTETFMSNVFMIAVMIKISNFLNSMRLFEDVLRTMR
LNWLDVMRLSSGELEKKEIMQGLSMKARSRGRVYGLVVVMTGAMYGLMPLIGSNKVAS
LRDRSYPFFGRYLFDRNSDTVYRLCYLSQLMSGSVTAVANFATDAIFLFCVYHFCAQLRIL
QTDLLKLGGPRFDSREALVQLIRRHQKEIRNVRALQSLFSISSLQQLFLSCLMICLNGFKLI
VSLCNREVDILMYIVCLPVTLFQILFYCQPGNELIVQSQSLDEAIQQSHWVNLDRLSKRQL
FFMIQRSQKPLAITAGKIYVLSLENFMRIVKTAMSALSVLQAMYRKTGS
>NvitOR80
MHCSYSFFLVTAFAMWRPRSWDDSKILTALYTLYSILSFTVYYTFLISQILDIVLLAENIQQIT
ENMIQLINVVNVSQKSLCFFLKRKKIIRFMDYFFEDMTLPQSPREKEIQKSFDDESKGNSQ
KLFVLYSVSVVMYVYMPFFISKREDRVLPYRAWRPYSLDNVNYYYLAYLHQSWSVTIAA
TGNAATETLVSGFMIQICAQFEILEHRFMQLPKILKEMRENGESESTVLATERSIIIKLIHHH
WRIFEMTELFNDIFVFVILSQFVTSITVLCVSTYNLALCKSVNNDFVTIFMYLLCMLLQIFM
YTWYGNEITLRSCDLGNRIFLSEWRSLNPPTVKNLLIIAQRTMKPIILSSGYVITLSNVAFTSI
VKTSYSVFNVLNV
>NvitOR81
MHILSLTFTFFKIYGFWRPLSWKSPTLGFLYDVYTFVMFMIVFTFALSQLMSIILTVQTVDE
FTSSSFILLSIVSACFKASNLLLKRKSLVRLLNVLISTTCKYQDDDEKMIQDMFDKKARRN
TVWYMALIQSSVFMITLQSIFINIPQKTLPFPAWLPYNYSNTRLYAISYTHQVIGNAASATLH
AANDALISGIMLQICAQLEILKHRILKLPTIVLKMNSGKEAPMNTVASKESELLGNIIKHHN
CIFQFSKDINDTFSMALFAQFFIAALVICSSVYELSKIVLLSSDFVALLSYLSCMLVQIFLYC
WYGTAVTMKSWSVGDTIFATDWSPLSMGLKKSLLIVMIRAKKPIELKTGKIFTLSILTFAKII
KASYSAFNFMQQA
>NvitOR82
MRVLPITFGILTVCGFWRPISLESSIPKQMYNCYSIFMCFLIYTFTLSHLIDIVISAADFESLTG
SCFMLLSMMNVCCKMTNILYFRKNIVELLQILASDHCTAKDVVERDIEKKFHKRARSVTL
CYWILTETTCMLITLRTFFGSSKQILPFKAWIPYEITGLAVYWTTFFHQTIAHVAAANLQIA
NETLICGLMIQACSQLEILKYRLKKIPDESKIDKFPLQSTVNNAQNTNKKDTKTLLVNCIDH
HRRIIEFSEKLNSTFNVILFVQFAISSLVLCSSVYLLSKMKLVSVHFMSLSLYLSCMLYQIFLF
CWYGNEVILQSLDLGNAVYHMDWTILSTEDKKKLLIVILLVRKPIQFTSSFLVSLSIESYCKI
LKTSYSVFNLLQRTSI
>NvitOR85
MRSLTFTFKVLSLCGIWLPLHWQSHRRLRLFYKIFSISTVVLTNIFILLQGLLLALSEFDWQF
LAEILFTLLTAFSVSFKATNFLMRRDKIICLADMLLKSWCIPRNAVEIEMESRINEFLRVFTIY
FNALAQLSLACLLIMPLVQDPDKRELPFRMWLPYDIRNQWNYWSTYVIEVGPMIVGILLN
VTTDVVVSGFVLQACIQLDMLKHRLNKLPNIVKVAKRKRLASEEVVRSFERKTLHQAAR
HHDYIIKYAKVVTETFDVVIVEQFFAGALIFSVIIYVLTIGKVPILQKLMSVGYLICMLGELF
AYCWFGNEITLKSLEFSDDIYKIDWMALSDSSNKKLIFIMMRATQPIIMSYGHLVILNIESFK
SILKITYTAFNILKESTSTT

>NvitOR86
MLELPYKLLILTGIWMPEDWTHKHQKLGWLIFSIISIGLVFMQFSSLVIFLMISKSCAQFFER
VFLIPAGVSSLQKIYIFITHRKELIDLGKMLLKDYCIPRNFEELSIQHRYEELIRVLTLVCFVL
VNITMMNLLVLPLVTNGENRTLPMNVWLPYPVDSDASYWLTYTHQTLGTLLLGTGAVGS
TLMINGFMHQVCCQFEILSSRFQKLPQIIKRLQLLKKPNHLIYEYEKKSMKQYVQHHLYIF
RVADTINDIFKSVIFQQFCISSIVVSASIFQLSTRPDKDMEFIMVFCYLICVLVEFLIYSWFGN
ELMLESLHFQTSVYQIDWTALSIGSGKDLVFIMMRASKPVIMYCGHFIILSLESYLGILKAS
YSVFNILRRSSN
>NvitOR87
MHILYLPFKLLTLTGIWMPEDWTQKQQKLIWVLYSMVSIGLVFMQLSSQIGYLMQSKTWA
QVNERLFFIPTGISSVHKIFIFIVHRKDLISLGNMLLKEYCIPRNAEELSIQERYNEIIRVLTLA
CAFLVNVTMMNLVTLPLVTSGDNRTLPMRVWLPYKVDSDMSYWLSYAHQTVGIVFVGT
GAVGSTLMINGFMYQVCCQFEILSSRFQNLPLIIEKFQSLKKPNQLIYRYEKRVMRQNIRHH
LYIFRFAEALNKIFKSVIFQQFCLSSIVVSVSIYQLSTRPEKDLEFIMVFFYLVCVLVEFLVYS
WFGNELMLESLNFQQTIYEIDWTSLSTRSSRDLVLIMMRASKPIIMYCGHFIVLSLESYIGIL
KVSYSVFNILRMSEE
>NvitOR88
MHTILQLPFKLMTLTGIWMPKEFTSQYEKQGWTLYSIASITLMAIQSLTLSITLILSENSEQF
FETLFIVPTGLQNLQKIYVVVAHRKKLMDLEKMFSNDYCIPRNVEELLIQRKYDENIRILTL
SCIILMNLTVANLIASPLFDAYFTTMNTRTLPMRIWLPYKMDLNIIFWLTFIQQSVGVIFVGY
CIISTTLMINGFMYHVCCQFRILSCRFKKLPQVIDYFRSLKKPYNVIYQYERRAIKQNVQH
HLCIFRIAENINDTFKSVIFQQFCISSIVVSASIFQLSTRQEIDMEFFMVLFYLICVLVDFYIYS
WFGNQLMLESLNFQRSIYEIDWTTLSTNAGKDLVFIMMRASKPILMYCGHFVVLSLESYV
GILKVSYSVLNLFRRSK
>NvitOR89
MEIIEQLEKMRILQVPFKVLTWSGVWMPEDWTQNQRKLKYNLFSFVCIGLMTIQSCSLTV
YLMMSKTWSQFVETLFLIPPGLSNLQKIFVIMLHRKKVIDLVNMFENGHCIPRTADEWSIQ
QRYDATIRVVTLVCFVLVNVTMVNMVTTPLFLKADERILPMKVWLPYSIETDFFYWLSYM
HQTLGVTLVGSGIIGSTLLINGFVYQVCCQFEILSSRLEKLPQIIRNLRSLKKSDHLVHQYEL
KLIKQIVQHHLYLFSIAETVNEIFKSVIFQQFCVSSIVVSASIFQLSTKPDTKTEFIMVLFYSIC
LLVELFIYCWFGNKLMFESLNFHQAVYDADWTVLSNESGKDLMFIMMRASKPIIMYCGH
FIVLSLETFLSILKVSYSVFNVLRRSHG
>NvitOR92
MQSLKVSFTILTYCGIWQPIYWTSGWHRTSFNFCRVVFRPLPYLLASAQLARIALVDMSFE
ELTEVIFILLSIVNICCKSVSILMRRADLIKLTKMLGIVSASPQDSDEFNIQHQYHQFIRYVTL
SSLVLVEITAITFLIPPFFQPENNRTLPFKIWLPYDYSMDKLFWITYFPESITIILASLISVSSNT
LIFGFLIEACGQFELLNHRFMTMPLYIEDFAKGEKITTYEVCKLEKQLLSRNIRHHTFIFEFV
DIFKKTFSSAIIGQYIVSSLVISTSVYQLSTNTTMDVVFFTNLLYLMCMLLEFFLYCWFGNE
LTVKSEDFGRKVFRTNWLALSTKSNKDIFVAMLRSSKPIIVSTGFFAVLSLESFMKIIKLSFS
AFNVLRTASDYQ
>NvitOR93
MHVLPESFMMFTCAGVWQPVHWSACDSRFLLYKLYTLFSIVLVYTLTISELMGAILLTQSL
EDFTDISFLLISTISVCCKIASIIARRDRVIHLTEMLLEVQCIPKNVRELEITRKFDKIARFTAL
SCIVLAEATVVVMSTGPLFQKAENRTLPFKSWLPYDSTTTPCTFWLSYVHQTAAIVLCATV

NVANDSLICGFMTHSCSQLELLNRRLLELPRAVKLKMKKLPRRLMCNVEAMIVSRHVKH
HVHIFKFAENINVIFTPVILVQFCMSSIVLSLSVYQLAVRSANGIQFITMVMYLTCMLVQFF
MYCWFGNEVTLKSVEFGQAIYNIEWTSLQVQTSKDLMIMMIRAKRPIIMSSGALVTLSIKS
FTSILKASYSTFNVLQRSSHN
>NvitOR94
MHVLPEAFNLATYIGLWEPTHLESSIARCFYKFYTCLSFALIILTMITQILAMLFFTKTLDEF
AETAYMLLSAINASVKGVVILLRRKHVIDLAEMLLKKECVPINATEKRVCSYFNKISRYTV
LSCIVLAEGTISALALLPVVFEQGELVLPLRAWYPYNAGSGLGYWLSYLHQAMALTLIAA
YDVANDTIITGFMVQACAQLELMTCRFHRFSWRGSNAVMRNGARHQLRLFEKRMVAQS
VRHHLLIFRFTEIINSIFAPVILVQFCLSSGVLCITVYQMSASKSNGLKVIVLSLYLVSMLVEF
FLYCWFGNEVTLKSLGFNIAVCEMDWTAMHVQTLKELLIIMVRSTSPIFLSCGPLIKLSLES
FTNILKISYSAFNVLKQFD
>NvitOR96
MLSIHFQVLTISGVWCPNHSSSVQRIFYKCYSFIVVVLMYSLALSQLARIIFVKQSFNEFND
TFFISLSTNFACFKAASNLVNQKQIVSLVNMFKHNCCLAHNDSERSIQKQYNDSCSKIIISLL
ILVETSAFFVVVAPLCGTMDNQDLPYQVLLPYDLSNKLFFWLTFVHHSFGAVLFTAISITND
AVITGFMMHVCGQLIILQHRFALLSRSLANEVSKKGRITDFDMMLERHWLRQIVYHQNHI
SNIAKKICSTFNEIVICQFFISGLEICVSVYQLSVRNNNTVELCTYAIYLMVMLGQFFVYCY
FGNEITLQSKITHRAIFDIDWTSFSLSLKKDLTLIMLYSSKPIAMSCGPFAHLTLESFTNILKT
SYSIFSVLKTAT
>NvitOR98
MNNQQMNEDLAPIPFRILKFCGWWRPLNMSTWRRAVYSCFTVIMLTLLVTITLTVLIGVT
QMSATDDLFADNVFLMFALINSVFKATNVLLSRRRFIKMLEIVQDTRWRDLRNDEEIEIQD
RYRKTIRKISVYFTTAVFVAIILRVVAPLLDLSDEIKLPVDAYCPCDIRHSSCYWTLYWHQAL
GTGVATLTHAAKDCLISALLLQTCAQLEILKNRLLSIADTCVVAGNKTGAADRVEKLEQKL
IGDCVRDHESIFEFAKILNDSLNVMLFGQIAVTIPNLCLSIYLLSTQKIASMDFMMTTQFFSA
VVIELFFFCWYGNEVTLNSLDVENAISEMDWTLLSTRSKKDLLMMMVRTSRPILFRVGPI
MNMNIDSFLSIMKTSYSAFSVLQSTGD
>NvitOR99
MKFQDSIEYQLLPIPFMVLTLCGTWCPENWSKKRKRIYKCVTTVLVSLGIILLVEMLVFIIV
KSGKDNIDLENIFATICIAVGLYKKINILYHRPKLMNFISNYTKNEWNKPKNFEEATIHLNIL
SETRYISYAYAAFILVSIIFRSITPILESGTFIILPLDACYPYNADNFIAFSLTYLHQIISGVTLTC
MHIGTDTLFVGLLLQMNYQLHILKNRLRQLGNSKTYKNNTQTIKDRELFIKSKISQRVREH
ESIFRFGYDLQKTFKPILMAQMVVVVPSVIINVYFLSIYTDRLNLKYFMTFFFALVSLMQIY
MFCWYGNEILLSSSDVGDALYESNWFALDQSTKKIMLTMITRSSKIFLISAVAIIPLDIDTFIK
IMKTSYSAFNLLQRTTAQ
>NvitOR100
MHEKLIAIQKANVEYELLPFQFLLLTIWGIWHPKDWPVRLKNISNIIFIVVFCLDIIICFEMSI
YLVLSIGTNDFKLVNIFFTSATITGIYKAIKTMQIRESFRTILLNYFNYEHLCSLNTKERMIRE
SNQAQIRKVTVIYSASMAGIFALNAIAPALSQPDSTMQLPVDAWYPYSIQKSLNYWLTYFH
QIILGSSLICVHIGTDTLFVGLLLKLVCQINILRYRLQSLTSLCSKNFEHFNAMGRKFIYRYIH
HQNEIYEFSKVLNNKFQAVLLIQVITSIPNLCINVYTLSKYSGIINMDYISIFFNTTSSLIQLFI
TCWYGNEVLLSSLQIKKSIYEMDWTKLDVPTKKLLIVIMARSLRPIAFSVAHVIPMNIESFI
KIIKISNSAFNVLQQT

>NvitOR101

MHLKFVGILRSNIEYELLPFQFVVLTVWNIWCPKDWPRRLKNTSIILFIVILILNFIMCTEMLIYFILSIGTEQFKLTNLFFVSASITGVYKSLKIMKNRKIIRCFVRNYFNHQWIKLLDDEENEIHEKINTRIRHITVTYFISMISIILMKDLGPIAESGLAIQLPADGWYPYDIENSVLFGITYVHQVILGSFVICAHVGIDTLFVGLLLKLLGQINILKHRLQILGNSLDHKMISLNKFESFQTVQKHLILECIHHHKRIYRFGEDLNKIFQEMLLILVVSSLPNICINIYALSSNLKNINMDYIATFFSTTSAFIQFFIACWFGNEVSLNSVEVRNAVYAMDWNKLDTPTQKLLIVVMARSLKPIEFSVGYIIPMNVDSFLKIIKASYTAFNLLQQTSSS

>NvitOR102

MHSTLAAIVKNNIEYKILPFQFFLLTFLGIWCPSNWSLKSKTAHNVYFTFIFFLDFLICIEMFIHFVSSFGTDNFKLINFFLVSANITAVYKSIRLMQNREVLRYFIISYFDYEWTKSHDSVEHEINSKIDLRIRRVTVIYSASMIGIVLLKAMSPIAESNGISLPVDAWYPYSIEKSRWFWITYLHQVILGSSAVGAHIGIDTLFVGLLLKTSGQIHLLNYRLRNLMLLKECNFAKLKEYSEKNVVLRCIYHHKRIYRFGGDLNDKFQEILFILVVSSLPNICINVYSLSSYKGNINVQYIATIFSTTSALLQFFIACWYGNETTFDSLQVINAVYEMDWTNFHVSTKRLLIFIMLRASKPLKFSVAYIIPMNLDSFIKIIKASYTAFNLLQQTTN

>NvitOR103

MDLSQCLEYRALPMQFYIFTLSGVWCPSNWTSLLKLSYNMYTTTIAISGILFWASMFVNLIITKNESEYFYENVFAISTLTYAMYKEFFVLKKRKEIQQMLKLSFDDEWYRPFDNREIQIIDHYAHETRWVTQVYAIGIIAGLATKAIMPMLNSNSAWVLPIEAWYPYNTSNLKNYLFAYTQQLMGGIPLICLHISVDSLFVGLILQMCIQLKLLQYRLQKTFSTDIDLQEEKNIERNIKISDVIIANYAFKHQCIFRLGNYLNQEFRGILAGQVMITIPNICINVYLLSQHRGGITLHLVDSFLCFTTCLMQIFLYCWYGNKIILLSIDVANTAYTTNWLSLNISSKKKLLTIMVRATRSIQFAAGTFIMNIDSFIEIIKTSYSAYRVLQKTS

>NvitOR105

MIIRKTLEHQVLPIPFHILTLWGIWCPEHVQPRLRRFYFAFTCIVIISEILLTTEVFINLIIIIRNKRFELDVFFILTSLMNGLYKALNILLTRKRIAKLITIGFEDRWRFPRDDSEKKILQNYKFESWRIHLIYAGACLAGVTIKLVGPMMKQNADIEFPAPAWYPYDTNKPVYFWLAYVQQMFVGGATISMHIGADTMLSGLMLQSCIQLKLLKHRFKHFFQHYEQVKGRLHLSSTKRKVEIALMKQYICDHQFVVSYANKINRNFSGWLIAVLIVVVPNICINVYLLSFSKIGLNVDFITSLGLFSISLFQIYLPCWYGNEVMLHSSEIANSIYDMDWVRLSPTARKTLIIVMIRSSKPIQIRAGYFVSMNLRSFLSIMKTSYSALSVLQQTT

>NvitOR106

MTTTTIIERTGSADVYGIENRLFSISFNVIKLSGFWRPTTFRKPFDYLYEMYTLFCLVGILMLIATIIVDNVVTEKSIRSLIENLYLILTVSNGISKLCNIYHRRDRVISMLQRSSEDRWSVHRDEEEARIVEESIESESYIIRFCIYLVTINNVSNALNPILNPDPEHDLMVDAYSPCDRSKSALCFWTAYLYQVFGYVSTSLVHVGCDCLVFNFVDRLCAHLKILEHRILQLPDLVEANACDEIRYLKSCIEDHHSICEGIKELNDTFYETIFIQFVTSISVLCTNIYLLSMQDLFSAEFIAVFVYLCCAFVQNFFYCWYGYKVSVNTLHISDAIFNMNWCILKRESKKILSYVMMKTSQKVFLFNSAVVTLTPESFVNILKVSYSAFNILQQTK

>NvitOR107

MEVMDTIKSTDIMPLPFFYLKLSGAWKPSSWPSYLRLIYDSYTILMTFFIMKVIIVTEILYVIFAEENQSKVLKDNVYIICTFINGWFKMFNLICRRKNIANLVKGCIAKQWNPPRDNYESSVLAATKQTSRKITLAHASVVGSCVVSTLLNSVLSSPPFLPVDAWYPCNITLPICFWTSFVHQSI

GYTVTAIVHVANDNIVVGFMMQICAQLNVLNRRLLLVHVEVEKAARQQKDQSQITSLETT
LVNDCIVNYRDILKFAEQLSETFIETIFIQFCAGLSVICTSVYVLTTLNIFSFEFFGMFLYLWC
MLGQMFLYCWFGNEVVLNSSKLFHSIYNMDWIKLQSQTQTKLLFMMLVASSPIQLFRGAI
IRVNLDAFINILKFSYSAFNILH
>NvitOR110
MDDDILCVRSLQINSVSSKIRKMKEMNPAKSVDVLSTSFLYFKLIGAWRPLNLPKWLRVIY
DLFTISMVILMYEMLIVTEILAIIFAEENRLKVFQDIVHITITHVSGCFKMLFVINRRQSIMLL
VNGCVAKQWYPPRNELEATILTKHNNLSRRITLTYATLVGASLLAAVLNPILYSTRVLTIAT
WYPCNISLPICYWSSYAHQTMGILAMAIAHVATDSLIVGFTIKICTQLNVLNQRLLSINFQL
ENTSARCQKSQEQSLALEAILVNECIVNYKDILRFADLLSRTFIEIVFIQFCVGLTVICSTVYL
LAKLSIFSYDFFGLFLYLGCMLMQMFLFCWYGNEVVLDSTKLFHTIYNINWIELQIQTQSK
LLLMMLVASSPIQLFRGAIIKVNLDAFINILKFSYSAFNLLQKSS
>NvitOR111
MDEIIDPIKRTDVLPISFLYLKLVGAWKPLDLPKCLRLIYDLFTIFMVIFICKLLIISDILCVVF
AEENRFAVFKGIVHVTITHLSGWIKMLHVLSRRRSIMLLVNGCVAKQWNPPRDRHEASILT
SFDNSSRRTTIAYTIQVSAAVSMLVLSPVFSSTWFLPIDNWYPCNISSPICFWPSYVHQSMGI
VAIAVAHVATDTLIVGFMIQICTQLNILNHRLLSIHIKLEDTARRQKNQEQISAVETLLVNECI
SHYIDILKFADLLSKTFVEVVFIQFCVGFSVICSIVYLLAILSILSFDFFGTLFYLGGMLSQMF
IYCWYGNEVVLNSTKLFHTIYNMNWVAFQIKTQKKLLLMMLVALSPIQLFEGAIIKVNLD
AFINVLKFSYSAFNILQKSS
>NvitOR113
MMSEIFPTTYFFLKISGFWRPYSLKLPLYLCYQIYTAFSFATVLSLIILLVLYCAFAHDKFLEL
LLENMYLVISFSNCISKTSNIILRKKNVEKLLQWIREKRWLAERDLEESCIVAHSKLMEKAI
PQFCTLLVCANGIGNLMNPIIRANPDKKLVIEAYPICDRSRPVCFWLTYLHQCFGFVIVNVI
HVACDCLIYNFIDRTCAHLKILGHRLQKLPVLVKGIRHQGIDTVEFEKSYVIDCIKNHQGIFI
FIRELNDTFCETVFFQFLSSILVLCTNIFLLSKQELFSPEFIAVFSYFCCVLAQNFFYCWYGY
KLSVNSLAFVDAIAKINWVELDMKTKKMLVYMMLITSNKVELFNNAVVNLSPASFINIVK
ISYSAFTVLQRTSHKEMKI
>NvitOR114
MLRTEELLANSRANERRNERVGIEDQVFPATFLLLKAAGVWTPTTLKLRSQYMCYRIYSA
FCFISVLALVVTVSIENVVSSNASILESWYMLVIFSHGLLKIKNLQWRRVKVIHLLKECIMN
ERWSIARNQDERAIINESKRAEKFITHLWLSLLLVNGLGNALNPLIHENPNNSLIFECYSPC
DRSLPSCFWTAYAYQLFGYAISSTVHVGCDCLIFNFIERINAHTMIFIDRLQKLPSRVVEGKN
EGCLDASRHEARLLKECIQDHRRIYESVEELNNTFYEVVTIQFLTTISIVCTNIYFLSKQELF
SADFIGVLVFLVCVLTQNFIFCWYGYKLSESSSYIVNAIFNMDWLVLNKRSKGLLLFAMMS
ASNEIKIFHNALVNLSPETFLQFVKMSYSAFNLLQQSN
>NvitOR115
MHQAIKIANGRIDADGIPNVEGLEKRVFPRTFLLLIVGGMWAPTTIKSRALFACYQLYTVF
CFVSVCMLIITILIDNVLSDDKTMESLVEYAYMLIVFSNGLVRIINLVSRRDKILRLLQGNIM
LDRWQSLRDDEELAIIAESKVSEKLVLKIWGSLILMNGISNAVNPIIHENPDNTLMFECYSP
CDRSVSYCFWMTYSYQLFGYVIMSVAHLGVDCLIYNIIDQINSHYKIFLNRLLKLPARVRE
KARDDVAAALRYENNYIKECVADHHSIYKAAGELNDIFNELVFIQFISCISLLCTNIYFLSKQ
ELFSPPFIAVFAFLCCALTQNFFFCLFGNKLSQTGSEIAGAIFGMDWQELQKETRRKLLFIM
LLTSKGIALFNNAVVNLSPETFLKLVKVSYSAFNLLNQSTHK

>NvitOR117
MDLLPVHFRTFQFFGLWYNDPCSYRLIKLVHRSLIVLLIVHLSLFQMIALFSAKRNVDEYT
NTLFLALTYFVHIYKTLVFMAKNRSVNEMLDEFRSDICRTRGPEEEHILAKHVQRANWAY
SGRMILTLFAGSIRVVLPILIGFSTGKLELLPFDTYFFNVKHLVQYALVYVLQTLAIITVIVTD
VCLDSTPCACMILACAQLEICRHRIKHDNMVLYENSEDGPGRGFNEEMALKEYVKHYVLI
QEAVHRIQSVFIAIVLPIFSSALLTLCTSIFQLAQKNHTTGEYCFIILYLCCLLVQTFSLCWFG
NELQSKGEIVTSAVYETDWTVLKPCLKKSYRYLMFMGQNKFIISFHGQCTLTLQTFIWMIK
TSYGAFNLLKQVADT
>NvitOR118
MDLLPMHFRTFRFFGLWYDDPRSYGLAKLVHRSLVVILIVHMSLFQMIALFSVKHSVDEY
TNTLFIALTYFVNIYKILAFMAKNRSVNEMLDKFRLDVCRTRDAEEERILAQYLHTANWT
YSARMILTLCTGVIQIVVPFLIGYFTGKVGLLPFDTFFFNVEDLAQYALVYALQAVAIVTVVI
TDVTLDSTPCACMILACAQLEICRYRIKHDNNVIANEVTGDGEMNSECKPGKELREKMAL
KKYVKHYVLMREIVDRIQSVFISIVLPIFCSALLTLCFSTFQLAQKNQTTGEYCFIITYLCCL
LVQIFCLCWFGNELQFKGEIVSNAVYETDWTVMKPRIKKSYWYLMFMGQNKFIISFHGQC
TLTLQTFIWMIKASYGAFNLLNQVADTKY
>NvitOR119
MDLLPMHFRTFQFFGLWYSDWRSYRFFKLVHRSLELLLIVHVCLLQIIALFSVKHSVEEYT
NSLFIGLTHFANIYKTVVFMVKNQSINEMLDKFRLDICRARGREEEQILAKYLHKANWTY
SARMILTLFCGSISIVVPILVGIFTGKLELLPLDTYFFNVNDLKQWTLAYVLQSLTVITVVVT
DVCLDSTPCAFMILACAQLEICRHRIKHDNMASHEIGKDVPRKGYKEEMALKEYVKHYV
LIREVVYQIQSVFISIILPIFCSALLTLCTSIFELAQVIVFLFNYHTTGEYCFIMSYLCCLLVQIF
CLCWFGNELQLKGEIVSNAIYETDWTVMKPCTKRDYWYLMFMGQNKFIISFHGQCTLTL
QTFIWMIKTSYGAFNLLTQVADT
>NvitOR122
MDVLPLNFRTLWLCGIWHEENEKLTVPRIAYRFLVICLMFYFTFTLSAVVFVENSNVSELTE
AIFLAVTYITLCLKIVNFAFRRAEMIEILHDFRHPYCKAEHSEESEILKGYSKQARKMYIYL
MAFVMSDVAYFWSTFAFKVSKNIMELPYHTYQFYNMSSKAILFSTAALQATSVLYSVSINI
SFDTMTAGLLILTTGQLELNAHRLSKLGEHNVDSMNGYIAHNVLINGTVDKIESFIKTVVIP
FLFFSLLSICASVFQLSEYSVFSLEFLGLFSFAICILLQVLVYCWFGNELMLKSEAVTDAIYR
SDWTMLSPQNRKSLQVMMICNKDGRTVSFGGQCSLTLETFVWILKTSYATISLLNRVSA
>NvitOR124
MDILPLNFRILRYCGIWYELPEHLWLVKIVYKIFVVVVIFSFTLSELIELALTYDDLQNLTEC
LFLTLTFLALCFKMINFMCRQESLKALLNTFRDEICQPKTLEEKDIIEKNRSMLRLFCISYFS
LGILSGSTLVFVPFASFKSSKIELPIKTYQPYDVEDFVLFSLTYFHQILSMYLGVLINVSLDM
LVCGFIFLTCGQLDLCYYRIVSSNMYTMNNNIRHHAVTKDIVKKMQSFSIVVVVVPLFIFSLI
TLCTSLFLMPEKEIMSFEFITLFIYLTCMLTQIFLYCWFGNELQLKSKTISDAVYHSNWTRLT
PKLRRNLLFTMFISQNGLMISFHGQCSLSINTYVSILKTSYAAFNLLRKTSNTLGV
>NvitOR125
MDMLPSFRVLAYLGIWIEEGSSFVFLRRLCGLFLSNTIFYFTLTEVIELYLLRNNIEELVDVM
FLTVTFAMLCLKILNFNFRHKGLLNLLTDFRMDVCKARSPEEENILNKYTTKILNIFQNILV
LSQATGIFFCVLPFITLEPADYEIPYKTYQFYDDTTAMGFTITCVIQFIALIFGIFINVSMDTMI
YGFIILSTGQFELISYRINKSSKENDRALLKQCIMHHNCMNNLVKKTTNLFMTVIAPLFFFS
LLTLCASIFQMSQNDIISLEFLGFAMYLSCMLCQVFLYCWYGNELKLKSADLVNEVFGSD

WTVLEYTEKKTLYLLMLSAQRPCDISWRGQCTLSLETFVWIMKTSYTAFNLLQRASDK
>NvitOR126
MDVLPLNFRSLQYCGIWYEFPEHLWLIKTVYRTFIVVVIFSFTLSELIELALTYDDLQNLTE
CLFLALTFLALCFKMINFMCRQESLKALLNTFRDEICQPKTLEEKGILAKYQNILKKVFIFY
MSLGLMSGSSLLIVPLVSMENSRISTLPMKAYQPYDVEDTILLNITYFYQVFSTWIGIIINVS
LDLMVCGFIILICGQLDLCCYRILCTKTKMFHDNNVRHHAVIAEVVRRVKSFFIVVIVPLFI
FSLITLCTSLFQMPEKEVLSLEFFSLFMYLSCMLFQIFIYCWFGNELQLKSKTIVDAVYQSD
WTDLTPKLRRHLLFTMFISQNGLTISFHGQCSLSINTYVSILKTSYGVFNLLQKTSNI
>NvitOR128
MDVLHLNFRVLQYCGIWYEYPENLWLVKMVYKTLIVVMLFCFTLSELTELVLNRNNVHD
LTECLFLSLTFLTCCFKMINFLCRQEGLNRILNAYRADVFQPKTTEEKQMTTQYQNLISKFF
MVYLIMALLSGICLSLIPIISSASNETQFPAKSYQPYNTQDSTLYLITYFHQILSIFFGIFINVS
MDMLVCGFIILACCQLDLCCYRISLNKKDTSTNDHVVHHVLIGHAVNRVQSFFIVIIVLLFI
FNLIVLCTSLFQIPQKNIMTLEFFTLFVYLVGILFQVYVYCWFGNQLQLKSKTISDAIYESN
WPDLTPCKRKDYIFSMFMSQNGFTISFHGQCSLSIKTYVWIVKTSYAVYNLLQNTST
>NvitOR129
MEIYDSRYFVHAKRFQELLGIWPYQSRLKNNCSWVILSFLFIAMIIPQIVGLSVHAGKDSK
RTLECTFGTCYMLAIYMKLLVACADKDKAKFIFEYTARNFKKINDNDERKILIEYSERGRLI
GVVYTIFVLAALGVFVVVPLAPGILDVILPLENGTRSKFFILNGEFLVDKTEYFIEIYAFDSIC
CIVTVLIICATDPLYAAILEHCLAIFAIVKLRLRKYRVRKGLKCVSADEAEYEAIIRAVQLHR
EIIAFIETIQNNCSLYFAFEMGVTLISFTVNFVLAVLKTPDLFDRLRLAMVLFAQAVHLFYIT
WPGQKLIDHGEDLFKETYFNDWYKSSVKCQKALRFMSLRCSKPCKLSGAGVYVLNFAYT
LLILKTSASYITVVAQFEYKIVA
>NvitOR130
MEIYDSRYFVFNKRFQMALGIWPYQSRVKNSITYAGLVLVMIIMLIPQFIRLNTYLGKDIEK
TMENIFIFFYVFGIFVKLFTAHFAEDKLKILYESTAKNFETYTDAVEAEIMKRYSERGRLLTF
VFLLYMISAVAVSVVLPMCPIVLDSTDPLDQPRPRMFILNGEYIVDKYEYYFQIYTLDIISVF
LMICILCATDPMYAAIVEHCLGLFSICKYRLRNFNKSCGLQMVEHAEAERYGGDYAYAAL
VRAILLHKDIIKFTEIIQTSYSLYFLLEMGATIGILTSSSVVVVMKLKQPLELLRWSLFLFGVI
LHIFFLTWPGQKLIDFSSDIFQEAYLNDWYKSSLKCQNLLKFMSLRCSRPCELSGGGLYIM
NFINFATILKTSASYITVFSSV
>NvitOR132
MEIYDSRYFVYNKRFQTALGVWPYQSRVKNAIICGFLLLVMIALVVPQIIRLKMYIGKDKD
KSMENVFGLFYIFAIYVKLFTAVYAEDRLKILYESTARNFQIYTDKMEKKILHENSERGRLI
TLVFIMYMMTALIVFILLPMYPIMTDVIVPLDHPRARMFILNGDYLVDRDEYYFQIYVFEST
SAALTVFILCSTDPMYAAIVEHCLGLFCICKYRLNNFNKPRRTEIIEKANAESQVDEYAYTA
LVEAIQLHKNILKYTKIIQTSYSLYFLLEMGATMGLLTSTSIIIVMKLYRPLDCIRYFLVLIGL
LLHIFFLSWPGQKLINVSGDIFQDTYHNDWYESSLRCQRLLRFMSLNCSKPCQLSGGGLY
VMNFVNFARILKTSASYITVFSSF
>NvitOR133
MEIYDSRYFIHNKRFQKALGVWPYQSRAKNIVVCGLLLLLMLGMLLPQIVRLKKYAGKD
SDKMMENIFILFYIFGIYIKLFTAVYAENRLKVLYESTAKNFQIYTGEAERRILYEYSERGRL
LTLAFIVYMLPAVTVYVMLPMCPIIMDAAKPLDHPRYRMFILNGDYLVDEYDYYFYIYAF
DSMAAIVTVAIMCATDPMYAAIVEHCLGLFSICKLRLKNFNKPNGTKAIEKTYYYSETCGD

EYAYAALVKVVQLHKDIFKYTEIMQASYSLYFLLEMGVTMGVVVCNSVIIVMKLSQPLEL
VRWSLVLIGGLLHIFFLTWPGQKLINFSGDIFQDTYLNDWYESSLRCQKLLKFMSLRCLKP
CELSGGGLYVMNFINFATILKTSASYITVFSSF
>NvitOR134
MEIYDSKYFIHNKRFQMALGVWPYQNRVKNLSICGVLLLVMFGMLIPQLLRLRTYLGKDI
DKSMENIFILLYTFGIYIKLFTAHIAENKMKILYESTAKNFETYTDEAEKKIMKQYSERGRLI
TLAFLIYMVLALILFVMLPLYPIIMDATIPLDLPRPRISVLNGDYLVDENDYYFQIYVFDSIA
CTLTVFIMCSTDPMYAAIVEHCLGLFSICKYRLKNFNKSCGMRMVERADAERYGGDYAYA
ALVRAILLHKEILKYTQIIQTSYSLYFLLEMGVTVGILTATSVIIVMKLERPLDCLRYFLVFIG
LLVHIFYLTWPGQKLIDFSGDIFQDTYLNDWYKSSLKCQNLLRFMSLRCSRPCELSGCGLY
VMNLINFAAILKSSASYITVFSSV
>NvitOR135
MELFDSRYFIINNTCMKLLGIWPYSSHVKNYLRRCGLGLFLLSCYLPQFIPLYMYFGEDMD
QMIQNIGVILYVFGTSVKLITGVTAKDRMKIVYEKTARDFQTIVDKEERNILFEYSERGRTL
SITFIIYMWIALAIYVGLPMGPLVLDYFIPLQNGSRERGFVWKGEYLVDPDKYYLTIYAVEL
FSSVLSVTILSSVGPMYQAIVEHCLGLFVIVKFRLQICTRGGKKAEEESYRLIVKIIRLHNDII
EFTRIIEASYTSYFFIEMDITISLVTLISVNLISRLDYLFDSIRHIFILLGVMIHMFYLTWPSQK
MINHSTDLFHDTYSNEWYNCSIRCQNLLKFVALRCVEPSQLTARGLYVMNFENYASLVKT
SASYITVLLSFR
>NvitOR137
MDIFDRRYFVLNKALLRSTGLWPYEDRRKKLYIRTFVNLILGICVIFPQIVRIYNYFGVNMN
MVLEHAAVLMYITSIYLKFLTSVYYEEKLRVVYDNIAKNWQVIKDENEINILIQYSENGRL
LTIGYTMYIIAAFCSYVFLPIVPVLLDVFNPLNQTRSRFYILGGEYFIINNVEDYGKVYAFDC
LAVIVTVWLISAVDSMYAASIEHCLGLFAIVKLRLQTCTRSICDGQKDECYKMIVRLIRMH
KDIINFTDILESSYSSSFLILVGINVIFLSFECIIVLTRFGQAMEMMRYSMIMVGIVVHLFYIS
WPGQKLIDFSLGLFQDAYLNEWYTCPTRAQKLLGLMTLRCSKPCQLTAGGMYVMNFSNF
AKIVKTSMSYMTVLASFR
>NvitOR139
MEIFDQHYFSVNKALLKSTGLWPYESRRRKFCIRTFINLILGVFVIFPQLVRIYNYFGVNMD
MVLEHAAILLFILTTYLKFLTSVYYEEKLKVVYDNIAKNWQAIKDENEVNILSQYSESGWF
LTISYIMYIVIAASAYSLLPMAPVLLDMIDPLNETRPRLYILGGEYFIVDNVEDYGKVYAFEL
VPAAVTVWLICAVDSMYAASIEHCLGLLAIVKLRLQMCTQPSCDSRKDVSYRLIVQLIRLH
KDIINFTDILESSYSSSFLILVGVNVLFLSFECIIVLTRFGQTMELIRYSMIMVGIVVHLFYLS
WPGQKLTDLSIGLFQDAYLNEWYTCSTRAQKLLNLMILRCSKPCQLTAGGIYVMNFSNFA
KIVKTSMSYMTVFASFR
>NvitOR140
MEIYDSRYFIINKTLMTKMGLWPYQHPLKKFLVRTFLVVFIFVSSMPQLYGLKKNFGVHM
DKIIEHLALLMYIYGIKLKLVTSILSEKKLKKVYENIMENWQQIKDVHERAILVEYSERGRT
LTIGYIMYMTSALLFFIILPITPMVLNVIKPLNESRPWDFIMHGEFPVNDMHAHYGEIYLFD
SLACIATVLVFCTVDSMYATCIEHCIGLFAIVKSRLDLSTKFVNRQGALGIKRDDKVYDLIV
KTIKLHKKIINFTHILESSYSTSFLILMGMNMLYCSLVSVLLIIKSDALMERIRYGTILLGLLI
HLFYISWPGQKIIDLSTGLFEDAYSNEWYETSIRSQNLLKFMRLRCLTPCQLTAGGIYVMNF
ANFASIIKTSTSYITVFASFT
>NvitOR141

MDIYDTYVLNINKKLLSFVGIWPYEEKKKNKFTRVFYLITMFCIIVPQMIGFYQHFGVDID
ELLENTGTIFFTLSIYTKLFTSIIFENKLKILYDSVAKNWKNITEKHEREILVKYSERGRMLTL
GYITYNFAAVIVYTTMPLMPFLLDIILPLNESRPSMFILNGQFYVDKHEHYKKLYAFDCLCI
FVIVPAALAVDTMYVACTEHCLGLFAIIKYRLAMSDKFISTRDIYLTEEKDSSYRWMIHTIR
MHIDILKFANILDKSYSSSFVILMLINTVYYVSVLCVLVLISLDKPLNLIRYYMLLVAICIHLFY
LSWPGQKLIDHSEGLFRDAYNNQWYEGSAKSKTLLKILTLRCVEPCLITAGGLVTMNFATY
LTIMKKSVSFITVFSSFR
>NvitOR142
MTMDFYNSRYFSINRRMMTIMGLWPYQDFKTKLFIRTFLAIVLGIALIPQIISIVKYTNEDS
DKVIQGIATLLYVTGITLKILTTITSEKKIEIVYRNIVDNWKLLDDENEIRTMTEYSEFGRLLT
IGYVAYMFFALGLFVTMPMLPMMIDVISPINGSRPRIFILDGEYIADKNENYGKVYIFESLT
CIMSVFVFSTVDSTYAVCVEQCVGLMAVVRLRLKLATAKAARMKYKSDSDEHDIPYQLVS
SSAKLHIKAISFARILDSSYSVNFLLSMGSNVMILSVGSVVILINLGRPMEFIRYSMIFIGLMI
HMFYLSWPGQKVIDSSQGILYDAYNNEWYECSKKTKTLLKFMMLRCIEPCQLTAGGLYV
MNIANFGSLAKTSMSYITVFASFR
>NvitOR143
MDFLDSRYFILNKKMLHILGIWPYQKRLERYAIRSVYFFFMGVSFVPQILCVKKYFKVDSD
KFIRGVTTLLYLSGVSLKLTIAILMNGKIQIVYSKVADNWKMFTDKDEIKTLLEYSEVGRM
LTLGYVVYMVLAVIVFITMPYLPVVIDIVFPINGTRPRLFVLDGEYIVDKYENYNKIYIFES
VCSVVSVPIFCTIDSTYAVCVQQCVALLAIVKLRLKVATKYTKNYLRDHKYNDASQQLIIK
SADLHNKVIEFAQILETSYSMVFLLLMGMNCLILSVGTLVILVNLNNPLELSRYIMIFIGLM
MHMFYVSYPGQQLIDRSSAIFNDAYNNEWYECSIKSQRLLAFMMLRCTKPCELTAGGIYT
MNLENFGSLVKTSISYIAVFASFT
>NvitOR145
MDKLKQSTVDIDTINNIFGNTYFKINKELQELVGLWPYQKGFSVRVVQTIMLFVLSFIMIPH
LNGIRVWCGKDLGICSENIAATIYLSGCFLKYLVVLLCKRDISKVYEKIAINWLTINDPNER
VILDKFSSLGKLKSIGYTVYVSAAGIGFSQFALLPFAFDYFSPLQNGSRPKIRIVRAEFFVDPI
EYYWHIYATYCIVTFVSAFTIISIDTSYTAVVHQNLGIFNIVKYRLSLAKKAVGTSKDLAYE
QIISAVRLHQDSLGFNNLIEVTYRVCFLLLILVCISFLTFGAITILENSDNWIDIVRLGSIEVGA
VIHLFYLSWPGQLVVSESEELYYYTYNNEWYNLSAESKTLLHFMMLRCINPCCLTAAGLY
VMNFENYGAIIKSTVSYITVLSSFRE
>NvitOR146
MNGFETSSLNSKIINEVFDNTYFKINKILQELIGIWPYQKRFDALIKQFIVILILSMVAMPHIN
GIRVWCGKDLGLCAENLAGVTYASGVCTKYFVVTRSKDQMTKVYEKITSNWLTITDPDE
RVILNKFALLGKYKSIGYVGYITVAAICFSQLGLLPILIDVVLPLQNGTREKLRVVKAEFGV
DPYDYYWHIYGAYCAISVVSSGVLMAIDTSYTAVVHQNLAIFNIVKYRLTQAKRAVNTVK
DVAYEQIISAIRLHQDSLEFNNLIEHTYDVSFLILILICVTFLTFGAITIMEESDSYLDMFRLSL
LECGVCIHLFYLSWPGQLVVTESEDLYYYTYNNEWYNLSEKSKTLLKFMMLRCMKPCCL
TAAGLYVMNFENYGAIIKSTVSYITVVASFRED
>NvitOR147
MTNEISQEKLNRVFDSHYFYLNKKLQIVSGLWPYQSRKRKFIHKLTMLCFLGTALVFLLNG
LRHWCGVDIDVCGENLVGLIYVISVLSKLFITSLYEEKFKIIYTRLAINWLELTDPQEHNILIS
FARQAKIKTVVYFVYMAAAGVGFCQIPMIPVFLDFINPLNETRPKILFVKAEFIFDPYKYFY
QLYAFFIGCAASAVFIVCSIDTTFTAVVHQIIGVVSIIKYRLNCATVSFNPNKDVSYKLIVHAI

QLHKEVLQFSDLIEKSYNIFFLVLTGLTVVFLSTGAIVMLVRVGAMLDLIRLVLVLIGAILHF
LFLTWPGQNLIDHTSDLFAAIYATEWYNVSERSKKLLSIIMLRSLKPCVFTAGGLYVMNLE
NFGSIMKTAVSYMAVVSSFR
>NvitOR149
MNKEEVDEAFNDSLLKINKELNIFNGLWPHRPDGDKLFRRIIVLTVLISVTLPHVLGMFIQC
GRNMALCGENICGFCYCSGVIAKFIVPIVSKEKFITLYEKIALNWKEITDPYEQSILEEFSKL
GRLKSWLYFVYCAVAGFAFCQMTALPALMDIILPLNESRPKILVTKAEYPFDPFEYYYELYF
LYCTAAVVSVSVLASTDSTYSVIIHQSLGIFGIVNHRLQKAAKHKNQEESYRVMVSAIELH
KSALEFLELIESTYQSAFLIFIFVTVAFLSFGSLIIVEHSEEIIDLIRMTLIEFGAMIHIFFISWPG
QLVIDHSENLFLSTYTTKWYNMSKKGKMLLLFMMMRCLKPSFLTAGGFYIMNFENYGSI
VKTTLSYVTVALSFH
>NvitOR151
MDEREIDLLYDNYYFKLNKKLQIITGLWPYKSRKYKLGIRAVVYATLSLVMIPLCNGFRTW
CGVNLDICGENLVGICYTMLIFLKYWVTTHSEERLKNVYRLVAKNWMEITDPHEHEILVD
YAKQGRLKTIGYTAYVVVAGIGFCQIPMISVILDIIIPLNVSRTKILFKGGEFILDPYQHFYKL
YVYFVITSFVIMTIIIAIDTNYTIIIHQILGLLTIVKHRLQRLAIPMNLKKDNSYHAIIKAIHLH
NDALQFVDLIESSYSCLFLVFIGFTIIIISISTSIMMAQIGKLLNMIRVAMFVLGASLHFLYINW
TGQQMIDHSKELYLNVYSNEWYNLTKEAKTLLKIVMLRCLKPSKFTAGGLYTLNLESFGT
IMESALTYVAIMSSFR
>NvitOR153
MNKKEVDKAFNDSWLQINKELNVFNGLWPNRPNGDKIFRRFIVLTVLITVTLPHVLGIVM
QCGSNMALCGENVCGFCYCSGVIAKFIVPIVSKKKFVTLYEKIALNWKDITDPYEQSILEE
FSKLGRLKTWFYFVYCIVAGFAFCQMTALPALMDIIQPLNESRPKILVTKAEYPFNPFDYYY
ELYFIYCTAAVVSVSVLVSTDSTYSVIIHQSLGIFSIVNHRLQKAAQHKNPDESYRVMVSAI
KLHKSALQFLELIESTYQSVFLMFIFVTVAFLSFGSLIIVEHSEEIIDLIRMTMMELGAMIHIF
FISWPGQLVIDHSENLFLSTYTTKWYNMSMKGKILLQFMMMRCFKPSFLTAGGFYIMNFE
NYGSIVKTTLSYVTVALSFH
>NvitOR154
MDIYDSRYYKTNIFYLKLLGLWPFDDFLNKRVRRILIIIAVVSLIIPQVIRLFEEWGRDIDIVIE
VIGSLIYFSGCQIKYLSFLRVEAKMKYLYNKIAEHWKSLSSKDEIKTLEEYGEIGRGLTLGY
IIPINIILVIYISLPLLPLLLDVIDPQNETRPKQFPYFAEYFIDDQKYYFELTIHGWIVCILSVQI
YGTFDTTYTQCVQHACGLFGIVEQRLRKATKLASSNAFSTQEEKDEKVYDKVIDAILLHK
EAIQFVNLIEDCYSFSYFFVVTLNTAVVSLAAVDTMLNLENGNTKQMVRIGALYIGFSFHL
LYNMSPGQRVIDSSTNIQNAAFHCDWFNASSKTKTLIRIIMLRSLTPCQFTAGKLIVLHLESF
AFVFKNSISYVTVVGSMR
>NvitOR156
MSESKTLKIFESDYYRTYKNSVKLIGLWPHENIHKKRITRFFITALLTTFMILQGIRLYEELG
NDIDIVLELIGSIAYFSGCICKYLTTIKAQAALQFLYEQIQGHWDTITNKRERQILEQSASES
QFLSKFYMGASYVALVVYTASPVLVPIILDIALPLNESRRKTFPYFIEYFIDTEFYYYQLMV
HGTICFTISVLVYISIDTMYAACCQHLCGLFDIVEHRLKEAVKTNSNRINLEPDRTDILMHK
LLNEAITLHQDSIEFAVLIENTYALCYLLVLGLNLAVIVLAAVDIVINLDDTNQIIRLSILYIAF
SFHLFFNSVPGQKIHDKSVNVMNSAYFSEWYNLPLNARKLIQLIIHRSLNPCQFTAGGLFV
LNIENFGSIMKSSMSYITVLASIR
>NvitOR157

MDIFNSRFYRTNCFFLKLLGLWPLGDVSNNRIKRVTVVSLVVSLIIPMVIKLVQEWGNDIDI
VIEVIGSLIYLSGSQLKYISCASVQSQIKFLYTEIERHWNTLTNEEEKKILKQYARDGYNLSF
GYLMLLNVILVGYLLVPFTPMLLDLIDPLNETRPKAFPYFAEYFIDNQKYYFELTVHGWIIC
ILSVQIYGTFDATYTQLVQHSCALFAIVEYRLGQATKMVASDEDSSHKDTDKVAYNMMVG
AINYHKQAIQFVGLIEKCYSLLSFLIIILNTAVVSLAAVVTMLHIEKGNQKQAIRIGMLYVAF
SFHLLYNSYPGQKVIDSSTRIQEAAFHCEWFNTSSKTKQLIKIIMLRSMVPCTLTAKTLVVL
DLESFAFVFKKSISYITVIGSMR
>NvitOR158
MSESKRLEIFESDYYRMYTNALRLIGLWPFECTYRQRIIRFFIIILLITFTILQGIRLYEEFGQN
LDIVLELIGSITFFIGCILKYIVTIQTQSMFQFLYEQIQSHWEIVTNRERRILEQSANDSQFFTK
LYMGAAYGALIVYVSTPIIVPNVLDVVIPLNESRAKTFPYFIEYFIDTEVYYYQLMAHGTLC
FTISALVYVSIDTMYATCSQHLCSLFDVVEYRLEKASKTDSKMNVNLDLNGNDKNIYKLL
NEAIVLHQDSLEFALFIENTYASCFLPVLGLYLTSIVIVAVDIVINLGDMNQIIRLSILYFAFSF
HVFFNCVPGQKIHDKSVSIMNSAYFSEWYNLPLEAKKLIQIIIHRSSIPCKFTAGGIVVLNVE
NFIVIMKSTLSYITLLSSIR
>NvitOR159
MSVRKDMHVFESQYYRIYKNSVKIIGLWPYENIQIKRVIRISIILLLISLVILQAIRLYEELGR
DLDIVLELIASLSYFAGCLSKYITTIRAQSAFRFLYDLIAGHWQIITDIKEREILEESTRQSQT
LCLSYMVAAYSALVVYSTMPAIAPAVLDIVIPLNESRKKTFPYYAEYFIDDEAYYYQLMGH
GTIVFTVSVMVYVSIDTMYACCAQHLCGLFSIVEYRLQEALRTDDKLHLEPPERDKLTHK
KLHEAIILHKDSIEFAFLIENTYALCFLLVMGLNLTVIVFTAVVIIINLGDMKQMIRLTLLFGA
FSFHLFFNCVPGQKVHDKSISIMNSAYFSEWYNVSLKSRKLIKFVMHRSLNPCQFTAGGLF
VMNMENFGSIMKSSMSYVTVIASIR
>NvitOR160
MSVYSSDYWKMPVLAQKFMGVWPFNNRQYDKCMRVFVYVALYSLIVPIGIRLVEELGVN
TAIAIENLVGQMYLNAAVIKFSMTILFKEKHKQIYELIARDWKMTSDKEELEIMEKHAAIG
RTISLAYGICCCSTAGAFLMIPTLLPLLDYVAPLDNSSRPVVLPYYAEYYIDQRKYYLPLML
KALVAGMISMTVFITYDMAFAMCVQHVCSLFDIINLRLQRASQLGSQLGSQLGRSARTSAS
YDSGVFRLIQKAIELHQIVIENVSSLENAYNLNWFFILLLNTTAVGGALLVVLLKLGHPEDL
VRYGMFFAAIFIHKYFIFLPGQKIINYSLEVFEYSYSCEWYNLSAECKVLIKIMMLRSIRPLN
LTGGKMFLLCMETYSAMLKAGMSYFTVFASTQSF
>NvitOR161
MELFESNYWKLTVFLQKLIGLYYFQSLWKNIVAWIYVYTFTLSFIIAIGVRLYQEIGIDINIVT
ENLVAEMYLIIVFAKLTTSVVYMKDLKRLYKSIANDWRVMSDEKELKVMHEYTDIGRKSV
QLYSGYMIIGIAIFLSLPISAPLWDYIVPLENATRPNALPYYAEYGVDQEKYYFPLMGQAVF
GGIGTGMLLVTFDLGFILTVQHVVALFALVCYRLDQAANLSLSVERGKIDFIKADRSAYEY
TVKAINLHQTVLGYVDLVENCYNAGWLVVLFMNMLLCGGGLAVLLMKTDRPEELLRYF
TVLFAGFIHFYYIFLPGQKIINSSLEVFDKCYASRWYNLSEKSKSLIKIMMIRSLRRCELTGG
KMFILCMDTYCNMMKTGLSVFTVLR
>NvitOR162
MFDSKINTQDDFNLDIFETTDYKLYKDGMKLIGLWPFESSTKKTLKRAFVLISMISVLISIQI
RFVEELNQNIDIVLQSAGSEILSIGCIAKFVTTLRAEDSFRVLFIQIAKQWASITDETECKILA
DNVKLCHPLCTFYRVMAVFALSSYACLPSFGPVIMNILLPLNETRQKRIPAPAEYFVDEEKY
FYILFSHGMILYMLVCVLYVTIDSMYSCIVHHTVGLVGIVTYRLQNIIDLDITSSPKNHTNN

LEIRRRLRRAITLHKESIEFAENIEATYSLCFIIVMFVNLFSMVFTAACGIRTLHYDKVESFRWLMLYGSIIFHLFFNSNPGQNLFDKTSEIINTLYFTAWYDSGISTSNKRIIQIMMIRCLRPCQLTAGGLLVLNMFNFGAIVKTSFSYITMLLSVG

>NvitOR163

MFDSKINTQDDFNLDIFETTDYKLYKDGMKLIGLWPFESSTKKNIKRAFVLISMISVLIFIQIRFVEELNQNIDIALQSAGTEILSIGCIAKFVTTLRAEDSFRVLFIQIAKQWASITDETECKILADNVKLCHPLCTFYRVMAAFVLSSYACLPSFGPVIMNILLPLNETRQKRIPAPAEYFVDEEKYFYILFSHGMILYMLVSLLYVTIDSMYSCIVHHTVGLVGIVTYRLQNIIDLNITSSPKNHTNNLEIRRRLRRAITLHKESIEFAENIEATYSLCFIIVIFVNLFSMVFTAACGIRTLHYNKVESFRWLMLYGSIIFHLFFNSNPGQNLFDKTSEIINTLYFTAWYDSGISTSNKRIIQITMIRCLRPCQLTAGGLLVLNMFNFGVIVKTSFSYITMLLSVG

>NvitOR166

MLSFKIQAKVNNGDVLGVGYWKLNKLMLKSVGLWPYQKSSTKMCIRTFIFIAIYSMMIPQIIRTFEEWGKNSEIVIENITGFLYFQVVITKYVTSCIAESNLQYLYVRITEDWNHFRDEGEQKVLSHFASHGRFLTIGYSVYLYTAGIAFTTLPCLIPAVLDLIIPLNDSRQKVLCFYGEYFIDQRVYYYELLLHTFVCVMCTIMLFTTIDAAYACCIEHVIGLFNIVDYRLNQAFNLVKDKYDTKSEVMRSEIHKCVLRSIEVHNHSIEIVELIQTTYTTCFFFTTGISLICLSLGTVDMMLSVNNYINFARVFFAWCGIVIYFFYISMPGQRIIDASSDIFNSVYFSGWYDFPLKTQRLLKFMMMRCSVPCQFTAGPLLVLNLENCGVILKTAMSYCTFVFAIS

>NvitOR167

MEEHEILNNEYFKVNRFLLKLTGLWPYQKRHVKLIIRILYICAIHSMMIPQVIRTVEEWGKDFEIVLENIVGFIYLQCVLAKYIITFTAEPQLVFLYKKMAFDWTRYIEAEEQLSLQRAASNGQLMTIIYSVYVNFAGVGFATLPGTLPTILNIIAPLNESRPTKVLCFYAEYFIDQEEYYYQLLFQTFIGVMSTVFINATVDTLYVICAHHSDGLFNIVSYRFQKAFNKSQERYQVKSRNLVAAKNLDEEIHEYVLTAINIHNESIEFINLIQSTYTLYFFIQMSLTIISLSLATVVAMMNLHDIINLIRIFFIWCGIILNLAYISIAGQQIIDTSLQIFDSAYFCGWYNHPLKTQRLLKFIMLRCSRQCQITAGPMLVINLESCSNILKSSLSYCTFMIAVS

>NvitOR168

MEHDVKKYFKYKRGIVFMLSASGVWPNYTSHPAAVRLFLNICSALASGCMFYCIVNFCLNYATNINAFTSCLGLMIGFFSTFIKVIILPMQKEDLQSLNEGVSASYERNLRIVKFRHHLLAHFPMFSRFFYLYSYSVGMSVLLLTIMPLLALRQGKYVRMYPQLVPFSYEPGGSLHWSIYAFEVFCGFYLWSVTSGVDSVFGLYALHMVGELRLLNVRFQMLKSSNNYAKDLKSCVDSHIMLMESRHKLQRIFGFLAIWLAITCAIALCALVFQALQAKHATIIRIIYLCGHCFLKLLQAYFYAWYGNIIAIESDACQSAIYESQWPGSGDKRFMNDVLVVLSQTPMIFKAKQWMPLRLDMFSKVVHTSVSYFFLLRTLDES

>NvitOR170

MELKVERKAKSGLREEDIFNNKYFILNKKLLALVGLWPYQDARLKRVVRILLVLCIYSMMIPQMMKGIEECREKNPNPEIILENISGFFYFQGVTAKFLTAILTEDKLKYVYEEVMKDWKRFTDKNEIAILCKFAHVGRVLTVVWSIYAAMSCLLFVTLPAVIPMILNIILTRNETFKKSLCIYCEYYIDQDKYFFYIFLHHIIAGIATIFLTIGIDTSYVNCVQHVLALFNVSSYRLKVAFDTIHHSKKNDYNLKTLENNVHSYVVSSIRLHQRSIKFVDTIQSAYNIVFFIVCALLLFGISIITVDLVWNVHNPINLIRIACLWMGTIMYMFYSNWPGQKLIDSSNELFDAIYTCGWFEFPMKTKILIRFMLLRSIDPCRLTAGPLLQMNFESCSLILRSAMSYFTVLVTTG

>NvitOR173

MDIFDGRYYKTSKWFLEFLGLWPFQSNRRRYVTCFIFVFMTATVVFPQVLLLIELKTSNFNI
LIENSLSIIFGFACLLKYGVTFASRSRLQTLLTQIASDWQRLTDKAEIDILSQYGEEGRYLVL
FYTVYVFLAWVTCNFVPFIPPLLDILLPLENGTHDLVYPFYADYVFFKQTDYHYESCLHVF
FVYFGTTSLFAGMDTIYVATVKHSCGLFAITWLETMARTGKSNRSNYSIKPNSVVHHDMV
EAIVMHNETIRFVELLEDSFSLCFLMVQCMIVAGLATLCFYMMRIYDKTFNMCQFSTFTV
GLVIHLLYLHWVGQKIIDSSDKVFYSTYYSDWYLISRNERQLTKIILARSLYPCQLTAGKISV
LSMETFGALMKTSMSYCTVLLSVS
>NvitOR175
MDIFDGAYYKSCKWFLSTLGLWPYQTDNRKRISAVIFFVVNISLAIPTLWLKSFTVTFENTV
SVMFAIGCCAKYVVTYTSSQRVRNKADELVRLFKQIASDWQRITDTTELSILTKYSEKGKF
LITFYQVYVWFGWTVYTLMPFIPYFLDKVSPLNESRPLLMPFYADYIVFDQADYHYTSCF
HIAFVYISSALLFCGVDATFVMSVQHTCGLFAIICHRLEGEKIKKESEYAQNVMKTLSEEEL
REVVIFHNNCITCSGLLEDSFNLSFLILNSMSVLGLALSGVYFIYIYEDYYKFVRIMAFFVG
LIIHLLYLNWVGQKIIDSSEDVFLAAYCSKWYVISTSARAFIKIIMVRALEPCRLTAGGLSTL
CMESFGILIKTSVSYFTVFLSVA
>NvitOR177
MDLFDGQYFKLNKIFLTICGLWPYQSKLRRRITFAMLASSTLLFIFTLVAGILSQSKFDFVNT
EETFIFIFYCSAGLLKCTILYNQQNKIKKLYERIATDWKQLTDTSERDILRSFLLEGRKLNFII
MISCSSAFIIYSCVDLLPRILKEKSEYHRPHSFPYYFRPMVINEKLYDLQVAVHITVIVFYAGF
AYMSAIATYISSVKHVCALYEIARYRLQNAVSYDKSNNSLLELVEDTSIVPKLVKVIDMHA
QALRGIQIIEKVFSADFFVLEASSLTALATDVYELKYCRANVRTFIRAKLLTPIVIIYLFFVNC
SGEQVIQACNDMRITAYYIDWYRTSSRARVFVLMIMRRTLNPKYLTAGTIVMIISIENFAAII
TTAWSIGTILLTT
>NvitOR179
MDSFDSQYFEINKRVLTICGLWPYQSKLGKRITFAMLASNTFLFNFTLIAGILTHSPGEFVNT
AETLVGLFFCSTGLLKCAILYNQQNKIKKLYERIATDLKKLTDNSERGILRSFLLEGRRQNF
LTMVYGVSAFIVCSCVEFLPRIFNEESEYHRPHSFPYYYRSMVIHEKFYDLQVAVHGTVIM
LYSGLTYMSAIATYISSVKHVCALYEIARYRLQNAIIYDKHNYPLQELTDDTSTIPKLIKVIN
MHKRALRVTKKIEKVFSADFFVMEASCLIALASGIFELNYFRGNVRAIIRPLLVMPIVTIYL
FFVNQSGQQVIQACNDIHTTAYNNDWYKASARVRIFVFMIMQRTLKPENLTAGSILILSIEN
FATILRAAWSFGTIMLTTLKHSPSRNEDA
>NvitOR180
MDLFYNQYFNINKHVSMICGLWPFQSQFGRRISYMIFAMSTFSMIFSLTAGIISQLNPDLLNI
LETCVALFFCVCGFLVCTILYNQKNQIKRLYERIAADWENLTDDLERDVLRTFLLEGRKLIF
ITLVYSFPAFSLFACITFLPRMFSEESTKLCLHSFPYYLESMVIDKNLCNLQVSLHYSVALGY
VGLSFLSVGATYICSVKHVCALYEIARLRLENATVRYGNYDPLGELTDETSIIHNLIEAIDM
HKNALRGIQIIEQVFSTGFFIIQIFGLSLLAILICELKYHEGEITEMIRFMLVLSVFVIYLFFMN
WSGEQVIQSCDDIQKTAYDIDWHRISSRTRIFVLMIMQRTLKPVHLTAGNMMILSIQNFGTI
LKSAWSFGTILLTTQKSV
>NvitOR181
MDLFDSQYFKINKLALTVYGLWPYQSEIGRIINHVIFVVTSFSMIFAMAAGIQSQVNAELK
NILETVVALVFCGAGLVKCTILYNQRNQIKKLYERIAADWEKLTDTSERDILRAFLLEGRHS
IVITIVYAVPAFCLFICVEFLPRIFSKESAKHRLHSFPYYYKSMVISENTYDLQVCVHLMVVI
IYVGFSYLCASATYISSVKHVCALYAIACQRLRNAIVYRKNSTPLKELIEDTSVIPNLIKVIE

MHKEALRGIQIIEQVFSAGFFVFEISALTTIAILIFDLNYHQGNPFQMMRVLLILSVFVLYLFF
MNWCGEQTIQSCNNVNAQAYNIEWYGISLKARVFVLMILRRTLKPIHLTAGTIMILSMENF
GTILKTAWSFGMILLTTQTSARNKDPNFFGY
>NvitOR182
MDLFDSQYFKINKRVLMICGLWPYQSILGRRIAFAMLANSIFLFVFTLIAGVISQSQLDIINT
EDTIIITFFCLLGLLKCTMFYNQQNKIKNLYECIATDWNKLTDSSEHNILRSFLLDGRKINFI
TMVFCSSAFMIYSCIDFLLRIFNKESEYQRQHSFPYYFKPMVIYEKLYDWQVALHVTVIVIY
SGLAYLSAIVTYISSVKHVCALYEIARHRLQNAIIACDKINHPLQKCIEDISLIPKLIKVIEMH
EQAVRGIRIIKKVFGADFFVLTVFCISALTIGTFELNFCRADIHSFIRVLLLMPIIMIYLFYVNY
SGEQVIQACDDMYTTAYNIDWYKTSSKTRIFVLMIMRRTLKSEYLTAGTMIMILSIKNFATII
KTAWSFGTLLLTTQKHRKNEDANFVAENTLI
>NvitOR183
MTLINSHYFKLNKLLLTFCGLWPYQTKLKRRINYTTFAIITLSMIFSLAGGIQSELNTGFMNI
SESIIALLFFSTGFLKCTIFYNQRNQLKILYEQTAHDLKKMTHHLERDILQAFLLEARNFNV
VSLVYSIPIYIVFAIATYLPQVFGFTNESAKYELHFFLYYKPMIIHESVQDLQVLIHATISTIYV
GSAYLCVSATYISSVKHVCALFEIARYRLKNVIVDHSNNNRRGLMKNASVISNLIKVIDIHE
KALRGVQRIDNVFNASLFILEVTALSAVTILIFHLNYHQGNFRQMTRYSTILSAFVSYLFFC
NWFGEQVIQSCNDIRETAYNVNWYNMSLRARMFVLMIMQRTLKPVHLTAGTVVILSMEN
FSAFLKTAWSFGTLLLTTQKPSPKENSIFFEY
>NvitOR187
MKLNGYDKFYYTLHKYVLTCIGLWPYQSRMSKRFFFITYGISSCSLIIALIAGLSEKWSTDP
VIILENMLGIIFLTSSTAESSILYMHESKIIEFYDKIKTDWKKLTNKKEIEILQMHTRKGQFVS
TAYILYGIPAFAIFGFVTFLPPILDPPSRVEYSHIFPYYFYCMIINEDFRYYQIVLHCMVSFSYA
SVSYLAVNCTFAKCVNHVCGIYAIICYRLQNAIEPTVVRGPFNKLKNSKLIRFNLIDVIAKH
REVIHGVDMIEQIFSTGFLVIEIAGFSGIALVIADILYNQKNAYQLFRIMVVTAIFLVYIFYIN
WMGEQIIQVSDDVRLTAYFIDWFTLSIEAQEIIHMIVWRSCKTNKLTAGSFVALSLENFLSM
LKTSWSVATVLLSAHRSQKNAHFTGYGITNSFTNSSST
>NvitOR188
MNIFDFPQRHLLTCLGLWPYQSKFTQRIFFTCAILSFFSLFVAMAAGLGEEWSTELVIIYETI
VALFVIFGGLAKCIVLFCRKHQMKSLYDQIRKDWQELTNEKEAAILQSFMLKGKAQIILYV
VCAIPGYFIFVALTYVPIISSEDASKDYSHTFPYYTDLLILSKRFRVYQVFIHAGLGIFCGGIT
YVAFMAMYITCVRHVCALYAIVRYRLENMVKSQDKLMDKLNDDEEVIPGLLEIICTHKRA
IKRVRLINRIFSRTFFMVEICLLICLALLIFDVKYNQHNVRLVIRMLMIALMFIVHVFCMNY
CGEQVIQFSTDVQYAAYFMEWYMISSRAQKILIMILCRSSNPDYLTAGNMALSLKNFASIV
RTSWSMATVLLTTQKVNRPSYSIS
>NvitOR189
MEEPDLDIPYMKLQKLLMNCCGLWPYNSRLINRLIYSFFLLILISTIVPLGLGLIEEANNDIV
TYFESLVSVVTMFGGVAQITMLRTIRNHLVKHLYKKITADWQTLKDAKEIKILSAFSFKGR
SLTFLYMLITMSSYVIYLMLIYIPLANDKATSWDYSKIFPYYSKHWIISERVRHLQVTLHGC
FGIFYGGVAYVVGMALYICCCKHVCGMYAIVGYRLKRLIISCEVTSSGKLRDDVLVYNLYA
IMDQHKEAIKGVHLLARLFSHSFFLIQLCLLVCLSFLIFGVQYNIYSHKGMVRMFPASIIFVA
HVFFMNYGGEQIIYYSSKIHTTTHFMQWYLLSVKSRRILLMLIRRSCKPEQLNAGTMTLSL
VNFTSIIKASWSMGTVLVSAHRKH
>NvitOR190

MPMEEPDSSIPYVKIQKFLMNCCGIWPYNSRLVNCLIYSFFVVFSFTTMTPLSLGLNEEAN
NDIVTYFETLVAVVAIFGGFAQITMLGIRNHLLKCLYRKISTDWRTLKDARETEILSAFSIEG
RSLTFLFMLITISSYVFYLLLTYIPLINDEVASSDYSEIFPYYSNDWIISDRMRHLQVILHGCF
GIFYGGAAYIIVMALYICSFKHICGMYAIVGYRLKRLVTSCKLTSSGELRDDDAVSKLYAIID
QHEEAIKGVRLLVRLFSRFFFIIELFLLICLAFLIFVLQYDIRSSKVIVRIFLASIILVTHVFFMN
YGGEQIIHYSSKIHTTTHFMQWYLLSVKCRRILLMVIQRSCQSEQLSAGIMTLSLENFTSIIR
ASWSMGTVLMSAHRKE
>NvitOR191
MDIFQSSYYIRCNRYLSFCGHWPYQSLRNRIRNFVLLMFLMSTILIPQIIKFWQLRHNIHVF
VAALPSMLYYCAFLFKNSFSMLQSKEIKKVLEKIKSDFQRYKDEDLKILHKYSGQANKINT
FYTVYMFMAVGGYSMLPLTLHVMDIALPKNESRLPTKPRLINYNIEAFDENIFFIIIHGVIV
DTAVIVFIIGFETLCFSFSYHVCALFVIVTNKIRDSIDERITSKHSEVDQDIFYRNFVKIVIMH
KDALDFVDTVETALSVLNLFAIGFAMMPLTITGFEFILSKGNVGEMARWSLFAFGEIVHLF
YYNWPGQKIRDHSLCVYQSCYAIEWYKEEIPDKCKKLLNLMMLRGQKPCSLTAGKVYIL
GLENFAAVMKVSMSYFTVLSSVM
>NvitOR192
MDILKSSYYIRCNKYLSFYGHWPYQNVIVKIRNQIVIMLLIMSIFLPQFMKMIEIRHYFHYF
ILSLPSLLYYTQFIAKNVFAFVGRKQIKNVLDKIQQDFQVYKGEDLAVLHEYSKKAQKFNK
FYTVYMFMVVGAYSMLPFTLYMLDTFVPLNYSRLPYKPRLVKYCITTFDDNILFIIIHGGIA
DMMAIVFVIGFDTLFLSFAYHICALFVIVTHKIRDAVNDEIDSQNSTINECSNLREDISYRNF
VKTITLHKYVLTFIDTIETAFSPLNVISIALAMVPLTITGFEVVMNKGNPGEMLRYAMYAIA
EMIHLFYYNWPGQKIRDHSMLIYEACYATNWYRKDFSVRSKKIMNLMMIRSQKPSYLTA
GKIYVLGLENFAAVMRVSMSYFTVLSSVT
>NvitOR193
MEDNVLDGPYYVYCKNYLSSFGTWPLQSYKKKVLLRTLMYLGCSSALIPHVTKAYELRN
HLEYFFLCIPSIIFYVQVLTKISCMILNEDKCKELIKQIKSDFQSYTGDNLRILNEYAEQARK
VNHVYIYYFMGTVVVYNTSAFVPLLLDLLVPLNETRPRPVLRLMKYNIQRIENNFFVTTL
HGFVLNILGMMLIMGFDTLLLNCSQHACALFQIVITELKDTIDKHKIEATSDTAKDTNSRD
VFYQEVVKVIIKHKHAIEFVDLVESIYAMANLLVIGITLGSITLAEFETVQHKDNHEIAFRYA
IFTSGELLHILFHNYPGQRIKDHSLMVYQSCYNCEWYREGITDECKKLLSFMMLRSQKPS
CLTGGGLYVLGLENYATILKASLSYFTFLSSV
>NvitOR194
MDIINGPDFVYSRICLRPFGLWPFQDPKSKLISRVITLMAVSTVLIPHIMKTYEFRNDFHILL
MCIPSLLYYAHYITKFLYIAFREEKVFRNVLERIKDDFVTFRGESLNHLTNYSEEARKFNTF
YMMYLCSTVVIYNVTAFIPHMLDFVFPLENATRPRHAARLVKYNIHQIDNNFYFVLIHGMI
FDVVAIAIIIGFDALFINCAQHACALFKIVVVELRKSTKLDEKMSNSASDLVTLQCRQDIFY
AKLVRTIIAHKHAIEFTDNLESTYALVNFLMIGIAVATITLTEFETIVHVNEVDIMCRFAFFSG
GELISMLYQNWPGQRIKDHSLRVHASCFECEWYREDVSYKSKRLLMFMMLKSEVPSALT
AGKLFILDLQNYVKIFKASLSYFAFLSSVAKVSSN
>NvitOR195
MKMKDNVLDGPYYVYSKNNLSPFGMWPLQSYKKKVLLRTLIYLGCNSVFIPHVCKAYE
VRNNFQYFFICIPSVIFYIHVMLKMACMILNEDKCKELFKQIKNDFEIYTGESLRILNKYAE
QARKVNRVYVYYFMGTIVAYNTLAFMPLFLDFLVPLNETRPRIITKHMKYNIKRIENNYFV
TTLHGYVFNILGMMVVVGFDSLLLSYTQHACALFQVVRNELKDTIDKHEIEVTSHAAKD

ANSRDVFYQEVVKVIIKHKHAIEFADLVESTYAVTNLLVIGITLGFITLVEFETVQHKDNRA
LGIRFAIITIVELLHILFHNYPGQRIKDHSLRVHQSCYDCEWYREGITDECKKLLSFMMLRS
QKPSCLTGGGLYVLGMENYATILKASLSYFTFLSSV
>NvitOR196
MAYAEEPRENILESSTFLYSKSSLRVFGLWPYQEPKQRLICRTSTAVLIGSLLIPTICIVLEQW
RNFDYVVLGLPSFLYYVEFVTKYMYLAANQKKLEQIFGHIKNDFDTRKDRKLEILKDYAT
ETRLFNHIYAAYLIIVVVMFNLSFYQPHFLDLIMPLNESRPRPIPRLARYYVTSLDESFNFVV
LHGLVIDWYSMIFFLGHDTLLVNCAQHACALFKIVINDIQDCLVVQKNDKADDEDQFYRR
ISNTIDLHKWALEYTAMVDKMYMYVNLCIIGVSLLAITLSQYQTAIHLDNTDLVIRYSFFSI
GELVHILYFNWPGQRIRDHSLSIYQACYNCEWYRDDISYRCKKLLKLMMARSQLPSNLSA
GKLYVLGYENFAQVLKASLSFFTVLLSVN
>NvitOR198
MDKERNMDRMNFYYIYSRISMLSLGLWPYQSWSSMMTLRSLWIIQHISIMLPELIKIYENR
GHFNLLIESLPPFTYNIVMAIKFTNGVLNQRKLKSILEKIKYDWNKFTDKKEIEMLCYYSH
RGKSLNTVYIGLVAVVLLSYMLLPMLPAVLDLINPLNESRPKSPLYMVEFYIDQDKYFYSV
LTHAYITSLAGVLPLFATDLLFSNCAHHACGIIKILGRRIENILSEEPALKRSYKVDDEKKAI
ACVIEHQNIIKYCESINSLYTTSFFLILSISIGLMSVTGFVTLIKMNEEFKDCIRFAMFTFAQIF
HQFCYYFLGQSVLNHEEKLKDYVSNFNWYKASPKTKFIIKFMIMRTLKPTKIRAMIFPLTL
ENFTSLMKTTMSYFTVIKSTR
>NvitOR201
MDKERNMKFYFVYTRLSMLCLGIWPYQSWSSMLTLRSLWVIQHISILLPEGIKIYKNRKNL
NSIIDGLPPFIYNVVIAIKFINGIINQHKIKSILEKIKNDWNQLSEKKEIEMLRDYSDTGKAFN
TVYLSLVTVILLSYMLIPMLPAALDLVNPLNESRPTSPLYLVELYIDQDKYFYSVLTHAYITS
LAGILPLFAIDSLFSSCAHHACGMIEILGGRLENIINEEASIKEIDNNEEEKNAIACVIEHRGV
IKYCESINSLYSTSFFFVLSFAIVMMSVTGFVAVIKMGEEFKDSIRFAMFTFAQIFHMFCYYF
LGEIVLHHEEKLKDYASNLNWYKASPKTKYIIKFMIMRALKPTTMRAIIFPLTLENFTTLM
KTTMSYFTVIKSTR
>NvitOR202
MEQHYSIRTYFKLNRVFMVSSGVWPYQPLHVARIIRLLWITQHISIMTPEIIKLIEVRGMAD
LLLECIPSVFYNIVIAVLYGTTIHHQRKIKELIEKIQKNWITISKKSEVEILTRYSNMGIRIGWL
YIGALYFTLFIFCLFPLSPIVMDYVNPLNVSRQRLPLYRVQFFVDDKKYYWTILMHAYTTT
MIGIIPLLTVDLFLANCTQHICGMMLILGKRLEKTMETTKLVVNKLDDNIYKDIRKCTILHT
EILDFIEDINYIFSTAFGILLAVLTFLTSFTGIVVLIKWGDWNEVIRFGMFTMAELFHAFCYS
YHSQDVIDHNNQIHKSIMNSGWYKSSMRTRVLVQMMFLRSNKPCLINCIIFPLSMENFTTI
LKTMFSYFTVVKSCRF
>NvitOR203
MDGKQCLQTYFIVNKVFLFSCGTWPYQHTIFAKTFRYFWITQQIVIMAAKSIKLYEIKNDT
DLVIEAVASFFYNISITIKFVNQVINEHKVKIILEKIQDDWKSLEDDSEIKILSYYARLGKLFN
FMYIGAVYSALISYMVLPLTPIILDFIVPLNESRPKQPLIMAEFFIDQDKYFYPLMIHAYLSVL
YGIIPLLGTDTLYMNCVHHSCGMLKILGNRIRNILNSSSRELSNKIKYEKMVKCIIQHQNIIE
FCNNINETYSTSFLIVLCFSITLMSFSGVATVIKLGDNFNDVIRFGFFSVAQIFHLLCYNYMG
QNVLNYGEELRAQIYNTNWYEASLKTQRLVKFMMAKNMHPIILRANIVPLCLPNFTRVIK
TSMSYFTVLQSTR
>NvitOR204

MEKENDCIRAYYKLNKFSMTLLGHWPYQSENSVKIVTFLWIFQHLSILLPELIKFVEIRNN
VDYVILAFSPIIYNIVVGIKFVNGSLNRHKIKITLDTIQSDWKSLRTEEEARILANYSSFGKL
CTVGWAWICTTTIICYLLFPFTPFVLDLIRPLNETRPRQLIYMVEFFIDEDKYFYEIQIHSYAT
TLIGFIPLISIDTFYAASVQHACGMFAILGHRLRRINGAMSKSKKRSDEDAYREIVSCAIQHD
KILQYCDNLNDTYTDSFFYIMGCNMISLSFCGVLLILMWGRIYDMLRNGIFTFAQIFHLFY
YSFQGQVLSDRSLMISDCVYDSEWYTASLRTRKIMTMVSMRSLKPFLLTAKVYVMSLPNF
TLVIKTSMSYFTVLKSSR
>NvitOR207
MIFSFKDKFFPYYAWQTVTVLEKAAGWWPFQNRTKNMTLRLMHFGNLLLIIIMCSVRLFQ
EYKQKKLYIVVENTVILVMIICTKMKVIMFFINEKQRKIFYENVLIHWKDTNDEEEMMILK
QYAKLGLKTIINYAISVNILFQGVPVLSDVINYLNDPNITYLKKELPIYIEVYIDQEKYFYQL
YVVLFFMTCAALLLAFSHELTFFQSVQHINAMFKIIEIRIIRLSKIVKRTECGLDTFKEADRK
IFVCISRAVDLHNAVLNNIKFINSSFGATYLVVLLFNCLIFGASLFLIFNNSDQKIHLIRYGLV
FLGLSVHFFIIFWPGQKIMDGSESLFNVCCSCDWYKLSKRSKNLLRIMMLKSVMQCQITAS
GMFVLSFETYVKLFKTGLSFVAVFS
>NvitOR216
MTISETDLAVFDGPHYSLNKKLLIMFGLWPTLSRTRKVICLIFFTMIDLSLYASFADGINYYR
KQKKWMYVIEDTISIIYLSVTWIKYVTSYIFESRIKLIYEQIAADWKSLIDEEEIQILNNYSAF
ARLLTVLYVFYAIIATTLFYVLPFLTIVIDRIKPLENGTRFRAQPYHQHYFDLIDNEKYYYH
MYIGHGYVVSIIVTVAVMAIDTMYAANIQHACGLFAIVRHRLSKIGILNGEREYEFRVVDD
KKVYESIRAVCEMHKNSIKIVELIWDSFSISFLIFMGCSLVGMGMLMFNYIFNMIHPIEKVV
GTGLLLGIAILLFYMNWIAQQLTNSSDEIFIAVYSNRWYNLSIKGQKLIYSLLQSNANSVTL
RAGGIAEMNLQQFAAILKTAMSYATVMMSMNG
>NvitOR217
MGIPDPENPLAIFYTDYYKYNRKLLEICGLWPELSRPRKIIMMILFALLMTSLIIPMGAGAIH
YFHKGRIMYVVEDLIGLLYLTVASSKYFTYSVFEGRILRLYHQVGEDWRTTTDEEERKILQ
EYSEFARLLSIIYFVYAAIGAFYFNMSPYLPLGLDRWMPLESNESRVRIRTYHPYYFDLIDA
EKYYYECYFFHGNSVVYYVSTMVGLSVDSMTAFNVQHICALFHIVGHRLRKIGSTLEINAK
GEKIARVDDITVVRQIKHVCGMHRTSIDSVELLQSSFGMNWLVFLIGTVTGIALLMFDLIFS
MKHPLEKMTGLVIFIGIQILIFYINWIAQKLTDSTEQIFLAACETCWYNLSVKGQKLVYFM
MQKNIIPLTLTAGGIAELNFQQFASVSKTSMSYAMVILQMNDE
>NvitOR218
MPVETKDQRKSYDFLSISSSHLTFMRLSSFLPLKGKSFFHPLSLLLQLWDHFIVIGYNVMW
QGYGIRMIQRGDVEVDFICEDIITVGFTIRYLLLCAKREKLCCLVESCEKLWDLLKDGEVIF
VRQFARKGYYFRNFILINAMLMAALYSVTAPFVRLPPIEANGTERKILPFRFFMDIQKEPAY
SIVFAFQSILLQFIDLMIVSTETVSLYLIMMACGYLRSVRNRLLSFKGNDDNTSEKGEAALK
FVIDCAHFHQQIMIFCEDIERMTRTLFFFACFCPIYNVSITGIVLFNNDEDKYKFLPLLCYNF
FQFFLCQWAPEHLAVESEDIALAAYSASLRPQAPSHREKINRILYFMMMRAQKPVQLTAGG
FVDLSIETFGAMTKSAFSFFMVLRKFRS
>NvitOR219
MSTKAEDSHTFQSISASHLNYLRLCSLLPWQGKGFSHPFSLLFQLWNHLAIFGFNAMWHG
YGIRMLQRGDVEIDLICEEITVLDITARYFLLLIKREKLGRHIETCRKLWSYLKAGEDMFVS
QFERKGYYLRNFVMINSVMVTTAFIITATFVRLPPLEANGTERRMLPARFFMDVQEDPAYS
IVFASQSILLLSVDVMIGSTQTVSLYPIMMACGYLRSVRNRLLSLEGSDNGTDAKGEATFK

FVVDCAHFHQQIIIFCEDIERMTRMLFFFACFCPIYNVSIAGIVILNSNEDKIKFVLLLVYNFF
LFFLCQWAPEHLTVESRAIAEAAYFASLQPLASSYREKINWILYFMVVRAQKPIQLTAGGFA
PLSIQTFGAMTKSAFSFFMVLRNFRT
>NvitOR221
MRGAMSAEPKDLRESFTFLSISSSHLIFMRMFSYLPLKGKSFSHPLSRLLQLWNHFAVFGF
NAMWQGYGIRMIQRGDVEVDFLCEDIITIGFTIRYIVMRIKREQLCRLVESCEKLWDLLED
GEAVFVRKFERKGYYFRTFILCNALLMAGSYSIAAPFVRLPPLEANGTERKILPFRFFMDV
QEEPAYSIVFVLQSIALQFLDFMMVMTETISLYLIMMACGYLRSVRNRLLNLKGSDSDPSE
KGEAALKAVVGCAHFHQQIMIYCEDLSKMTETLFLISCFCPIYNVSVTCLVILNTEEDNLK
FVPLMLYNFFQFFLCQWAPEHLTVESDNIAEAAYFASLQPQAPSHREKINRILYYMMMRA
QKPVQLTAGGFAPLSIKTFGAMTKNAFSFFMVLRNFKN
>NvitOR222
MTTKTKDGYTFLSISSSHLIFLRLSSFLPLKDKSFSHPLSILLQLWDHFVVMVYNSMWTGY
GIRMILRGEMEIDFICENVVVMGFTVWYIVIQMKRQQFCSLVKFCEKLWSYLEVGEEVVV
RQFERKGHYFRNFMLFNLLLMCTLFITTAHFIKLPPLEANGTERKILPFRFFMDVQEEPAYS
AMYTLQFFVCYFVVFMIASAETVSLYLIMMACGYLRSVRNRLLSLEGNDDDTGEKGEAA
FKLVVGCAYFHQQILIFCKDIERMTRTLFLFACFCPIYNASITGIVLLNNDEDKFKFILNLFY
NFFQFFLCQWAPEYLSEESEVIAEAAYFASLQPLASSHRQKINRILFFMMMRAQKPVQLTA
GGFVKLSIETFGAMSKNAFSFFMVLQNFRS
>NvitOR224
MPGAMSAETKNTFLSISSSHLIFLRLASFLPLRSKSFSHPLSLLLQLWDHWSVLAGNMMW
SGYGIRMTLRGEMEIDFICEDIIMVGFTMRYILLATKRKKLCHLVESCEKLWDYLEIGEDAL
VRQFERRGYYYRNFMMLNLLLMCTLYIVTAHFATLPPLEANGTERRMLPFKFFMDVQEEP
AYSIAFVSQSVVTYFICFMFVSTETVPLYLILMACGYLRSVRDRLLSIEGSDDDTSERGEVA
FKFVAGCAHFHQQIMIFCEDIKHTMRTIFLFACFCPIYNLSITGIKLLENDEDKFKFIVILVYN
FFQFFLCQWAPEYLIEESEDIAAAAYSASLQPQALSHREKINGILYFMMMRAQKPMQLTAG
GFVRLSVETFGAMTKNAFSFFMVLRNFSS
>NvitOR225
MSTEMEDPHESYTFLSISSSHLIFMQLSSFLPLKNKRFTHPLSLLLQLWGHFVVFASNVFWT
GYGIHMVMHGEVEVDFICEEIVVLDFTARYILLIVNREQLCCLVKSCGRLCSYLEAGEDIF
VRQFERKVYYFRNFVIINSLLVSTVFDVTAYFTRLPALEANGTERRMLPARFFMDVQEQPA
YVVTFVMQVILDYYLDFLIASTGAAPFYLIMMACGYLRSLRNRLLNFKGGDYDTSEQGE
AALDTVIGCAHFHQQMMIFCKNIERMTQTLFLFACFCPIYNVSITGIAILNSDEDILKFTPLL
VYNFIQFFICQWASEYLAEESEAIAEAAYFASLQPQVPSHRERINRILYFMMMRAQKPVQLT
AGGLVNLSIQTFGAMTKSAFSFFMVLRD
>NvitOR226
MSAKKKVQKEGDTFLSLSWSHILFLRVASFLPLKGKSFSHPLSLLLQLWDHINVIAFTSLW
QGYGYRMIKRGEMEIDFICENIITIGFTLRYIILCLNRELLCHLVESCEKLWDLLEDGETVFV
RQFERKGYNFRNFFFGNLMFMATLYTITAAFVKLPPMEPNGTETRMLPFRFFMDVQENPG
YAAAFVFQDVVVFYTDVIFASAETVPLYLVLMACGYLRAVRNRLLKIEGNDNDSSEKGEA
ALKVVVGCAHFHQQIMIYCEEIGQMTKTLFLVSCFAPIYNVSIAGIKLLENDEDKFKFIVIL
VYNYFQFFICQWAPEYLTEESESIAVAAYSASLRPQAPSHRQKINGILYFMIMRAQKPVQLT
AGGFVNLSIQTFGAMTKSAFSFFTVLRNFSG
>NvitOR227

MPTGMDSLTTTFQSISSSHLMFLQLSLFLPLESKSFSQRLISNLLQLWNHLMVIVYNVSYAG
YGIGMALRRDIEIDYICEQIVVETFSARYILLCFKRAQLRRLIESCKRLWGYLEVGEDIVVR
QFERKGHFFRHFLILSSLMAVTSYVVTAHFLRLPPLEANGTERKMLPFRFFMDVQEGPAFN
AMYALQIINSYYLVFMFASVETVSLYLIMMACGYLRSLQDRLLSLITEMNEDDLSKNGEAT
FNVVMGCAHFHQKIMIFCKDVDQMTRTLFLFACFCPIYNMSITGIKLLESDEDKFKYASLL
FVNLFQFFSCQWAPEFLIIESEAIATAAYFASLQPFAPSHREKINRILYFMMMRAQKPIQLTA
GGFIKLSIETFGAMVKSAFSFFAVLRSFRT
>NvitOR229
MLIEKKIESFTFLSISSSHLTFLRMAAFLPLDNRSFHHPYSRLLQLYGHICIFIYNTMWTGYG
YRMISRREFEIDYICEQMVVEGVCLRYIVLCAKREQLCALVESCKRLWSYLRSGEDVIVRQ
FERKAYFFRNFMLINSILVVMLFIGTACFVRLPPLEVIGTERKVLPFRFYVDVQEDPMFSAV
YALQAVVCTTISFVIASIETVSLYLIMMACGYLRSLRNRLLSLAENEDDAILAGETSFRLVV
GCAHFHQQIMIFCEEVDRMTRTLFLFACFCTIYNMSITGIKLLENDENKFKFGAILSLNLFQ
FFTCQWAPEFLIIESEAIGKAAYFASLQPMASSHRERINRILYFIMMRAQKPIQLTAGGFIKLS
IQTFGAMVKSAFSFFAVLRSFSST
>NvitOR230
MEREPIKYEDISRLYYRLFRTMGILPSSSSRRTTLLRVYFHVTIVLYYSMSMFDGLRMLGHN
DIEIEYVFEEVVIHGICARFLILSCRREELAELLLSCEKLWRMLKPGEDRVVKSYEKIARYL
AHYITWTTLVAIFFYIVAARIVKLPPAEVNGTERRMLPFRFYVDVQRQPWYDIVTVLEIVV
VLNIAMIVSTIETTGPFLITMACGYLRSIRNRLLAIADEAEGRGEISRLSTIRVVSCVKFHQKI
MRFCQDIEKLTSSVLLVQVVCTAYNISLVGFRILKNDPNAVKFVPLLLLNLLQLFTAQWIPE
HLLSESKAIANAAYSASLLHPEYEPRANRALLFVMLRANRPVQITAGGYMKLSLETFKRM
LTSALSFFTVLRSINDGAGDEGE
>NvitOR232
MRLHEINSFERVPASGTIRKFVEFRETDGSLRIFSPPHRGFTFGEPRLQLSLLQDASHLLRVY
WPTQQCSGVVEVDSIFVMVMAVSTIMRYYILVYHRFDFRDTMDACRVIWNDCTPNEHQI
VRWFERKTWMLFKLLAGSGMFINVFCSIGSIVVRLPPDEPNGTERRLLPYRWFIEDREYH
WLGYELIFGLQVLITHHLTVIAATVDTAGPLLMMISCGFFKALQERFFAAAARNEMILCKD
KLEFKQTIVSCSKFHQSVLVLCKKIEVMTRMIFMVQLICLGYNISLIGLKLTGTDPERFQYIP
NLVLCLCQLFITQWASDYLLEQSEEVATAAYFATLMSLDARIGGLLLTMVIRAQKPVQMTA
GGVIKLSIERFGSLITNAISFFMVLRNFTTQV
>NvitOR233
MRVHEINSFERALASDEIRKVAEFQESDGGLRIFPSTHRGVTFGKPRLQLSQLPDASHLLHV
HRTPHQRHGAVELDSVFVTVMATSTIVRYYILVYHRFEFRDAIDACRDIWEDCTPSEHQIV
RWFERKSWILFKLLAGSGLLINIFCSIGSIVVRLPPDEPNGTERRMLPYKWFIEDREYYWM
GYELIFGLQVLILHHLTVLTATVDTAGPLLMLLSCGFLKALQERFFAAAVSNEKFFIEDKLS
YQPLLTSCSKFHQNVLNLCRKIEVIMRMIFMVQLMCLGYNISLIGLKLAGNDPERFQYIPN
LVLCLCQLFITQWAADYLLEQSEGVATAAYFTTLMSLDPRIGGLLLTVITRAQKPVQITAGG
VINLSVERFGNLITNAISFFMVLRSFTA
>NvitOR236
MEETSAFYRRIRRIQTRVLRLAGLVPFENRTLIFAGTILMSIYVNFAFTAVSSVYIWAFFEDC
LNKRFNPDITSELFSFVGFHFRFMYIFSRRRKLGEMLGYAESLWERVRSEEKVHVRLFVRK
VSKLSVCYSGIILTTITLYVLSSQLPQLTAAATNETVHRVLPYPFYVDVQSSPRYEILLGAQI
VCLLTVTQTSVCVDTAIAFLIMIACGHFRLIQVRLGVIARHIEENEDKRKSQRSVGKNGEVI

EAEAEMDEEDFERTDDRVRERVKELVMHHQEILSFCDDIKNLSSEIFMIELISTTYNLSLIGI
LLAGNMPLAEKFKFAPVLFILTTQLFVCQYPPDLLIQESEAVANAAYFVPPFRRDRRRIDRIL
LSLLTRSQTPYQLRAGGQIPLSIESFGNMIRGAVSFFTVLRSFN
>NvitOR241
MELELLRYEAYTHNVIWFLKSAGLWPEAHPVPRKILSMVTLCSTFVVMVTVSNFSFQNVG
NVMVLTRGMSLAVSFSSAFSKVALFLLHHDDLVYLNKHLTGGFMRDMKEPENRPDLLNN
VKTFNRFMFTHAISVAIAMSMYSIGPLLALRKHGKYIRAFPAIYPFAYESGGLVHWILYALE
VSGAASLWTVTVGVDCVFGLYALQVCGELRILAKKFRELRATENYREKLHDCIQRHHVLI
NAKNKLDNIFGLISIWLAISGALVLCSLIFQVTELLKTNNSYLRAAHLCAYLLPKFLQIFTYA
WYGNLIAEESGACLDAMYGSHWTDSCDKNFKSDILIVLAQEPLALVAMGCMVIQLDMFT
KIVKTSVSYFFLLRTLNEENE
>NvitOR242
MIMEKEVEKYKKYKSNLKFMIVSNGVWPDYEKHPYCVRKFLNFCSISSISMTNYCMMLF
VIATTTDVRSFTSFFGLLLGGFGNLFKVCALTMNQKELHALNEGISASFERNLRVPENRPH
LLANFPMFSKFFNFLSYSTLGTIGFLTVIPLLHLRHGTYSRMWPILLPFSYEPGGTIHWIIFVF
ELVVSFFAWITTCGVDCLFGLYSLHIVGEMRLLSSRFQKLEWSENYRKDIRSCVKSHLLLL
KTLSQMQEAFGDLAVWFAFNSAASLCTLVFQFSQLTVMNPARVLYLLCHTCIKLVQAYSY
SWYGNIITVESEVCLNAAYNSHWPNHGDKHFMRDVLIILLQRPMVFKAKSFIALRLDLFA
RIANTTLSYFFLLQTLDEKV
>NvitOR243
MELELLRYRAYTHNVIWFLKSAGLWPEGHPVSRKIRSMVTLFSTFVVMVTVSNFSFQNVS
NVMVLTRGMSLAVSFSSAFSKVALFLLNYEDLVYLNEHLTGIFERDMKKPEYRPDLLKNV
KTFHRFMYTHVASLTFTLIMYVIGPLLALRKHGKYVRVFPAIYPFAYEPGGLVHWILYILEV
LGATCLWSVTSGVDCVFGVYALQVCGELRILAKKFKELGAIENYREKLNDCIRRHHVLIK
AKNKLDNIFGLISIWLAISGALVLCSLIFQITELIKAKSSYLRVVHLSVYLLPKFLQIFSYAWY
GNLIAEESTGCLEAMYDSHWTDSLDKNFKSDILIVLVQEPLTLIAMGCMVIQLDMFTKIVK
TSVSYFFLLRTLNEK
>NvitOR245
MDQKRFKYKAYERNVIWLLKSAGLWPEAHPVPRKILSLVTLFTSFVVMVTATNYSFQNV
GNVRMLTKGMSLAVSFSSVFSKIAFFILHQEDLLYLNKHLTGGFMRDMKRPENGPALLSN
VKTFNRFLYMHAVSVAIAMIMYSITPLLVLRKHGKYIRTFPSIYPFAYELGGLVHWIIYAVEV
SAAATLVTVSAGVDNLFGFYALQMCGELRMLAHRFRDLRAGNNYKDNLKDCIERHQVLI
NAKNKLEDIFGLITIWLAISGSLVLCSLIFQVSELIKNHVSYLRIAHVCAYLLPKFLQIFLYA
WCGNLIAEESKICLYAMYDSHWPDSHNTNSKRDILIVMSQEPLSVVAMGCMVIQLDMFAK
IVKTSVSYFFLLRTLSAENE
>NvitOR246
MEHDVKKYFKYKRGIVFMLSASGVWPNYTSHPAAVRLFLNICSALASGCMFYCIVNFCL
NYATNINAFTSCLGLMIGFFSTFIKVIILPMQKEDLQSLNEGVSASYERNLRIVKFRHHLLA
HFPMFSRFFYLYSYSVGMSVLLLTIMPLLALRQGKYVRMYPQLVPFSYEPGGSLHWSIYAF
EVFCGFYLWSVTSGVDSVFGLYALHMVGELRLLNVRFQMLKSSNNYAKDLKSCVDSHIM
LMESRHKLQRIFGFLAIWLAITCAIALCALVFQALQAKHATIIRIIYLCGHCFLKLLQAYFYA
WYGNIIAIESDACQSAIYESQWPGSGDKRFMNDVLVVLSQTPMIFKAKQWMPLRLDMFS
KVVHTSVSYFFLLRTLDES
>NvitOR247

MELELAKYKSYARHVITRLIFAGIWPESNKTIKTILYFISFTSTLTVSVTSINFGIQNANNVILL
TKGIGLASAFSSVFSKALLLPLHQEDIIFLKNRLTTKFMSDMETIEYRADLLSSVHVFSAFF
NMHEAMVAFAMFMYCFVPLYVLFKHGTYLRTYPCLYPFSYTPGGLVHWLIYALEVAGAIS
VWTITVGADCGFLMYALELCGEFKILARKFTELKAGDGYKRNLKECIERHHLIIEAKNRLE
DSYGLIVIWLALSGAFLLCSLIFQITELYDNHGSYVRIAHLCSHLVAKNLQIFMYAWYGNLI
ADESKAFLNAMYDSHWPEACDKNFKNDILIVLTQEPLVVVAKGCMYVQLDMFTKIVKTS
MSYFFLIQTLAN
>NvitOR248
MMDNEVASYVKYSSYLKRLTAFIGLWPDYQKQMPAISLLLSIQAAFSSFTTFCFIAYSCYLD
SADIGAFTSYIGGLVGYLTTVMKIFVLGIQQKNLKKLNNGISASFEANLKVPENRQYLLAH
LPMSLRFFYTYAITTGSSLALLVLIPLLLLRHGVYVRMLPLTLPFSYKPGGMVHWMFYLYE
ILCGWNLWTVAVGTDNLFSLYCLHIVGELKLLSSRFRNLKSSKNYRKDMKDCIQSHMLLM
KTFLKLQKVFGFVVMWFAITCALCLCSLVFQAVEMDKVSVMRVFYLFNHSFVKLLQAYL
YTWCGNIITVESEICLNAAYEAHWSDSGDKRFMKDILTVVLQRPMVFKANKFMELRMEL
FLKIVNTSVSYFFLLRTLDDDS
>NvitOR249
MESKVARYAKYKRDIKCLIVASGIWPHYEKHPHVLRKLLSFCSAFCSGSTFYCIVAFCFKYA
TNINIFTSCLGLMIGFFTTFIKIVILSMRQEDLQSLNEGVSKSFENNLKLPENQPHLLYHFPSF
SRFFYLYAYVVGISFVFLASTPLSIMLRYGKYVRMYPQLMPFAYEPGGSVHWAVFGFEMFT
GFYLWSVTIGVDSIFGLYALHMVGQLRLLGSRFQNLKSSSNYDKELGECVRSHIQLMKSR
HKLQRVFGFLAIWLAVTCAIALCSQVFQALHMRNTTPVRALYLFGHWFIKIVQAYSYSWY
GNIIAVESDLCLNSMYYSHWPGSGDKRFMADVLIILSQKPLVFKAKQLMELRLDMFLKIV
HTSLSYFFLLRTLDENPKAGT
>NvitOR250
MELELAKYKSYARHVIFRLIFAGLWPESNPKIKRMLSFVTFTSTLTVMVTAINFGIHNASNV
ILLTKGIGLASGFSSVFSKALMLPLHQEDIVFLKKRLTSKFMSDMETIEYRADLLSSVHVFS
AFVNMHEAMMAFAMSMYCFVPLYVLFKHGTYMRTYPCLYPFSYTPGGLAHWLIYALEV
AGAISVWTITIGADCGFVMYALELCGEFKILARKFTELKAGDDYKKKLKECIERHHLIIEA
KNRLEDAYGIIAIWLALSGAFLLCSLIFQITELYDNNGSYVRIAHLCSHLLAKYLQIFMYAW
YGNLIADESKAFLDAMYGSHWTEACDKRFKNDILIVLTQEPLIVVAKGCMYVQLDMFTKI
VKTAMSYFFLIQTLAS
>NvitOR251
MERDIQTYKVCSENVTLCLIFSGVWSATHPVLKKIAFFVTFFSTFSIMAHTLNFSLHNAQN
VRILVRGLAAASSFLSISSKAFLFLQHQNDLNYLKDYLTEKFMSDMKNPENLPDLLSNMR
MFAVFVTMYKTTIAFIMSMYCIVPLFSFLKYGKYLRVYPCLYPFSFVPGGVVHWLLYGWE
STGALSAWAISVGTDCAFGMYAIQICGEQRVLARKLKDLRVGSNYTRELRDCMERHHLIIT
AKNTFESLYGLISIWLAISGAIVLCSLIFQVTEYLENRGGYVRAIIFFAHFSGKMMQVFMYA
WYGNLINEESLAFPRAIYSSHWTDCCDTRFKNDILIVLAQRPLIVTALGCMNVQLDMFAKI
VKTSISYFFLLQTLKAKTEEK
>NvitOR252
MEEDIQTYKVCLQNVVICLIFSGVWPATRPLLKRIAFFVTFFSTFSIMAHTLNFSLHNAQNV
RILVRGLAAASSFLSISSKVFLFFQHQDDLVYLNDYLSKKFMSDMQNPENLPDLLSNVRTF
AVFVRMYKTTAAFIASMYSVVPIIAFLKYGKYLRVYPCLYPFSYAPGGVVHWLLYGWESA
GALSAWAITVGTDCIFGMYAIQICGEQRILARKLKDLRVGSNYKKQLRDCMERHHFIITVK

NKFEDLYGLISIWLAISGAIVLCSLIFQVTEYLENDGGHVRAIIFFAHFSSKMMQVFMYAW
YGNLINEESLAFPRAIYSSHWTDCCDTRFKNDILIVLAQRPLIVTALGCMNVQLDMFAKIV
QSSISYFFLLQTLKAKGEEK
>NvitOR253
MELEMAKYNSYSNTIIWSLICSGLWPKGHYVLKKILSCISFLSITTIMTTAINFSFQNARNV
QLMTKGMGTAVSFSSVFSKIVMVLYHQNDFIYLKKHLTTRFKRDLEQTENRQDLLFNVHI
FTKFVNTHEASMAFAMFMYCIGPILALYRHGKYVRTFPCLYPFHYEPGDVVHWVIYGLEV
TGATVIWFITIGVDCGFCMYALELCGEFKVLGRKFRELRVADDYKEKLRDCIERHHLIINA
KNRLEDAFGIMAIWLALSGAFLLCSLIFQITEILENHGSYLKIAHLCSHLLAKYLQIFMYAW
YGNLIADESQSFLYSMYSSHWIDACDKRFKSDILIVLVQEPLMLVAKGCMNIQLDMFLKIV
KTAMSYFFLLQTISSEE
>NvitOR255
MEVELKKYKRYYRDIKLLLVVSGIWPNFYPILDRVVSIVAAISTLLLTMALLNFCAHHVANI
MILTKSMGIAISFFSSFLKICIFLSHHDDLVYLNDYLTSSHTSDLSNPDDRSHLLEKFSSFSKF
FYTLTIAVALTFVLNTIAPFFALKRGKYLHIYPVIFPFDYEPGGSVYWSLISLELTAGFFVWS
VTSGVDSVFGLYALQMCGELRVLAKRFEELRATGDYRMRMRECMDRHHLLMRSRDILEK
VFGFLAIWLAVTSALVQCSLVFQAKVEFKTLSPFKIGFFFFYILMKLVQAFTYAWYGNLIAE
ESALCLNAMYNAHWPGSGDVRFMNDVLIVLSQKPLIFKAKSCMSLHMDVFTKIMNTAVS
YFFLLQTLDEGSVRHL
>NvitOR256
METELRKYERYSRDLKCLLVLSGIWPDFHPIIQPLLGCFAAFVCFVTVIAFLNFSIHHITNVV
VLTKSFGLVISFFSSFLKICVFLWHHDDLVYLKAALTDRFNTDNLNKSFRRFTLAKVNVFA
NLFYILTIAVGLTTGMAVVLLIISLRHGKYVMLYPSIFPFSYEPGSRVYWILLMVELFANLFV
WAVTSGVDSVFGWYTLQICGEFRVLAHKFQNLKSSENYRDDLKECVERHYVLMKTRDV
LQDVFGFLTILLALTSAIVQCMLVFQAIQVFKNLSLGMMVFLIAYITLKVVQAFIYAWYGQ
LIAEESEVCLGAIYNARWAGSGDTRFMSDVVIVLSQKPLIFRANGCMSLKMDIFIKILNTSV
SYFFLLQTLDEGSEHHQH
>NvitOR257
METELRKYERYSRDLKWLLVLSGVWPDFHPVIQPMLGCFAVLVCSLTAIAVLNFSIHHITNF
VVMTKSCSIAIGLCLSTLKLCACLWHHDDLVYLNTSLAASFNADNQNKSFRRFTLAKVNV
FANLFYILTIAVGLVIVMGLVFMILSLLHGKYVLVWPSIFPFSYEPGGWVYWILLTVQLLAN
FFAWTVPSGVDSVFGWYTLQICGEFRVLAHKFQNLKISENYQDDLKECLERHYALMKSGE
VLQDVFGFLAILVGLSSAIIQCMLIFQAIQVFQQLSFGMMILIFAFITLKHVQVFIYAWYGQL
IADESEDCIEAMYNAQWAGSGNIRFMRDVLVVLSQKPMIFRAKGCMLLKMDMFIKVLNT
SVSYFFLLQTLDEGLQN
>NvitOR258
METELRKYARYSRALKCLLVLSGVWPDFHPVIQPMLGCFAVFVCSLTAMATLNFSIHHITN
FVVMTKSLTIAIGLCLSTIKIVVCLRHHDGLVYLNSSLTASFDADNQNKSFRRFTLAKVNIF
ANLFYTLTIAVGLATGMGVVFLILALLHGKYVMVWPSIFPFSYEPGGRVYWILLLVELSAN
IFAWAVPSGVDSVFGWYTMQICGEFRVLAHKFQNLKTSENYQDDLKECLERHYALMKSG
EVLQDVFGFLAIMVAVTSAVIQCMLIFQAIQVFQQLSFGMMILIFAFITLKHVQAFIYAWYG
QLIADESEDCIEAMYCAQWAGSGDIRFMSDVLMVLSQKPMVFRAKGCMSLKMDMFIKV
LNTSVSYYFLLQTLDEGLQS
>NvitOR260

MELEVRKYENYSRDIKRLLIVSGIWPNFYPVLQRFVAVLAICCTAMTFMGAFNFCLEHVSN
VVVLTRGMGLLFTLLSTGMKICVFLHHQKDLIHLNQHLSARFLDDLQNKAYQSHVLARLP
AFSELFYSLTYTIGSTAFLTTILIPLLALRHGKYIQVCPSIFPFEYAPGGLVYWLLQLTEALAA
FFVWAVTSGVDSAFGLYTLQMCGELRILGSKFESLRVSDKYREELRECIERHHLLMKARDS
MEKTFGLLAIWLAVSSAVIQCTLVFQAMEVAKSMNPLRIGFFFLYIVLKLLQAFMYAWYG
NLIAEESAMCLNAIYNARWAGCGNSRFMTDVLIILSQKPLVFTAKGCVSLKMEIFSKIVNTS
VSYFFLLRTLDEGSQN
>NvitOR261
MEAKLAKYARYRNVVRRLLLLSGIWPHLEDTCRLYRVLTFSATFVIAALGAKVFAYCIDNI
AHVSLFAKGMSNAFSFYTSVLCYLVYRKDLVMLNDCLGRRFEDELKREDRRPLLLQSISV
YTRFMCIVAGLTATALVFYTLVPLVFIFKYKKLTQIYQGRYPFAVEPGGRVYWCVCFVESIS
VVFVWNVVCSVDNAFGLHSFRMCGLLRSLADRFAKLQPDDPGYIVELRDCVRTHQLVLR
AKEALQRVYGLVVLWTYVTSAIIMCSILYQADQAKKHMTVTRVIFFTSYITLKLLQSFTYA
YYGSLVSQESEKCQNAIYTSNWPGSGDLRLMKDVLIIQSQRPIVLRANGFFIVSMEMFEKI
VNTTISYFFLLQAVEEK
>NvitOR262
MMMELELLRYKAYTQNVIWFLKLAGLWPESHPVPKKILSTITLSSILVIVLTVSNFSFHNLG
NIMVFTSGMCMAASSTSAFSKVALFLLHREDVVYLNKHLSGGFMRDMDEPDNRPDLLSN
VKTFERFMVTHVISVAIAMFTYSVRPLLVLRKHGKYIRSFPAVYPFAYEPGGLVHWILYAVE
VSGTASLWTVTIGVDCVFGVYALQVCGELRILSRKFRELRADDNYKEKLKDCIRRHHVLI
NAKNKLENIYGLISILLITSTTLVLCSLVFQVSELMKTNSYLRTAHLCVYLIPKFLQIFTYAW
YGNLIAEESGACLDAMYGSHWTDSCDKNFKNDILIVLAQEPLALVAMGCMVLQLDLFAK
TVKTAVSYFFLLRTMNEGSE
>NvitOR263
MMELELLRYKAYTQNVIWFLKLVGLWPESDPLPKKILSTITLSSILVVVVTVSNFSFHNLSN
IVVFTSGMCMAASSTSAFSKIAMFLLHREDVVYLNKYLSGGFMRDMREPNNRPDLLNNV
KTFDRLMVTHVICVAIALFTYSIRPLLVLRKHGKYIRSFPAIYPFAYEPGGLVHWILYAVEVS
GTASLWTVTIGVDCIFGVYALQVCGELRVLSRKFRELRASDNYKEKLRDCIQRHHVLINA
KNKLDNIYGLISILLITSATLVLCSLVFQVSELIKTNSYLRVAHLCVYLIPKFLQIFTYAWYGN
LIAEESGACLDAMYGSHWTDACDKNFKNDILIVLAQEPFALVAMGCMVLQLDLFAKTVK
TAVSYFFLLQTLNEKNE
>NvitOR264
MKTKDESLQPNIFLQHYLNINSKMLRYMGLVVRTKGNKTDSKSKILERLPTYATNLISIIDA
FFQMRWIMDLWQRDNDLVMQITTSGISNIVCICKGFRLAYCREDIQTLFEKLATIWDQTCV
PEDIRDTIVKKAQSTLVFCRCYIVMMLGLGICFALPPMKNFLIQYFARKEMNHTYDYSERV
FLVRYPFEINSSSIYFSVLFEEQWVLFCSALYWVCCDTLFAQLTTHTSLHFEILQYDIEAVVN
RENDEDRLKQSMIDFVKRHRELLRICHMIEKLFSPVIFTTMLLTSINICVNVFELREMISEAK
LGDALLHGFHLVNIFFQLLVYCIFAERLTQQAGTIANATYNCKWTEKNNKLRIYLQILIMKS
QKPFHCTAYGFFPIDHKTITIIVNRALSFYMMLETTN
>NvitOR265
MKTEDKSAPLTPDFEDYTKINSLFLRCMGMGIGTDGNKKDRRSQIIERVPTALINVLCLLD
SVFQVQWVSELWKTDKKLVLQILTNALSNIVCLCKGFQLAYSREDLQTLFEDLAMIWRKR
IPHHEIRDEILRGAQKTLVFCRCYISMILVLGLCFGLPPLKYFILQFTDRNANRTYDYTERIF
LVRYPFDVNNLTAYNFIFMEELWVLYSAAIHWMCCDTLFVQLTSHTSLQLKLLHYDIEASG

NTEDERQFKENVMDIIKRHQELLRICDLIEDVFSPVLFVIMLLTAMTMCVNLFELREMLLE
AQYVGAILHSFHLINVIFQLLIYCVYAETLTEQAGSIAEAIYNCKWTENSHEVRTNLRMCI
MKSQKPFYCTAYGFFPIDHRRITYIFKTAMSYYMMLHQTTS
>NvitOR266
MKTEDSPSVTPAFEDYTKLNSLLFRCMGMGIGTDGNKRDKRSQIIERVPTVLINIICLLDFV
YQMQWINDIWKTDKKFVLQILTNALSNFVCLCKGFRLVYNREDLQTLFEDMAVIWRRRM
PRHEIRNEISREAQKTLVFCRFYVIMILFLSLSFCLPPLKYFILQFTDRNANRTYDYTERIFFV
RYPFEVNNLKVYNFLFIQELWVLYAAALHWMCCDTLFVQLTSHTSLQFKILHYDTETSDN
TKDERQSRKNIVDIIRRHQELLRICDAIEDVFSPIIFIIMLLSAITMCVNLFELQEMFLEAQYA
GIALQSFHFMSVFFQLLVYCDYAETLTEQAGSIAEAVYNSKWTENGHVLRMNLQMCIMKS
QKPFYCTAYGFFPIDHQRITTILKTAMSYYMMLYQTTS
>NvitOR267
MSQPTEDDLEYYFAFNLKLLALVGFKCSMDKKEKGLGFVNKLPSYIMCIQGTILSLFEVYL
LRDIYKDEDKTIVMQVLSQGVENTLNVCKGFFLAYSIERMENVLQEIKFLWNTYRPSPDN
RKIILAEAQQTYSYCKIYFCVLASCCTSYFLCYLPALFKLAQQYRDREANNYTYDFSQRLL
LLKYPFDIPSIPIYFLVELQEGFYLFYAAALFFVSGDTLFAQTVTHICLQFKILKFDIDAMFNP
ENTGEKDHLNLVTFIKRHRDLLRVCALIEEVFSPIILSMMLLSSIALCVDLVGIRGTMEKNN
YEETAVVITLMMLTLLQILFYCTFAEKITEETRSLADTMYGCNWTMKNNKLGLYIHLMILR
AQKPFQCTAYGFFPIGHSQLTTIINTAFSYYMMLQTTS
>NvitOR268
MLPPTEDDFEYYFAFNLKLLSLVGFKCSLEKNEQSLSFINKLPSYMMCMHGIILSMCEVYF
IRDIYSNENKTLVMQILSQGINNTLCISKGFFLAYSIERMQNVLQEIQFLWKTYRPSQDNRK
IILADAHRTFLFCKIYFCVLASCCSSYFLCYLPALFNLAQQYRNRDANNHTYDFSQRLVLL
KYPFEIPNIPTYFLIELEEGFYLFYSAALFFVSGDTLFAQTVTHICLQFKILKYDIDETFNSES
TGERDHSILVNFVKRHRDLLRICALIEEAFSPIILSMMLLSSLSLCVGLVGVRGTMAKHSYE
ETAVVVTLMMLTFLQILFYCTFAEKITEETRSLADAMYSCDWTVKNYKLGLYIQLIILRAQ
KPFQCTACGFFPIGHSQLMTIINTAFSYYMMLQTTS
>NvitOR269
MKTSVNQPTEDDLEYYIGFNLKLLSSIGLKCSLEKNLKSLGLINKLPTFIMCIHGLIFFIFEN
YFIRDIWSSDKTLAMQILSQEVSNIQCISKGFFLAFAIERMQNVFQEMQYLWKTYRPSQDN
RKKILLGAHQTFSFCKIYFFVLLSCSISYFLCLIPSLFNLAQQYRNREANNYTYDFSQRLGL
VKYPFEIPNIPTYLLIVFQEAFYLFYTAALFWVSGDTLFAQSVTHICLQFKILKYDIDATFNR
EDMRDHLSLVTIVKRHRDLLRICKLIEEVFSPIILSIMLLSSLNLCVNVVGIRGTIAKENYQE
TAINVTIFMLTFLQILFYCTFAEKISEETRSLADTIYNCDWTVKNYKLRFYIQLIIMRCQKPF
YCTAYGFFPIGHLQLTTVLNTAFSYYMMLQTMN
>NvitOR271
MKMTAINPEDYFGLNIKLMSLCGLRCSMTKTIGSFINKVPTFLANLVGIIYLVFQATFVMEA
VRLRDVALTSQILSQLVSNIQCITKGFLFAVSIEKMQSILYEIRSLWERYQPDIEIQESILDDAD
RTLNFCKYYVIANFSCVLAYALPLVLNLFMQYQARESTNHTYDLSQMILLVKYPFEVTKV
SRFIILVLLEEYLLVVSVIIWVSSDTLFAQTTTHICLQFKVLKQDIEKTFNYGGPNSKEILLKL
VHRHRELLRMCMLLEDVFSPIIFFTVFLSSVNMCVNVIGTRETISDKTYLNTGIYATILTMTI
FQILFFCIFAEKISEETTSLADMVYNLNWTAKDNQLGFYIYFIIVRAQRPFYCTAYRFFPIGH
QRLTSIIRASFSYYMMLQTTDNK
>NvitOR272

MKMTSINPEDYFGLNIKLMSLCGLRCSMTKTIGTFINKVPTFLANLVGIIYLVFEATFVIEAV
RLRDVALISQILSQLVSNIQCITKGFLFAVSIEKMQSILHEIRFLWQRYQPDEEIQESILDNAD
RTLNFCKYYVTANFSCVLAYALPLVLNLFMQYQARESTNHTYDLSQMILLVKYPFEVTKV
SRFIILVLLEEYLLVMNVIFWVSSDTLFAQTTTHICLQFKVLKQDIEKTFNYGGPNSKEILLK
LVHRHRELLRICMLLEDVFSPIIFFTVFLSSVNMCVNVIGTRETISNQTYFNTGIYATILTMTI
FQIFFFCIFAEKISEETTSLADMVYNLNWTAKDNQLGFYIYFIIVRAQRPFYCTAYGFFPIGH
QRLTSIIRASFSYYMMLQTTDKK
>NvitOR273
MQTNTEEKAITAVDAEYYFDLNIKLMSLIGLKCSMTETVTKFIYKIPTFLTNVLGIIYLIFQIS
YVREAVRSHDTSLAAQILSQTVCNIQCNSKGFLFVISIAKVQAILHEIRILWETYPPDDEIQK
SILLVADKTVTFCKYYVTANLSCVLAYALQMGLNFFMQYQAREATNHTYDFSHIILLVKYP
FVVTEIPTFITLFLSEEFLLIMGATLWAIIDTLFAQVTTHICLQFKILKRDIQEKFNTEGSNDK
EILLKLLRRHRNLLRICMMIEDIFSPIIFFTVILSSVNMCVNVIGARETIASKAYFETCIYASIF
LMTIFQIFFFCIFAEKLSDETTSIADTVYDLNWTTKDYKLRLYLRFIIVRAQKPFYCTAYGFF
PIGHQRLTAIIRASYSYYMMLQTTDGK
>NvitOR275
MQSKERDKPKVLDIEYYFDLNIRVMSLIGLRCDGPKITGFVHRIPTYTSNTIAILILIFEICLM
SDPVCSSNMELTIQTASQTVSNIQCVSKGFLFVNAIEKLQVVYNELQVLSQKYPLEDEIQVL
VFDIAEKTMNFCKYYAIAICSCILFYYTPIVVNVIVYILQDPSTNHTFDFTQTLFYLKYPFTI
KTFPIYSTIVSIEAVNLIAQGIFWFLGDTLFAQVTTHICIQFKILKHDIQKTFNDEGSKSKEILI
GLIKRHRQLISMCMLTEDIFSPVIFSVMILSSTNLCVNIIGASTAINDGDYMNAGVYATILLIT
VFQIFFYCIFAEKTTEETRSLADTVYHLNWAMKDHHIRLHILLIIMRAQKPFYCTAYGFFPI
GHQKLTSILSTAYSYYMMLRTTANV
>NvitOR276
MDYKMQTKEETIEVNAQYYFSLNLNLMSMIGLKCNMTENVGRFYHRIPTFITNVCALMY
QSMTVYYLVEAISAKNTSLSIQIISQLVSNIQCFTKGFFLAFGINKIQFILQEKQILWKKYPPN
NNNHHTILGIAQQTLTFCKFYVVAIFSCVMSYDVPLAINIFMQYLKRESTNYTYDLSRRVIL
VKYPFEVTEISTYVILCLQEALFVFIQCIFWVNSDTLFAQVTTHIGLQFKILKCDIEAAFNRD
DAKNKEILIELVNRHRELLRICMLIEDVFSPIIFCTVFLSSINICVNVIGVRETISEKAYLDTGI
YFTMLLITLFQIFFFCIFAEKLTEETRSLADAVYNLNWTIKDYKLRVYINLIIMRAQKPFYCT
AYGFFPIGHQKLTGIISTSYSYYMMLQTTDK
>NvitOR277
MKTLAKVQSTKDDIEYYFGFNLKLLSQIGLKFSMNEKTDKFTFLQKLPSYIFLVEGMILFIL
EVYLIRDTIQSDTLLSIQIMSQIISNLQSVSKGFLVLNKAKSIKNVLETLGIIWRKYPLNNSD
RALLNAPSKIISLSKIYWGIAVALLVIYDLPPFVIFFMQYQNRDAMNHTYDLSQTILLLKYPF
NITRKSTFFFLISQEAFVLYASGVYWIGSDALFAQFTTHICLQFKILKCNTKEVFNRGSKEA
HSSLIDLIKRHRELLKICEMTEEIYSPIIFSTMLFSAINMCVNVVGVRETITRGFYQETGVYL
FLFLVTFAQILLFCIFAERITEETKSLADLAYNLEWTKEDHKLRVYILFIILRAQKPFSCTAYG
FFPIGHKKLSSIINASFSYYMMLQTMS
>NvitOR278
MKSQENIFTKDDIEYYFDFIFKSLNTLDLKFSISKKTDEFKFRHKLPTIIGCLIGLIIFFLEIYFI
RDALHNHTILPIQIFSQVISNFQSISKVILIVYKVNKIQQILEKIGVLWKTYTPDEGNRAVLY
NTLQRTLSICKIYYAVLIATVLIYYVQPIVNFVGQYGARNSINHTYDYSQTLVIIKLPFKVTQ
KRYFFVISQEAYLLYMSGVYWGCSDTFFACFTTQICYHFKILKYHTKAFFDEKNNNSRLNL

VTLIKRHQELLRLCVLIEDVFSPIIFSTILFSAMNLCVNVIGVQETILNGSYRQAGIYLFLFIIT
FSQILFYCAFAEATMEEAWSLADLAYNLEWTSKDYKLRYYIHVIILRAQKPFHFTAYGFFPI
GIQKLTSIINASFSYYMMLQTVS
>NvitOR279
MKSRENIFTKDDIEYYLNFILKSLRTVGLKLSLSKKIDEFKFRHKLPTIIGCSIGIIIFFLQIYFI
GDALHNHTILPIQIISQVISNLQAVSKGFLVVYKINKIQRILEQIGVLWKMYTPDESNRATLY
NILQRTRSICKTYYAVLIATVSIYYLQPIANFMGQYGARNGINHTYDYTKTLLIIKVPFQVTL
KRYFFIISQEAVLLYMSALYWACSDAFFACFTTQICYHFKILKYHTKVCFDVKNENSRLNL
VTLIKRHQKLLRLCELTEDVFSPIIFSTMLSSAMNLCVNVIGVKETISNGSYRQTGMHLFLF
IITFSQILFYCAFAEAMTEEACTLADLAYNLEWTSKDYKLRYYIQVIILRAHKPIYCTAYGFF
PIGIQKLTSIINASFSYYMMLKTVS
>NvitOR280
MKTQENDLSIEDDIECNYSGYIFKAFHFMGLKLSLKKKTDGFKFVHKLPTTIGILQSIVVFF
LQMNFIRDVVQCDSNPPIQIISQVISNIQAGLKQTLLVFKKIEDIQRMLETLGEFWKKYSPD
KNYRVVLFRELGKTSSLCKYYFGTLVGIMIAYDVQPLVYFLTYYFEQNATNHTYDLSRRIL
LVKYPFEITRKSTYCFLLSQEAYLLYITAIYWANGDTLFAQFTTHICLQLKILKYETGKFFNQ
SNQEGRSDLLILIRRHQELLSMCDMIEDIFSPIIFSTMLLSAINMCVNVIGVTETIAAGSYEE
TGIYTFIFIATFLQIIFYCVFAETLTEETRSLSDFVYNLEWTSKDYRLRFLIQVIILRAQTPVYC
TAYGFFPIGHQKLTSIINASFSYYMMLQTVK
>NvitOR281
MKSQEDTKDDIEYYLGFILKSLHTAGLKLSISRKTDEFKFYHKLPTIIGCSIGIIIFFLQIYFIR
DALHNHTILPIQIISQVITNLQSISKGFLVVLKINKIQRILEQIGVLWKSYTPDESNRATLYSIL
QRTLSICKTYCAVLFVTLLIYYLQPIANFLVQYRERNGLNHTYDYTKTLLIIKVPFQVTLKR
YFFIISHEAVLLYTSALYWGVSDTFFACFTTQICYHFKILKYYTKVFFDVKNNNSRLNLVTL
IKRHQDLLRLCELTEDVFSPIIFSTMLSSAMNLCVNVIGVRETISNGSYRQTGMHLFLFVITF
IQILFYCTFAEAMTEEACTLADLAYNLEWTSKDYKLRCYIQVIILRAQKPIYCTAYGFFPIGI
QKLTSVINASFSYYMMLQTVS
>NvitOR283
MQIKVVENLTLTKHDIKYFFKENLKLLSKIGFKCSLTKKSEKFKFHHKIPTYIANFCGLIVFA
LQIYFVIDKIQTNTVLAMQSLSYAVINVQSILKGFMTANSIENIQQIFENLGIFWQKYMSRK
PGRELILDRAYKTISLCKFFFVMAIVCYFLFVMQFLIKFSIQYLNREATNHTYDFSNTVDLI
KYPFEIPNLPVYFLLISVEINYLFVCIVFWCNTDSLFVTLTSHVYVQFKALKLDTTLAFNNS
MLKERSILIDMVNRHRELLRMCYLIEDTYSPIIFSTTLLSALNMCVTVYAVREYIDKGYYLE
MGIPLFLFIGASLQILFYCIFAESLTDETRSVADSVYNLKWTTKDNKIKFYIQMIIMRCQKPF
YCTAYGFFPIGHQQLTSIISAAFSYYMMLQTMSN
>NvitOR285
MKIVGEKSSPNKDIEKYLGLNLKMLSCIGLDVSLENDDVIQERRILEKMPIFMTNGLGIFA
AILQISLITDSMTHNRMFLATQVSSHLFSNMLCISKGYQLATAIAKLGEILREIALIWKQNPL
NDEFHRNILSDAAKTLLFCKVFVVVTLCAVFGFGLPPLQNLFFQYLHARNSANHTYDYSQ
RVFIIEYPFPIQDVLTYSSVLLEEEYLLLASGLYWVCCDTLFAQLTTHISLQLEILQYDIETLI
NRESAEDRLNENFIIIVKRHRKLLSICELIESVFSPVILTTVVLSGMNICMNVFELSKTISEGN
YAEAALHAFLFMNTFLQIVFYCTFAEKLTEQTSFVANSIYNCKWTEKNCKFRVYLQMLIIR
SQNPFYFTAYGFFPIGHKRLTTVINTAFSYYMMLQTTS
>NvitOR286

MTLLHETVNHPPQNNDIREYLGLNLKMLSFIGLEFNLNNDRPIKKSKFMQILPIFMTNVVS
LTIAALEITFIAFVLRNHEEHLAVQICSELFSNILCIGKSLRMATAVASIQTALDEVSILWAKH
RPNQHCKMEIMKKARNTLNFSRWYLGFIITGIAGFALPPIHNFVYHYFIRDANNYTLAFSK
RIFLLRYPFEIKNVPLFFFVLTEEGYILLISAMHWVTCDTLFAQITTHTSIQLKILHYDIGALI
NHETVEHRLKAKILIIIRRHQCLLRVCRLIEDIFSPVILTTVLLSALNICVNIFETKAMNAEGN
YARAALHANLVLVLFLQILFYCSFAETLTNQTSAIAESVYNCKWTEKNHKLGFYLRMIMM
KSQSPFYCTAYGFFPIGHARMASIISTSFSYLMMLQSMS
>NvitOR288
MKKTILQEYDKENQKAFDEAKTLITWNKYLMSALGLWPSHRYDFIFVSLFCYYIFHFLLD
YAAFYFALRSFNLIKIIGATMENVTMAQIFLRLYTMRRYNRQYGEILEEFTRDFSVKNYKSE
EERNTFLSYNSRSKFFIKIVVIFLGVTAILYFTKPLIRQLSLSKNVNTTKAFTYDLPYRIHLLY
KITDIQTYIATYISRIPILYIIGFTQTAMDCLTLTVIAHLCGQLGVLSIRISNLDVVNKSNELNE
IIQRHQKLIKIGLRLRRMYRLCLLGHFLGATIAICILVYQVLISIAAGQKTNLVTFFVFGFLNI
FRLYTHCWVGEYLIHESINVSHAYYRCKWYKLPLKDQKSFIICIKRSQQPLSLMAGNFSHY
SLVMFTNVMKSAMAYLSFLRNFI
>NvitOR289
MMQNHQLQGQAELDDSSQVFRYNYILLTTLGLWPASLSDVRFFLNFGYFCYEMLLEYLD
LFLFIDNFENVLMNLTENMAFSQIFIRMLMLRIYNSELGEIIGDAKKDFDAKNYTEEERKTF
VAYHVKSRTFMKLLITNTALTASSYYVKPLLGQMGELMEYANSNGENSTFIFMLPYRFYTF
YELNDAQTYFWTYGSQLPFVFISGFGQSAADCLMVTLVYHVSGQMAVLALRIASIDTHPS
KCTQEVQKIVKAHIRLLRMGKVIQRTFSATLLGHLVGATSLVCILGYQILTSLANGERAILIS
FFAFIFLVLLVLYAHCTVGESLITESERVSQAYYDCEWYNMSKENARIIILCMARSQKPLQLT
AGKFSMFCLQTLTDSIKASMGYLSVLRTVM
>NvitOR291
MNISGSEQTILAKYKNDLQKASKILTWNRRLLSLLGLWPESPMDLLFCASAVYYIFYLGLIF
VSFVLYLKKKILNVSIFIALLSYGHISARLLLLRRHNRTFGVLFAEMKQDYELRNYKSDQE
LRVFLKYNILAKSMIKFLLFCSTFFAIVFYVKPLLMTYNIHRAIRKSHRNATAPFVLAQNSF
YQFYKITTVKKYAINYVSMLPFSVLTGFINCATDCLVLTIGCHLSGRLAALSHRIRNVEFCN
GSQEFKAVIRLHQQVLRIGDMVENSLNTLMTCHILTAGVIMCFILYKTLIYLRPGKRIHLIHI
VILLSLNIVRLYFHCCVGEFLMQESRVVHEAFFECKWYTMLLQDRKLIVLNLLRSQRPIRF
AARGLGTFSIELFSEVLKSSLGYLSVLRNVI
>NvitOR292
MNLYESDVNQKSLTKCKDDLKNASKILTWNKRLLLLLGLWPESPMDFLFCASAVYYIFYL
GLDFVSFVLFLRKKILNVSIFIQLLAYGHISARLLLLRRHNKTFGILFTEIKQDYELRNFESD
QELRMFLKYNRPAKTMIKLLFICSTFFGVVFYVRPFLTTYFVHRAIRKAHRNVTAPFFWNT
YFYKFHKITTINVYAMHYVSEFPFSILTGIISCATDCLVLTLGCHLSGRLAALSHRIRNVNFR
NGSQEFKAVIRLHQQVLRIAEMIEDSLSSLMLCHILVASILMCIVLYKTLICLRPGKRIHLINT
VILLFLNIVRLYSHCCVGEFLIQESRAVQAAFYECKWYTMPLQDRKLIILNLLRSQRPIRFT
AGSLGTFSIQLFSEVLKSSLGYLSVLRNIV
>NvitOR293
MEFVRKAYGDKQRKRQASSKCISRADRVFKRCVFFHKFVGIWLEKDRSQRLLDRLKGYV
SAAFTLGICIFQIVMLSVESSSVVTVLQNNLLILIRKTKEIAAPLHASKIKERKIVDRWLNNQ
DKILKKILLTSYTFTFSSYSLFPLLKENGLPFTGRLPAICYVNPWYPTIFAAQLVFIIFRFFCVLS
NDILCITFLCQLCSELELVKHLIVELGNGKDRNVKQIIIRHAMVLDYGEIICETYSATLIMQH

LNCSIFLCLSGLVTMKTSDMFALLKIGSLSLIGIITMLIICFVGEMVMSSSLEIASTIESSVYK
DYRNDVANLKLLNFMLMRAQKPLCMMVCTQGKLSLRFFSENINKVASFFIYLKTLVE
>NvitOR294
MNIKSAESSLQSTTFEYEAIFHKVVGVWPGDDYFLARYSRIRGYVLAAFAVVVCVFQFTA
LLEANSDDVPENDFINLMRMTKEINEFSTLTDEEETIHIDWQSVQDKLMKIISRYYFLTVIG
LYFVAPMFRNALPLRGIVPEVLRVTPWFQMIYVLQCLLLLSNVITSISSDAFSVTFMCQLCK
QLELVQCSIKHLGSHTKVNLAETINRHAVALDYGQRVCNTLKTMFLLQHIFISMFLCFAGVI
VLNTQNSLILMKMIVISVIFVSTLLIICFVGETITSSSLKIASATESSNYEIFLGDVSTLRTVSFI
LCRAQKPLRMAVSLSGSMNLSFFTETMNKLVSAFMILRTMME
>NvitOR295
MGLIEVLESRKIFLWICGLWPKEYQHKPKLSQMKLYFIWFNMLMMCLLVFAGLVAIATPD
NIPQASIRLPRKRMMKVIMETLKLEETSKFENDNDLEIIRSWRRTDGVLKYHMRIYGFISVA
YCFLPIISQVNTYPAQTIIQASLFVSPWYEFFYGFHCAQLFLYLFIIIATDGLSMILIFKLCEEL
QRFECLLFERHRADDATLSEKYKSRGEFLRCIIRKHCTILDYGESICNLLTGALFTQYFLLS
GTLCFSVFTILSSNSSAMANQMSIMAGTCIVQLFMISLAGELVSTRSLALADALLQSDFCCS
IFGELKSSELRQTVLMQLRMQKPLKLSIGTLGVINIEFFSRIMKGVYSFTMLLRTSYV
>NvitOR296
MYVKLLSTREPHGGDASDGQLINTELRPKHTNLLYRTQAKIKMNVGEEYDKLALPMTLS
SRVVGSWPSRAELEGQGGRSVLVHRLHRYLAIVSIYLMSMGVAAEVIVFFGEDMNETIEC
ALISSAFFMALTRIITFASHQPEMLYVVETMREDWIRSTDEERAILRDKCLFAFKLAKFFAIS
VTITCSAFILMPMLELKFVENAKRMLPYRGYFFFNHTVPGVYEYVYLVNSMLGVLGCSTI
ACATSFSLITSIHGAAKFAIVQKDFERIDQVTWNNSEIVGRCVRRHQECIRFAETVENIINVL
ALAQFVISTGLICFAGFQMTTMLTDRARFTKYASFLNAAVTELFIFSYGGQSLKSESEEVAE
GVYSSNWIGSALSSNLRLIVLRSRKPCTITAGKFYDMSFESFLKVLSSSFSYFTVLLAMEEE
>NvitOR298
MLGKSSLNSKIPIRERDFNYSMKLSRITLSIIGLWPFRENIRCSNFKFVVILVSILMTLLSSLTF
VYQTDDDDKMFHSLINSLYMLMTLVKLLMMRCKNDKLEVILSEMRIDWRKYERFSDGN
KRLVDLYTGKARTSSFVCIFFMEFSITTYFISRVAYALQQPAKIREWDLPYTAVYPFEVTSSL
FVPMYLWQVFSAMCLGSVTISIDCLLVTTACHATGQLAALCENIKSYGHEQRHRDETLSSE
IECSCIRCIIERHVDIVRYCRLVEDAYNLILLTEFIGTTFQFCLQMYIIVEHSHDKNIVGLLSFC
IYLLVFNFRLFMYCNVFDAMVEMGEKVGASAYDISWYDFHPEAVRQLMFCILRANKPLN
VTAGKFFSLNRNSYKNVIMTSSSYASVLLSIK
>NvitOR299
MKTESSNARLKGKNVTFCTEVFECDEILQLDNELGLIQWSLKLMGIWPFWTRFSNVKFFL
CGAILAFNVVGCFSGILNVNSDIEQFIECLLYFNVNLATLLKFLIVKYKRRSIEFILRCILDDC
SRYSHLSVSCRSRVAGNIKKRKLMTLTLALFILAPVAGKRSFLSMLWSTAYIFSAFAITKYIE
YRDDLAMIRELPIFSALPTFVRHSQIFYLALLSGLFGILMSTLVIVTIDSVFAILMIHATNQFI
VLSEELKAYREDHLDACYKISKNMRNCKCMRCIIDGHVNILRYTLNFYWYSKFILYKIDSV
FSIVHIAVILLSMNLHLFMYCVTSKSMTDASEQIGIRAFKMKWYSFQKTTVRSIVLMTLRS
QIPCYVTVAKFINLSLETYTSVLKTSISYASVIIIAREHLVNER
>NvitOR300
MGKGKVRSFSDYFWLSQGMLKFCGVLPMPERGLFVNYFLIMLSISSLVFLFFPGFYIIAFH
GSEINAAAKVDIIAGEALEIWVTTIKALVLLPCRQTMLSVSRRATRLLVDIEDEKEQQLAEP
YARRGYYLLYGFGGTVFFALLSIVIKPFGQQVQYGANGTILASKDLPYSIGIVHENQQLFN

AWWIGQCFAGIIAIIAIIGIDTTLAIFVLHACGHFRILRSRFQAVAENSSSRRMSVSGRDDRR
RLIDLIDKHQEIIQFVSTIIIIRYVITLRKCFLFSIKLLRRNRIRLQSGGTDSNDTQHLSDLRIRL
QFAGGKSLYIYIYTSSQVGFEAYNLRWYDWIEDDKSLVTFLITRSQKPMLITAGRFTSISLET
FSAVLSSAFSFFSILRKTL
>NvitOR301
MFVRIIILNEINFLGNMDSEIYDSEYYHEVKLLLTYFGLWPNLSRFRKVVSFIAMVAMPISL
VIPMSFGLKRAIRLKEPIQIIEDTIGILYFLAITTKYICTFIFEGRMIVVYEQIASDWKKIKDKN
ELEYLHGRAKEGKIITILYLGYGAVGCTIFASTPYLPLFLDLVIPLNVSRDKIYPYYADYDIV
DSEKYFYTLYTLHGIFIIILVTMSAISIDCLFIMMVKHSVGLFQIVCYRLKKIGEEHNEKPHE
CKRLMDDKIIHTRMKEIFDSHKSSIECVDAIQASFDVSFLFIMTMSGVGVSLILFDLLLNLD
DLTQILRINSMMFGVYIAVFVICYAAQMTLNSSEIVFNDTYCGYWYNISPNARKYTQMVM
VRSMKPCIITAGGLINMNLQSFFAILKTSVSYATVMLSMQEESNMQN
>MmedOR9a
MVTSLVQEMVTPGSSRDQEVLELYVAIQAKDIDEVIDILPHIFVVIASLIKFGNIYLKKDRFK
ILLDLIVKDWETLTNELHVLDKVTATGDKLAYLYRMTLLSFLVAFNYIPLIPPTLDIILPLNE
TRPRQQLFQVNYIFFDVDDHFFAIYLHMSWAGSLTVFVIVTIDSLYMLIIHHASGLFDVCGY
QVQMACKQKKMEDEMFKQCVITHHKALELVFDFLEECSQIMNLMLVGMNMIAISLTAVQ
IILYMDQPMDAMRFVLFLIAEKFHLFILSLNGQILLNHAVALTDKIFSSNWYDIPVKYQKSL
YMMTIRCSKACMLSAGGLYDMNIENFGKTVKACISYFTMFLSVRG
>MmedOR13a
WLRCVTLSFTMWSLLFGIYLQTTIMYHSLDDVENLIFGLLNLLSVLVPLIKMLALLPRRKK
LFGLIAYMVRNFLKADYDDFETSILTTCKRKCTFFVCGSVCFTELTIVSYVCAPLLVNLFM
NESERVLPFKMYVNLPIQATPYFEIAYITQVLALCPVGFSYFSLDNVLCIINLHIAGQFRILQ
YRLSDKYSGQVQNGLDQKSDLYLKNNASDVFKSCIRQHQTLITYCKQLQEVFGLIVLVQV
MTFSMLICLDGFQVLLVDLVQRKVIFFFHLLTTVCQLIMFSYSCDCIIRESVNLATAAFSGP
WLQLPASKRIENLKKDFIMLIMRSNKPCCISGSGFFIVSLETCTRVITTAGSYFTLLQQAQN
NISS
>MmedOR2
MGMEYTSDIAVRLTIFYLKVVGFWFAANRLQQLFRCLTVIYTIVMSVFALWIQSMGIYYC
WGDYTMCTYIFVNILGIAISLLKLCLLLVQKEKFLRLIEFMQRNFWHSNYSQKEIKIFAGTK
RICIYFVCSFSFISQLTVLLYSIRPVLLNIGKNESERVLLFHMWLDLPLSTTPYFEIMYVLQV
LSLYQCDVGYICFDNIFCVMCLHSAGQFRILQYRLKNVHQLASKHEDNSNNSASYFSYKC
FVVFKDCVRQHQIIIAFCKLFEEVFKVIVLCQVIMFSMLVCLVGYQIFLVNLNLPMRVSLTS
FIISNLCQLWVFTYSCDTMTRESLSVGTAMYEAPWPQLPTDKFGKMIRKDMQIVIMRSKR
GCHITACGFFPISLETYTKIMSTAMSYFTLLKGSTIDVDT
>MmedOR1
MENRTKLKKIKPNKHLENSLSIIYYMGMWPSESKYKRLYMLYTVFSFMFLLGIFLASQIAY
IIVNRKSVDKIIAGATLLMTNATHAYKAILIICHHKRIKDLTDITRSETFIQDNGKYEKIVRH
YTWQGVFHHIAYQSFGLMAVISWGVTPVLNLLTQRSKELSMEGWYPYNTSSTPAFEITSSY
QAVAIFLCCINNVAIDTLITGLITIACCQLAILSSNIASLNCAENTEPIGINNNTDLEISTSKNY
NKLYEDLKSCVEHSNMIFDFSKQIQDTFGTVIFLQFLVNCIIICLIAFNIAQMKDYIPYVLCG
MLMYMCCMTYQIFIYCWHGNELYLHSMNVTFSAYANNWWYNSKDFKQAICIIMVRVQQ
PLILTAGNVMQLSLQTFVRILRMSYSIFTVLQSSANS
>MmedOR82a

MKKPISLTIESFFDENVLSWSKRLLSLSGLWPENRNDVQFFFYITYVVIFTWLEIVTLLQNIH
DLERSLKNITLSFPTILIVLKAVMFRLNMHLVLPLLALVKRDVEQGLYQTQEERQTAVWYN
VAATLFSTSSALSLFFVPTLFYTKPVVGCFLSQFVNCTLPYELPMKVNHIYEVTEMRTYALF
CVYLIPVSMMLTIGATGADSLLVTLTFHICSQLSILSQRVRSIDLEPQIYFPKMKILVERHTEL
LRLANVLSETFSSLMFVQTLGLIFSLCIVVYQLLMTSESGEDMNTVHFIIYSCAVVLLAFCY
CFLGECLINESSEIQAACYFTNWYNLPDKYVRSLMFCIARSQKPLYLTAGKFYVFSLETFG
VIIKASMAYLSVMKSMV
>MmedOR63a
MKKQSNNSIDYYILPNKILCTTLGISLSDKKRSLCGQIFAYLRLFVAVASISSFVVPQAMLLFI
KWNDLKILSEVGGILTTLAQFEFKLIYLAIRREKTYKLYKEVRSLWNSTDDPEEKRSYEEFA
YWARRFTIIFYSFGTWTTVIYTASAAVDCIIIQYSANNDTITRSLPFDVWYGTNVSESPSFEI
MFTVQTVSAIYNSAAVWGIETSCMTVILHVSGQFKLIKTWINNIGVKIKNEPKDHYYKCPP
DIEDGLVRCIRHHQRLVNVVNELNDLLIPIIFIQLLTSGIKICLSGFAVMNNNTNAELIKAVLY
LFGMTTQLLLYCYPGEILIRESEEVGDAAYLNVCWYKLPPSNRRQLLLTILRAQKCCSITAV
TFQRLSFRTLTGVFNTAASYFTLLRQMQETSM
>MmedOR92a
MEKVSKDTIDYYILPNKILCSMYGIWPSTEERSTIKKVFSVLHLVLSVMIACSVLVPEIMLIS
SNWRDLSVVAGAGSLVVTVGQFLFKTAYLVAKKEKACRLLDELRSLWNSTDDPVEKKSY
EVFAYWGRTCTIAFFVSGMSTTSMFMISGVLDSLNQQDLDNSSNRYLPYDVWHEMEYSK
SPEFELLYAGQIMSSFISCFGLCGLDGMCLTTILHVSGQFRLITTWLNNIGIEMKCKPIDLHN
CPVKLTADLVRCIRHHQRLINVVEDVNTLLAPIIFVQLLTGGIQICLSGFAVLSNNVGDDLVI
FIAYLASVTIQIVMYCWPGEILIQESQKVGHAAYLNVPWYQLPLFHRRQLLLIIIKSQKYCC
ISALTFKSLSSHTLTNVFNTGSSYFALLRKVQERSM
>MmedOR22c
MGKVAKDSIDYYVLPNKIMCSAIGIWPPDEEQSFGGRLFVGFRVVFSIAAVCTIFVPEIMMI
AVNWGDLRILTGVGCVLTTVAQLIFKMIYLIARKERSYKLYKELRSLWDSSHDSKERQCYQ
GLAYIARNCTIIYHTSGLLTVAVFTVSAVFDYVKFGQDNNAANRHLPYDVWYGTDVTDSP
GFEIAFACQVLAASICTIGVTGLDTTCATSILHICGQFRLMCMWISNIGIKINCDSPRTVTTD
LIRCIRHQQRLISAVKDVNNLLTPIIFVQVLTSGIVICLCGFAVLRGTGDDLFKFIVYLTAVMI
QLMFWCWPGEILIQESLEVGYAVYLNIPWYNMEPACRRQLLLVILRSQNVCSISALTFRTV
CIHSLTTVFNAAASYFTLLRQMEEKAMSK
>MmedOR7
MMKFKQQGLVADLMPNIKLMKASGHFLFNYADGSGKSMQKIYSSVHLVLILMQFAFCGI
NLVQEREDVDDLTANTITMLHFTHTIVKIIYFAVRSKLFYRTLGIWNNPNSHPLFAESNARY
HQIAIKKMRILLLAVMGSTVLSTLSWTILTFIEDPVKKVTDPVTNETMFVEIPRLMVRSWY
PFDASHGMAHVMVLIYQFYWLLFSMASANLLDVLFCSWLLFACEQIQHLKNIMKPLMEF
SATLDTVVPNSGDLFKAGSATQNQVPDQEPPPPLTPPAGDNMLDMDLRGIYNNRQDFTAT
FRPTAGMTFNGGVGPNGLTKKQEMLVRSAIKYWVERHKHIVRLVTAIGDAYGIALLLHML
TTTITLTLLAYQATKIHGVDTYSASVIGYLLYSLGQVFMLCIFGNRLIEESSSVMEAAYSCH
WYDGSEEAKTFVQIVCQQCQKAMSISGAKFFTVSLDLFASVLGAMVTYFMVLVQLK
>MmedOR43a
MENFGSILQFVSASREQDIDTMMECVPVSATLLGTMIKMINHNNNRKRFENIFNLMAELW
ETAETNGEVHVLNEITEQGSKMGSLYRKSIWGFVMLFLCVPLFYPLLDYVVPLNETRPKA
PLFKLNYLVNTDDYFYTVYFHIALCCLITVLIVTTIDSLYIVIIHYTCGLFAVCGHQVKKAAE

IEGRNSHSRGRNQDLLKRCVITHYKAIRNHLICYTLRFHEYMEESTRMSYLFEVCLNMIG
MTVTAVQTVMYFDKPEEAFRIVMFLLGQQFHLYIISLPGQMLIDQSLQLVNDIYFSKWYQ
MPVQFERTLHIMLIRCSRPCKLTAGGLYEMNIENFGSIVKTSMSYFTVLLSFRE
>MmedOR49a
MLNLRSNNGEHPLKTCIAKYHRNERQTYDVSRIPCLRVFKKLLMYMGQYPCQTKINSQIR
VTLLIVTLISLLLPGVSINNQEVLQFLSAMQEQDIDTMMECVPISATVLGTMIKMISHNSNK
KKFENLFNLMAEVWETAENNGEVHVLNEITEQGSKMGSLYRKSIWGFVLVYLGVPLFYPI
MDFVAPLNETRQKPLLFKLNYMVNTDDYFYTIYVHIVWCSIIIVLIVTTIDSLYIVIIHYTCG
LFAICGHQVKKAVEIEGRNMHSSDRNQDLLKSCVIMHYKAIRFHEYMEESTRMSYLFEICL
NMIGITVTAVQTVMYFDKPEDAFRTVLFLLGQQFHIYIISVPGQMLIDKSLQLVNDIYFSSW
YHIPVQLEKTLHIMQIRSCRPCKLTAGGLYEMNMENFGSV
>MmedOR94b
MQTLKWSYKILDILGFSLTSHWTSLWRRALYNSYGMILVISLHFMSATQMLDLFNVTNQE
DFVDNLYVTLVFLCDCCKTIMLLRRRGNIAKLIDELKEEPFATLNAEETEIQRKFMQQIERN
TITYALIIDVYVVATIIISFFTDYRHGGLKFRAWLPYDYSSPLLFTVTYVHQMVVMVFATNFI
VACDSLFSGLLVNIYCQFELLEYRLKNVEKYSYYSLKLCARHHRRIYEFATAVNEVFTAIISI
QFIVNTFALCFNHYRLSQLEFGAKFGEAAAFMFCVLAQIFYYCWYGNEVKLKSLTIVDVA
LDSTLMSLDNSTKKMFLTITMRAMEPIQFTSIHIVSMNLESFITLVKTSYSAYTMLQQMH
>MmedOR71a
MGKIIIYSFFILQVLDIFFNVQNQDEFTENFTLTSMALNVLLKRHMLSTRRTNILSLIKRLDK
SHFLPVTKEEMKIRSKFENIIECATKMYATGLAFFAFSVPFISIVIDFKSRKLYARMWVPYNY
SSASLYLLTSSYEVVASIYGVSISIACECLYTGLILHVCCQFEILEHRFKTLNGKDTRVVNQC
ASHHNLIYKFADVINNEFKTVMSFQFFNSTAMICLSIYQLTYAKNSTAFMEIMMYLVCVFL
QILFYCLCGNMVKMKSIEFSDNVYSSDWPSWNNSSKMVLLMVIRRSRTPIEFTSMHVVSL
SLESFMSLLKTSYSAYNLMKTTR
>MmedOR67c
MSTILIFQYWYCINHIKTDSLTDLLDGLCITCSNTLLLVKFLIIWSRRRVVSEILAIMAEDWT
NCKSEWNLEAMIDKATLSYRITRIMLVSFLSSSILYTLGVFFGSDNDDGTSNPNERKFVLR
MEFPFEATISPIYEVIVIVQIIAQATFAVMAGMLMTLNATFVLHLVSQVDIICERLTQILNDN
NEEKSRVGIIKKIILKHQRILDLCNNVDYVLTFISLIQFFLNTVVICFLSFILVTSLNTDEAAIII
SKCFPYFVVIHLEALILCYTGEYLTTKSKSISWAAYNSNWYQLSIRECRALLLLILRSQRPM
TLTIGKYINLSLETFANITQAIQCDKCCIYIVLLMLKSENSTVNA
>MmedOR30a
MNQGIYSDLSIVTAKYFLKLAGVWFTMDDAEERQRLVALLWVFYTGLYCVVVNVKNISH
YWGHDMSCCVFGMSNLLIITMAISKVIVLRIRRLELADVVIYAERHFWHYNYDSEEQLIFA
KCRKFCKIWIMFLFCILPASMSGYMTTPIVNNIGRNKSDREFPLEIRSDLPITETPYFELIFTF
QTICVLTLGVAYICPDAILCILNMHVVSQFRMLQYRIMNCWVCENKQKDTLEYVNHCSAA
MKECVRQHQSLILFCEKLENVFTFTIFWHMVIFSLLVGLNCYIILLADTPFARKSIFMFHVIG
SFVHLIIFAYCCNGLMEESLNVCSTIYFGSWNTLPMNRIGRMLRLNVRMIMLRSMKPCYLT
AGGFFPVSLETATSLLSSTFSYFTLMRERFLRNDNQ
>MmedOR67a
MHARVSNGVSPNKYYDTDIKYTFELCQWILKPLGIYPFVYSNVSKLERGVSVLLLITCCSII
QFVIVPFGHYVLFYEKDINARIKFLGPLTFCSTALVKYVYLCLKAPAFKRCIEHVERDWKK
LQDQVYRDVMINYISMGRNLITVCAMFFYTGGLSYHTVMPLLSKVKTENVTIRPLTYPGY

EAFFDVQRSPTYEIVYFMHCIYVMVAGNITMVAYSLATIFTSHACGQMKIQILRLENLTDER
VTEKRGEDRLTIIVKDHVEILRFTKLVESAFREICLIEVIVSTLLICLVEYYCLMEWETSDSV
AILTYAMLLISFIFNILMFCYLGELLLEQGNRVATASYETLWYNLPAKKARDMVLLLAISKT
PPKLTAGKIFDLSLYTFGVVLKSSMVYLNVLQTMIEL
>MmedOR83a
MQKTSVYKKTIDDYEKNVNLSIQWCRWILKPIGVWPTSDVTGTQKCMYRLINVMCYAFL
SFLCAPCSLYVILEVEDVYNQIKFFGPSSFCVMALMKYYLLILHENDIRDCIKRIELDWKDI
SHHDDKKIMMENAKFGRQLIALCTFFMYSGFAFYYIALPISVGKIQAKDENLTFIPLVFPFS
SLIIDTRYSPTNEIVFFLQLIAGAVMHGITSAACGLAAMLAVHACGQMEVLMNWLKHLID
GRSDIGDTVDARIASVVNQHVRILKYLTRTQSTLQLISFVEFLGCTLDICLLGYYVIMESKS
NDITSTVTYIILLTSLTFNIFIFCYIGELVAEQCRKIGEMTYMIEWYRLPGNKKLCCVMIIAMS
NSSIKLTAGNMVELSIETFTNVVKTAFAFLNVLRTMT
>MmedOR49b
MVYRNASYKLDTEYTIRFPKALLTPIGIWPLYQDDTALRKTRRQVQIALIFCSMCFLLIPHA
IYTYHDCEDLKRYMKVIAAQVFSLLGIVKFWTIIINKNEISFCLTELELQYRDVECEEDRKL
IRESAKIGRFFAILYLGLSYGGALPYHLILPLLSEKVVKSDNTTQIPLPYLSNYVFFVIEDSPF
YEMTFAFQMFISIIILSTNCGIYILIAGITMHCSGLFEVINRKIDLFMKETNGKLRDRLRFIIQR
HVQATEYAAMIEKTFNVVFLSEMLGNTVIICFLEYGVLVEWEDHKTLSTMTYFILMTSILS
NVFIISFIGDRLKQVSTRVGRTAYFLPWFELPMDVVKDVSMVILRTSRPSSLSAGKLFDLSL
QGFCDVFKTSAAYLNFLRTMTA
>MmedOR46a
MIANLNQVLCVLELAGTFTCTWPASNSSKFRTVLRNVRWTFVMINVILLTVSLVFGIYYYR
SDIVILTKSISELTALLEVILDLLFCKMNHRRLQGLIGRIRMYLQVADEQENKIIQSYVDRYK
KLFSVIAIAYISTGISFSLAPLFSGQKLPADGWIPFSVEFVGIYWVVYLVQVYCILQTALCIG
VDFMITTLFCFTAARLDILGSKMKRVNRYDLLVSCVKEHQEILGFVDDTKAAVQALLFKT
NITMGSALICGAFPLIYNQSLAVTSQFLCMVVSGCGHLYVISWPADDLKESSLRFATSVNDI
QWIGQPRKMTNVVLIMMQRSRKPCLITMGGLLPPLSLEYYAHFLTSISSYFMAMRTMIES
Gustatory receptor:
>AgifGR1
MSIEPCEIVIDEASQTDNEDNTDSFHFPPRSRRVIDNASSAIQQSPVFINQTTTRKKSHKNYL
NNVHDFHSAMGPIITIGQYFGVFAIDGIKNPTPETMTFKIKSFRTIYSCGVSLNILILLYCIVF
HIIRESINKNKEVSSSLIPFGIFIGLSFLINVICICRSQMWIDLQMKWKKVEETLDMFYLHTP
QLRIKFQIFFSLAEIFKLGAHVMAVVFSAKNANGVIEQIQLRGKFLVDAFYGSNDISFDSWL
GILGIYFLIILVFASFIRMFTDLFIIFLAIGLTERYKAMNEHVSDLIKSNCQPINWSRLKIHYE
MLSDLVRETDRVISPLIFLSISSNIYFICIYLLDGLIPQHDGILNAIYFFGGFVLLVIKTIAVILFA
ARINDESKKPILFLSKCPVQGPSFEPQWLQLQLSIDEVALTAMNCFSITRKFLITVTTTVISYE
IVLLQFQKSMN
>AgifGR2
MRISNSGIMIVIIGNTQLSIGLSFIFQYSIVVTAITARIQSLNSTILAIGGCELTSENMSVFTRYI
TLNRSSVENSLPSTRRSMAIIRNVMSNVNKFYSPLFLCIIPYYCSSIIYSSYFITMLIIKSSAISF
IFLYFIVFNISMLVPMITLTKSVDELKDKFQYLGELVHKLLSKSAPNGILNQELKEFARDML
VNDVEFSGYGMFSLDGSFLLSLLGVIMIILGQL
>AgifGR3
MDGVNNPIPEMMTFKMKSWRTIYSCVVSLNILIVLYCVVFHIIDQKKEIKLCTLLPFAIYMG

LTILINVVCIFRSQMWINLQIKWKKVEESLDMFYSQSPRLYFKFKIIFAVTEFLDVGNHITSV
IFRADNENGVTNYLKSRGKFLTEAFLGSNTLFLDSYWLGILGIYVLIIPIIAAFIKSFTNVIIIF
LAIGLTEMYKAMNEHVSDLIKSNCQPINWSRLKIHYEMLSDLVRETDRVISPLIFLSISSNIY
FICIYLLDGLIPQHDGILNAIYFFGGFVLLVIKTIAVILFAARINDESKKPILFLSKCPVQGPSF
EPQWLQLQLSIDEVALTAMNCFSITRKFLITVTTTVISYEIVLLQFQKSMN
>AgifGR4
MINTGGLVQTTDSLHLALKPIIIFAQFFAVFPVDGIASPDTSQLKFTWKSFKIFYFLVSMSIAL
FLTCSSFIRVVSTEFHSTKMTTLVFSGTSCLTSLLFLRLAKNWPNFSAAWEKVEREIAIRHR
CPKKMSLVKRFKLISGIILSLAFLEHALSLVSGYLSAKECAILRGDSDILGVYFKTQFPQVFN
KTSYSLWKGVVVQSSNVLSTFSWNFMDLFLILLSAALTFHFGQLNDRLNSVKDKTMPEW
WWCEARCDYNRLACLTRRVDADISGIVLLSFGTNLYFICIQLMNSFDRMPNVTRTVYFCFS
FGFLIARTATVSLSAASVHDESLLPAPVLYSVSASSYSTEIIRFLTQVTTDNIGLTGMKFFQIS
RGLVLTVAGTIVTYELVIVQFNSVQQVNPSNLMNACEVK
>AgifGR5
MENILNSFDVNNQNIRSKINNISIIFTIIAVLEHCLYGINHTVNTGFPGYISGLYNFFISTYNID
AEKFSFANFNSAFIMTMFLLFINYLATVIWSFTDLFVILVTTGLTERYVWLNKRVKYLSRRN
YQAIPWNRIRSNYAILSDLVKQTNNNISALIFLSYTANVWFTLIGLFREIATHRTGQYEKIYT
FVSYCYIIGKIIAIALSASKINIESKEALPDIYLCQSSKFTDEAKRLQYQLTYDDIVFTGMNFF
SITRNFMLVTAGTLATYELILVQFHDQANK
>AgifGR6
MLLQISKSPRFDKKLRSKIWPEMMMDEDNHEPDFPLHLISRSMLLDTISRNSDIKTIKKKPT
IDDFEYFHKAIGPIIEIANIFGIFPIYGSRSPSPSMLSFKMRSFKSYYAIAVCVILGIFSFISIVHM
MTVLQSSVLSLQEGISAATAGAVFYSDSFIGAVIFYFLSPKWTKLQQEWRLMEQQLDSYKE
NNSTCPPLRRKFTIITFIVLFFAIIEHIMSLIVNCTQIDGQSSSSNMTFERYIEAYTVKSHGFIIS
SIDYSLGLGIVCFIASKFATFIWNFTDLFLILVSTGIAERYKYLNKNVLSSIENKKFDFNWLD
LREKYAMLSSLVKEVDSVLAPVIVLSFFNNLYFICLQLLNGLSSNEKTAFDILYLFGSFIFLV
GRTVAVTLSAARIYDQSRMVLPAIFNCPASSYNIETERLQHQLSTDEIALTGMKFFSITRNF
MLAVAGAIVTYEVVLLQFNASMKS
>AgifGR7
HANVCEYTNFDSSNDSVNYLIDEKFNDNYQLINKIRFAHMELVKVAKITDSVYGLSMTITL
MITSATCTGILYIIWVTVCIKNQGTMMEKLEQIEILLGWLFFSILKFVIISFACGRTSNSASNI
GNIICQLHEPSASKKFCSELTNFVFQLMDTKLVFKPCGLIELNNSTLCYIVSSILSLLIVLIQIR
DIPSSSEELL
>AgifGR8
MRISNSGIMIVIIGNTQLSIGLSFIFQYSIIVTAITARIRNLNSTILAIGGNVLSSENISVFTRHSK
LNLNRSDVIILIASIRRSMAIIYNITTNVSKFYSISILCFLPYYCTGIIHSFYFVVILFINSNSTP
MFIFQLIWYNVAMITPMVILTKSTDKLNDKFQYLGELIHKLLSRSAPNGILNRELKQFARD
MLVNDIEFSGYGMFPLDGSFLLSLLGVTVTYLTILTQITK
>AgifGR9
GIILGLYQRFTYLNQFILTKDIMEFKENEINQPWNSIKLQEVWLMHWHLTKATKIINRIWSN
QMFFWITITFLFMLHRTYITLNKIIYLVVMREALLITGAFINIFTLFVFCHLTSEQANKMAQN
VFSPNTIVQKRMNATENDILAVGYFRRKKIRFTAAHGLIHVHLPLFISMIGAMTTYLVILYK
SPS
>AgifGR10

HVFRMLMVVQPCYATTTNSKKTAILVSQLLSTTWDSEICKQLEYFSLQLIHRPLDFSACGL
FSLDRGLVTSMAGAVTTYLVILIQFQKADDTKDTSNMLKNATLLLRNVSSLHNSTALKTF
>AgifGR11
YYIILSFLFPDSTKSKAKLFVLFYWIIIETVPIIILTNNVTKIMNEMKATSGIVYIILAQSTVFD
KIQNELNAFGLELLHKKISFSAYGIFSLDNQLLQSISGTVVTYLVILIQFQM
>AgifGR12
HYILNNIVMYWCGYNTYQGWIWFDLGLVIGPNALVIFITSLLVLKKRFKKINRLIIDLPGVN
STVLSRYYEPISHLKAIGQVHSDLKELLSKISNLFSLPLIILLSTNFMEFAACGYMAYNKFK
DDEQQKWQFNNIVTVLALMSWVILHFCQMFFLIDSASSTVKEANETGNILHQVMLQHQFI
NTREVEMISLRLLQDPVKVSLYGFIELDFSFLYNIIKSITSFIIIMLQLDE
>AgifGR13
MDGIPKSSSTLNSDVQNLIRIKSVKNARISPQNPEVIVQPRKLLSTPCQINKVHPLITEFNEP
ECFHRSLIPVLIFSQLFGIFPISKVSNKNPKMLNFNVKSFITFYSTIVLLAIFYLAFQSTIILIQT
VNKTGGCTLDTEGISNAVFITAYYCSVFLQCLFFFTLPKKWIVLQIKWDNMEKKLNKSSQD
PPKLSRKFNIISLIIIIFCIVEYTWWNIDIQGSENSDESFDVIWITINNSNNTINNFQIYILMISGI
YPAYIWNFTDIFIILVSLGLAERYKHLNKFALVSSTCDNHEHYWNQLRINYSILSNLVKETD
NVLSPLIFISVGHNFFYICLQLFIGISFDDLNILDISKDYTIFAFVFIVMRTFGVLYSIARINDNS
KIILPIIYQCPLSKYTNETYRLQCQLMSDEVALTGMNFFSITRISILKIVGAFVTYEIILLQFHS
SGK
>AgifGR14
MIYLLIYLWYFFQVNSTFFISIFLWIIDNFATVIWNFTDLFIILITTGLTDRYVWLNKRLKYLS
RRKYQTIPWKQLRKNYAILSDLVKQTNNDLSALIFLSCATNLYFIWLGLFKEIALDKTGLFE
KIQFFVAYCYLIGRMIAVTFSLSRINILSKEALPHIYLCQSSKFTDEANRLQNQLTYDNIGFT
GMNFFSITRNFMLVTAGTIVTYELILVQFHDQANK
>AgifGR15
LAYKVENNYPLLETITYILFYGTITSQTLLFYGFMMTLYRRFNHLSELVVKKEWMMEMKI
QTLSGTWDGLKLQNVWLMHSNLSTAAEKINNCYSVQLLLWIFSSSMSVLTRLYTMMNFK
ENFEYITPRDTFCALGVFFNLILIIFSCHITSRRANRIADKVFSPNSTIHRQSNTIEGNKTASIY
FCIRKIRFSAASGFIHIHLPLLLSIVGAMTTYLVILSKSS
>NvitGR2
RQSSNEDEPECFHRAIGNILLMSQFFGILPIRYIRSSSVRNFSFYKFAPRVIYSYFVLLAISVM
TSISFLHLFRTLNANSFQTKGGIADATVGAMFYGNSLLGNLMFLRLCPKWISIQHDWRAM
ERLIDNNGKWKGPVLRWRFTLISSTILSLALLEHILSMVNNTPSDVWFGKKNLEDFLIIYTN
KSHRFIVRNVDYNFTLGLFIFFISKVSTFTWNFTDLFIMLVSTGLAERYKRLNARILEATPAQ
LSVTDWHELRECYAVLSALVKKVDNEISGIILLSFTNNIYFICLQLLNGLSPSTAEHPIINSIY
FFGSFIFLIGRTTAVTLLTARINDQCKLILPILYNCPSVNYCREAQRLQQQIATDDVALTGHRF
FSITRNFMLAVAGAIVTYEVVLLQFNIALQRDEELNNMAAGSNGEKRKEGLYNKTRRA
>NvitGR18
QTFPAMRRSKCDDLFLKCLFYVLKFLGVAPMAIDNSPAPEKDSPRYVRFVASKLGVFYNGI
IACLTVVYPSYMTVRYLTSSEYTKNIELEKVIDEAQSTFAMITSTFIIVNICVRQKRAAILANQ
LSSIHSVIMDLAIDVGDDVRRNTIISYIKKIVFVNLVTTIVWVASTPPEEYQYLSYFIVMSFY
NIIIHAMLLQYSLVLKLLQQLYRSVNADLSSLLKKSSSISDDNNCHLMLKRLKHLRQIHATL
CQISQDVSNFYSLPMLFCVTHVFLTQIIYCYYVVMTLIVWNINEKPILVILNCVTLVTLLAVS
MTILVRDAGVTAVESKSTGEIVSGSIDDCQDHEIERQLNVFSNYLLHKDVHFSVFNLFPLNE

SLLISIVGSITTYLVILLEFEVDSTKK
>NvitGR51
MGDIFEDMEKPFLPMLISNWIFGIGIIEYPIRRQLKLLSIVYSTLILVVYAYLVYICHAHIYLTA
VAQIKPIIEMLHYYTNILITISIIIFGWFQTQGLQKCMLKAAQANLLMQQIGIFKNHSNILKN
ELRKFMAFFLFILSIIIINSTVTFYNFVPNYQQIIFIVILQNVPLLYGYIADSSFLNIIGYAYFKF
DSLNKLLKSISITKADNPMHKIIAKQPFYEKVYPQVYSSIDYKDYTFLIKKIKLAHLRLVKL
CREANNLYSFHILLSIAIAFVMIINKIFNIYVVLNDDDIDEGSKFRTIVRSVNWLIYYIVRNLT
SCCLCTTVLNTATKTGDLICELYDEPYITENTRAEIRYFNIELVQNKLEFSAYGVVNIDLTLL
QVMASTIATYIIIIVQFQKLHFVPNALVGNQTNTYRI
>NvitGR49
MGDIFEDMEKPFLPMLISNWIFGIGIIEYPIRRQLKLLSIVYSTLILVVYAYLVYICHAHIYLTA
VAQIKPIIEMLHYYTNILITISIIIFGWFQTQGLQKCMLKAAQANLLMQQIGIFKNHSNILKN
ELRKFMAFFLFILSIIIINSTVTFYNFVPNYQQIIFIVILQNVPLLYGYIADSSFLNIIGYAYFKF
DSLNKLLKSISITKADNPMHKIIAKQPFYEKVYPQVYSSIDYKDYTFLIKKIKLAHLRLVKL
CREANNLYSFHILLSIAIAFVMIINKIFNIYVVLNDDDIDEGSKFRTIVRSVNWLIYYIVRNLT
SCCLCTTVLNTATKTGDLICELYDEPYITENTRAEIRYFNIELVQNKLEFSAYGVVNIDLTLL
QVMASTIATYIIIIVQFQKLHFVPNALVGNQTNTYRI
>NvitGR43
MILRKSTEDIIFFTTCLFYYFKVLGIAPISLYIKSTKKSASQCVVFTRSNRALVYDVVLILNLV
TANIYKILYLCLRVSSTKIITIEAVTNCLEDFVTCLSAVFILIIICFSREKLSAMVNAISGLTECL
DGFGVENPKKHKLQLEIGMIILVNITTWILVFVTTAVAEFSYLLYDTIMYSNVIVVNALLIQY
GVVLKLLRHNFKMLNENLLVISQEVPIKIQSPVESNRRVERLSQLRKLHASMCKVSRDVS
NYYSYPALACVVCVFYTLIYTCYYLTRPIVLYDQNLRGDMFVMSLVYGLLLVFSVVILTKS
VTATIDESDRTKEIINAGLLRFEDDEKMSKKLNQFSSYLLHTDVKFKVSKLFSLDDSLLTSM
ASSIATYLVIVLQFLQK
>NvitGR42
MFLKCLFYAFKLFGIAPMAIKALTSKKNKACHFLFVSSRLGILHNCILLCISISTIYFIIDDTLS
RSLFTDKSNLELVLDTACGICVALTSVVILLKMSINREKAIIIINKLNIIYQRKVESDKKNPSIL
LVGSVKIIIFSIFIPTIFAAIALGLEQSSILISCLPFTTYQMTIIQYTLILKLLHYLYQSTNTELQS
VLTSKVPISIVQNRLLGMDTQRVSTKIELLREIHVVLSHLSNEVSGFYALPMFFCISNKFLVL
IQYCYYIATVLSHKQDTTGQYEIILHCMSFSTVEALSIVYLTRAAGLVVTESKRTGEIVSQLI
VDCPNKLVLKQLNGFFSYLLQVQVDFSVFNLYQINESLLTSITSYITTYMVILLQFKETSCR
SGSTEHTENMSTPPSVD
>NvitGR41
MFLKCLFYAFQFLGTAPITIKALGTKKNKACHFLFVSSRLGILHNCILLCISLPTIYLMIEDIL
ARSFLRKKTNLELVIDGVCAIYVVVPAFAFLLKISISAEKAIMIINKLNVIYQKRKIEFKKESP
PVLLLRPVKIIVFTNVVPLIIGCFVSGQHHISNLLPLPSFLTYQMTFMQYTLILKLLHYLYQS
TNTELQSVLRSKVPIFIVHNRFLGIDSQRISNKIELLREIHVLLCHLSKEVSDFYALPMFFCIS
NKFLILVQYFYYAAAILSDKQETATQYEILILTIWFTIVEALSLVVLARVAGLVVKESRRTGEI
VGGLIAECPNKLILKQLNGFFCHLLLVQVDFNVYNLYQINEPLLTSFTSYITTYIVILLQFKG
ISCPSDSTDHTENVSTPPSVN
>NvitGR29
YFSIDLLYAKCLYYYFKCVGLATMSVSFKSTVENKKVPYSLFSPSKIGFLPNLVIVLIVIGTH
FFSLKMAFEVDEIETSVKFDRTVESVRLTFGVGVSVFILVFFCAKQEAAIDIANNIKKASVL

SANFSTKTVSQKELFSVYRATGWIFSAHMVIWFLIYCSTPWSFGLMIYYVSLNIYELVITST
LVQYSILLKIVRQIFRNVNANILDIFGDSCAIDFHTVGTIGNNRSEVRFRRKMRKFSQLKDL
HISVCDVAASLGQFYSIPALFCIKYEFISFTFYFYFVTKLFTGMYHETITIHTIFFYVFGILHFI
VPLIDLVGSTSAVVNEGKTSVELISKWIEVVKDQEQSTVRMSHFPNYFAQKKLKFTAAGLF
PLDGSLILSIAGSITTYLMILLQFEGIKPYSS
>NvitGR15
MVKGTKYLFDIFKVFGLATMSMTDCTKKNNFKNRKMFSYSYHGIIYNGVLICFLIIAGIYK
MYYIRDKLIDQSRMSEVIDVFGNFIIYAVSVVLLSKYMISQTLAVRIGNNLYSINLVLKRFNL
KYKNQYMIMHYKLVLLFDITIWLGVIIIGSFSDCTFIAAILTYIPNFIINCLVIQYVVIIIFIYGE
AKALNNQLRKYVDRAFSNTLLYQFRRPVLSVHYYLPENNEIILLQKSCLSIYEVSNNVSKF
YSLSILICIVKLFFSIILNTYFFLKPSIFGKSMITSTMNHVWSISWLTLDTFSLCILTQYITMTV
NEIKKTGDIVHQILRHSTSLGVIKQLNNFSLHLLHKNIQFTAMDMFSLDCTLLHSIVGSITT
YLVILIQFQENSSEKHKP
>NvitGR11
MKTTIINCILIKCVFYFMKLIGVCPFVLDKKEILKSSTSGKMYNLLLIVSYIYSYVIVIKCRR
NLHYSEETQLGIIIDMIGITLKYSAVIVCWYTLAVHQTQVKSIIQHLKVIANNQTMLASKCR
REKINNEFKTFRYGLIVINILGLTILTQNNFINNYYKNCTTDFTFTLFDIFQIVIYNVIFIFLRIV
LYTQENYRIINKALNKCTNYNELDNVNVTYDTILSLKKLQSAGLAHKNISDLLENIVDFFR
LPVLLIITAVFVQILIDVHLILYFIKTENWNHIKYYSLIHLLITFAIRVSATYFICSISDSTGIEGN
NTKNIINVILNKWRFTKSHKNLAKMFIFNLHEHKIQISLYGLFNVDYSLLKNLCSSTIMYVI
FMFQLDGIIK
>NvitGR10
MVNLSDIKPLFYAARFFGCAPHRVTDSDVLLTTSGLIYSGIWALGFVCCCCYGLRLICAGV
YTGERNMLALTAVRTLLAYVCFLADDALTMRWNERLRSALLQLRNFDVAVSYGRKRSVN
WKLRCCCWMLVGTIIAYWIGVGYVTYKCEMTNPLFNAITYVIANAAISMQLIKFAGLLILL
RQRFRYLRELLPLEAAHPNSARRAIQLQDIWWLHCSLANAAETINSCYSLQLLLWIFTMW
LNALSRIYAMNETLVDSGQFLLMLRESLLVTACIGNLMLIALACHYTAREANSVGRAAFAP
QTSFSRKRSLLEHSLEVGVYFSLRQLHFSAAGGFIQVDLPLLLSIAGAMTTYLVVLHNNS
>NvitGR7
KNSSSGSSPSSSIQDIRPTFLIARVFGLAPYAITNSSINVSKRGILYSVPWLGFYLYALYNRLN
LYTHSDLETKFRILSVTRTALAVIALLVDLVVCTFRDDRFQNALDCVRKYDLAVKYDVETN
ARLMRIHSWTIYSFMITYYLAIGWFTYVDEPYEGVMAAVIYVYLYLPLSIAVMKFVALITSI
LLRFRHLHRMLLPGFLSIMMELDSEPKRLHLRDVCWLHSCLCAAAANVNSLYSLQLMLW
FANLTFNTISRINDFGQPQNSIDAFKLARDAGLVLIFVTLVFFIAGVCHVTSTQANKVGAVV
FSPGSRYFRSRVVDHQDKEDKFYIGQYFALHPLHFSAASGFFQINLSLLLKIAGAMTTYLVI
LKSPSNC
>NvitGR45
KNMLFGKQIKKFDYLKLLYYYFKVFGLATMTFVTDSTKTTPNRFGTFSRSKYTIVYNVVII
LVFVMPCLYNMTIFCVGTNRVKFEDFADCIQINMALFVTIFILSKFCISSDSLISIANSISRITE
SLLTLSSISLQKRIKVSFEIKQAFIVNITMWIAFIVINLSEIEPWMKNAVNMYVSNFLVSVLIL
QYSVILKFLQYDFKILNENLIEFRNEDSMKIRSPTETKAKIDGLLKLQKLHESLSDTSRRVS
MFYSYLMLVSVLNIFIMLIFVCYYLAKPIILTHDSNFSSIMLLRCFWYGLLFVVLLVTLTKF
VTATIEESRRTKEIISSCLMIPDADEKLLNKLNQFSLYLLHRDVKFTVWGLFTLDESLLTSM
AGSITTYMVIVLQFQQKD

>NvitGR58
MGKKPMTRPILPLLISNWVLGIGIIEYPIGTPRPTFSFIYSTTLLVIYCTTSIMIRHEIFRVSIILK
NNTVPMTIVFYTNIFLTISIVTLGWYRSKGLRRYVAKAAVADDLMERIGIPNNHGKMLRAV
AGQVIKGFFLVTVLIAIIAVIVLVEDAPLQTKILISSVMSFPLFTMFVSDAMFTSCVRCACYR
FTELNKVLKAVLTSTHAFPQHKRVCSSVFESGGQDSNFVINNVSQRKNPAVIVKLAKEIHL
QLISACQEINNTYGLHLLLSIIFAFAVITGNMYLCYMSSRNSNIPHYILVKTLVVSGIWIVHY
GMKICYFSIVCGCCTENSIKTGDYINEFYDEPSTTNETKLKIRQFNMQLIQKPCKFTAWGFV
DLNCHLIQVMIGTITTYLMILIQLGTTTYVADDSYSKYLKSFSSLTY
>NvitGR47
MGKKPMTRPILPLLISNWVLGIGIIEYPIGTPRPTFSFIYSTTLLVIYCTTSIMIRHEIFRVSIILK
NNTVPMTIVFYTNIFLTISIVTLGWYRSKGLRRYVAKAAVADDLMERIGIPNNHGKMLRAV
AGQVIKGFFLVTVLIAIIAVIVLVEDAPLQTKILISSVMSFPLFTMFVSDAMFTSCVRCACYR
FTELNKVLKAVLTSTHAFPQHKRVCSSVFESGGQDSNFVINNVSQRKNPAVIVKLAKEIHL
QLISACQEINNTYGLHLLLSIIFAFAVITGNMYLCYMSSRNSNIPHYILVKTLVVSGIWIVHY
GMKICYFSIVCGCCTENSIKTGDYINEFYDEPSTTNETKLKIRQFNMQLIQKPCKFTAWGFV
DLNCHLIQVMIGTITTYLMILIQLGTTTYVADDSYSKYLKSFSSLTY
>NvitGR53
MSDIINKLEKVVLSIAYYTNWFCGIGIIEYPIGKQHRVMSFLYTGVVLIVYSVLSVYVYSDF
AIVSRDYEVNQTMMKGVYCATFILTFSTIVMGWYRNKEIRSILQRMNIAMRIIDKLGASKN
YTKAFTVQVGYAVGTLTFLIIIVIINALVVYKRDPKHDSHVLTVMAVNYPLIILQVVDTLFIN
IIQYARKNMRVINDVLREMLTSTQDFPQHTKIVRRYLRTLDSPELDTDIVDQKTDAEDKMY
TINMSKKAHLTLVKICQETDDTFGLHILLSVIVAIITITVSIYHIYMLVDYLSISRAIYDNTLLP
VCILLIYYYVKIHAISHFCSSTSEEVAAVITGDIISELYDDSSIGIESQTEIRQFGDQIVQNSLTF
KAHGFVTLDFTLIQNVVGFVTTYLMILIQFGSSTSIEISR
>NvitGR30
GNMLIKNLSLNETVLEKCILYFFKLSGIATLNFDFKLSTNRSKKFSSTFTRSKTGIAYNAALI
SLITIVTNYLIEFQINHNMYKNFYDKLDIGYAALISVTAVLILVKFSFQQEKTLTIANELNEIR
DSLSLNDCSVDGKGHALRRFIVLVFLAHFLFLTILFSTSYVLNNTTINIVRTTLNYIAIYLSNF
IMHSMMLQYSIILKLIEHLSRGINDDLVEFSRPPQGLNSLTFTKKTTSQRVGQLANLRKNFS
SLCKVSQDVSEFYSWPMLLCLSCNFIAFVRAAFYIAMPIVHGTDAFTANIYVRCCCYISHN
AFSLIILTKSVTASMTENRKTREIVNDCIENCDDQEILKKLEKFSCYLMHKKITFSVFNLFSL
DESLLMSVIGSITTYLVIILQFQNNNAE
>NvitGR28
PKKFYQNARISDLWFAKCVYYYFKTVGLATVSLRLKSVKKNKKNSSSLCTSSKLGILINVV
LSLIVIAIFSYTTIVIAEGTFKNSLKFDRAIGVIRIILGSSAALIILITFSCKQGSITEIANNMQVL
TIFSVLSANFKTKIGNTNESFSIFRETGGVFFVNIIAWLLLFVTVPTTNWKVFAVTPYVPEVI
MTSMLVQYNMVLNLVKRLMEVVNANLLYTSQYDDKYEDNQITMIKNDRNENSFKRKIIK
FTQLRDSHYMLCDISEDLEKFYSRLVLLCITYIFGSLILCSYFNTKEVLKQGVEFLTLRATLF
FGVTVIHYIMPLVNLTRSTSAVIAESKRTVKIVNRWSGNFHNQPEIAMFNQFPNYLDQPNL
EFTAGELLALDGSLLISIAASITTYLMILLQVQDTSPN
>NvitGR21
MICKPHYTSDFFFLKCLFYWFKIFGTSPMGIDFTSAVKSNDVPQDVHFVFSKLGILHNAILV
CLAIIPSCVTIKEAYHTEYTDRLQLERVIDTVHAVATIMTFYFILINVCINQKRAVAIANQLNS
IYSQSTSLFSKSKIRPNRAFRSVKKIVSIHMVLMLLLCLFAFTFVHQNLIYHLFINFTNVTIY

VMALQYSLVLKLLQHLYRSLNADLRSFLITRDRRDPSSISLPIDELQCRLMQVYEIHASVSR
VSQDVCDFYSLPLFFLFAIAFFTLVLFFYYFMLVLLLMNKMIDYGFVLPFFLTLLGLTILAIS
TLARIAGGTVKESNITREIVSESIMNQRNQPISGQLRDFLHYLQQKNAKFTVFDLFPINESL
LMSIASSISTYLVILLQFKESNSTQQTAPSSAT
>NvitGR14
MKKTLYVCVYYFVKILGLCPFTRKKGRFVKVSHVGMSYNVLITILYSRAFVKAIQNRHSIR
LSQETPLAVIIDTFTHVLSYSTIVSSWLVCAFRQKIFIKVFQSFKNVENLENDLLPSMHCSKN
GLEENLKEFRARFIVVNLICIIFTGSTVVIISMCEDMKNQSWFWFIYNIPINVTFNVAFILTEF
MRCLRKHYKIINREASKLARSRKRSFARMPMAFSKKLQTIGRIHSDLTELGETVVKLFSLP
VLMTVHGHSANIILAIHGLYRILKSDQSMGGPCVLYAPTVKFMIYSIIIFFICSIPVSTCNEAD
KTLRIIGQIPYEWHEEEMHNKMIKNLMFQLYQKRLQVSIFGFFNLDYSLFRNVWIVIIMYLI
FVLQLDPSSFIFAILK
>NvitGR6
QKKSPGNEESPLYEVVCPAVYLARVFGLAPYELEDARPDNNRPKRLGASTVYCFFSIFWLI
VYTYIVVISLIRFGGLDRDKPVLGVTEDGKLILNYLVSMVDTLLCIRCRERFVHVWNSIQD
FDESFQLGNVPAGRDSPRYSPILRRARFWVWTILTTNVVGWTMINQLGMHAFGEPYLQNI
GYMLTYVGTCVAVLKFVGVVMLLGQRFAYLNEELARQRKKEEGRRSRAADEIVKKIESS
YNKLLSTSEELGNIYSFSLFLYLLNLFCHAVSNMYFFTIWTILDPGYLKNPKIVFCLFSWLLI
YLVQMLLIHVACHFTSLEANRMASVLLDWRRQAYRQSKYEFSSTLHYLNRRLNFTAAGC
FNVNLPLLTSIFGHLTTYLVILLQIPDSSNS
>NvitGR4
MYKDLYNTLEPIMWVWRFVGAYPFVIRGPIGSQQYVLSTYSIMLSLIFLIVTLQYCYKSVD
FINQSATEYSLFLITISVQQVSNVICFFDSVVLKLFFGKKITRSIENIAIQDKKLSKIGCSLNYS
QAVRKGRLYIFILFIFLCFHIHSELVYFNGGAPLLLRIFGNYLAMLQVANVILFAWLMFNVG
LRFQVINCKIKTCIFDIEWADQNSSCLLVLRTTAKAHAQLCQAAKIINGTFVLSIINCVLSAFI
FTTTMLYYIFMELKNTLPFSHAVYYCSVILIHATTIVIIVQSCNWVHRNAHATVKVLHEFSK
ENNRFDDDKHLNQIIHNFSMQILHHNLTFSAWGLFPIDSTLLQSLAEAVTTYLVILIQFDPLV
T
>NvitGR46
MLFLKKSNNKNLDYLKLLFYFFKVFGLASMTIDATTAKNTRNHFWTFTRSKSTVIYNVIFI
LVFVISNIYSMTFFCRGTYVVNFETIGDCGQTTLSLFVALFILTKSCISRNTLIIIANSISRITDS
LLSLSSTSIQENSKISSEIKKMFIINISTWTVLFGTFAFDLKPLTKYGIVVFFSNCIINHLVIQYS
VILKLIKHNYKILNENLIEFGDQESMAIRSPSEAKIKVDRLLKLQKLHESLSDTSREVSNYY
SYPMLVCVLHVFIILIFVCYYFFKPMILHSKNLSTFTFLRTIGYGFAYGLLLVTLTKCVAATID
EQNRRTKEIIGSCLMISADEKVLNKLNKFSTYLLHRDIKFTVLGLFSLDESLLTTMVGSITT
YMVIVLQFQQNLKR
>NvitGR23
STHCFLFNFDEMFLKSLFYFYKIFGVAPMTLDSTKNRPSDVSFAYSKFGILYNLFLVILTIFAF
YQCAILVHFDVSVGMDFQRFVNTTHLIFFVFTTLIVLIIFCVRQERAVALANRLSSIYYLNK
NIKLSAILPSIKGIVSMTFITTIMWLVTTPYDDAHLLTYYVAISLYNFVINSVFLQYSVLLKLL
YHLYRSLNTDLRSLLESLDIAIEMDRHSNNEIKLTSGRLKRLREIHMLLCHLSGDVADFYSL
PIFFCITNAFFVLIIYSYYVFRGFAIAIMPVLVTVHCTTMMVVIVVSLTILLRTASVTAAESRV
SGEIVSESMASCSNQFIGRQLEVLSIYFLHKNVKFCVFNLYSLDESLLMSIVGVITTYLMILL
QLDGSSNCK

>NvitGR52
STHCFLFNFDEMFLKSLFYFYKIFGVAPMTLDSTKNRPSDVSFAYSKFGILYNLFLVILTIFAF
YQCAILVHFDVSVGMDFQRFVNTTHLIFFVFTTLIVLIIFCVRQERAVALANRLSSIYYLNK
NIKLSAILPSIKGIVSMTFITTIMWLVTTPYDDAHLLTYYVAISLYNFVINSVFLQYSVLLKLL
YHLYRSLNTDLRSLLESLDIAIEMDRHSNNEIKLTSGRLKRLREIHMLLCHLSGDVADFYSL
PIFFCITNAFFVLIIYSYYVFRGFAIAIMPVLVTVHCTTMMVVIVVSLTILLRTASVTAAESRV
SGEIVSESMASCSNQFIGRQLEVLSIYFLHKNVKFCVFNLYSLDESLLMSIVGVITTYLMILL
QLDGSSNCK
>NvitGR50
RNNKINPRKGRPILNIADSFRPFVWINGLMGFGMIEMPYGRPWPKLSVFYGLLRAIAFSILS
WYVFKNIQPNTRISALMFLIYKIIIAASVGAVVISSIMGLINHEKSKKLYKKIKLVDETLKMF
GVEPEYASDLRRNRNIIINYYRAIIVLFIIKLGSSYIFSREAFCTRNILNVLYFNIPSIINPLVDIN
YTSKIHVLEKRFERLNALIHNVTTSPSKMMHTHDFKKYEMILNNRGVISVIPKYCFKNRN
NIEHLLKVTRQLHLDLCNTARNMNNLIYTQMSAQLSAIFVHLTAGTYCFYFIFNEKTIIPLQ
AKIHSYVLLIFSIICGIVRIILITYATAGKISKILHEIQIQNTEKKLTNEIHQFCMQLKQHPLSFT
VCGFVELNFSYVTGFVGAVTTYLMILIQNQTDMIEAAKTMVDPIKSNSTSVTPA
>NvitGR48
RRTKPDADALPSVENVTRTLTPVLWLSRFSGLTVFEMPAGTPWPKFSAAYALFLCSAYGTM
IWFGETFIVKETTSVPLVIFIYALVKYVNAFLAAVSFAVGLLHYKKTMKFTKRLKHVDETL
KVFGIEPEYAASRKENIRIVLIWIVATVFQIIGDAAICFVIYDPAYIAILKFFIFHIPFQSMSLLE
LTFAMKISTVRSRFEKLNALFQNVLENPVLPMHFKHVNKYHNILRRQHSEEGDRNRKNLE
LLLMTSRQLHLELCAITREINEVYGKQLAMSIAAKFIYVTGYGYVFYLYYNEPSISLSIKIL
NCTYIAYNLTFITVMMIYFIGQSVAAQKTTQIAHEMPVTQSQTKIIDEIHQFSLQVTQHPLEI
TAASLFTLNFAFMRGFIGSMTTYLIILIQYQPNIAAAAKSMIDEIMANMQNVSAFYHFANFS
T
>NvitGR40
MFIKDFINKKDKVFVKFIFYYFKVLGTATISFNTESTKSRKRNEWKFTHSKSSIMYNIGLIIF
VTTVSSFGFIYASFQHQTNFKKFERVTDRAEDVFNILSVTIIMMMFCFKYKNMAGIANKMS
MIYQSLISSCPQTLYKSFTDNILLHIILIISPYIIIWPFIIVFNFINFPEFEIYNFTIFMNDIVITALL
MHYSTVLILLKYFFKMFNVRLSFMLEEQDYLCEIQYLNCNNRSKGKIKELFHMRKLYASL
YEVSQDVSSFYSGPMFLCIFKILVSATLSLYYVAKPIIIDSHTILNVEIIRNSMFGLIYATALLIF
TTLVTQTARESVKTREITNRCIINFENKYIIKELSQFSTFLLQADVTFTVYGFFSLNQSLLTS
MTASMTTYLVIILQFQQNN
>NvitGR39
MDILKITLNFFKMCGLATMRFDAGTTQNTSVQSSWCTSSKKGQVYNLFLICLITASNGYVA
TIVYEYNISTQEFDKSFDVAQYIYTSATTVAILMLYCFCQGRAVSIVNNLKIMHKLVTNVNS
RLSKEEEPTMGGLKRIAIMTTVIWFVVVFTSCNLSFGVVMYYLTLYPCILIINCTFLQYTMI
LHLLKQLFAILNANFRYVSRQSIVPKIAVAATNSMLQISTKKTQPFSDLCELYTSLCDLSMDI
SKFYHLAMLFCVSHVFITLTTWLYYITKPLVTGAIELSIIDYTHSLLILMHNFFMLFILTKSV
DAVVDEKTGKITNRWLANLQDQHLVNELNLFSNYLLHKNVSFTVYGLFSLNETLLMSITG
SITTYLVIILQFQSGVQK
>NvitGR38
MLSIKHMFYFFKLSGVATMKFNTNLIETGRVQGSWFSGSRKGIAHNIVLICLIFVFNCFGVR
TVYYWSHIQFERVIDVVLYAYTTVIAILILIVYCFRQKQAIAIANKLQILREFTMSINGQLNQ

GEQLVVSSLKRISVAHLVIWFALIMSTSTEMYMLVYSIATYPCILIINCTIVQYSVVLKYLRQ
LYVILNANFFNCSKQFSANTSSVRTNSLSDLRELYMSLCNLSADVSEFYHLLMLLCLSYLF
VTLLLWCYYIVAPIFVKATVLPILYGYVRSLIIVIHHVLMLVILTQSVSALTRENKKTGEIINK
GISNLGNQRVLNELNMFSNYLLHKDMKFTVYGLFELNESILMTFAGSITTYLVIILQFHSRD
>NvitGR37
HQSFQYKLSEKNMITLKRIFYFFKICGLATMKFDSNMAVKERLQGSWFVNSCKGKVYNAI
LIILLSIATYYVTAFVYDLSISSGEVGKLFDIVSYVFTTITTIVILAVFCLCQKDAVLIANNLRR
THMLIANINSQLSNEKINILRTAKIICIVNLVMLILVFITTLRQEFGIIMYYTTVYPCLFVINFT
FLQYSLILQSLKQQFTILNRNFHYVLRQCIMQKNLGATGSSFQTHKVRAQSLSKLCELYAS
LCDLSTDLSKFYYPTMLFCVLYTFMMSTTWIYYTVEPVIVGKTKLTTFKYIHSLIFLIHHIS
MLIILSKSVNAVVLENKRTGEITNRGLANMQNQQTINELNFFSNYLLHKNVSFTVYGLFSL
DESLLMSITGSITTYIVILLQFQSSVQ
>NvitGR35
LFMILKNWKISDLLFLKCVFYLFKLFGLATTSIQTKPSTSRFHLPLFTRSKLGQIYNVILAIGI
SFVYACLIRWTIKHYEHHIISRSHQAIDYTHTTMAMITAIFVLLVFCIQQEKFLNLGNRTVRL
GELLVGFYEVQRPSKQKTLTKHVKEIYIMVGMTWLSIFVTTETGGYFKNVVYFSMIYLCN
QIITLTLLQYSAILRCLQQILWFVNENFLQFSKEPCQMDKIKIQFSKLRELYLSVCEIAEDIER
FYAKPMLLCIVYVFVTLIFFASFITKPMVSAVVISDFQICHCSFRILHYVVALIILVKSVTAAV
SESKRTGKIVNKWLGDCNTLQVDPKIYHFSNYLLHHNLHFSVLELFSLDGSLLMSITASITT
YLVIFLQLQE
>NvitGR34
LKMPFNNPLTSGAAVLKCVYYVCKACGLAPIAIRSNDGRKLRFPPFEHSKAGLLYNAVLIL
IILSMSAAIIQCTFVYRTKAEILKFDGVIDVTHNTMASVTAIFVLTVFCIQQRKILELANRMR
VLGEMSQSLCDVEICGGRKRLLRDVMMICLTTCCTWLSIFFTTQVESYKGLLYFSYIYLCN
LIITLTLMQYSIVLRLMQQILRVVNANFHHFSTEPSQRKLKVQIIETSCQGIGRFTRLRELYL
SLSEVAEGFEEFYSQPMLLCIAYIFLTLIFYAHLITKPMVVGTRSVTNPQLCHCVFRIMHYVI
SLITLAKSVSTVITESKKTSKIFNKWLGTLDSLQLDPKLYLFSNYLLHHSLQFSVFQLFSLD
GSLLMSITASITTYLVIFLQFQNHQTTDS
>NvitGR33
MFGKFDETILEKLLFYFFKMFGMATMKYEISTIKNEKIKKRRLFTYSKIDIVYNSFLILVTTI
LNIILVGLKIDDKTSLPNVRGVQKITDVVQFGFATLTCVFILVYFCARQKKAIEMADQICQI
YGSMIVNNCDTGEKRSILLITLFVFSINFIFWLVMIITSVNVTMATPNNLIFYYLAVYSCHVI
MQTLLMQYSIILNMIGYFFLHINKSLVILLKKPNQLFLDAQCRNIDKTRGERLLKMRKTYL
ILCKISEDISDFYSPLMFFCLSVTFVTLIRSGLYIAVSIADKESNLTIKGMIHCIGYVVHYYFLL
VMLTKKASKIVTESKRTGEIVSDCVHYVDNQEIILNQFSNYLLHKKIKFTVFNLFVLDESLL
LLFAGSMATYLVIMMDF
>NvitGR32
MWFSNFHDTREAFFVMFVYLFFKAFGLATVKFNFESIKKTLIAGKEASEVLKPSRVGIAYN
MLLIIILSILNYVAIRVSYERPDFTDRSELEMKIDTVKAVTACFSSFIILLIFSFQQEKYVLTVH
EVLSIRQSLISINSAIYFENESIWKIITKLLIFMFVNWILLFITIEIQNDYQFLLYFVTTNLCDMI
MTHTVLQFSIALKMIEQLFRVTNANFDHDSKASFRLNDEICLNVALKKVQVILNKLSRLQD
LHLSLCNVFEDLAGFYAQSMLLCVWYIFVSMILSAFYVTKPIITGNTGLSVVMYLRTVIHF
LHHTSLLIMLTKCVTDLIAEREKTGKIISVWLAKIDNQQFEKKLTKFSIYLMHQKVKFSVF
GIFSLDNSILLSIIGTITTYLIILQQENLSSNSNNSNCH

>NvitGR26
MHIRNIHCKITIGKVLHYSLKIFGLAPFSLDVEFLSNNKNQVSSEALTCSQLGVIYNLILAVL
IMVITYLTFKISNKTHIFGSGTDLDMAIEAIKTVWACISSVIILFLFGTQQKKLVQCGNIMLM
IRERLITINETLYLENKFLWKSITKISLVVIVMYILIIVTLGGYIDLARLIYVIGATLCDVIIIFT
VIVYGIKLKMIKQLIKIINANINSISTEFNRASNNILQNNEMKNAQIICDKLSRLQNLHLLLF
NATEDLTNVYATSILLCTLYIFLSIILNLFYILKNVMTGVASLSAILIRHIFIQFIHCTCSLIILTN
SVTDLVLESNRTGKIVSEWLTKLKNSRVENEVTKFLLYLKEHELQFSVFGIFSLDTSMLLSI
TSSITTYLVILLQLQFQQN
>NvitGR25
VKMLHKNIVNKESIILKFIFYYFKLVGLSCVSFSSKSRLDICFLTSKLGALYNVILALLITCFN
YYVVLIVVKVSFGSLHFDRAIDFGRVCLAVISSVFILITYCFKRKKATIILNRINTIAELSVNL
RSNGKSGHDGLCKPAKRIFFVFLVTWLVLIAVTPKLKFYALLYFAAQYSCEMVIMCMLVQ
YSMLLSILKQLFETINARFTISSEANFQVRRFSQHRSNEFGLEVKLNRFSYLRELHLSLCEV
AEDLSQFYSQSLLFCIAYVFSSLVLYAYFFVKIVTQKGDGIINTATTRFIIVKLLHYIGPTVTV
TWAACAVVNESNRTGKIVNKWMGDSRDQYVAIKLNQFSNYLLHQKLSFRAAGLFSLDGT
LMMSIAASITTYIMILLQFQDSTKR
>NvitGR22
MFIKCLFYAFKIFGLAPMVIDTTSTEKNNKEAHKIIFLSSKLGVLYNAALAILITLPTYLAITF
AYSDYVGRLEFEKITDTIQSVFTIFTSVFILINVCVHQKRAVDLANRLQTVNYLSMTKVSCS
DKSVKLLSSIKRIVLANAVTTILYFAVTPSNETRVLIYFLVINVYNTIIQATLMQYSLILKLLH
HIYRSLNSELSSLNKSLIFVGEFNQFLNNSVQTILRRLQTVWVTHLLLSHVSREVSDFYSLP
MLLCLSNAFLTLIMYSYHFVRALFIWKEHSGNMVVLILRTFMQVVTVAVSLTILTRAAGLT
VSESKRTGEIVSESTVYGHNRQIRSRLKEFADYLLHKELKFCVFNLFALNEELVMSIAGSIS
TYLVILVQFNETSSIEN
>NvitGR20
MIVSKCVFYFFKIFGLATMRLDENETVRDSWCSGSKKGQVYNAILTCSIIASNCYVARLVY
KENLSHREFEKTFDVVQYVYTTVTVAVILTVFCFCQGRAVLIANNLRKTYVLVENINSQMS
KKEEDPVISGLKRIWITSTVIWISVVFTTSKLQFAVVMYYMTVYPCILIVNCAFLQYTIILHL
LKQLFTILNANFLYVSTRQSVVTRKVEAAHSSFQAAEQSSQQFSDLRRLYMLLCDLSMDV
SKFYHLIMLFCVTYVFSTLTMWLYYITAPLVTGTTPSKLQYIHSLMIVTYHIFMLIILTKSVD
AVVQEKNKRTGEITNGWLANLQNQQLINELNLFSNYLLHKNVSFTAYGLFSLDESLLMSIT
GSITTYLVILLQFQ
>NvitGR13
MQSRHTYTKLFVRCFLRIIGLFPLPLFLDINGNPIFSYLGLFFNLCLVFAYIFMSIIAFQKRMTLV
LPKETVVAQIVDMIADGLENLNIISCLLVVAFRQKCLVKFYEKLKAIDLRLYDINVKHCKTF
DINMNIISLGRKLTIVGISFFIICTVDHLRLLFDNYLLSIRFWIAYQSTKIVIYNLIIVFCETMIF
FRKSFAKLNLLFQQSSSSYDKIQDIGQLHKILSELVDDFVGFYSFMVGSTIVHNFIHLSSNM
YRIYLFLKFNGDWSLLDFLDFTSIMIWLNVKILILYFLCALPAAVSEEANKKSIFIHRMLKAV
RENDLTSNNKRIARLLTILYQTNLEISVYGVFILDFKCFQSILTTSTMYIVFMIQLEHLK
>NvitGR12
MKKVNGIHIIKTLYFYEKLMGLCPFILSNKIVIKFSYIGAVYNLLITLIYTYYFILIIGLRFELH
LTRESTLSIALDAFGLAFQYCSIVSAWLTLTFRQECLKKILVTFAKVNLLANNLSMTLTRYC
LRKLQYIAVRLMLINLMYIVIFLSEHYLLKTYKKFEEHASTWIWFNLPKLVIYNIFGIFIELM
IILQQDYRALNKVISYSFSEKIDATSFCNFSESPGVISKKLCTIAEFHENLSDILEYTTNLFSLP

LLFALLASFLHLTLDSYIVYQHLLSKRMWEFNDFSSYVICLVWISTKILGFYFLCSVPDSTS
AEANHTVIILIKIINNCYKVRSCRDMMKKLMLQFKQKKNYASLYGLFSLDYYLFKNIMSTS
VMFLVFMFQLDDLVT
>NvitGR9
GKKWKIFSATDFLSLIKPSLLVCRFFGLISYKILNGKIEQSKNCGSYCAIVTFVYICASLLILY
IINVSPYMNRASTWMLQGNCFYTLVNFMLVSNFVFKSSTIKILQNLADTTAKLPSEKFVKIS
KWIHSKDLVLYLLLLLHVPKVFVGNIYAVLSKIIGTYAAMTIYLLDFQYNSYVFIIASCFEHI
NEELVQLNYNACKERGHLLRRVYHHQFNPLLFVKLRYLKQWHYELNEIIRKINSNFSLQV
VATVIMTFTELTFGLYFYILDRRHKVRSLDKEIWYFYYHTMVMYFSTKLLLLTLTCQYANN
ENYKTRTIVNEIIISTDNKLFKEEIYLFSLQLLHTDNKFIAKGVQLDATLLTGMAKGIFTYLL
ILIQFLITN
>NvitGR8
CKNLTFVQITNIYQLMRPYFFLYKLYGLFPYKISKNQIHSSKIGLCHTFFVAMSCIVYFVIAM
YQCFYSLDIVFDTTESLMQFTSYFMLGTFIAVYSCASNKYKFLLLKKLILLSSMLSEKEFFE
VAKVIYFKDIIGYIFLMGQIFNIASEDLTAQNISKMFALHITMIVFLMDMQYSNFVFLLKSCL
KNVNNNLQLLTKSYEGCEIISCNKSMQLLQFNNLQLIKLRKLQHNHHHVSCVIKELNTVF
TLQIIATVLMTFAEVTFGLYFFILHIQGKKGIDLDKQLWFNYFITSVTYYSLKMAVMVWIC
QETKNESLKTGIIVHDVILNNNNEQLKSELSLFSLQLLQCNNEFTSKCIVMNANLISGVVSG
IATYLLILIQFLNTKKSTSKNNEQ
>MmedGR6
RSGLMRNSDSLHVALRPVITLAQCFALFPVNGINAPDASGLSFTWGSFKILYCALTLIMSAF
MTVASIIRILSTKFHTTKITTLVFSVTSCLTSLMFLKLARKWPKFAKSWEKIEGELTIRYNQP
SKYSLVKRFKVVTIIIVTLAFLEHALSLASGYISARECACLLGDNDVAAIYFKTQFPQVFNK
TNYALWKGIVVQCTNLLSTFSWNFMDLFLILLSTALTYHFNLLNKRLNNVKNKTMPEWW
WAEARSDYNNLASLTRQVDSYVAHIVLLSFGTDLYFICIQLMYSFDRMTSVMRTIYLSYSF
GFLLGRTTAVSLTAASVHDESLLPAPVLYGVNGSSYSSEVIRFLTQVTTDNIGLTGMKFFSIT
RSFVLTVAGTIVTYELVLIQFNNVQQVNHLNLTNVCEVK
>MmedGR64f
KKKQFLGENSPLYTSVCPLVYIIRGFGLVPYEFEDNQLVPCDSYMIISFFWLFMYTYIVSGFI
IEFIESEKNRKKVLLYAEQARTVFNFAVVISDLLLCMRTRKEITWIWNKIQDYDQAMRDLG
YAKNEKSARMWVWFIIGGNTIIWAVVSSSGMNAFNEPWLHNVSFMIIYVGAAAAITKFSG
LVMILGDRFKQLNEIARSSVQRSRWIHSYPIIDDKLIDCLHSELTVIGNNINKVYKFSLLLWC
ANLSFHSVCCGYFVLNWLLDGNFRWKYIECLTAWFVASVYQLFLIHYSCHYTSSEANCMS
YIMLGWKRWLYTHDSKMEVETSIHLVNRQLHFSAAGCFYVNLPLLHSTAAILTTYMVILL
QID
>AmelGR1
MRPIIMLAQFFSLFPVSGVNSPDSSYLRFTWRSPKFIYCTISFLSSSIMTIFNVLRIVTTGISSI
KMTTFVFNGTNLIASFLFLKLAMRWPCLMVTWEKLEKELSQRHRKISKISLSMKFKIVTIV
VMTFALVEHSLSIIHGYFKAKECIEFHREQSILGVYFQMQFPQIFSRTSYSLWKGILVDIINIL
STFSWNFVDLFLILISIALTDQFRQLNSRLYSIRGKAMPEWWWAEARSDYNHLATLTRQLD
SHISIMVLLSFATDLYFICIQLLFSFNPMRGIIEKIYFGFSFGFLLARTTVVSLCAATIHDESLLP
APILYSVSSSSFSTEVMRFLSQVTTDNICLTGMKFFSVTRSLVLTVAGTIVTYELVLVQFNTT
QQTDASNATIVCEVK
>AmelGR2

MHSEDQIQLMMLKTKDGLGEIPKGKGRGSNLKIWSSVMYHKDDNNIEDISANQENDLST
KRPRAERNYFRNSEALENFHCAIGPVLKAAQIFGMFPVSGIGSSSLSKLQFKIFSLLTMYSG
FIALMISFMTIVSMIHMLKTFNASTFQIRGGLGAATVGAVFYGNSLVGSILFFSLSSRWVSL
QYEWRAMERYIDSNSTEPTRLRWKFFIISTMVLVLSLIEHVLSIFNNIDGYEWNESNSTFHN
FLEIYTLRSHSFIFDTLNYNFVYGLYVFVVSKLATFTWNFTDLFIMLVATGLAERYKSLNKK
LAVTMTKCQAAFNWRELREDYAILSCIVKKVDDHISPIILLSFANNVYFICLQLLNGLSISD
KNSVLSEAYFFGSFAFLICRTCAVTLLTARIHDQSKQALPYLYNCSTSSYSVEVQRLQCQLA
TDDIALTGLRFFSITRNFMLAVAGAIITYEVVLLQFNGK
>AmelGR3
MEVKRVEEKRKILFNNELCQAIFPIYYLGKFCGLVPVRFFVHTSEGCQARLNIIDLIYSLCV
LVLLLSAEIWGLWRDLKDGWEYSTRLKSRTAVIATCSDVLGVMSLTVVCIVGSPFRWKYL
QLVINKLIEVDEKIGVSSAKVARRFTIVLTICSLSYLWFNSIIDFYTWNRKTKVDNKAMTGK
GPINYAPLYFMYTVIISTEIQYTVSTYNIGQRFIRLNTSLKDLFNANSNNNDNAIDYFRKCPE
TAAHDMDDKKIWNLKPKRQIVLGSYRLSRKLDENKMYVNNISELIMVHSSLCDAVSLINS
TFGVVILAVTVTCLLHLVITPYFLILQAGERHEWIFLIVQGGWCIFHITRMLIIVQPSYSAIAE
AKKTAVLVSQLLSCTFEANIRRELEIFSLQLLHRPLEFSACGLFSLDRNLITSIAGVVTTYLVI
LIQFQNADDTKDDFDIIRNATQILKNASPLQNFTGLKTIV
>AmelGR4
MVDKLYWSRNMHLAIMLFLFFFKFFGLATFSLNNRRNSKIWSSKNVVLFVNSKLGILYNL
FVSFLIITLNFSLMPLIFYAEYAFRTNITIILETFQALLGSSVIILTLLSYCIFQSVIKEIGNYLIRI
ELILHRLQQPINQKYIFNLLFFVCLFKFIIFVALLFTEIIYFKPEPITLLGNLIPTIFAGLLFVQY
FFVITLINEMFIKLNCIMQNFYQNRLDDFNSNILYQNRRIFLNCSRIHLLLQIRNIHDHLCNIS
REISQFYAFPTLTGLCFIFFTSLYIIYFFLAIFLKNINVDLILVINGILWIILLLCPFGLLTSKITKI
VNEIEKTGCIVHILLNCVIDQKVKKELKQFSFQLLHQKIIFSTNGYFTLDNKFFQSMMSTMI
TYLVILLQFQTQTLNENVNSCNCMECL
>AmelGR5
MVDKSYWSRNMDRLTLLFFFFFKFAGLATFSLNNRANLNKKNSENTMFFISSKLGILYNLF
GSCLIIALSFYSIPITMYADYVHKTIVTITIEIFLIILGCFVMTSTLLFYCIFESVIIRIGNYLINIE
NVLRHLQQPLNRKHIFNVLFFICLFMLILLIILLITEIKNFNPNPLVLMASIIPLIFVGLLFIQYF
FVLNLIYAIFVKLNCIIQSFCRTRYDDINFKILNQTRCVFMSYSKIQLFIQIRDIYDHLCDISRE
VSDFYSFSILTALSFIFLVILYNFYYLFLHFVNDKLNFLLITNAIIWIMLPLSLLALLTSKVTNV
INEIEKTGCIIHVLLNCTIDRETKIELEQFSLQLLHQKVKFTANGYFTLDNTLFQSMINTVTT
YMVILFQFQMEISNENDKFCNCTQCR
>AmelGR6
MMKSIRKELARYPAKRVLPLTKPRLKPFDVETTFAEVTLARKEHTRKYHGPDSLLYSAIYP
VVCVMKVFGLAPYDFTGDEITPSNACLIFSFVFIGIYCHIIYIVYKRFLNVRRDKAILSVVET
TKVIPTVLRDLMVTVNYLVAMHDLIFTIFTRKTFSRIWNAQQDFDERLSQLGYPRKETKIKI
AAWILLASQIVIWTAVNQSGMFAFEETWTFNVSYMCTYIGTATAVYKFFGMASFLGLRFH
QLNQIAKENLPPRVGYKSSNVSRQKTIQDLHNDLMLSSETLGSLYSWSLLFWLGNLSIHSV
SNLYFIIDWVILTPWTNIAWPLIINMWCWLIGFITQLLALHIACDYTINEIFNRKIPNSRLILW
APFSSNGMQGQYKNFHTKQDSFRTSLYLLNRRLRFSAGGLFDIKLSLLCSIAHVMSTYLIIL
LQFPSS
>AmelGR7
MYEPKTLKETIKPLMVVNFVFGMGLTGIEAKKPSKKLEYVYTLCNLVLFYFINKLTLPYY

DKYYVISTFELSRFIFQWMFHANIWTITLLIIITRIKAQQLRTIVSLVELSDQKMENIGLSPKH
RCLMMYQIKRYIFLGIYTLIFVVLIYHCHYESTTPALIKLCLSILPNIPFIVFGVSTISFCFWVT
CLKLKFRQLNELLRSMRIMESPIHKRVLEMTNNFENNRFALYRNEHVRRNTNTIRAVKQIH
LEIIKIVSFLNQTFGIQILIQMTVSVVFTTNLLHLLYRVIWLNFTLPELLQELISVIFWILIYGS
QILYVNHVCASTNSEAVNIGNIICEFYEPFATKEFQAEIRDFTLQLIQNPVVFTAYGFFNLDH
SFIQGVIGTITTYLVVMIQVGDLSNSDKSILS
>AmelGR8
MIDRLKCNGKRVKNCKRQSSALCFGNYASLIYYIAFINRFMGFLPYKLESSKFMYSKLYSV
FSTIIIIIYVFLAIFSLYEINFYIVMNDGLPRILHRNSIALFGPAVFICSYASNRSTLQAIQRVQH
VSHALSTEIFSKLIKTLLIKDMSFFMPLGTFILYMIVNYDFCVFLFICWYSFFGLLIISTLYLN
NVYILNACFKFINNSLIKIKEIVINDEPHLLRREYHMKKNPILLMELRILKKQYTELIEAVQL
LNHSYSLKNEMILLMLFFDITFNLYIYLILYNTEVGKITKINSTLGFAIFYIVYIVISISMIEIIR
VQMKKIGSNIHKILVHTFDDQIITELELFSLEVLQKDNKFIMFGLEMDLTLVTDMTCKITTF
LLMLIQFLFVPPC
>AmelGR9
MWLEKLKKHKIWKIFKATDFNSLMFPCFFICGIIGYFPYKWRRPVYLFSKTRFVISTFILLLS
IFIWFNLFYQLNFTDIIKKNTTEVIHENMYALLIGIGIITMHILSSTRLCMIQNLLKISSILSEK
DFNDLAKRIHKKDILGFLFILIHLPNCSKSDIHVTLRNVTHLYILFINFSVDMFYVNCIWVL
KVCFKKLNKSIHELKKFRSGNDLRVETIVQQKNSLLLIKLKHLEEKHLEISDVVQLVNDTFI
IHIIVLVITTFSTITFNLYFFLLKMYSPKTENIKFWFIPNLAPALFFFIKFAMIIWICESTTNEAK
KIKWTLYDAFSDSTDPMVRREVHLFSLQIMHRNNKFTAKTFDMNSVLLGQITKGVLIYILI
LFQFLLNYRLCS
>AmelGR10
MVLVGDTNLIFNAGLILGCTALCASKDNVKARRLRDIIFIVFSMTLYLVSLVVIYIYVFSYE
DSDLKSTLIIIIRVFLIYLCLFTDASVTTLWNWKIRSVLSQLRNFDRATKFRDFSKGNKLRIIC
HVTMFVSFSYWAIVGYFTYRIEAKVPIFHGIIYFIMDASMNTQILIFVCILFLIEERFRHLCS
MIELSKADKIIEAHRSIRHSTLQQIWWLHCSLANATEIINSVYAIQLLFWISSMSFNLMSRIY
SLKVFKLSDYGKIRESMLVTDCAWNLVLITTVCHMTAHQVDLSLNQTEISPISRRTYGDITL
LFVQANRVGELIFSPYSSVSLKRVHLQENIEAAAYFQLRKVHLFTVAGLIRVDLPLLLSIFSA
LTTYLVILT
>AmelGRX
MKDAIDIFKLKILTRIPKTQYASLLPIIIINEIFGLRVFELKGRLRYGWSIIYVGICIILYGIFLNA
IINTDYKNWLAYQEIIYKLTSFINIMCVMIFIILGMLNTERSRKIIARCEQIDNILQSFGIEKNY
RKIFLQLIQILIIFSIIITSIISLNWYWYIYTYKLQEFNLILLLYIPIITTTLGIFTFTVFVRHIHFEV
LKLSHQLNQAYTYQILMDFIVEFLLVLCTLYNFYFYLKSFNVSELLSNIGIIGFLIWTFLNLI
KIIFINNHCTNLYREVQITAHLLRQLEICYLDNSIRDEIVPETESNSNVQQFSLQLLLHPLQFT
AGGYILNNKLSMLFFSTITTYLIVLIQISSTPNLIQPLSYISSH
>AmelGRY
MKDTIEAFKKKILAWTPETQYASLIHIIIINEIFALRIFESKKQLRYGWSIIYASICIILYIVYLN
TIININYKKWFAYNEISYKLASYINITCVMVSIILGMLNTERSRKIIARCEQIDNTLKSFGIEK
NYRKTFFCVTQTLIISSVIIISIILLNYYSTIFVRYQVDLNIFIFILIITMNLGILTFTILISILQDKL
HKINKIIKEIYQLSNTNDINIKYNMQSLILCKFVIIMDYNNRKYFVQHFFQITRYIHFEVLKL
SRELNQAYTYQIVLDLIMQFSSLLSVLYNGYFYLISLKLSEVLSNKGFIGFFGLISLFIIKIVFL
NNHCTKFYHEAEVIAYLLRESEIYYLDNSIKDEVQQFSLQLLLYPLQFTAGGYILNNKLSM

LFFSTITTYLIVLIQISSTPNLIQPLSYISSH
>AmelGRZ
ZLTHLKEKSPETQYVSLIYIIIINEIFGLRVFESKRRLQYDWSIIYTNICNILYAIYTNEIININY
KNWPAYQEISYKLIMYINIIGIIIFMVLGMLNTERSRRIITKCEQIDNILESFGIEKNYKKNFL
VIQILIISKTISMILLNFFYITSKEERZKFNLSILIYIIIAIMYLGKCIFYFNNIILHDKLHKMNKL
IKEVYELSNTKDIDIRYEIQLYIAHKFIIIMDCYNKKHFVQHFLQLIRYIHFEILKLSRELNQA
YTYQILFKLIMQFSLLLFTFYNEYFHLMSLKLSEALSDKQNIGFLIWIYLYSTKVIYINSQCT
KFYCEVEVIAYLLRKLDICYLDNFIKDQVQQVLLQLFLHPLKFTAGGYILNNRLSTIFFGIIIS
YLIVLIQISSSPSLTKPLSKNL
>DmelGR2a
MEFGMDTLRALEPLHRACQVCNLWPWRLAPPPDSEGILLRRSRWLELYGWTVLIAATSFT
VYGLFQESSVEEKQDSESTISSIGHTVDFIQLVGMRVAHLAALLEALWQRQAQRGFFAELG
EIDRLLSKALRVDVEAMRINMRRQTSRRAVWILWGYAVSQLLILGAKLLSRGDRFPIYWIS
YLLPLLVCGLRYFQIFNATQLVRQRLDVLLVALQQLQLHQKGPAVDTVLEEQEDLEEAAM
DRLIAVRLVYQRVWALVALLNRCYGLSMLMQVGNDFLAITSNCYWMFLNFRQSAASPFDI
LQIVASGVWSAPHLGNVLVLSLLCDRTAQCASRLALCLHQVSVDLRNESHNALITQFSLQL
LHQRLHFSAAGFFNVDCTLLYTIVGATTTYLIILIQFHMSESTIGSDSNGQ
>DmelGR5a
MRQLKGRNRCNRAVRHLKVQGKMWLKNLKSGLEQIRESQVRGTRKNFLHDGSFHEAVA
PVLAVAQCFCLMPVCGISAPTYRGLSFNRRSWRFWYSSLYLCSTSVDLAFSIRRVAHSVLD
VRSVEPIVFHVSILIASWQFLNLAQLWPGLMRHWAAVERRLPGYTCCLQRARPARRLKLV
AFVLLVVSLMEHLLSIISVVYYDFCPRRSDPVESYLLGASAQLFEVFPYSNWLAWLGKIQN
VLLTFGWSYMDIFLMMLGMGLSEMLARLNRSLEQQVRQPMPEAYWTWSRTLYRSIVELI
REVDDAVSGIMLISFGSNLYFICLQLLKSINTMPSSAHAVYFYFSLLFLLSRSTAVLLFVSAIN
DQAREPLRLLRLVPLKGYHPEVFRFAAELASDQVALTGLKFFNVTRKLFLAMAGTVATYE
LVLIQFHEDKKTWDCSPFNLD
>DmelGR8a
MSGHLGRVLQFHLRLYQVLGFHGLPLPGDGNPARTRRRLMAWSLFLLISLSALVLACLFS
GEEFLYRGDMFGCANDALKYVFAELGVLAIYLETLSSQRHLANFWWLHFKLGGQKTGLV
SLRSEFQQFCRYLIFLYAMMAAEVAIHLGLWQFQALTQHMLLFWSTYEPLVWLTYLRNLQ
FVLHLELLREQLTGLEREMGLLAEYSRFASETGRSFPGFESFLRRRLVQKQRIYSHVYDML
KCFQGAFNFSILAVLLTINIRIAVDCYFMYYSIYNNVINNDYYLIVPALLEIPAFIYASQSCMV
VVPRIAHQLHNIVTDSGCCSCPDLSLQIQNFSLQLLHQPIRIDCLGLTILDCSLLTRMACSVG
TYMIYSIQFIPKFSNTYM
>DmelGR9a
MSLWLEHFLTGYFQLCGLVCGWSGSRLGRLLSSTFLVLILIELVGEIETYFTEENPDNESVPA
YFAKVIMGVNMAYKMIHAWIALSALFECRRFRYLLEELPPVKATSFIYRHLILEIILFACNA
FLVLSEYTIRGIYLENLRYAYSLQAVRARYLQMMVLVDRLDGKLEQLHHRVISGSSDYKTL
RLDYAHLAKVTRSLSHLFGLSLLLLNVLCLGDWIIVCNVYFMVAYLQVLPATLFLFGQVM
FVVCPTLIKIWSICAASHRCVSKSKHLQQQLKDLPGQTPVERSQIEGFALQIMQDPIQIDVC
GIYHLNLQTLAGMFFFILEALVIFLQFVSLVRT
>DmelGR10a
MTSPDERKSFWERHEFKFYRYGHVYALIYGQVVIDYVPQRALKRGVKVLLIAYGHLFSM
LLIVVLPGYFCYHFRTLTDTLDRRLQLLFYVSFTNTAIKYATVIVTYVANTVHFEAINQRCT

MQRTHLEFEFKNAPQEPKRPFEFFMYFKFCLINLMMMIQVCGIFAQYGEVGKGSVSQVRV
HFAIYAFVLWNYTENMADYCYFINGSVLKYYRQFNLQLGSLRDEMDGLRPGGMLLHHC
CELSDRLEELRRRCREIHDLQRESFRMHQFQLIGLMLSTLINNLTNFYTLFHMLAKQSLEE
VSYPVVVGSVYATGFYIDTYIVALINEHIKLELEAVALTMRRFAEPREMDERLTREIEHLSLE
LLNYQPPMLCGLLHLDRRLVYLIAVTAFSYFITLVQFDLYLRKKS
>DmelGR10b
MRVGKLCRLALRFWMGLILVLGFSSHYYNPTRRRLVYSRILQTYDWLLMVINLGAFYLY
YRYAMTYFLEGMFRRQGFVNQVSTCNVFQQLLMAVTGTWLHFLFERHVCQTYNELSRIL
KHDLKLKEHSRFYCLAFLAKVYNFFHNFNFALSAIMHWGLRPFNVWDLLANLYFVYNSL
ARDAILVAYVLLLLNLSEALRLNGQQEHDTYSDLMKQLRRRERLLRIGRRVHRMFAWLVA
IALIYLVFFNTATIYLGYTMFIQKHDALGLRGRGLKMLLTVVSFLVILWDVVLLQVICEKLL
AEENKICDCPEDVASSRTTYRQWEMSALRRAITRSSPENNVLGMFRMDMRCAFALISCSL
SYGIIIIQIGYIPG
>DmelGR21aNEW
MSFWAVSRGLTPPSKVVPMLNPNQRQFLEDEVRYREKLKLMARGDAMEEVYVRKQETV
DDPLELDKHDSFYQTTKSLLVLFQIMGVMPIHRNPPEKNLPRTGYSWGSKQVMWAIFIYS
CQTTIVVLVLRERVKKFVTSPDKRFDEAIYNVIFISLLFTNFLLPVASWRHGPQVAIFKNMW
TNYQYKFFKTTGSPIVFPNLYPLTWSLCVFSWLLSIAINLSQYFLQPDFRLWYTFAYYPIIA
MLNCFCSLWYINCNAFGTASRALSDALQTTIRGEKPAQKLTEYRHLWVDLSHMMQQLGR
AYSNMYGMYCLVIFFTTIIATYGSISEIIDHGATYKEVGLFVIVFYCMGLLYIICNEAHYASR
KVGLDFQTKLLNINLTAVDAATQKEVEMLLVAINKNPPIMNLDGYANINRELITTNISFMAT
YLVVLLQFKITEQRRIGQQQA
>DmelGR22a
MSQPKRIHRICKGLARFTIRATLYGSWVLGLFPFTFDSRKRRLNRSKWLLAYGLVLNLTLL
VLSMLPSTDDHNSVKVEVFQRNPLVKQVEELVEVISLITTLVTHLRTFSRSSELVEILNELLV
LDKNHFSKLMLSECHTFNRYVIEKGLVIILEIGSSLVLYFGIPNSKIVVYEAVCIYIVQLEVL
MVVMHFHLAVIYIYRYLWIINGQLLDMASRLRRGDSVDPDRIQLLLWLYSRLLDLNHRLT
AIYDIQVTLFMATLFSVNIIVGHVLVICWINITRFSLLVIFLLFPQALIINFWDLWQGIAFCDL
AESTGKKTSMILKLFNDMENMDQETERRVAEFTLFCSHRRLKVCHLGLLDINYEMGFRMI
ITNILYVVFLVQFDYMNLKFKTD
>DmelGR22b
MFGSSREIRPYLARQMLKTTLYGSWLLGIFPFTLDSGKRIRQLRRSRCLTLYGLVLNYFLIF
TLIRLAFEYRKHKLEAFKRNPVLEMINVVIGIINVLSALIVHFMNFWGSRKVGEICNELLIL
EYQDFEGLNGRNCPNFNCFVIQKCLTILGQLLSFFTLNFALPGLEFHICLVLLSCLMEFSLNL
NIMHYHVGVLLIYRYVWLINEQLKDLVSQLKLNPETDFSRIHQFLSLYKRLLELNRKLVIA
YEYQMTLFIIAQLSGNIVVIYFLIVYGLSMRTYSIFLVAFPNSLLINIWDFWLCIAACDLTEK
AGDETAIILKIFSDLEHRDDKLEMSVNEFAWLCSHRKFRFQLCGLFSMNCRMGFKMIITTF
LYLVYLVQFDYMNL
>DmelGR22cNEW
MFASRSDLQSRLCWIILKATLYSSWFLGVFPYRFDSRNGQLKRSRFLLFYGLILNFFLLLKM
VCSGGQKLGIPEAFARNSVLENTHYTTGMLAVFSCVVIHFLNFWGSTRVQDLANELLVLE
YQQFASLNETKCPKFNSFVIQKWLSVIGLLLSYLSIAYGLPGNNFSVEMVLINSLVQFSFNC
NIMHYYIGVLLIYRYLWLINGQLLEMVTNLKLDCSVDSSRIRKYLSLYRRLLELKGYMVAT
YEYHMTLVLTTGLASNFLAIYSWIVLDISMNINFIYLLIFPLFLLVNVWNLWLSIAASDLAE

NAGKSTQTVLKLFADLEVKDIELERSVNEFALLCGHCQFNFHVCGLFTINYKMGFQMIITS
FLYLIYMIQFDFMNL
>DmelGR22d
MFRPRCGLRQKFVYVILKSILYSSWLLGIFPFKYEPKKRRLRRSMWLIPFGVVISSSLLILM
VKQSAEDREHGIMLDVFQRNALLYQISSLMGVVGVVSICTVHLRTLWRSKHLEEIYNGLM
LLEAKYFCSNAVECPAFDGYVIQKGVVIVVGLLAPWMVHFGMPDSKLPVLNVLVVSMVK
LGTLLLALHYHLGVVIIYRFVWLINRELLSLVCSLRGNHKGSSSRVRFLLKLYNKLVNLYS
KLADCYDCQTVLMMAIFLAANIIVCFYMIVYRISLSKMSFFVMLIMFPLAIANNFMDFWL
SMKVCDLLQKTGRQTSMILKLFNDIENMDKDLEISISDFALYCSHRRFKFLHCGLFHVNRE
MGFKMFVASVLYLLYLVQFDYMNL
>DmelGR22e
MFRPSGSGYRQKWTGLTLKGALYGSWILGVFPFAYDSWTRTLRRSKWLIAYGFVLNAAFI
LLVVTNDTESETPLRMEVFHRNALAEQINGIHDIQSLSMVSIMLLRSFWKSGDIERTLNELE
DLQHRYFRNYSLEECISFDRFVLYKGFSVVLELVSMLVLELGMSPNYSAQFFIGLGSLCLM
LLAVLLGASHFHLAVVFVYRYVWIVNRELLKLVNKMAIGETVESERMDLLLYLYHRLLDL
GQRLASIYDYQMVMVMVSFLIANVLGIYFFIIYSISLNKSLDFKILVFVQALVINMLDFWLN
VEICELAERTGRQTSTILKLFNDIENIDEKLERSITDFALFCSHRRLRFHHCGLFYVNYEMG
FRMAITSFLYLLFLIQFDYWNL
>DmelGR22f
MKMFQPRRGFSCHLAWFMLQTTLYASWLLGLFPFTFDSRRKQLKRSRWLLLYGFVLHSL
AMCLAMSSHLASKQRRKYNAFERNPLLEKIYMQFQVTTFFTISVLLLMNVWKSNTVRKI
ANELLTLEGQVKDLLTLKNCPNFNCFVIKKHVAAIGQFVISIYFCLCQENSYPKILKILCCLP
SVGLQLIIMHFHTEIILVYRYVWLVNETLEDSHHLSSSRIHALASLYDRLLKLSELVVACND
LQLILMLIIYLIGNTVQIFFLIVLGVSMNKRYIYLVASPQLIINFWDFWLNIVVCDLAGKCGD
QTSKVLKLFTDLEHDDEELERSLNEFAWLCTHRKFRFQLCGLFSINHNMGFQMIITSFLYL
VYLLQFDFMNL
>DmelGR23aA
MKTLECLTRRFLEVIFSVLALVPLPPISQLGWLFLSLAIRCCWIVYFIYLLDVAISFSWVAIEN
VGNAVGTMLFVGNSVLGFALLLESVLKQKTHSQLEDLRVQTELQLQRLGMFGRSRHAAY
LLPLIGVQFTCDLVRLATNFGETVSPVFCISLPLMWLLRYRYVQLVQHVMDLNQRSIHLRR
SLLSMASGNDLWQPYGVQECLQLQTLRTTYERIFECYETFSDCYGWGMLGLHLLTSFQFV
TNAYWMIMGIYDGGNVRSLIFNGATGIDFGTPIATLFWHGDSGAENGRQIGCLISKLVKPQ
GSKLYNDLVSEFSLQTLHQRFVVTAKDFFSLNLHLLSSMFAAVVTYLVILIQFMFAERSSTR
GSG
>DmelGR23aB
MFPPTRVQASSRVVLKIFHFILVAFSLRSRRLSRLVLWLQFLGWLTWFISMWTQSVIYAQTI
DCTLDCSLRHILTFFQTVSHAFIVVTSFLDGFRIKQDQLDEPIAFEDSDPWLAFTVLAMLVP
TLGVEYLVCSNAPEYAFRIRIYHLKTLPSFLALQVQIISFILEVMKVNIRVRQTKLQLLILAR
ELSCRWPQRKQKPQFSDQQAHRVKDLKRRYNDLHYLFVRINGYFGGSLLTIIIVHFAIFVS
NSYWLFVDIRTRPWRIYAILLNLGFIFNVALQMAAACWHCQQSYNLGRQIGCLISKLVKPQ
GSKLYNDLVSEFSLQTLHQRFVVTAKDFFSLNLHLLSSMFAAVVTYLVILIQFMFAERSSTR
GSG
>DmelGR28a
MAFKLWERFSQADNVFQALRPLTFISLLGLAPFRLNLNPRKEVQTSKFSFFAGIVHFLFFVL

CFGISVKEGDSIIGYFFQTNITRFSDGTLRLTGILAMSTIFGFAMFKRQRLVSIIQNNIVVDEIF
VRLGMKLDYRRILLSSFLISLGMLLFNVIYLCVSYSLLVSATISPSFVTFTTFALPHINISLMV
FKFLCTTDLARSRFSMLNEILQDILDAHIEQLSALELSPMHSVVNHRRYSHRLRNLISTPMK
RYSVTSVIRLNPEYAIKQVSNIHNLLCDICQTIEEYFTYPLLGIIAISFLFILFDDFYILEAILNP
KRLDVFEADEFFAFFLMQLIWYIVIIVLIVEGSSRTILHSSYTAAIVHKILNITDDPELRDRLF
RLSLQLSHRKVLFTAAGLFRLDRTLIFTITGAATCYLIILIQFRFTHHMDDTSSNSTNNLHSI
HLGD
>DmelGR28bA
MIRCGLDIFRGCRGRFRYWLSARDCYDSISLMVAIAFALGITPFLVRRNALGENSLEQSWY
GFLNAIFRWLLLAYCYSYINLRNESLIGYFMRNHVSQISTRVHDVGGIIAAVFTFILPLLLRK
YFLKSVKNMVQVDTQLERLRSPVNFNTVVGQVVLVILAVVLLDTVLLTTGLVCLAKMEV
YASWQLTFIFVYELLAISITICMFCLMTRTVQRRITCLHKVLKNLAHQWDTRSLKAVNQKQ
RSLQCLDSFSMYTIVTKDPAEIIQESMEIHHLICEAAATANKYFTYQLLTIISIAFLIIVFDAYY
VLETLLGKSKRESKFKTVEFVTFFSCQMILYLIAIISIVEGSNRAIKKSEKTGGIVHSLLNKT
KSAEVKEKLQQFSMQLMHLKINFTAAGLFNIDRTLYFTISGALTTYLIILLQFTSNSPNNGY
GNGSSCCETFNNMTNHTL
>DmelGR28bB
MSALRRVRKYFISSQVYEALRPLFFLTFLYGLTPFHVVRRKMGESYLKMSCFGVFNIFIYIC
LCGFCYISSLRQGESIVGYFFRTEISTIGDRLQIFNGLIAGAVIYTSAILKRCKLLGTLTILHSL
DTNFSNIGVRVKYSRIFRYSLLVLIFKLLILGVYFVGVFRLLVSLDVTPSFCVCMTFFLQHSV
VSIAICLFCVIAFSFERRLSIINQVLKNLAHQWDTRSLKAVNQKQRSLQCLDSFSMYTIVTK
DPAEIIQESMEIHHLICEAAATANKYFTYQLLTIISIAFLIIVFDAYYVLETLLGKSKRESKFKT
VEFVTFFSCQMILYLIAIISIVEGSNRAIKKSEKTGGIVHSLLNKTKSAEVKEKLQQFSMQL
MHLKINFTAAGLFNIDRTLYFTISGALTTYLIILLQFTSNSPNNGYGNGSSCCETFNNMTNH
TL
>DmelGR28bC
MDIEMAKEPVNPTDTPDIEVTPGLCQPLRRRFRRFVTAKQLYECLRPVFHVTYIHGLTSFYI
SCDTKTGKKAIKKTIFGYINGIMHIAMFVFAYSLTIYNNCESVASYFFRSRITYFGDLMQIVS
GFIGVTVIYLTAFVPNHRLERCLQKFHTMDVQLQTVGVKIMYSKVLRFSYMVLISMFLVN
VLFTGGTFSVLYSSEVAPTMALHFTFLIQHTVIAIAIALFSCFTYLVEMRLVMVNKVLKNLA
HQWDTRSLKAVNQKQRSLQCLDSFSMYTIVTKDPAEIIQESMEIHHLICEAAATANKYFTY
QLLTIISIAFLIIVFDAYYVLETLLGKSKRESKFKTVEFVTFFSCQMILYLIAIISIVEGSNRAIK
KSEKTGGIVHSLLNKTKSAEVKEKLQQFSMQLMHLKINFTAAGLFNIDRTLYFTISGALTT
YLIILLQFTSNSPNNGYGNGSSCCETFNNMTNHTL
>DmelGR28bD
MSFYFCEIFKPRDAFGAEQTLLLYTYLLGLTPFRLRGQAGERQFHLSKIGYLNAFLQLSFFS
YCFLAALIEQQSIVGYFFKSEISQMGDSLQKFIGMTGMSILFLCSSIRVRLLIHIWDRISYIDD
RFLNLGVCFNYPAIMRLRLLQIFLINGVQLGYLISSNWMLLGNDVRPIYTAIVAFYVPQIFL
LSIVMLFNATLHRLWQHFTVLNQVLKNLAHQWDTRSLKAVNQKQRSLQCLDSFSMYTIV
TKDPAEIIQESMEIHHLICEAAATANKYFTYQLLTIISIAFLIIVFDAYYVLETLLGKSKRESKF
KTVEFVTFFSCQMILYLIAIISIVEGSNRAIKKSEKTGGIVHSLLNKTKSAEVKEKLQQFSMQ
LMHLKINFTAAGLFNIDRTLYFTISGALTTYLIILLQFTSNSPNNGYGNGSSCCETFNNMTN
HTL
>DmelGR28bE

MWLLRRSVGKSGNRPHDVYTCYRLTIFMALCLGIVPYYVSISSEGRGKLTSSYIGYINIIIR
MAIYMVNSFYGAVNRDTLMSNFFLTDISNVIDALQKINGMLGIFAILLISLLNRKELLKLLA
TFDRLETEAFPRVGVAMHQVAANKKMNRLVIILVGSMVAYITCSFLMISLRDTTTFSISAVIS
FFSPHFIVCAVSFLAGNVMIKLRIYLSALNEVLKNLAHQWDTRSLKAVNQKQRSLQCLDSF
SMYTIVTKDPAEIIQESMEIHHLICEAAATANKYFTYQLLTIISIAFLIIVFDAYYVLETLLGKS
KRESKFKTVEFVTFFSCQMILYLIAIISIVEGSNRAIKKSEKTGGIVHSLLNKTKSAEVKEKL
QQFSMQLMHLKINFTAAGLFNIDRTLYFTISGALTTYLIILLQFTSNSPNNGYGNGSSCCETF
NNMTNHTL
>DmelGR32aNEW
MSPNTWVIEMPTQKTRSHPYPRRISPYRPPVLNRDAFSRDAPPMPARNHDHPVFEDIRTILS
VLKASGLMPIYEQVSDYEVGPPTKTNEFYSFFVRGVVHALTIFNVYSLFTPISAQLFFSYRE
TDNVNQWIELLLCILTYTLTVFVCAHNTTSMLRIMNEILQLDEEVRRQFGANLSQNFGFLV
KFLVGITACQAYIIVLKIYAVQGEITPTSYILLAFYGIQNGLTATYIVFASALLRIVYIRFHFIN
QLLNGYTYGQQHRRKEGGARARRQRGDVNPNVNPALMEHFPEDSLFIYRMHNKLLRIYK
GINDCCNLILVSFLGYSFYTVTTNCYNLFVQITGKGMVSPNILQWCFAWLCLHVSLLALLS
RSCGLTTTEANATSQILARVYAKSKEYQNIIDKFLTKSIKQEVQFTAYGFFAIDNSTLFKIFSA
VTTYLVILIQFKQLEDSKVEDPVPEQT
>DmelGR33aNEW
MIQIMNWFSMVIGLIPLNRQQSETNFILDYAMMCIVPIFYVACYLLINLSHIIGLCLLDSCNS
VCKLSSHLFMHLGAFLYLTITLLSLYRRKEFFQQFDARLNDIDAVIQKCQRVAEMDKVKVT
AVKHSVAYHFTWLFLFCVFTFALYYDVRSLYLTFGNLAFIPFMVSSFPYLAGSIIQGEFIYHV
SVISQRFEQINMLLEKINQEARHRHAPLTVFDIESEGKKERKTVTPITVMDGRTTTGFGNEN
KFAGEMKRQEGQQKNDDDDLDTSNDEDEDDFDYDNATIAENTGNTSEANLPDLFKLHD
KILALSVITNGEFGPQCVPYMAACFVVSIFGIFLETKVNFIVGGKSRLLDYMTYLYVIWSFT
TMMVAYIVLRLCCNANNHSKQSAMIVHEIMQKKPAFMLSNDLFYNKMKSFTLQFLHWE
GFFQFNGVGLFALDYTFIFSTVSAATSYLIVLLQFDMTAILRNEGLMS
>DmelGR36a
MFDWVGLLLKVLYYYGQIIGLINFEIDWQRGRVVAAQRGILFAIAINVLICMVLLLQISKKF
NLDVYFGRANQLHQYVIIVMVSLRMASGISAILNRWRQRAQLMRLVECVLRLFLKKPHV
KQMSRWAILVKFSVGVVSNFLQMAISMESLDRLGFNEFVGMASDFWMSAIINMAISQHYL
VILFVRAYYHLLKTEVRQAIHESQMLSEIYPRRAAFMTKCCYLADRIDNIAKLQNQLQSIV
TQLNQVFGIQGIMVYGGYYIFSVATTYITYSLAINGIEELHLSVRAAALVFSWFLFYYTSAI
LNLFVMLKLFDDHKEMERILEERTLFTSALDVRLEQSFESIQLQLIRNPLKIEVLDIFTITRSS
SAAMIGSIITNSIFLIQYDMEYF
>DmelGR36b
MVDWVVLLLKAVHIYCYLIGLSNFEFDCRTGRVFKSRRCTIYAFMANIFILITIIYNFTAHGD
TNLLFQSANKLHEYVIIIMSGLKIVAGLITVLNRWLQRGQMMQLVKDVIRLYMINPQLKS
MIRWGILLKAFISFAIELLQVTLSVDALDRQGTAEMMGLLVKLCVSFIMNLAISQHFLVILLI
RAQYRIMNAKLRMVIEESRRLSFLQLRNGAFMTRCCYLSDQLEDIGEVQSQLQSMVGQL
DEVFGMQGLMAYSEYYLSIVGTSYMSYSIYKYGPHNLKLSAKTSIIVCILITLFYLDALVNC
NNMLRVLDHHKDFLGLLEERTVFASSLDIRLEESFESLQLQLARNPLKINVMGMFPITRGS
TAAMCASVIVNSIFLIQFDMEFF
>DmelGR36c
MDLESFLLGAVYYYGLFIGLSNFEFDWNTGRVFTKKWSTLYAIALDSCIFALYIYHWTGNT

NIVNAIFGRANMLHEYVVAILTGLRIVTGLFTLILRWYQRCKMMDLASKVVRMYVARPQ
VRRMSRWGILTKFIFGSITDGLQMAMVLSAMGSVDSQFYLGLGLQYWMFVILNMAMMQ
QHMIMLFVRTQFQLINTELRQVIDEAKDLLLSPRHQGVFMTKCCSLADQIENIARIQSQLQ
TIMNQMEEVFGIQGAMTYGGYYLSSVGTCYLAYSILKHGYENLSMTLSTVILAYSWCFFY
YLDGMLNLSVMLHVQDDYWEMLQILGKRTIFVGLDVRLEEAFENLNLQLIRNPLKITVV
KLYDVTRSNTMAMFGNLITHSIFLIQYDIEHF
>DmelGR39aA
MGTRNRKLLFFLHYQRYLGLTNLDFSKSLHIYWLHGTWSSTAIQIVVVGVFMAALLGALA
ESLYYMETKSQTGNTFDNAVILTTSVTQLLANLWLRSQQKSQVNLLQRLSQVVELLQFEP
YAVPQFRWLYRIWLLVCLIYGAMVTHFGINWLTTMQISRVLTLIGFVYRCVLANFQFTCYT
GMVVILKKLLQVQVKQLEHLVSTTTISMAGVAGCLRTHDEILLLGQRELIAVYGGVILFLFI
YQVMQCILIFYISNLEGFHSSNDLVLIFCWLAPMLFYLILPLVVNDIHNQANKTAKMLTKV
PRTGTGLDRMIEKFLLKNLRQKPILTAYGFFALDKSTLFKLFTAIFTYMVILVQFKEMENST
KSINKF
>DmelGR39aB
MDFQPGELCAYYRLCRYLGIFCIDYNPTKKKFRLRRSVLCYIVHFALQAYLVGCISVMVTY
WRRCFKSELTTTGNHFDRLVMVIALGILVVQNAWLIWLQAPHLRIVRQIEFYRRNHLANV
RLLLPKRLLWLIIATNVVYMANFIKTCIFEWLTDASRLFVITSLGFPLRYLVTSFTMGTYFC
MVHIVRLVLDWNQSQINAIIDESADLKMTSPNRLRLRVCLEMHDRLMLLCNDEISLVYGFI
AWLSWMFASLDVTGVIYLTMVIQTKKSIVLKLITNVVWLSPTFMTCAASFMSNRVTIQAN
KTAKMLTKVPRTGTGLDRMIEKFLLKNLRQKPILTAYGFFALDKSTLFKLFTAIFTYMVILV
QFKEMENSTKSINKF
>DmelGR39aC
MKRNAFEELRVQLRTLKWLGVLRFTIDFNKCLVRENASEERSAWLYLIGVVGITCSLIVYS
TYFPSHFIMGKHNTTGNCYALINIRSCSIVTMLIYTQLYIQRFRFVALLQSILRFNQISGSHRE
EGRFAFYYYTHLSLLIICMLNYAYGYWTAGVRLTTIPIYLLQYGFSYLFLGQVVVLFACIQQ
ILLSILKYYNQVVLKNIKSSKESREFYYNFCKYNQVIWLSYTEINHCFGLLLLLVTGLILLIT
PSGPFYLVSTIFEGRFRQNWQFSLMSFTAILWSLPWIVLLVLAMGRNDVQKEANKTAKML
TKVPRTGTGLDRMIEKFLLKNLRQKPILTAYGFFALDKSTLFKLFTAIFTYMVILVQFKEME
NSTKSINKF
>DmelGR39aD
MSKVCRDLRIYLRLLHIMGMMCWHFDSDHCQLVATSGSERYAVVYAGCILVSTTAGFIFAL
LHPSRFHIAIYNQTGNFYEAVIFRSTCVVLFLVYVILYAWRHRYRDLVQHILRLNRRCASSC
TNQQFLHNIILYGMLTILCFGNYLHGYTRAGLATLPLALCMLVYIFAFLVLCLLLMFFVSLK
QVMTAGLIHYNQQLCQGDLISGLRGRQQILKLCGGELNECFGLLMLPIVALVLLMAPSGPF
FLISTVLEGKFRPDECLIMLLTSSTWDTPWMIMLVLMLRTNGISEEANKTAKMLTKVPRTG
TGLDRMIEKFLLKNLRQKPILTAYGFFALDKSTLFKLFTAIFTYMVILVQFKEMENSTKSIN
KF
>DmelGR39b
MLYSFHPYLKYFALLGLVPWSESCAQSKFVQKVYSAILIILNAVHFGISIYFPQSAELFLSLM
VNVIVFVARIVCVTVIILQVMVHYDDYFRFCREMKYLGLRLQCELKIHVGRLKWQSYAKI
LALGIGFLVTVLPSIYVALSGSLLYFWSSLLSILIIRMQFVLVLLNVELLGHHVSLLGIRLQN
VLECHLMGANCTLDGNANRLCSLEFLLALKQSHMQLHYLFTHFNDLFGWSILGTYVVLF
SDSTVNIYWTQQVLVEVYEYKYLYATFSVFVPSFFNILVFCRCGEFCQRQSVLIGSYLRNLS

CHPSIGRETSYKDLLMEFILQVEQNVLAINAEGFMSTDNSLLMSILAAKVTYLIVLMQFSS
V
>DmelGR43a
MEISQPSIGIFYISKVLALAPYATVRNSKGRVEIGRSWLFTVYSATLTVVMVFLTYRGLLFD
ANSEIPVRMKSATSKVVTALDVSVVVMAIVSGVYCGLFSLNDTLELNDRLNKIDNTLNAY
NNFRRDRWRALGMAAVSLLAISILVGLDVGTWMRIAQDMNIAQSDTELNVHWYIPFYSL
YFILTGLQVNIANTAYGLGRRFGRLNRMLSSSFLAENNATSAIKPQKVSTVKNVSVNRPAM
PSALHASLTKLNGETLPSEAAAKNKGLLLKSLADSHESLGKCVHLLSNSFGIAVLFILVSCL
LHLVATAYFLFLELLSKRDNGYLWVQMLWICFHFLRLLMVVEPCHLAARESRKTIQIVCEI
ERKVHEPILAEAVKKFWQQLLVVDADFSACGLCRVNRTILTSFASAIATYLVILIQFQRTNG
>DmelGR47a
MAFTSSQLCSLLTKFTALNGLNTYYFDTKTNAFRVSSKLKIYCAIHHALCVLALAHMSYST
ASNLRVSVTVLTIGGTMACCVKSCWEKAQGIRNLARGLVTMEQKYFAGRPSGLLLKCRY
YIKITFGSITLLRIHLIQPIYMRRLLPSQFYLNVGAYWLLYNMLLAAVLGFYFLLWEMCRIQ
KLINDQMTLILARSGQRNRLKKMQHCLRLYSKLLLLCDQFNSQLGHVAIWVLACKSWCQ
ITFGYEIFQMVAAPKSIDLTMSMRVFVIFTYIFDAMNLFLGTDISELFSTFRADSQRILRETSR
LDRLLSMFALKLALHPKRVVLLNVFTFDRKLTLTLLAKSTLYTICCLQNDYNKLKA
>DmelGR47b
MQRDDGFVYCYGNLYSLLLYWGLVTIRVRSPDRGGAFSNRWTVCYALFTRSFMVICFMA
TVMTKLRDPEMSAAMFGHLSPLVKAIFTWECLSCSVTYIEYCLSLDLQKDRHLKLVARMQ
EFDRSVLMVFPHVQWNYRRARLKYWYGTVIVGFCFFSFSISLIFDTTRCTCGIPSTLLMAF
TYTLLTSSVGLLGFVHIGIMDFIRVRLRLVQQLLHQLYQADDSSEVHERIAYLFEMSKRCSF
LLAELNGVFGFAAAAGIFYDFTIMTCFVYVICQKLLEREPWDPEYVYMLLHVAIHTYKVV
ITSTYGYLLLREKRNCMHLLSQYSRYFSGQDVARRKTEDFQHWRMHNRQAAMVGSTTL
LSVSTIYLVYNGMANYVIILVQLLFQQQQIKDHQLTSGKDVDIVGPMGPITHMD
>DmelGR57a
MAVLYFFREPETVFDCAAFICILQFLMGCNGFGIRRSTFRISWASRIYSMSVAIAAFCCLFGS
LSVLLAEEDIRERLAKADNLVLSISALELLMSTLVFGVTVISLQVFARRHLGIYQRLAALDA
RLMSDFGANLNYRKMLRKNIAVLGIVTTIYLMAINSAAVQVASGHRALFLLFALCYTIVTG
GPHFTGYVHMTLAEMLGIRFRLLQQLLQPEFLNWRFPQLHVQELRIRQVVSMIQELHYLI
QEINRVYALSLWAAMAHDLAMSTSELYILFGQSVGIGQQNEEENGSCYRMLGYLALVMIP
PLYKLLIAPFYCDRTIYEARRCLRLVEKLDDWFPQKSSLRPLVESLMSWRIQAKIQFTSGLD
VVLSRKVIGLFTSILVNYLLILIQFAMTQKMGEQIEQQKIALQEWIGF
>DmelGR58a
MLLKFMYIYGIGCGLMPAPLKKGQFLLGYKQRWYLIYTACLHGGLLTVLPFTFPHYMYD
DSYMSSNPVLKWTFNLTNITRIMAMFSGVLLMWFRRKRILNLGENLILHCLKCKTLDNRS
KKYSKLRKRVRNVLFQMLLVANLSILLGALILFRIHSVQRISKTAMIVAHITQFIYVVFMMT
GICVILLVLHWQSERLQIALKDLCSFLNHEERNSLTLSENKANRSLGKLAKLFKLFAENQR
LVREVFRTFDLPIALLLLKMFVTNVNLVYHGVQFGNDTIETSSYTRIVGQWVVISHYWSAV
LLMNVVDDVTRRSDLKMGDLLREFSHLELVKRDFHLQLELFSDHLRCHPSTYKVCGLFIF
NKQTSLAYFFYVLVQVLVLVQFDLKNKVEKRN
>DmelGR58b
MLHPKLGRVMNVVYYHSVVFALMSTTLRIRSCRKCLRLEKVSRTYTIYSFFVGIFLFLNLY
FMVPRIMEDGYMKYNIVLQWNFFVMLFLRAIAVVSCYGTLWLKRHKIIQLYKYSLIYWK

RFGHITRAIVDKKELLDLQESLARIMIRKIILLYSAFLCSTVLQYQLLSVINPQIFLAFCARLT
HFLHFLCVKMGFFGVLVLLNHQFLVIHLAINALHGRKARKKWKALRSVAAMHLKTLRLA
RRIFDMFDIANATVFINMFMTAINILYHAVQYSNSSIKSNGWGILFGNGLIVFNFWGTMAL
MEMLDSVVTSCNNTGQQLRQLSDLPKVGPKMQRELDVFTMQLRQNRLVYKICGIVELDK
PACLSYIGSILSNVIILMQFDLRRQRQPINDRQYLIHLMKNKTKV
>DmelGR58c
MNQYFLLHTYFQVSRLIGLCNLHYDSSNHRFILNHVPTVVYCVILNVVYLLVLPFALFVLT
GNIYHCPDAGMFGVVYNVVALTKLLTMLFLMSSVWIQRRRLYKLGNDLMKMLHKFRFN
LGNDCRNRCLCKGLLTSSRFVLLTQQLLTRDSVVNCESNSSLRQAMVPYQSAAIVYALIMI
LLMSYVDMTVYMVEVAGNWLLVNMTQGVREMVQDLEVLPERNGIPREMGLMQILAAW
RKLWRRCRRLDALLKQFVDIFQWQVLFNLLTTYIFSIAVLFRLWIYLEFDKNFHLWKGILY
AIIFLTHHVEIVMQFSIFEINRCKWLGLLEDVGNLWDINYSGRQCIKSSGTILSRKLEFSLLY
MNRKLQLNPKRVRRLHIVGLFDLSNLTVHNMTRSIITNVLVLCQIAYKKYG
>DmelGR59a
MKRIGQAYNVYAVFIGMTSYETMGGKFRQSRITRIYCLLINAIFLTLLPSAFWKSAKLLSTA
DWMPSYMRVTPYIMCTINYAAIAYTLISRCYRDAMLMDLQRIVLEVNREMLRTGKKMNS
LLRRMFFLKTFTLTYSCLSYILAVFIYQWKAQNWSNLCNGLLVNISLTILFVNTFFYFTSLW
HIARGYDFVNQQLNEIVACQSMDLERKSKELRGLWALHRNLSYTARRINKHYGPQMLAM
RFDYFIFSIINACIGTIYSTTDQEPSLEKIFGSLIYWVRSFDFFLNDYICDLVSEYQMQPKFFA
PESSMSNELSSYLIYESSTRLDLLVCGLYRVNKRKWLQMVGSIVVHSSMLFQFHLVMRGG
L
>DmelGR59b
MVYWMIKLYFRYSLAIGITSQQFSNRKFFSTLFSRTYALIANIVTLIMLPIVMWQVQLVFQQ
KKTFPKLILITNNVREAVSFLVILYTVLSRGFRDTAFKEMQPLLLTLFREEKRCGFKGIGGV
RRSLRILLFVKFFTLSWLCVTDVLFLLYSTDALIWVNVLRFFFKCNTNNILEMVPMGYFLA
LWHIARGFDCVNRRLDQIVKSKSTRKHRELQHLWLLHACLTKTALNINKIYAPQMLASRF
DNFVNGVIQAYWGAVFTFDLSTPFFWVVYGSVQYHVRCLDYYLIDNMCDVAVEYHDSA
KHSWSEVRWTKEISSYVIYANSTKLQLWSCGLFQANRSMWFAMISSVLYYILVLLQFHLV
MRK
>DmelGR59c
MVDLVKTILLIAYWYGLAVGVSNFEVDWLTGEAIATRRTTIYAAVHNASLITLLILFNLGNN
SLKSEFISARYLHEYFFMLMTAVRISAVLLSLITRWYQRSRFIRIWNQILALVRDRPQVVRG
RWYRRSIILKFVFCVLSDSLHTISDVSAQRKRITADLIVKLSLLATLTTIFNMIVCQYYLAMV
QVIGLYKILLQDLRCLVRQAECICSIRNRRGGVYSIQCCSLADQLDLIAERHYFLKDRLDE
MSDLFQIQSLSMSLVYFFSTMGSIYFSVCSILYSSTGFGSTYWGLLLIVLSTASFYMDNWLS
VNIGFHIRDQQDELFRVLADRTLFYRELDNRLEAAFENFQLQLASNRHEFYVMGLFKMER
GRLIAMLSSVITHTMVLVQWEIQNDES
>DmelGR59d
MADLLKLCLRIAYAYGRLTGVINFKIDLKTGQALVTRGATLISVSTHLLIFALLLYQTMRKS
VVNVMWKYANSLHEYVFLVIAGFRVVCVFLELVSRWSQRRTFVRLFNSFRRLYQRNPDII
QYCRRSIVSKFFCVTMTETLHIIVTLAMMRNRLSIALALRIWAVLSLTAIINVIITQYYVATA
CVRGRYALLNKDLQAIVTESQSLVPNGGGVFVTKCCYLADRLERIAKSQSDLQELVENLST
AYEGEVVCLVITYYLNMLGTSYLLFSISKYGNFGNNLLVIITLCGIVYFVFYVVDCWINAF
NVFYLLDAHDKMVKLLNKRTLFQPGLDHRLEMVFENFALNLVRNPLKLHMYGLFEFGR

GTSFAVFNSLLTHSLLLIQYDVQNF
>DmelGR59e
MDSSYWENLLLTINRFLGVYPSGRVGVLRWLHTLWSLFLLMYIWTGSIVKCLEFTVEIPTI
EKLLYLMEFPGNMATIAILVYYAVLNRPLAHGAELQIERIITGLKGKAKRLVYKRHGQRTL
HLMATTLVFHGLCVLVDVVNYDFEFWTTWSSNSVYNLPGLMMSLGVLQYAQPVHFLWL
VMDQMRMCLKELKLLQRPPQGSTKLDACYESAFAVLVDAGGGSALMIEEMRYTCNLIEQ
VHSQFLLRFGLYLVLNLLNSLVSICVELYLIFNFFETPLWEESVLLVYRLLWLAMHGGRIWF
ILSVNEQILEQKCNLCQLLNELEVCSSRLQRTINRFLLQLQRSIDQPLEACGIVTLDTRSLGG
FIGVLMAIVIFLIQIGLGNKSLMGVALNRSNWVYV
>DmelGR59f
MRSSATKGAKLKNSPRERLSSFNPQYAERYKELYRTLFWLLLISVLANTAPITILPGCPNRF
YRLVHLSWMILWYGLFVLGSYWEFVLVTTQRVSLDRYLNAIESAIYVVHIFSIMLLTWQC
RNWAPKLMTNIVTSDLNRAYTIDCNRTKRFIRLQLFLVGIFACLAIFFNIWTHKFVVYRSILS
INSYVMPNIISSISFAQYYLLLQGIAWRQRRLTEGLERELTHLHSPRISEVQKIRMHHANLID
FTKAVNRTFQYSILLLFVGCFLNFNLVLFLVYQGIENPSMADFTKWVCMLLWLAMHVGK
VCSILHFNQSIQNEHSTCLTLLSRVSYARKDIQDTITHFIIQMRTNVRQHVVCGVINLDLKFL
TTLLVASADFFIFLLQYDVTYEALSKSVQGNVTRYK
>DmelGR61a
MSRTSDDIRKHLKVRRQKQRAILAMRWRCAQGGLEFEQLDTFYGAIRPYLCVAQFFGIMP
LSNIRSRDPQDVKFKVRSIGLAVTGLFLLLGGMKTLVGANILFTEGLNAKNIVGLVFLIVG
MVNWLNFVGFARSWSHIMLPWSSVDILMLFPPYKRGKRSLRSKVNVLALSVVVLAVGDH
MLYYASGYCSYSMHILQCHTNHSRITFGLYLEKEFSDIMFIMPFNIFSMCYGFWLNGAFTF
LWNFMDIFIVMTSIGLAQRFQQFAARVGALEGRHVPEALWYDIRRDHIRLCELASLVEAS
MSNIVFVSCANNVYVICNQALAIFTKLRHPINYVYFWYSLIFLLARTSLVFMTASKIHDASL
LPLRSLYLVPSDGWTQEVQRFADQLTSEFVGLSGYRLFCLTRKSLFGMLATLVTYELMLLQ
IDAKSHKGLRCA
>DmelGR63a
MRPSGEKVVKGHGQGNSGHSLSGMANYYRRKKGDAVFLNAKPLNSANAQAYLYGVRK
YSIGLAERLDADYEAPPLDRKKSSDSTASNNPEFKPSVFYRNIDPINWFLRIIGVLPIVRHGP
ARAKFEMNSASFIYSVVFFVLLACYVGYVANNRIHIVRSLSGPFEEAVIAYLFLVNILPIMIIP
ILWYEARKIAKLFNDWDDFEVLYYQISGHSLPLKLRQKAVYIAIVLPILSVLSVVITHVTMS
DLNINQVVPYCILDNLTAMLGAWWFLICEAMSITAHLLAERFQKALKHIGPAAMVADYRV
LWLRLSKLTRDTGNALCYTFVFMSLYLFFIITLSIYGLMSQLSEGFGIKDIGLTITALWNIGL
LFYICDEAHYASVNVRTNFQKKLLMVELNWMNSDAQTEINMFLRATEMNPSTINCGGFF
DVNRTLFKGLLTTMVTYLVVLLQFQISIPTDKGDSEGANNITVVDFVMDSLDNDMSLMGA
STLSTTTVGTTLPPPIMKLKGRKG
>DmelGR64a
MKGPNLNFRKTPSKDNGVKQVESLARPETPPPKFVEDSNLEFNVLASEKLPNYTNLDLFH
RAVFPFMFLAQCVAIMPLVGIRESNPRRVRFAYKSIPMFVTLIFMIATSILFLSMFTHLLKIGI
TAKNFVGLVFFGCVLSAYVVFIRLAKKWPAVVRIWTRTEIPFTKPPYEIPKRNLSRRVQLAA
LAIIGLSLGEHALYQVSAILSYTRRIQMCANITTVPSFNNYMQTNYDYVFQLLPYSPIIAVLI
LLINGACTFVWNYMDLFIMMISKGLSYRFEQITTRIRKLEHEEVCESVFIQIREHYVKMCE
LLEFVDSAMSSLILLSCVNNLYFVCYQLLNVFNKLRWPINYIYFWYSLLYLIGRTAFVFLTA
ADINEESKRGLGVLRRVSSRSWCVEVERLIFQMTTQTVALSGKKFYFLTRRLLFGMAGTIV

TYELVLLQFDEPNRRKGLQPLCA
>DmelGR64b
MPQGETFHRAVSNVLFISQIYGLLPVSNVRALDVADIRFRWCSPRILYSLLIGILNLSEFGAVI
NYVIKVTINFHTSSTLSLYIVCLLEHLFFWRLAIQWPRIMRTWHGVEQLFLRVPYRFYGEY
RIKRRIYIVFTIVMSSALVEHCLLLGNSFHLSNMERTQCKINVTYFESIYKWERPHLYMILP
YHFWMLPILEWVNQTIAYPRSFTDCFIMCIGIGLAARFHQLYRRIAAVHRKVMPAVFWTEV
REHYLALKRLVHLLDAAIAPLVLLAFGNNMSFICFQLFNSFKNIGVDFLVMLAFWYSLGFA
VVRTLLTIFVASSINDYERKIVTALRDVPSRAWSIEVQRFSEQLGNDTTALSGSGFFYLTRSL
VLAMGTTIITYELMISDVINQGSIRQKTQYCREY
>DmelGR64c
MQQSGQKGTRNTLQHAIGPVLVIAQFFGVLPVAGVWPSCRPERVRFRWISLSLLAALILFV
FSIVDCALSSKVVFDHGLKIYTIGSLSFSVICIFCFGVFLLLSRRWPYIIRRTAECEQIFLEPEY
DCSYGRGYSSRLRLWGVCMLVAALCEHSTYVGSALYNNHLAIVECKLDANFWQNYFQR
ERQQLFLIMHFTAWWIPFIEWTTLSMTFVWNFVDIFLILICRGMQMRFQQMHWRIRQHVR
QQMPNEFWQRIRCDLLDLSDLLGIYDKELSGLIVLSCAHNMYFVCVQIYHSFQSKGNYAD
ELYFWFCLSYVIIRVLNMMFAASSIPQEAKEISYTLYEIPTEFWCVELRRLNEIFLSDHFALS
GKGYFLLTRRLIFAMAATLMVYELVLINQMAGSEVQKSFCEGGVGSSKSIFS
>DmelGR64d
MLRSHLSVHGLQMERSVQENTLHYTIGHVLIIARIFGVLPLAGINPNGKPENVRFRWFSPYI
LFFVVAFTFVIADFMLSTKIVLNDGLQLYTMGSLSFSVICIFCFGSFIKLSRRWPHIIRETALC
ERIFLKPCYANQEGLNFTRFLRRWALILLVAALCEHLTYVGSAAWSNYVQIRDCNLKVGFV
ENYFLRERQELFSVFEYRAWMVFFIEWNTMAMTFVWNFGDIFLFLMCRGLKIRFQQLHW
RIRQNLGKPMAKEFWQEIRSDFLDLDSLLKLYDKELSGLILVCCAHNMYFICVQVYHSFQ
VKGAFMDELYFWFCLLYVISRLMNMMLAASSIPQEIKDISNTLYEVRSSPWCDELGRLSE
MLRNETFALSGMGYFYVTRRLIFAMAGALMGYELVLFRQMQGAVVQKSICSRGPGSSMSI
FFS
>DmelGR64e
MARTTGDPAKRRRCMSRIKFWRRSRVGSEATLGIIKYRVVEKDTKRFKLSLIKAWLLRIRQ
EDYKYSGSFQEAIKPVLIIAQIFALMPVRKVSSKFAEDLTFTWFSVRSYYALVTILFFGVSSG
YMVAFVTSVSFNFDSVETLVFYLSIFLISLSFFQLARKWPEIAQSWQLVEAKLPPLKLPKER
RSLAQHINMITIVATTCSLVEHIMSMLSMGYYVNSCPRWPDRPIDSFLYLSFSSVFYFVDYT
RFLGIVGKVVNVLSTFAWNFNDIFVMAVSVALAARFRQLNDYMMREARLPTTVDYWMQ
CRINFRNLCKLCEEVDDAISTITLLCFSNNLYFICGKILKSMQAKPSIWHALYFWFSLVYLL
GRTLILSLYSSSINDESKRPLVIFRLVPREYWCDELKRFSEEVQMDNVALTGMKFFRLTRGV
VISVAGTIVTYELILLQFNGEEKVPGCFEN
>DmelGR64f
MKILPKLERKLRRLKKRVTRTSLFRKLDLVHERARKKAFQESCETYKNQIENEYEIRNSLP
KLSRSDKEAFLSDGSFHQAVGRVLLVAEFFAMMPVKGVTGKHPSDLSFSWRNIRTCFSLLF
IASSLANFGLSLFKVLNNPISFNSIKPIIFRGSVLLVLIVALNLARQWPQLMMYWHTVEKDL
PQYKTQLTKWKMGHTISMVMLLGMMLSFAEHILSMVSAINYASFCNRTADPIQNYFLRTN
DEIFFVTSYSTTLALWGKFQNVFSTFIWNYMDLFVMIVSIGLASKFRQLNDDLRNFKGMN
MAPSYWSERRIQYRNICILCDKMDDAISLITMVSFSNNLYFICVQLLRSLNTMPSVAHAVY
FYFSLIFLIGRTLAVSLYSSSVHDESRLTLRYLRCVPKESWCPEVKRFTEEVISDEVALTGMK
FFHLTRKLVLSVAGTIVTYELVLIQFHEDNDLWDCDQSYYS

>DmelGR66a
MDNMAQAEDAVQPLLQQFQQLFFISKIAGILPQDLEKFRSRNLLEKSRNGMIYMLSTLILY
VVLYNILIYSFGEEDRSLKASQSTLTFVIGLFLTYIGLIMMVSDQLTALRNQGRIGELYERIRL
VDERLYKEGCVMDNSTIGRRIRIMLIMTVIFELSILVSTYVKLVDYSQWMSLLWIVSAIPTFI
NTLDKIWFAVSLYALKERFEAINATLEELVDTHEKHKLWLRGNQEVPPPLDSSQPPQYDSN
LEYLYKELGGMDIGSIGKSSVSGSGKNKVAPVAHSMNSFGEAIDAASRKPPPPPLATNMVH
ESELGNAAKVEEKLNNLCQVHDEICEIGKALNELWSYPILSLMAYGFLIFTAQLYFLYCAT
QYQSIPSLFRSAKNPFITVIVLSYTSGKCVYLIYLSWKTSQASKRTGISLHKCGVVADDNLL
YEIVNHLSLKLLNHSVDFSACGFFTLDMETLYGVSGGITSYLIILIQFNLAAQQAKEAIQTF
NSLNDTAGLVGAATDMDNISSTLRDFVTTTMTPAV
>DmelGR68a
MKIYQDIYPISKPSQIFAILPFYSGDVDDGFRFGGLGRWYGRLVALIILIGSLTLGEDVLFASK
EYRLVASAQGDTEEINRTIETLLCIISYTMVVLSSVQNASRHFRTLHDIAKIDEYLLANGFRE
TYSCRNLTILVTSAAGGVLAVAFYYIHYRSGIGAKRQIILLLIYFLQLLYSTLLALYLRTLMM
NLAQRIGFLNQKLDTFNLQDCGHMENWRELSNLIEVLCKFRYITENINCVAGVSLLFYFGF
SFYTVTNQSYLAFATLTAGSLSSKTEVADTIGLSCIWVLAETITMIVICSACDGLASEVNGTA
QILARIYGKSKQFQNLIDKFLTKSIKQDLQFTAYGFFSIDNSTLFKIFSAVTTYLVILIQFKQL
EDSKVEDISQA
>DmelGR77a
MPLPLGDPLALAVSPQLGYIRITAMPRWLQLPGMSALGILYSLTRVFGLMATANWSPRGIK
RVRQSLYLRIHGCVMLIFVGCFSPFAFWCIFQRMAFLRQNRILLMIGFNRYVLLLVCAFMT
LWIHCFKQAEIIGCLNRLLKCRRRLRRLMHTRKLKDSMDCLATKGHLLEVVVLLSSYLLS
MAQPIQILKDDPEVRRNFMYACSLVFVSVCQAILQLSLGMYTMAILFLGHLVRHSNLLLA
KILADAEHIFESSQKAGFWPNRQELYKGQQKWLALELWRLLHVHHQLLKLHRSICSLCAV
QAVCFLGFVPLECTIHLFFTYFMKYSKFILRKYGRSFPLNYFAIAFLVGLFTNLLLVILPTYY
SERRFNCTREIIKGGGLAFPSRITVKQLRHTMHFYGLYLKNVEHVFAVSACGLFKLNNAIL
FCIVGAILEYLMILIQFDKVLNK
>DmelGR85a
MYSLIEAQLLGGKLVNRVMASLRRIIQRSLGYFCALNGILDFNTDIGTGNLRRYRVLFMYR
LLHNFAVISLTLKFLFDFTDHFKYIESSTLITVNFFTYFTLVFFALLSSMGSCYQWQNRILAV
LKELKHQRDLSRHMGYRVPRSKQNSIDYLLFALTVLLILRLSIHLATFTLSARMGFNHPCN
CFLPECMIFSMNYLLFAILAEITRCWWSLQSGLKMVLLNRQLSTVAFNLWEIERLHTRFQC
LIDLTSEVCSIFRYVTLAYMARNLWSGIVAGYLLVRFVIGNGLQDVELVYLVFSFITCIQPL
MLSLLVNSMTSTTGSLVEVTRDILKISHKKSVNLERSIEWLSLQLTWQHTHVTIFGVFRINR
SLAFRSASLILVHVLYMVQSDYISITN
>DmelGR89a
MLRFPHVCGLCLLLKYWQILALAPFRTSEPMVARCQRWMTLIAVFRWLLLTSMAPFVLW
KSAAMYEATNVRHSMVFKTIALATMTGDVCISLALLGNHLWNRRELANLVNDLARLHRR
RRLSWWSTLFLWLKLLLSLYDLLCSVPFLKGAGGRLPWSQLVAYGVQLYFQHVASVYGN
GIFGGILLMLECYNQLEREEPTNLARLLQKEYSWLRLIQRFVKLFQLGIFLLVLGSFVNIMV
NIYAFMSYYVSLHGVPLTISNNCLVLAIQLYAVILAAHLCQVRSAKLRKKCLQLEYVPEGLT
QEQAMASTPFPVLTPTGNVKFRILGVFILDNSFWLFLVSYAMNFIVVILQTSFEHINHGEI
>DmelGR92a
MFEFLHQMSAPKLSTSILRYIFRYAQFIGVIFFCLHTRKDDKTVFIRNWLKWLNVTHRIITF

TRFFWVYIASISIKTNRVLQVLHGMRLVLSIPNVAVILCYHIFRGPEIIDLINQFLRLFRQVSD
LFKTKTPGFGGRRELILILLNLISFAHEQTYLWFTIRKGFSWRFLIDWWCDFYLVSATNIFIH
INSIGYLSLGVLYSELNKYVYTNLRIQLQKLNTSGSKQKIRRVQNRLEKCISLYREIYHTSIM
FHKLFVPLLFLALIYKVLLIALIGFNVAVEFYLNSFIFWILLGKHVLDLFLVTVSVEGAVNQF
LNIGMQFGNVGDLSKFQTTLDTLFLHLRLGHFRVSILGLFDVTQMQYLQFLSALLSGLAFI
AQYRMQVGNG
>DmelGR93a
MFSSSSAMTGKRAESWSRLLLLWLYRCARGLLVLSSSLDRDKLQLKATKQGSRNRFLHIL
WRCIVVMIYAGLWPMLTSAVIGKRLESYADVLALAQSMSVSILAVISFVIQARGENQFREV
LNRYLALYQRICLTTRLRHLFPTKFVVFFLLKLFFTLCGCFHEIIPLFENSHFDDISQMVGTG
FGIYMWLGTLCVLDACFLGFLVSGILYEHMANNIIAMLKRMEPIESQDERYRMTKYRRMQ
LLCDFADELDECAAIYSELYHVTNSFRRILQWQILFYIYLNFINICLMLYQYILHFLNDDEV
VFVSIVMAFVKLANLVLLMMCADYTVRQSEVPKKLPLDIVCSDMDERWDKSVETFLGQL
QTQRLEIKVLGFFHLNNEFILLILSAIISYLFILIQFGITGGFEASEDIKNRFD
>DmelGR93b
MVYGFTMSGLLVMPRILRCLNVSRISAILLRSCFLYGTFFGVITFRIERKDSQLVAINRRGYL
WICLVIRLLASCFYGYSYDAWSGQYEDMYLRAFFGFRLIGCLICSVIILVMQFWFGEELINL
VNRFLQLFRRMQSLTNSPKNRFGDRAEFLLMFSKVFSLLFVFMAFRLMLSPWFLLTLVCD
LYTSVGTGMITHLCFVGYLSIGVLYRDLNNYVDCQLRAQLRSLNGENNSFRNNPQPTRQA
ISNLDKCLYLYDEIHQVSRSFQQLFDLPLFLSLAQSLLAMSMVSYHAILRRQYSFNLWGLVI
KLLIDVVLLTMSVHSAVNGSRLIRRLSFENFYVTDSQSYHQKLELFLGRLQHQELRVFPLG
LFEVSNELTLFFLSAMVTYLVFLVQYGMQSQQI
>DmelGR93c
MIERLKKVSLPALSAFILFCSCHYGRILGVICFDIGQRTSDDSLVVRNRHQFKWFCLSCRLIS
VTAVCCFCAPYVADIEDPYERLLQCFRLSASLICGICIIVVQVCYEKELLRMIISFLRLFRRVR
RLSSLKRIGFGGKREFFLLLFKFICLVYELYSEICQLWHLPDSLSLFATLCEIFLEIGSLMIIHI
GFVGYLSVAALYSEVNSFARIELRRQLRSLERPVGGPVGRKQLRIVEYRVDECISVYDEIER
VGRTFHRLLELPVLIILLGKIFATTILSYEVIIRPELYARKIGMWGLVVKSFADVILLTLAVHE
AVSSSRMMRRLSLENFPITDHKAWHMKWEMFLSRLNFFEFRVRPLGLFEVSNEVILLFLSS
MITYFTYVVQYGIQTNRL
>DmelGR93d
MKATKYSVGILRFMSFYARFLSLVCFRLRKQKDNNVWLEEIWSNRSRWKWISVTLRIVPL
CIYAFTYAEWISNRMLITEKFLHSCSLVVSIPCYLSIIHLKICHGPEVTKLVNQYLHIFRLGTL
DIRRRSQFGGGRELFLLILSVCCQIHEYVFILVIASRLCGFQHIIWWVSYTYVFIICNSIMCFG
FIWHLSLGVLYAELNDNLRFESGFQTAFLRKQQRIRVQKSMALFKEISSVVTSLQDIFNVHL
FLSALLTLLQVLVVWYKMIIDLGFSDFRIWSFSLKNLIQTLLPVLAIQEAANQFKQTRERAL
DIFLVGKSKHWMKSVEIFVTHLNLSEFRVNLLGLFNVSNELFLIIVSAMFCYLVFVTQCVIV
YRRRYVI
>DmelGR94a
MDFTSDYAHRRMVKFLTIILIGFMTVFGLLANRYRAGRRERFRFSKANLAFASLWAIAFSL
VYGRQIYKEYQEGQINLKDATTLYSYMNITVAVINYVSQMIISDHVAKVLSKVPFFDTLKE
FRLDSRSLYISIVLALVKTVAFPLTIEVAFILQQRRQHPEMSLIWTLYRLFPLIISNFLNNCYFG
AMVVVKEILYALNRRLEAQLQEVNLLQRKDQLKLYTKYYRMQRFCALADELDQLAYRY
RLIYVHSGKYLTPMSLSMILSLICHLLGITVGFYSLYYAIADTLIMGKPYDGLGSLINLVFLSI

SLAEITLLTHLCNHLLVATRRSAVILQEMNLQHADSRYRQAVHGFTLLVTVTKYQIKPLGLY
ELDMRLISNVFSAVASFLLILVQADLSQRFKMQ
>DmelGR97a
MRFLRRQTRRLRSIWQRSLPVRFRRGKLHTQLVTICLYATVFLNILYGVYLGRFSFRRKKF
VFSKGLTIYSLFVATFFALFYIWNIYNEISTGQINLRDTIGIYCYMNVCVCLFNYVTQWEKT
LQIIRFQNSVPLFKVLDSLDISAMIVWRAFIYGLLKIVFCPLITYITLILYHRRSISESQWTSV
TTTKTMLPLIVSNQINNCFFGGLVLANLIFAAVNRKLHGIVKEANMLQSPVQMNLHKPYY
RMRRFCELADLLDELARKYGFTASRSKNYLRFTDWSMVLSMLMNLLGITMGCYNQYLAI
ADHYINEEPFDLFLAIVLVVFLAVPFLELVMVARISNQTLVETRRTGELLQRFDLQHADARF
KQVVNAFWLQVVTINYKLMPLGLLELNTSLVNKVFSSAIGSLLILIQSDLTLRFSLK
>DmelGR98a
MEQMSGELHAASLLYMRRLMKCLGMLPFGQNLFSKGFCYVLLFVSLGFSSYWRFSFDYE
FDYDFLNDRFSSTIDLSNFVALVLGHAIIVLELLWGNCSKDVDRQLQAIHSQIKLQLGTSNS
TDRVRRYCNWIYGSLIIRWLIFIVVTIYSNRALTINATYSELVFLARFSEFTLYCAVILFIYQEL
IVGGSNVLDELYRTRYEMWSIRRLSLQKLAKLQAIHNSLWQAIRCLECYFQLSLITLLMKF
FIDTSALPYWLYLSRVEHTRVAVQHYVATVECIKLLEIVVPCYLCTRCDAMQRKFLSMFYT
VTTDRRSSQLNAALRSLNLQLSQEKYKFSAGGMVDINTEMLGKFFFGMISYIVICIQFSINF
RAKKMSNEQMSQNITSTSAPI
>DmelGR98b
MVAQKSRLLARAFPYLDIFSVFALTPPPQSFGHTPHRRLRWYLMTGYVFYATAILATVFIVS
YFNIIAIDEEVLEYNVSDFTRVMGNIQKSLYSIMAIANHLNMLINYRRLGGIYKDIADLEMD
MDEASQCFGGQRQRFSFRFRMALCVGVWMILMVGSMPRLTMTAMGPFVSTLLKILTEFV
MIMQQLKSLEYCVFVLIIYELVLRLRRTLSQLQEEFQDCEQQDMLQALCVALKRNQLLLG
RIWRLEGDVGSYFTPTMLLLFLYNGLTILHMVNWAYINKFLYDSCCQYERFLVCSTLLVNL
LLPCLLSQRCINAYNCFPRILHKIRCTSADPNFAMLTRGLREYSLQMEHLKLRFTCGGLFDI
NLKYFGGLLVTIFGYIIILIQFKVQAIAANRYKKVVN
>DmelGR98c
MEMEAKRSRLLTTARPYLQVLSLFGLTPPAEFFTRTLRKRRRFCWMAGYSLYLIAILLMVF
YEFHANIVSLHLEIYKFHVEDFSKVMGRTQKFLIVAIATCNQLNILLNYGRLGLIYDEIANL
DLGIDKSSKNFCGKSHWWSFRLRLTLSIGLWMVIIIGVIPRLTLGRAGPFFHWVNQVLTQII
LIMLQLKGPEYCLFVLLVYELILRTRHVLEQLKDDLEDFDCGARIQELCVTLKQNQLLIGRI
WRLVDEIGAYFRWSMTLLFLYNGLTILHVVNWAIIRSIDPNDCCQLNRLGSITFLSFNLLLT
CFFSECCVKTYNSISYILHQIGCLPTAEEFQMLKMGLKEYILQMQHLKLLFTCGGLFDINIK
LFGGMLVTLCGYVIIIVQFKIQDFALIGYRQNTSDTS
>DmelGR98d
MEANRSRLLAAARPYIQIYSIFGLTPPIQFFTRTLHKRRRGIVILGYACYLISISLMVIYECYA
NIVALQKDIHKFHAEDSSKVMGNTQKVLVVAMFVWNQLNILLNFRRLARIYDDIADLEID
LNNASSGFVGQRHWWRFRFRLALSVGLWIVLLVGLTPRFTLVALGPYLHWTNKVLTEIILI
MLQLKCTEYCVFVLLIYELILRGRHILQQISVELEGNQSRDSVQELCVALKRNQLLAGRIW
GLVNEVSLYFTLSLTLLFLYNELTILQIVNWALIKSVNPNECCQYRRVGTCLLLSINIFLSCLY
SEFCIQTYNSISRVLHQMYCLSAAEDYLILKMGLREYSLQMEHLKLIFTCGGLFDINLKFFG
GMVVTLFGYIIILVQFKIQFFAQSNFMQNINSTELKAYTA
Ionotropic receptor:
>AgifIR1

MFQHSNCR
LFCFYYLLVGVELVFSFNDFPSLVNANASMAVVIEKSFYERKILIKEIFIHSKDAYEKSVASF
TNVVDKIAREKMNISGLSIHVFQDIQTNLARDYMILLSVSTCQTAWNLFKRAKIEKLVHLA
ITDINCPRLPAKEGMSIPLVDPGEELPQIFYDLRVSGAFHWSKATFLHDDSIERETIGKIIQAF
DDELPILARGLSTNSLFFFERGKSDTVTNQNIQNILVKFSTGMFLSNQFIVIVAHDTLSHVL
QTARSLKMLNPSTQWLFIVPNMVEFPYGSRRFPIELLGEGENIAILYNETYKNYQNDIKYN
ENISIRALCFVQEMMGSLAIAIEKVSSIETHLYGQVTEEQFEATGFSKLDRSQILLEYLSDQF
YNKTQDSSGSSCKVDCDMNWILQSGLTWGNTIGSSIDTQPHKLIVTGIWTPDRGFVSMDH
MFPHIYYGFRRKSLPIATYNNSPWHYQKLSPNLKEKNVNDLRGKWDGLIFDVINELSNKL
NFTFKTVSGGNEPQMISTKNDSLLKCSMSAAEKVPSDVIELVRTKSVFIGACAITSVLYEKE
KNVNFTNAISTQTYGLLTGIPQPQSRALLFTSPYSTQAWICIVISIIVVGPVLWAVHFYSPRR
LRSVDDENSKNPGSASDYMWYIYGALLQQGGMHLPKSNGARLVVATWWLVVMVVVAT
YSGSLVAFLTFPKMEPVVKTIDDLLERREELTWSIPKDSLLDDYIKEYDAHGQKHDTVSYL
LSINKIKKGKHVVIDWLSSLRISIKNNYRSSGHCDFSLGTDVLLLQEPIFMLVPAGSPYLDII
NIQLQRMLEAGLTNKWINDRMPTKDQCWLNAKNSNSAAANRKVNLQDMQGIFFVLFFG
YITAVLFLCFEHYIYSRKISKERLLILPFVL
>AgifIR2
MYLIFIILLNIFIVGLSSVIDDTTGKFIKSLTSSIHSSASITAFFCIKTDKAVEVTKIFSQYSLIAD
IHTSFDNYNFDNLNLQIAHQHYYVIDFDCNDAVSIIKLANMKRMFVAPFKWLIINDQNMSI
TNDIEDDYYEKNKSSLFSVFKNINIYPDSEVFIGQREIINNTFKLLSLYRPSFNHDLIIENRGS
WNYRNGLVLTNHDSSSRRRRDLKKTPLKSCLVMTNPDTLNHLTDYKNKLVDAVTKANYP
WIIHLVNRINATVNFTVRDTWGYKDKNGLWNGMIGLLDSGEIDIGGTGTFLIPERIGVVDY
VQLYTPTGSKFLFRRPPLSYVSNLFTLPFSRSVWLAIVIFLFLVCCLLFITMKWEFNLSIKNN
NPMSYYKTIKPTISDNFLVILGAVSQQGFSYEATIISTRIIIFMVLLATLSLYAAYTANIVALLQ
STTDSIKTLTDLMNSPLKIGIYDIVYSRYILSTIEEPVRKEFYEKMVKGNEHVWMSLTEGIQ
KLRQGLFAFHVEVGAGYQLIQETFEEDEKCGINEIDIFNLLNPLLVIKRQSPYRELIRVGAM
WIHETGLQKRDIRRLYTEKPTCVGGTSFVSVGITECYAAISTLFYGAALTFIIFIIEIIWKKWF
DKSEINVEENDMNNIEIDEENISQLDVDYLE
>AgifIR3
MLNLKKESWISDNDFKAIIKLSFPFSLCCNAYVKGKTLGVDVLFNQFIKNFKYEYTVQSLS
YDCHAYFGLASSENDMMNKVKERVPTSISTTELLLIIDDKITSNSSILDVSIYKNSNVNIISR
SGYWTLSENYLFPRVFKKVKRLKELRHKRGIVDLQGRQLQVAAFYNPPFCYLSTTINQTIN
KIDGEFFLANDDRELDGIETKLFLLMSKRLNFTWKIRKPNHYFRYGRPNGSNWEGGMIGQ
LFRNEIDFAFSGIWLKHDQYQYVNLTEPWYQLLIHYLVPRPRPQTSIWALTRPFTIEVWLLL
IVVIIIQSINITIKARINQRVPKKFKSFILTITELLNIGSWSPLGTYGLRLQIHLWHVYGILIVTA
YSSSLASRLTTPDYEPRIDTIQQFIDKNLTWGREAPVPNFDDYFNLRDPYAGKFPDKFTIDID
EEDRHVKIKKGNYAIIGRIVQGIFFPENHIRDADLKNYRVMKQEVGDYYATFAVQPWLLHS
INKMVLWLRECGFTKYHLTNVIHRRTSLSLRNVLEEHDIKNNEGARVLILKPLIAGFGFFLI
GLFISTLVLIYELRKKYPSISIFKTIKIMLNKKKFKKQKQYKHKSNIITTKI
>AgifIR4
MFQHSNCRLFCFYYLLVGVELVFSFNDFPSLVNANASMAVVIEKSFYERKILIKEIFIHSKD
AYEKSVASFTNVVDKIAREKMNISGLSIHVFQDIQTNLARDYMILLSVSTCQTAWNLFKRA
KIEKLVHLAITDINCPRLPAKEGMSIPLVDPGEELPQIFYDLRVSGAFHWSKATFLHDDSIER
ETIGKIIQAFDDELPILARGLSTNSLFFFERGKSDTVTNQNIQNILVKFSTGMFLSNQFIVIVA

HDTLSHVLQTARSLKMLNPSTQWLFIVPNMVEFPYGSRRFPIELLGEGENIAILYNETYKN
YQNDIKYNENISIRALCFVQEMMGSLAIAIEKVSSIETHLYGQVTEEQFEATGFSKLDRSQI
LLEYLSVITII
>AgifIR5
MIAIRVDREN
SKESFQKVCSVVFNGVTIILDTTYTGWDKIRSYALQNGIIYVRSSGSIIPYVNAVDDLLLKK
NATDVALIFGNEEELNHSLYYLIGNSIIRLVVIDELSDLTITKIRTMRPSPSYYVIYASTDKMEI
LFRTAIHGGLVKRDAIWNLVFTDMDYKKFSLIAGKNDLNVTVGILSMSSEYCCRLVTETY
CTCPSDIQIGAKYLQKLMFLLMSTVIQIQESGINLEPIKANCQKNGVLASADNNITVNAFIS
ALRDKIEDYNGIFLYREDQYLISYRAIIDIELLDGKNLEPLANWTKRNGIVEAAGKEIKPSK
RYFRVGTAEALPWSTIKKNPETGEPMKDSEGNIIWEGYCIDFIETLSIKMNFDYDLIIPADKT
FGEKLSNGKWTGLIGDLARGETDIAIGALTMTSEREEVIDFVAPYFEQSGILIVMRKPVRKT
SLFKFMTVLRVEVWLSIVGALTLTAIMIWLLDKYSPYSAKNNKKNYPYPCREFTLKESFWF
ALTSFTPQGGGEAPKALSSRTLVAAYWLFVVLMLATFTANLAAFLTVERLQAQVQSLEQL
ARQSRINYTVVAKSPTHQYFINMKNAEEKLYNVWKEITLNSTSDQVEYRVWDYPIKEQYG
HILQSITAVGPVRNVAEGFQKVIASEEAEFAFIHDSSEIKYEVSRNCNLTEIGETFAEQPYAVA
VQQGSHLQEEISRK
>AgifIR6
MIMTGIDLMLMGICIISNTTNCYRNHSLLINDKRHDWEFDLDVIDLSTTAQNLKSKDENSL
PKVMKITTLDMSPYSSIYELNGKTMGNVTSYAFYIFDLISKKFNITYEIVMPDQKILGDHK
KGVIGLLHERKVDMAVAFLPINPDMHPYVSYSPMLTQIELKAMMRRPDNSAIGSGLLAPF
TKQVWILIVIAVIISGPIFFGIIFLRSCLWKNSKTDNYTFLQCAWFTYGAILKQGSTIIPKTDS
NRVLFASWWIFITILTSFYTANLTAFLTLAEFTLPYKSIDDIVNDKRMWSSTSEHYIDYALKQ
SKIEEFNILKTSFKNNYGRFINKPSDKEILEIMYLKKKIYLGEGNHFQRMIVNDYLENVRK
KEPQNRRCKYVIMPEIIYEQPVAFAYPKDSLYQEKFDKQLRNLVETGIFKHLEQMKLPAMH
YCPLKLNSIERKLKMNDLSLTYKIIAGGFTLATLLLIYEKTGLHLLQIYKSRHNKRKPVLTRI
PTQTLPFVYPVNNYFDNYPNTGNPSPIYQSISMNKRIIFNGRDYLEVDLPLGGKRLIPTRSP
SALLFQYYK
>AgifIR7
MIKKFFMLWTIMEYLFTRSESLVINDHYKKLIIDVTTTTHAASSFTAFLCSSPDEAVQLSKD
MTNNYKIHNLQNNFKNYEFSVDNKQIAHQLFYIVDFQCEDSVNLLIKANLSQMFLAPRK
WLILQDLTNSTSKLSQNKLINLFNDFDAYADSEVYIAQGYQSKINNSIELLSLYRPSPYRNIV
IENRGIWDEQNGLRLNNHDPSSRRRRDLQQTPLKSCLVVTNPDTLNHLTDAKDKFVDTIT
KVNYPWILHIVNRINATINFTVRDTWGYQDDNGTWSGMIGLLDRGEIDIGGTATFLVSSRI
GVVDYVQLYTPTKSKFIFRRPPLSYVRNLFTLPFSRSVWMTIIIFLSLVCLILYVTMKWETK
HICKNHSAVVQYSVDWNPNPAISDNFLVILGAVSQQGFFYEAKMISTRVVVLVLLIAALSLY
AAYSANIVANIQRTSDSIQTISDLMDSPLKIGIHDIVYNRYYFKTLDDPVRKKFYDTKVKDK
SNIWMNVTDGIENVRKGLFAFQVETSAGYQVMQDTYAEDEKCGLHEIDILNILNPLLVIKR
QSPYRELIRVSALWIHETGLQKRDLHRLYTSKPSCVGQTAFISVGLTECYPAILTIAYGALLT
FGVFILELIWHYCNDNYLKNTIPDFDRNSEKSLDEKTPSIVDFDGTSLE
>AgifIR8
MKITTLDMSPYSSIYELNGKTMGNVTSYAFYIFDLISKKFNITYEIVMPDQKILGDHKKGVI
GLLHERKVDMAVAFLPINPDMHPYVSYSPMLTQIELKAMMRRPDNSAIGSGLLAPFTKQV
WILIVIAVIISGPIFFGIIFLRSCLWKNSKTDNYTFLQCAWFTYGAILKQGSTIIPKTDSNRVLF

ASWWIFITILTSFYTANLTAFLTLAEFTLPYKSIDDIVNDKRMWSSTSEHYIDYALKQSKIEE
FNILKTSFKNNYGRFINKPSDKEILEIMYLKKKIYLGEGNHFQRMIVNDYLENVRKKEPQN
RRCKYVIMPEIIYEQPVAFAYPKDSLYQEKFDKQLRNLVETGIFKHLEQMKLPAMHYCPLK
LNSIERKLKMNDLSLTYKIIAGGFTLATLLLIYEKTGLHLLQIYKSRHNKRKPVLTRIPTQTL
PFVYPVNNYFDNYPNTGNPSPIYQSISMNKRIIFNGRDYLEVDLPLGGKRLIPTRSPSALLF
QYYR
>AgifIR9
MIAIRVDRENSKESFQKVCSVVFNGVTIILDTTYTGWDKIRSYALQNGIIYVRSSGSIIPYVN
AVDDLLLKKNATDVALIFGNEEELNHSLYYLIGNSIIRLVVIDELSDLTITKIRTMRPSPSYYV
IYASTDKMEILFRTAIHGGLVKRDAIWNLVFTDMDYKKFSLIAGKNDLNVTVGILSMSSEY
CCRLVTETYCTCPSDIQIGAKYLQKLMFLLMSTVIQIQESGINLEPIKANCQKNGVLASAD
NNITVNAFISALRDKIEDYNGIFLYREDQYLISYRAIIDIELLDGKNLEPLANWTKRNGIVEA
AGKEIKPSKRYFRVGTAEALPWSTIKKNPETGEPMKDSEGNIIWEGYCIDFIETLSIKMNFD
YDLIIPADKTFGEKLSNGKWTGLIGDLARGETDIAIGALTMTSEREEVIDFVAPYFEQSGILI
VMRKPVRKTSLFKFMTVLRVEVWLSIVGALTLTAIMIWLLDKYSPYSAKNNKKNYPYPCR
EFTLKESFWFALTSFTPQGGGEAPKALSSRTLVAAYWLFVVLMLATFTANLAAFLTVERLQ
AQVQSLEQLARQSRINYTVVAKSPTHQYFINMKNAEEKLYNVWKEITLNSTSDQVEYRV
WDYPIKEQYGHILQSITAVGPVRNVAEGFQKVIASEEAEFAFIHDSSEIKYEVSRNCNLTEIG
ETFAEQPYAVAVQQGSHLQEEISRNILELQRNRYFESLTAKYWNSSLKGKCAVADENEGITL
ESLGGVFIATLFGLALAMITLAGEVIYYRRKNTVSGKEQKQESGGRLRSGNSKFIQRITQK
GGKIQIKSSPAISLGLSRRRGSKTRISHISVHPKNFPFND
>AgifIR10
MRINLLLLFLSIVNLCHGDAIVQKFTKNFFGEFNVHHVVIFACWDEIDSFKYVRSIMELGT
VVSYIKITPTLQNKIEDILQVNYYHIGVFLDFDCPFSNIVLEKFSNQLVFNESYIWLLHTKSS
LPMGESLRNLPLTVESELTIAIRDQDTFKMYDVYNPSYRHGGSLNVTYRGQWNSSIGLEN
KLTQYKYSRRANFNLLTLNFSCALINPPLPNLQTYMSSRDNPHFDTMTRYHFALGQQLQD
MFNFTINLNVAKTWGYLVNGTFNGIIGDMLAGKVDISISPFQYKAERLSVCEFTVETWVV
KPNFIFRHPIGNSLENHFLQPFTYKLWYAILVVGFIYWLLLLASLKVEIYYGNSNESSGSLV
TTPASETGLITMAAISQQGLSDGPSIISGRIIFLSLFIWGLLLYQFYSASIVGSLLAARPRWLN
SMDNLTDSSLECALEDVAYHRDYFATSTNPIDSRFWNAKIKPTKKRPNGGYYSAIEGMQK
VKNGGFAFHIDPATAYKVIEDTFEEDEICELHEVEMLPPRKVTLVTSKHSPFKKMIIYGLRKI
VETGIAHRLRQIWHHQKPKCPESYSSKPSPVPIEQFNPAIFLLIIGIIIAFFIMLLENIFFYYHTS
NLNFNDDHEVSSAD
NQLDDMNDEQIDIDTISG
>AgifIR11
MNQHLVLFILTYVAIHVVGQQRKQQSIPKGPRSINLFVINDENNDVANRSVGNSLKNIRDK
HPEWLGNVLIVKINGAEPHEALEKICSTWDKAVRNGGPNVPDLLLDMTRSGFGAETVNSF
TSAVGIPTLSTQFGQIDDLRHWRDLTDDKKLYLIQVMQPADLIPDAIRQLSIQMNISNAAIIF
DDNFIMDHKYKSLLVNVPTRHVIVKSKNTKKQIDEQLTRIRSLDLVNFFILSNDKVLTSILD
DAEAKNFTNKKYGWYGFSLDDVDPKCDCKNITILFFKPKITTLNQQRISELTTKGSLPKPIL
TSAFYYDLTKLGVLAMRLSTEDGSWPYEPPHITCNEYNGNNTPIRNFDFLKQLNKTMTNN
DFQPTFASFYWGRKNGQHHAKFDMKVSLIVIDNGNTISTDELGTWEAGIDSPLKLSGINNE
KVSNHTAVTSFRIVTVETPPFIMYNKEKDEWSGYCIDLIEEIRKILGFEYEIRLVDDNNFGS
MNDDGEWNGMIKELVDKKADIALGALSVMAERENVVDFTVPYYDLVGLSILMKKPKAE

TSLFKFLTVLENDVWLCILGSYFFTSILMWLFDKMSPYSYQNNKDKYKDDDEKREFTLKE
SLWFCMTSLTPQGGGEAPKNLSGRLVAATWWLFGFIIIASYTANLAAFLTVSRLESPIESLD
DLSKQYKVTYAPIKPSEAYTYFERMSNIESRFYEIWKDMSLNDSLSDIERAKLAVWDYPVS
DKYTKMFQAMADAGFPSTMEEALARILKLNTSSSDSEFALIGDGTDIRYLEMTTCNYIMV
GEEFSRKPYAIAVQQGSPLKDQFNNAILQLLNKRQLEQLKAKWWQNNKKKMSCEQQDD
QSDGISLANIGGVFIVIFVGIGMACVTLGLEYWWYRCYIGKNRRNKSIERKRLP
PPSQIIKPMRFESKKNKLLFKAAPSVRSRF
>AgifIR12
LEQSHALRPANVSCELEHPWDGGLSLINYINSVEMKGLSGPIEFKEGRRIQFKLDLLKLK
QHSLVKVGEWSPSLGVNVTDTAAFFEPGIVNVTLIVVTIPEQPYVMLRNTGNFSGNERYE
GFCIDLLREIAQMVGFTYRIELVPDGKYGVYNYETKEWNGIVRELMEKKADLAVGSMTIN
YARESVIDFTKPFMNLGISILFKVPTSHPARLFSFMNPLAVEIWLYALAAYVLVSVTMFV
VARFSPYEWNNPHPCHTGAEDIVENQFSMSNSFWFTIGTLMQQGSDLNPKAASTRIVSGI
WWFFTLIIISSYTANLAAFLTVERMITPIENAEDLAGQTDIFYGTLDSGSTMTFFRDSMI
ETYKKMWRFMENKKPSVFVPTYEEGIQRVLQGNYAFLMESTMLDYSVQRDCNLTQIGGL
LDTKGYGIATPMGSPWRDKISLAILELQEKGEIQMLYNKWWKLPSDICKRKEKGKENKA
NALGVSSIGGVFVVLLCGLAFAVLVAICEFCYNSRKSSVNEREIPSVPTLPTCSGSLQTISQI
GQHNQHQHNQESLCTEMARELCRTLSCRPSSRKRKGCEKCSTHVVGYTLDNPSVTPINGI
RSQRNSLGADIPAHIHMHHHVPHDYEGN
>AgifIR13
NNLFGHTFRVMSIEDPPQSMISRNIKNEIIGFKGFFGGVIELLQENMNCTIIYQDNDEWGYE
LDNGSWTGMIGSLINNKTDIVAAELMMTAERLDAIKFTTPLYSTKIRAFIKRPSSSPIKWAA
YYAPFSMGPWSILGISILVTGASISFVKYIEAANKINDEPASLTDTILGVFGALCGQGMEASS
LDPIRIIHFVIHMCGLVILAAYSAALISSLAVKTFVMPFTTMKGLLQDGTYRFGVVSGSADY
SFFQNTSDEVLSVLFSEILVKEVDLPSNYLEGLTKVCDEYNYAFMTVDDAVAQLSSSVNCIL
VPLDTISQTSIGMGLRPGSPYRGILDSNL
>AgifIR14
FVTPWFPEKVLTPSSGFLDFAISMRPDYHRAIIDTVRHYGWKKIIYLYDSHDGLLRLQQI
YQDLHPGNESFRVETVKRIQNISDAIDFLHTLEKLSRWSNKYIVLDCPTDMAKDIVVSHV
RDVGLGRRTYHYLLSGLIMDDRWESEVIEYGAINITGYRIVDSSRRTVKEFLEGWRRQDS
VALPGAGRDSISAQAALMYDAVLVLVEAFNKFLKKKIDRNNPKRPGVPGSSQPVNVSRPL
DCNINNGWVTPWEHGDKISKFLRKAEIEGLTGQIRFNN
>AgifIR15
WFFTLIIISSYTANLAAFLTVERMVVPINSPEELASQTEVQYGTLHHGSTWDFFRKSQISLYS
KMWEFMNSRKHVFVNTYDEGIRRVRQSKGKYALLIESPKNEYINEREPCDTMKVGRNLD
AKGFGVATPIGSPLRDKINLAVLSLKESGELTKLVNRWWYDRTECRHGDKQDARNELSLS
NVAGIFYILISGLLLALAVALLEFCYKSHTEATRAKIPLSDAMKAKARLTIGGGRDFD
NGR
>AgifIR16
HASKKACQQVKFGVQAVFGPSDSILGQHIHSICDALDIPHLEVRLDLDIEAKEFSINLHPAQ
ALLNAAYQDVMNFLNWTKVAIIYEDDYGFIKLRDLVRSNQAIDVVLRQADPDSYRQVLT
DIKSKEICNLIVDTKPENMHHFLRMILQLQMNDYKYHYLFTTFDIETFDLEDFKYNFVNIT
AFRLVDADDVGVRGILRDMERFQPAGNTILNKSRVI
>AgifIR17

GIGEATLWCTSIMCMQGSPWNPSTLSGKTVLIASLMFALVTYNAYAGFITSILSVQAAGIRN
LEDLFQNNYKLGYSDVDDEYMRNANDSDLRQLYIKAFNGRESGLSTTLGLQKSVKGGYG
FFVSATLARRALRNSLIHERCSLKEIEIKQTFTIVALPMEKNSPYEKIINLSILRMSER
GVITRI
>AgifIR18
NRRSFRIMFILCGTLGLTGAAPILSYQSNIFKQSGFGLGSDFSIIITGCTIVIAGTTCVSIVKIIG
KRKLLLIAAPIAVLSLAVVATFFTLLEHGVNVHNFNWIPIVFVVIYVFVVGLAFNPIPLAYLG
EIFSFEVKVPAGICSSLYYAVSTTVTVKMYQILADNYGTFFPFWIFTGITFILWILIYLYVPET
EGKSLAEIQIML
>AgifIR19
FLAPRKWLILQDLTNSTSKLSQNKLINLFNDFDAYADSEVYIAQGYQSKINNSIELLSLYRPS
PYRNIVIENRGIWDEQNGLRLNNHDPSSRRRRDLQQTPLKSCLVVTNPDTLNHLTDAKDK
FVDTITKVNYPWILHIVNRINAT
>AgifIR20
DLAEQTEISYGTLEGGSTMTFFRDSKIGIYQKMWRFMDAKRSSVFVSTYEEGVKRVLEGD
YAFLMESTMLDYAVQRDCNLTQIGGLLDSKGYGIATPKGSPWRDKISLAILELQEKGVIQIL
YDKWWKNTGDVCNRD
>AgifIR21
RVGTELDSKGYGIAMPPNSPYRTAISSAILKLQEEGKLHILKTRWWKEKRGGGKCREDNQ
KTGSAANELGLANVGGVFVVLMGGMGVACVMAVCEFVWKSRKVAVEERKASLRSEIPS
ELRY
>AgifIR22
GFQAISAKYVRLPSQEIERNKTYIVTTIVEEPYIMLKKLETGKVLVGNDRFEGYCKDLADLI
AQKLGIKYELKIVDDGKYGSENPDVPGGWDGIAGELIRNEADVAIAPMTITSERERVI
>AgifNmdar1
MDMKNAQARVVLMYASRKDSEVIFKNAGSLNMTGAGYVWIVSEQVLMASNAPEGLLG
LRLINSTDEKAHIKDSLYVLVSALREMNETKTITEAPKDCDNSGSIWETGKELFGFIKKQVL
ERGETGRVAFDDNGDRIFAEYNVVNVRKNREHVSVGQFVYSPDTQRMKLRLNETKIIWP
GHLKTKPEGFMIPTHLKVLTIEEKPFVYTRVSEIAECLPDEIPCPHFNRTEHDVTRFYCCKG
YCIDLLKELSKTINFTYTLALSPDGQFGSYMIKNSSLGGKKEWSGLIGELVDERADMIVAPL
TISPERAEFIEFSKPFKYQGITILEKKPSRSSTLVSFLQPFSNTLWILVMVSVHVVALVLYLLD
RFSPFGRFKLANNDGTEEDALNLSSAIWFAWGVLLNSGIGEGTPRSFSARVLGMVWAGFA
MIIVASYTANLAAFLVLERPKTKLTGINDARLRNTMENLTCATVKGSSVDMYFRRQVELSN
MYRTMEANNYDTAEDAIHDVKIGKLMAFIWDSSRLEFEAAKDCELVTAGELFGRSGYGIG
LQKGSPWADAVTLAILDFHESGFMEILDKNWILQGNLQQCEQFEKTPNTLGLKNMAGVFI
LVGAGIVGGVGLIIIEMAYKKHQIKKQKKLEIARHAVEKWRNLIERRKNHRAIPLRRDQAI
STNDPTTVSLVVDAVARLNQPLKSPAKNPGRAWPGDDDMRRRSSSRHDDNIRLSPAVDVS
HLVV
>MmedIR25a.2
MYNFHWRNILAFILTQCLIQTLQQNQPQNTVRSVNLFIINDEENIIANNSLNNAITNIKDKD
PTVLGNVIVVQINGSDPKPALEKVCAVWDPIVRKGGPGVPDLVIDTTRSYFGTKTVNIFTSS
LGIPKISGRYGQQSDIKHWGNLTADQKNYLIQIMPPVNLIPKAFRQLECEINASNAAIIIDNN
YVRDPAFKSLQQNISTRHVIVQAQINNDDIDKQLNRIHDLDIVNYFVLGRENTLTNYLDVA
DDKNLTGRKYGIASNQQYLRKLTAQNLLPAPWLASTFYYDFVHISVEAMRSAIEGNLWPK

EPRYFTCDEYNGTNTPMRNFDLLSQLRNATTNGIKPTFTRFHWGRSNGEHHAEFNRRVSM
VVIGDGNSIYSDDLGLWNSEIRSPLNLTENFDTGLAHYDSIPIYRIVTVRRPPFIDFNNETND
WEGICIDMLKEMQKFMRFEYKIYESPDDKFGTMDESGNWDGMIKELMLDNADIALGTIS
VTAAREYVIDFTIPFYEPVGYSAITKRLLDRTRLFSFVGSLSVKVWSSAIVGFFTSSILIWVF
DRWSPYSYRNDPNGRFSENYVRKFRLIDSFWFVFSSLSPQGGGIPPKNLSGQMVAGVWW
LFGFITLAAYSANLAAYLTVSRLQPILKSWDDLKEQFDIQYSPVNPSDAYTYFERLKDIEER
FYFVWKDISLNESLTDRERAALAIWEYPVDDEHTRLLGQMNEYGFPSSTEDGINRVLGLPP
YPNDSSYALIAEATTVRYLAMTDCRFLLLEGEFSRKPYAIAVQQGSPLKELFDNAILKLLTE
DKISELKKKWWDNNPKRQPWCPQRINKSDGMDIRQLGGIFIVLWVGIASAIVTLAVQYW
WYRYRSRMRFKKDLENAGSLDNVEPKKFKVKPALRRISRVTNN
>MmedIR64a
MKSRTSVYLLFIIFLALSLSAIEGYDVLAKFTGDYFKKFYVTQIVVFGCWENHISVEFSKSI
MLNTHSKLMYVNINDNLNLDKLLKVDYWSLGIVLDLDCQRSHIIFNQFSEQNLRHNESYF
WLMPTSKEKIPDYFHKLPLNIANEMTLAVRKNNNLQNSGNETNNNNISYILYDVYNPSYR
HGGKLNVTYMGHWSLNDRDEGTLTISLTQYKYKRRGNLYGLVLNASIVVDHPPVPDYNT
YIHNPINPHLDTMHRYNYALTLQLRDYYNFTMNLKRGSTWGYLINGTFNGIIGDMMKGL
VDFGATPFQYKPERLDAIEYTVQAWMARPCFIFRHPTTNELSNPFLKPFEMKVWYVIGIFA
AVNWIFLYTSVKLEHKLVMKQPVCTLDTYPASEIIMITTSAICQQGLSDGPRFYAGRIVFIFL
FIWGFLLYQFYSASIVGSLLAGKPRWINTLQDLADSNLEVGIEDIAYNYDFFATTTDPVAQQ
LYREKVAVNKKRKREPYYTIEEGLQRMQKGGFAFHVDVATAYKIIEETFDVNEICDLVEIQL
FPPKHTATATARFSPFKKMVTYGLRQVVEHGMARRLRNVWMHRKPECPESHKDDPVPIM
ITEFSPALFLMSCGWFVSMCIVIGEKLAMKKQEIGEMKDGDADAKGYEDPEDFDRETAST
NSQTSRKSG
>MmedIR76b
MRGIELMLIGIYALTSNNTIDYTLPGRYDWEENLEEVIKKSFLTSVQKEQEYNEIPKEIKVTS
WDESLYSSISEVNGEYKGGGYALKVFDIIAAKLDFKYKIVLPEKPILGDNVTGVIGMLNQS
KADVAVAFIPIIPEYLRFVQFSPILDYLEIAVLLERPLQSAIGSGLLAPFSRTVWICIFVSLALV
GPIIYIVTSYRAYLWGRTKKDKYKFIDCVWFAYGAILKQGSALTPEADSNRLMFASWWIFI
TIITSFYTANLTAFLTLSEFTLAFTSVKDIVSQHKTWSAQQHYIVDITINRNDPEELTPLRTSY
RQHRGIFYHISNKDNKTEKVASYLTNRRLFIDEIQFVENFIKNDYLNRTRQNMVEKKKCFY
IKMPNPVFQQNRGFAYPSNSTISKIINRELLHLSESGIIQHIESVNRPMVTYCPLMLGSTERK
LDNNDLELTYQVIATGFLFSLISFIVEVSQHYFRCRVCLCCADSCTCCKTRMKEPVSPSKRP
QLPLPPPPTITTVPIKTEFSSFTKPPVIPIYDTIDNFKNSKIHYVNGRDYFVVHETTGERRLVP
VRSPSALLFQYNH
>MmedIR75u
MFSAPTKWLILQDIRYDNDSNSSKNYNDQENKLKMFSAPTKWLILQDIRYDNDSNSSKNN
DDQENRLKDKLKDFEIFPDSELFISQRFDDDKIKILSIYRPSFYVDLIIEEQATWDINNGINFF
DSIPTSRRRRNLQLTPLKTCLVLTNPDTLNHLTDYKDKRIDAVTKANYIWMMHLVNQMNA
TVTYTWRDTWGYPDKNGTWSGMIGLLDRGEIDFGGTATFLIKQRIGVIEYLQLYTPVGSK
FVFRKPPLSYVSNLFTLPFGHTVWYAIGVMSCIVLGFLYITMKWEWKEIEKSPGEQDELKS
NPTISDNLLILLSAISQQGLSYEPRTISSRIVTFMLLIAALSLYASYTANIVALLQSTSSSINTLK
ELINSGLKLGIYDIVYNRYYFGVLDDPIRREFRERFVTNKTSIWITLEDGIEKVRQGLFAFH
VDTAAGYQLMQETYEEEEKCGLQEIDYMGVLDPMLVIKKKSPYREIFRVGSLWLRETGLQ
QRDTPRLFTKKPVCVGHTSFISVGTTECYAAFYTITYGAAIAFGFFFLEIILYKCFGNKMTD

DEESTECDADIEINTTEISRQSSESLEATLE

>DmelIR25a

MPRNAFGQCTLTDVIPSLWIVFINEVDNEPAAKAVEVVLTYLKKNIRYGLSVQLDSIEANKSDAKVLLEAICNKYATSIEKKQTPHLILDTTKSGIASETVKSFTQALGLPTISASYGQQGDLRQWRDLDEAKQKYLLQVMPPADIIPEAIRSIVIHMNITNAAILYDDSFVMDHKYKSLLQNIQTRHVITAIAKDGKREREEQIEKLRNLDINNFFILGTLQSIRMVLESVKPAYFERNFAWHAITQNEGEISSQRDNATIMFMKPMAYTQYRDRLGLLRTTYNLNEEPQLSSAFYFDLALRSFLTIKEMLQSGAWPKDMEYLNCDDFQGGNTPQRNLDLRDYFTKITEPTSYGTFDLVTQSTQPFNGHSFMKFEMDINVLQIRGGSSVNSKSIGKWISGLNSELIVKDEEQMKNLTADTVYRIFTVVQAPFIMRDETAPKGYKGYCIDLINEIAAIVHFDYTIQEVEDGKFGNMDENGQWNGIVKKLMDKQADIGLGSMSVMAEREIVIDFTVPYYDLVGITIMMQRPSSPSSLFKFLTVLETNVWLCILAAYFFTSFLMWIFDRWSPYSYQNNREKYKDDEEKREFNLKECLWFCMTSLTPQGGGEAPKNLSGRLVAATWWLFGFIIIASYTANLAAFLTVSRLDTPVESLDDLAKQYKILYAPLNGSSAMTYFERMSNIEQMFYEIWKDLSLNDSLTAVERSKLAVWDYPVSDKYTKMWQAMQEAKLPATLDEAVARVRNSTAATGFAFLGDATDIRYLQLTNCDLQVVGEEFSRKPYAIAVQQGSHLKDQFNNAILTLLNKRQLEKLKEKWWKNDEALAKCDKPEDQSDGISIQNIGGVFIVIFVGIGMACITLVFEYWWYRYRKNPRIIDVAEANAERSNAADHPGKLVDGVILGHSGEKFEKSKAALRPRFNQYPATFKPRF

>DmelIR40a

MACNELHNGYRAKFLTIVYWIAATYVLADVYSAQLTSQFARPAREPPINTLQRLQAAMIHDGYRLYVEKESSSLEMLENGTELFRQLYALMRQQVINDPQGFFIDSVEAGIKLIAEGGEDKAVLGGRETLFFNVQQYGSNNFQLSQKLYTRYSAVAVQIGCPFLGSLNNVLMQLFESGILDKMTAAEYAKQYQEVEATRIYKGSVQAKNSEAYSRTESYDSTVISPLNLRMLQGAFIALGVGSLAAGVILLLEIVFIKLDQARLWMLCSRLQWIRYDRKV

>DmelIR68a

MRCLWILIVAFISLAMATSIPIPIANPAPLSGYEMQLKILLQKILWVANVKRCFAVITDDLHYPIYDRIFFESVGRRVIPFFVMRTNESDDLQRPSRQVELFVKAIKSSDCELNVITILNGWQVQRFLGYIYDNRSLNMQKKFVLLHDLRLFESDMIHLWSVFIDAIFLKRQLDNKYTISTIAFPGILSGVLVMKNIANWELGKGLNGRILFADKTSNLFGTSLPVAISEHVPMVLWANATKSFQGVEVEIMNALGKALNFKPVYYKPNQTENMDWTELDGGASVAYGSGNPDGYAQNGTHIDSMLVDEVAAHSARFAIGDLHLFQVYLKLVELSAPHNFECLTFLTPESSTDNSWQTFILPFSAGMWVGVLLSLFVVGTVFYAISFLNAIINGNVSSEFFRCLRPNRNVPMDPKIYRRISFRIAISRYRSSKGDRMPRDLFDGYTNCILLTYSMLLYVALPRMPRNWPLRVLTGWYWIYCILLVATYRASFTAILANPAARVTIDTLEDLLRSHIPPSTGATENRQFFLEANDEVARKVGEKMEVFGYSDDLTSRIAKGQCAYYDNEFYLRYLRVADESGSALHIMKECVLYMPVVLAMEKNSALKPRVDASIQHLAEGGLIAKWLKDAIEHLPAEALAQQEALMNIQKFWSSFVALLIGYVISMLTLLAERWHFKHIVMKHPMYDVYNPSLYYNFKRIYPQH

>DmelIR31a

MNLLISMFILILAAGEGEIIPSMEESVVTNFVKSLVKTKQAIVFSCLFKDFKEISLALMRINQFVSVVNLNQSYSLTSILTRENYARTSVMVNARCSGSSELLFEASENRYFNKTYQWFLWGVDLEVQSLFPLNLNYVGPNAQITYVNETADGYAYWDIHSKGRHLKSNLEINLIATLINDTLNIARDIFHLQSIDFRGQFNGLTLRGASVIDKEDIISNEQIESILSRPTKDAGVAAFIKYHYELLGLLRERFNFTVNFRNSRGWAGRLGNTTFRLGLLGIVMRNEADIAASGAFNRINRFAEFDTIHQSWKFETAFLYRYTSDLDTHGKSGNFLSPFSDRVWLFCLLTLGAFSIIWVLFEIIDYKILRIR

VNSQKLEHLNQKSSVICIKTTCIERILQTFGACCQQGLDPNPVDRSVRFLVMTLFLFSLVM
YNYYTSSVVGGLLSSSDQGPSTVDEITASPLKISFEDIGYYKVLFRESQNRSITRLIEKKLSS
SRSLNELPIFSHIEDAVPYLKAGGFAFHCEVVDAYPVISEYFDANEICDLREVSGLMEVEIL
NWILHKNSQYTEIFKTAMCNAQEKGFVERILRRRQIKKPACQSLYTVYPVSLSGVLPGFVI
LICKSINKFS
>DmelIR7c
MLHSAVHNVSLVYALVWAIDNYYGMATSTPLAVVQFPTSRESRRLHNDLIDAALGRSSGT
GRIQFLLEDDRVEMTETDTDPPPPSGLTGRPIAIWFLDSLRSYFRLEMYLNQLGSPYKRNG
FFLVIYTGLEDQPMESLKIMFRRLLNMYVLNVNVFLQRDGTVHLYTYYPYGPHHCQSSLP
VYYTAFQDLAAPANGFGLTKPLFPRKLTNMHGCEMVVATFEHRPYVIIEDDPKTPGGRSIH
GIEGLIFRSLAERMNFTIKLVEQKDKNRGEILPDGNFTGILKMMVDGEVNLTFVCFMYSKA
RSDLMLPSTSYTSFPIVLVVPSGGSISPMGRLTRPFRYIIWSCILVSLIFGFVLICLLKITALPGL
RNLVLGRRNRLPFMGMWASLLGGLALYNPQRNFARYILVMWLLQTLILRAAYTGQLYLL
LQDVEMRSPIKSLSEVLAKDYEFRILPALRTIFKDSMPTTNFHAVLSLEESLYRLRDEDDPGI
TVALLQPTVNQFDFRSGPNKRHLTVLPDPLMTAPLTFYMRPHSYFKRRIDRLIMAMMSSGI
VARYRKMYMDRIKRVSKRRNLEPKPLSIWRLSGIFVCCAGLYLVALIVFILEILTTNHRRLR
RAFNVINRYAA
>DmelIR76a
MENLLVESYYFSTVLSFFAQQFFADSHATCIFWHPAFDFRLETVHPMPLIIMDWHRWANRS
DQDVYDYKIKEDEFEGKGIPYNDWTLRLTVAIERSHCETFIAFQEQIPEFARYFYHASIYSI
WRSLRNRFMFVYTKEFEDKKDSYLSGYIFQDQPNILVITSQYLNSSTFEIKTNRFVGPRNFN
KNPEPVEFYILQRFDAKGTKATWETQSAMSSKMRNLKGREVVIGIFDYKPFMLLDYEKPP
LYYDRFMNTTDVTIDGTDIQLMLIFCELYNCTIQVDTSEPYDWGDIYLNASGYGLVGMILD
RRNDYGVGGMYLWYEAYEYMDMTHFLGRSGVTCLVPAPNRLISWTLLLRPFQFVLWMC
VMLCLLLESLALGITRRWEHSSVAAGNSWISSLRFGCISTLKLFVNQSTNYVTSSYALRTVL
VASYMIDIILTTVYSGGLAAILTLPTLEEAADSRQRLFDHKLIWTGTSQAWITTIDERSADPV
LLGLMEHYRVYDANLISAFSHTEQMGFVVERLQFGHLGNTELIENDALKRLKLMVDDIYF
AFTVAFVPRLWPHLNAYNDFILAWHSSGFDKFWEWKIAAEYMNAHRQNRIVASEKTNLDI
GPVKLGIDNFIGLILLWCFGMICSLLTFLGELWRGQG
>DmelIR8a
MELPLLVLLLALRFAGSEVLKITFWIEPVQRAEFDTDIAMVLKELDALRLDVKVDDTTLTL
TRSEDGLDMQRFCEILSTVGASAVIDLTYSHWEEGYNLVRSLGIGYVRLERIMRPFLDMFG
DFMRQKRANNVAMVFMNARDAVEAMQQMLVGYPFRTLIMDASQTDPGQHFLERIRSLR
PAPTYIALFARAAAMNGIFEKVQKADLFQRPLEWHFVFLDTRDRVFKYRRQAELCTRFTL
NPRAICRSMPMPDLYCGSGFTMQRAMLLNVLRSLINAAQVSPGYPLAIYQDCNATASSSE
VSDPLEKDDYNWLDMVHWSNFLAYAPPLPHIQDQFQSPVPGLTFAVNISAGYYSSEHEAK
TDLAAWSSVGEMRLLNETISPARRFFRIGTAESIPWSYLRREEGTGELIRDRSGLPIWEGYCI
DFIIRLSQKLNFEFEIVAPEVGHMGELNELGEWDGVVGDLVRGETDFAIAALKMYSEREEV
IDFLPPYYEQTGISIAIRKPVRRTSLFKFMTVLRLEVWLSIVAALVGTAIMIWFMDKYSPYSS
RNNRQAYPYACREFTLRESFWFALTSFTPQGGGEAPKAISGRMLVAAYWLFVVLMLATFT
ANLAAFLTVERMQTPVQSLEQLARQSRINYTVVKDSDTHQYFVNMKFAEDTLYRMWKE
LALNASKDFKKFRIWDYPIKEQYGHILLAINSSQPVADAKEGFANVDAHENADYAFIHDSA
EIKYEITRNCNLTEVGEVFAEQPYAVAVQQGSHLGDELSYAILELQKDRFFEELKAKYWNQ
SNLPNCPLSEDQEGITLESLGGVFIATLFGLVLAMMTLGMEVLYYKKKQNALEITQVRPVN

DSSGSGGNSSTAPPTATSTTKQAWHIPVLEAEEKPAKVSPPPSFETATFRGKKLPARITLGDG
KFKPRHGLYARRNLGASDSHSGYME
>DmelIR75a
MQLVQLANFVLDNLVQSRIGFIVLFHCWQSDESLKFAQQFMKPIHPILVYHQFVQMRGVL
NWSHLELSYMGHTQPTLAIYVDIKCDQTQDLLEEASREQIYNQHYHWLLVGNQSKLEFY
DLFGLFNISIDADVSYVKEQIQDNNDSVAYAVHDVYNNGKIIGGQLNVTGSHEMSCDPFVC
RRTRHLSSLQKRSKYGNREQLTDVVLRVATVVTQRPLTLSDDELIRFLSQENDTHIDSLARF
GFHLTLILRDLLHCKMKFIFSDSWSKSDVVGGSVGAVVDQTADLTATPSLATEGRLKYLSA
IIETGFFRSVCIFRTPHNAGLRGDVFLQPFSPLVWYLFGGVLSLIGVLLWITFYMECKRMQK
RWRLDYLPSLLSTFLISFGAACIQSSSLIPRSAGGRLIYFALFLISFIMYNYYTSVVVSSLLSS
PVKSKIKTMRQLAESSLTVGLEPLPFTKSYLNYSRLPEIHLFIKRKIESQTQNPELWLPAEQG
VLRVRDNPGYVYVFETSSGYAYVERYFTAQEICDLNEVLFRPEQLFYTHLHRNSTYKELFR
LRFLRILETGVYRKQRSYWVHMKLHCVAQNFVITVGMEYVAPLLLMLICADILVVVILLV
ELAWKRFFTRHLTFHP
>DmelIR60b
MRRSLYLIIAIGLVDVHCVSLRYILNALENELQYRAILLVESASEIESCWEQKYIQGAVPILN
FNANQSLYLKDALNTNILALVCLNENVESTMQALYENLEDMRDTPTILFVLSDSKVQDVF
LECLRRKMLNVLAFKGLDRGFVYSFRAFPTFRVIERNVMDILQYFEQQLEDLGGHTLTTLP
DNIIPRTVVYKSPDGSRQLAGYLYPFLRNYVSTINATLKVCWHLVPEDGMIQLGEVVRLSE
IHDVDFPLGMHGIEHGSTSQNVPLEVSSWFLMLPMEPSLSRAQFFIMLGFEKVTPVLLLLTI
LLSTAHRIEMGLRPSWRCYVLGDRVLQGTLGQAFFLPRRLSVKLMLVYSLILLNGFTFSNY
SITSLETWLVHPPSGHPIHSWEQMRTLNLKVLIVPSELDSMTKALGKQFTESNSDLFELSKS
GNFQDKRLAMDQSYAYPVTCTLWPLLEHAQIRLPKPEFRRSREMVLIPLLIMAMPLPKNS
MFHKSLNRYRALTHQSGLYEFWFKRSFNELVALRKIHYKVNGDHQIYRDFEWQDFSYVW
LGFVGGTIASILVLLAEIGYHRWQLNQN
>DmelIR56b
MLLDTDLASGVIRSPYSFDIPHAFIFNETQFVVPKFCGPYMEIVKHFAEVYHYQLFLDSLES
LPKKSVVEQDIISGKYNLSLHGVIIRPEETSDFFNATQHSYPLELMTNCVMVPLAPELPKW
MYMVWPLGKYIWTCLFLGTFYVALLLRYVHWREPGNATRSYTRNVLHAMALLMFSAN
MNMSVKLKHASIRVIIFYTLLYIFGFILTNYHLSHMTAFDMKPVFLRPIDTWSDLIHSRLRIV
IHDSLLEELRWLPVYQALLASPSRSYAYVVTQDAWLFFNRQQKVLIQPYFHLSKVCFGGL
FNALPMASNASFADSLNKFILNVWQAGLWNYWEELAFRYAEQAGYAKVFLDTYPVEPLN
LEFFTTAWIVLSAGIPISSLAFCLELFIHRRKQRRPQYERFECYDY
>DmelIR54a
MWTVITGIVLWAPVLVAGSAVDFIFRAAAEHSLSVIMIRIDYCPYNWAKDIFENQTIPVVVL
SDSETFINIRMFSRPLHVACLPGHELQKDLALLENFTSSLMDFPSQKKIVYISNNFSDPTRM
DYIFETCYHRRIWNIVGLLASDEHRYFYRYHLYPSFRTEYRSLESSTIFDKDFPNMHGHPLT
VMPDQWLPRSVLYVDRRTGKQILAGSVGRFFHVLSWKLNATLQLSKKVTTGRFLNATAL
KELSESFSVDVPASLTIMERVEQLASTSYPMEVTHVCLMVPVARRIPIKDIYFILSSASNMFL
AIVIVSSYGLALNLLRNMTHRDVRLVDFVLNDKALRGILGQSFNLPLSRSFSTRLIFLMLGI
VGLNVSSIFGAGLDTLMAHPPRQFQARSFAGLRRTKIPLVTTEEDFPTWMKLRVPMLVVN
VSEYNHLRNGRNTSNAYFASRLYWNLFSEQQKRFTRELFIYSTDDCLWSLALLSFQWPQN
SLFTEPVSQLILEVNANGLYDFWVGMHYYDMTAAGLSGLEDPSLQLKEREHPTSLRIVDF
QWMWQAYGTFMVIAILVFLLEVSWHRITSLFVSLVY

>DmelIR85a
MSIQWLKHILLLAILVNLAGTRENHIPLDLKKSSIVMVKMSQILCKARIKVLFVYFENQTS
HEHTGQILKEVTKCDISNQNTPLEAVKDDGILMYMVMITTNISQPLELSLIRKKSAAKHRS
HVFLLVRDADTVSDAWMRASFRQFWKIWLLNIVILYWRDGRLNAYRYNPFMDNYLIPVD
NKPNEVPTLEQLFPKTIPNMQRKPLRMCIYKDDVRAIFWRQGTILGTDGLLAAYVAERLN
ATMMITRPHSYNNHNLSSDICFLEVAKEYVDVAMNIRFLVPDTFRKQAESTVSHTRDDLC
VIVPKAKTAPTFWNIFRSFGSLVWALILVSVLVANVFCYILKSEVGRVPMQLFAGALTMPM
TQIPPNHSIRLFLIFWLYFGLLICSAFKGNLTSMMVFQPYLPDINQLGALARSHYHIIIRPRH
VKHIQHFLTLGHKHESRIREQMLEVSDTQMYEMMRNNDIRFAYLEKYHIARFQVNSRVH
MHLGRPLFHLMNSCLVPFHAVYIVPYGSPYLGFLDSLIRSSHEFGFERYWDRIMNSAFIKS
GVKVVNRRRGSGNDEPVVLKLQHFHAVFALWLVGIGMACIVLAWEHLTHNYNLAVTKRR
D
>DmelIR67a
MLPILVPVLLLFNETSWINPILTSIYKDRHHETVLLLQHSQHGNASGLERFPWPVFSFNEQM
DFYVRGKYNSEMLVLIWQTGNSDWDLDLWQALDRSLLNMRKVRVLLLRKWEKIPTADV
AATAEHLLFLHVAVIGQGNRIYRLQPYAPQSWLQVDPIESPIFIKIRNYFGRYIVTLPDQFPP
RSIVYRNPKTDEIQMTGYVYKFLLEFIRIYNFTFRWQRPIVQGERMNLILLRNMTLNGTINL
AISLCGFETPSELGVFSDVYDMEEWYIMVPRAQEISIADVYVVMVSGNFLIVLIIFYFIFTIL
DTCFGPLLLKERVDWSNLMLNERMISGIMGQSFNMSARNTISSKVTNATLFLLGLVLSTLY
AAHLKTLLTKRPTSQQISNFKQLRDSPVTVFFEEAERFYLKHAWDRPIRYIKDQLNFRETIE
YNALRMGLNRSNAFSALTSEWMIVAKRQELFKQPIFTVQPELRVIQTSVLLSLVMQSNSIY
EDHINDLIHRVQSAGIVEYWKHQTLREMITMGMISQKDPFPYVAFREFKVGDLFWIWLLW
VSFLFMSFVIFLCELLVDCFISKTLIRNKRPH
>DmelIR21a
MSYYWVALVLFTAQAFSIEGDRSASYQEKCISRRLINHYQLNKEIFGVGMCDGNNENEFR
QKRRIVPTFQGNPRPRGELLASKFHVNSYNFEQTNSLVGLVNKIAQEYLNKCPPVIYYDSF
VEKSDGLILENLFKTIPITFYHGEINADYEAKNKRFTSHIDCNCKSYILFLSDPLMTRKILGP
QTESRVVLVSRSTQWRLRDFLSSELSSNIVNLLVIGESLMADPMRERPYVLYTHKLYADGL
GSNTPVVLTSWIKGALSRPHINLFPSKFQFGFAGHRFQISAANQPPFIFRIRTLDSSGMGQLR
WDGVEFRLLTMISKRLNFSIDITETPTRSNTRGVVDTIQEQIIERTVDIGMSGIYITQERLMD
SAMSVGHSPDCAAFITLASKALPKYRAIMGPFQWPVWVALICVYLGGIFPIVFTDRLTLSH
LMGNWGEVENMFWYVFGMFTNAFSFTGKYSWSNTRKNSTRLLIGAYWLFTIIITSCYTGS
IIAFVTLPAFPDTVDSVLDLLGLFFRVGTLNNGGWETWFQNSTHIPTSRLYKKMEFVGSVD
EGIGNVTQSFFWNYAFLGSKAQLEYLVQSNFSDENISRRSALHLSEECFALFQIGFLFPRES
VYKIKIDSMILLAQQSGLIAKINNEVSWVMQRSSSGRLLQASSSNSLREIIQEERQLTTADT
EGMFLLMALGYFLGATALVSEIVGGITNKCRQIIKRSRKSAASSWSSASSGSMLRTNAEQL
SHDKRKANRREAAEVAQKMSFGMRELNLTRATLREIYGSYGAPETDHGQLDIVHTEFPNS
SAKLNNIEDEESREALESLQRLDEFMDQMDNDGNPSSHTFRIDN
>DmelIR60e
MVIKMISFLLVSVLLCLVGASDSESMQVQVLQDLNLALQTELNVFIDFECCATSEILHKLD
SPRILLSSNSREARDLRIRGNFTESTLIIVSVMDSDLNPLVASLLPRLLDELHELHIVFLSNEE
PGFPKQDLYTYCFKEGFVNVILMSGKGLYSYLPYPSIQPISLSNVSEYFDRARIIRNFQGFPV
RILRSTLAPRDFEYSNEQGGLVRAGYLFTAVKELTYRYNATIESVPIPDLPEYDVYLAVAEM
LHTKKIDIVCYFKDFSLEVAYTAPLSIIREYFMAPHARPISSYLYYSKPFGWTLWAVVISTVL

YGTVMLHLAARGARVEIGKCLLYSLSHILYNCHQKIRVAGWRDVAIHGILTIGGFILTNVYL
ATLSSILTSGLYDEEYNTLEDLARAPYPSLHDEYYRSQMKAKTFLPERLRRNSLSLNATLL
KAYRDGLNQSYIYILYEDRLELILMQQYLLKTPRFNMIRQAVGFTLESYCVSNSLPYLAMT
SEFMRRLQEHGISIKMKADTFRELIHQGIYTLMRDDEPPAKAFDLDYYFFAFVLWTVGLIS
SLLVFFAELVSGHL
>DmelIR41a
MFIDLSWSLVLSAIVGKYLNESTICIFWNDKFEFQLLHKSDYISFVGINIKSFDDNGGHYIID
TGLKKKELQNKHLFLDELVIKIIISIEVTHCETFVVFDKDIDRFVNAFNKASVYSIWRSLHN
KFVFAHIANESPESRNHFFEDQPNILFVVRDHSSASSFDIKTNKFVGRKAENPSQMILVDRY
LASEQRFQFGKSLFADKLNNLQGREVIIAGFDYPPYTVIKHNMSTNAQDMGVSGESDFKN
VYIDGTETRIVLNFCEQFNCTIQIDSSAANDWGKVYPNMSGDGALGMLINRKADICIGAM
YSWYEDYTYLDLSMYLVRSGITCLVPAPLRLTSWYLPLEPFKETLWAAILLCLCAEATGLV
LAYKSEQALYVLPGYREGWWTCTSFGVCTTFKLFISQSGNSKAYSLTVRVLLFACFLNDLII
TSIYGGGLASILTIPSMDEAADTVTRLRFHRLQWAANSEAWVSAIRASDEALVKDILYNFHI
YSDDELLRLAQDQHMRIGFTVERLPFGHFAIGNYLGPQAIDQLVIMKDDIYFQYTVAFVPR
LWPLLDKLNTLIYSWHSSGFDKYWEYRVVADNLNLKIQQQVQETMTGTKDIGPVPLGMS
NFAGFIIVWILGSAIATLTFLLELSLTYILKQSNLK
>DmelIR94c
MSKVFKLLVLPLIYLSLTKGSKNPQLKFLRELINVIEEGREIRTIMVIKHSRDEYCHLDQWN
PRGSPILRTNEMGSIRISGYFNDQAVILACMGENSDYGLLKSLANAMDNMRQERIILWSER
EPTKMLMDYISQQADRYNFAQIIIVTMNEDVDAVPSLHQLNPYPTPRFRQITNISNIRRTSFF
GCGLSFQGKTAILKESVVSNIRFKVWSPSGPIPLSELKDYEIVQFAVKYNLSLKLYDQNESK
SDHFDIQLGPLFITKDFPTQMAFVSPNTACSLIVIVPCSPKWRFMDVLHKLGVLKLIGCLLI
AYAVFVLIETLILWLTHRISGREVRLTSLNQLLNPRAFRGILGLPFPEFRRSSISLRQLFLVISV
FGLVYSNFVSCTLSALLTKPAQNPQVRNFKELRDSGLITIMDKYTHSFIEKHIDPEFFDHVL
PHYLILQKKEALRMIWNFNDSYSYVMYTTTWKSLNTVQKSFDERVFCESESLTIAWNLPR
MYVLGNNSVLKWMLSRYITYMPQTGIPDSWTEQLPKVLKLLYNVTSPRRIKEGAVPLSIQ
HLSWIWHLLFIGESIATLVFIVEILLQKSNQHTSNMRERSSEDDDFV
>DmelIR94b
MSLIFNLLFILILSQAVSQETEFLQLKYLNNIVRSMIKLHKMETLVIVKHHLDNNCSLQNW
NAHGMGIIRTNDQGKLIMKDTFNSRTLAIICIGQNSHITLLRNVFETFGKVQQKKIILWTQM
ELKEKFFQEISKKSRDLKLLNLLVLKAVTKDKLLIYRLNPFPSPHFKRIENIWTPNDTLFMD
TKFNFHGMTAVVKHDYNWTIQMGNIRKFPISRIEDKEVIEFALKYNLTLQFFNDVERFDIEL
RKRIILKSNSTQPIDSGIPMVFSSLLIVVPCGNYLSIQDVIKVSGIEKWIFYIILVYVIFVLIEIT
FLGVTILISRQSRHQMIPNTLVNLCAFRAILGLPFPETRRTSLSLRQLFLAIALFGMIFSIFINC
KLSSMLTNPCPRPQVNNFEELKTSGLTVVMDHDAENFIEKEIGVDFFNQYMPRKVTLTFTE
RAKLLFSLKGNHAFTLFSESFAIIESYQRSKGLRAHCTSEDLIVAERVPRIYILENNSILDRPL
RRFIRQMQESGITNHWLKNIPSSLEKNLMQITIPYDRERVHPLSIEHLTWLWCILILGYSISM
IVFFVEMSLKRRKKNLENRAPNICIC
>DmelIR93a
MNPGEMRPSACLLLLLAGLQLSILVPTEANDFSSFLSANASLAVVVDHEYMTVHGENILAH
FEKILSDVIRENLRNGGINVKYFSWNAVRLKKDFLAAITVTDCENTWNFYKNTQETSILLI
AITDSDCPRLPLNRALMVPIVENGDEFPQLILDAKVQQILNWKTAVVFVDQTILEENALLV
KSIVHESITNHITPISLILYEINDSLRGQQKRVALRQALSQFAPKKHEEMRQQFLVISAFHEDI

IEIAETLNMFHVGNQWMIFVLDMVARDFDAGTVTINLDEGANIAFALNETDPNCQDSLNC
TISEISLALVNAISKITVEEESIYGEISDEEWEAIRFTKQEKQAEILEYMKEFLKTNAKCSSCA
RWRVETAITWGKSQENRKFRSTPQRDAKNRNFEFINIGYWTPVLGFVCQELAFPHIEHHFR
NITMDILTVHNPPWQILTKNSNGVIVEHKGIVMEIVKELSRALNFSYYLHEASAWKEEDSL
STSAGGNESDELVGSMTFRIPYRVVEMVQGNQFFIAAVAATVEDPDQKPFNYTQPISVQKY
SFITRKPDEVSRIYLFTAPFTVETWFCLMGIILLTAPTLYAINRLAPLKEMRIVGLSTVKSCF
WYIFGALLQQGGMYLPTADSGRLVVGFWWIVVIVLVTTYCGNLVAFLTFPKFQPGVDYLN
QLEDHKDIVQYGLRNGTFFERYVQSTTREDFKHYLERAKIYGSAQEEDIEAVKRGERINID
WRINLQLIVQRHFEREKECHFALGRESFVDEQIAMIVPAQSAYLHLVNRHIKSMFRMGFIE
RWHQMNLPSAGKCNGKSAQRQVTNHKVNMDDMQGCFLVLLLGFTLALLIVCGEFWYR
RFRASRKRRQFTN
>DmelIR92a
MLLQPLVMHLSQLLRIIVGQYFAEFPSILIVYNNSASTTPLQLEYLSALELVLRELSKPIRLQ
WINVAFLKDLNDLEDQVMGALNSSVTEGFITILSQTHHFIHARYYATRNANVRLKDKRYL
FLCEDESPAELLCMDILQFYPHHLMVRPGTETAPTGPTGPHPDPRRGGGASVSTKNKDDG
EGGAGNKTTSPYRDINFELWTQKFVGAVGNLDALLLDAFLPNETFANRVELYPNKLLNLQ
RRSLLVGSITYVPYTITNYVPAGQGDVDPIHPQWPNRSLTFDGAEANVMKTFCQVHNCHL
RVEAYGADNWGGIYDNESSDGMLGDIYEQRVEMAIGCIYNWYDGITETSHTIARSSVTIL
GPAPAPLPSWRTNIMPFNNRAWLVLISTLVICGTFLYFMKYVSYRLRYSGTQVKFHHSRKL
EKSMLDIFALFIQQPSAPLSFDRFAPRFFLATILCATITLENIYSGQLKSMLTFPFYSAPVDTIE
KWAQSGWKWSAPSIIWVHTVQSSDLETEQILARNFEVHDYSYLSNVSFMPNYGFGIERLS
SGSLSVGDYVSTEALENRIVLHDDLYFDYTRAVSIRGWILMPELNKHIRTCQETGLYFHWE
LEFIDKYMDKKKQEVLMDLANGHKVKGAPQALDVRNIAGALFVLAFGVAFAGCALVAE
LLIHRMDLSK
>DmelIR87a
MSTPEQRFWLAALLFLLSQHSEVRGFGINLMKVQTEDKGQEACILALLRKYFDSGDGLSG
SVLCINRNYQLPNIEEQLLRGVNNYENYPWSLLITNSREGPSPAKFLMNEKPQCYFLIVDN
LEDEDLDEVFEHWKGMVNWNPLAQFVVYLASLEETDEEMNDLMVELLLTFINKKIFNVN
VIGQSEENQFYYGKTVFPYHPDNNCGNRVISVELLDACDYPSEETDSEDENDEDEGDGAQ
EEDDGPQEEGDGEQEEEDGPQEQEDGDQAKGDEGQENDDGGLENKVENEFRIGASDDD
ELENDLSSNSSEPEAIIEEFFRAKFEDKFPRDLSGCPLTASFRPWEPYIFRNSEEQPVDDYYY
GLQGDEDDYNDTSPNYGESDDESYADPGEDGDGAIPDTETQSGGKLKLSGIEYEMVQTIA
ERLHVSIEMQGENSNLYHLFQQLIDGEIEMIVGGIDEDPSISQFVSSSIPYHQDELTWCVAR
AKRRHGFFNFVATFNADAGFLIGIFVVTCSLVVWLAQRVSGFQLRNLNGYFPTCLRVLGIL
LNQAIPAQDFPITLRQLFALSFLMGFFFSNTYQSFLISTLTTPRSSYQIHTLQEIYSNKMTVM
GTSEHVRHLNKDGEIFKYIREKFQMCYNLVDCLNDAAQNEHIAVAVSRQHSFYNPRIQRD
RLYCFDRRESLYVYLVTMLLPKKYHLLHQINPVIQHIIESGHMQKWARDLDMRRMIHEEIT
RVREDPFKALTFDQFRGAIAFSGGLLLVASCVFAFELCYVKYVYRTEKRERKTKKITKKVH
NIKIQHD
>DmelIR84a
MIKLQVKVISWPLIILTAFLRVLQIESINTNFLELAAFEDFLRSEHLSHVLVVRGDDADGDW
KIECHQKLLANYRVQFYRPEMSANFEDLMFYGSPRTAVLVLNSEHVLVRRQVFGVASEAG
YFNNSLAWFILGSGRESLPVEQLIDQLLSGYRMGIDADITVALRGPDNASMLFYDVYRISR
QANTPLIIEKKGLWTHSGGYQKFGNFKNTWVIRRRNFLNVTLIGSTVLTEKPPGFGDMEY

LADDKQLQQLDPMQRKTYQLFQLVERMFNLSLAISLTDKWGELLDNGSWSGVMGQVTS
READFAVCPIRFVLDRQPYVQYSAVLHTQNIHFLFRHPRRSHIKNIFFEPLSNQVWWCVLA
LVTGSTILLLFHVRLERMLSNMENRFSFVWFTMLETYLQQGPANEIFRLFSTRLLISLSCIFS
FMLMQFYGAFIVGSLLSESARSIVNLQALYDSNLAIGMENISYNFPIFTNTSNQLVRDVYV
KKICKSGEHNIMSLQQGAERIIQGRFAFHTAIDRMYRLLLELQMDEAEFCDLQEVMFNLP
YDSGSVMPKGSPWREHLAHALLHFRATGLLQYNDKKWMVRRPDCSLFKTSQAEVDLEH
FAPALFALALAMVASALVFLLELFLHWLPDFRRRLGTMST
>DmelIR7b
MKYWLYILSCCSLVASTMESSSDWDLAEALAQVVANSEMGRFKTLYIYTHTNSQSTGGHL
EELLDQVLMIVPNNLQARRLLLQQSMEYKPYVHAVLALVDGLPSLSAIYARIRATQDLSHT
LIYMSMPTDAYGEEMQATLRFLWRLSVLNVGVVLRPPGDHILMVSYFPFSALHGCQVISA
NVVNRYQVGTKRWASQDYFPSKLGNFYGCLLTCATWEDMPYLVWRPDGSGSFVGIEGAL
LQFMAENLNFTVGLYWMNKEEVLATFDESGRIFDEIFGHHADFSLGGFHFKPSAGSEIPYS
QSTYYFMSHIMLVTNLQSAYSAYEKLSFPFTPLLWRAIGLVLILACLLLMLLVRWRHHHEL
PRNPYYELLVLTMGGNLEDRWVPQRFPSRLVLLTWLFATLVLRSGYQSGMYQLLRQDTQR
NPPQTISEVLAQHFTIQLAEVNEARILASLPELRPEQLVYLEGSELQSFPALAQQSGSSARVA
ILTPYEYFGYFRKVHPMSRRLHLVRERIYTQQLAFYVRRHSHLVGVLNKQIQHAHTHGFL
EHWTRQYVSAVDEKDESVARIASTSYSTLDGIDGDPSLSESEEDQQVAPVRQNVLSMREL
AALFWLILWANLGAVVVFVLELLLPRIKLRKILRKMKSDIKKQISKLVRK
>DmelIR75d
MKVQVAHWLPLIFFLLVSGTPRVAGSWRSEYSRQDPDPKTRWGNQLPDMLVAYYRHHGV
HSLMLVVCHTDIADFRLWKLWQHFNLNNFYVQVSTESSLRDLQHVDALDEHKDAPPPKS
FHANNSTHWETSFLLPALPYKMGILLLEFSSECALNLLRWSAASEHNYFTTNRFWLLLTED
PGDIDLLEDPEIFIPPDSELRVLHYENVGNFSCSLIDLYKVAAWKPLKRTLVGHNIRNSRHVI
HALQHFGSAITYRQDLEGIVFNSAIVIAFPDLFTNIEDLSLRHIDTISKVNHRLMLELANRL
NMSYNTYQTVNYGWRQPNGSFDGLMGRFQRYELDLAQLAIFMRLDRIALVDFVAETYRV
RAGIMFRQPPLSAVANIFAMPFENDVWVSILMLLIITTVVLVLELFFSPHNHDMSYMDTLN
FVWGAMCQQGFYVEVRNRSARIIVFTTFVAALFLFTSFSANIVALLQSPSDAIQSLSDLGQS
PLEIGVQDTQYNKIYFTESTDPVTKNLYHKKIASKGENIYMRPLLGMEKMRTGLFAYQVE
LQAGYQIVSDTFSEPEKCGLMELEPFQLPMLAIPTRKNFPYKELIRRQLRWQREVSLVNRE
ERKWIPQKPKCEGGVGGFVSIGITECRYALGIFGCGAAVSFVLFLFEFIFRHFKQVYRIIKGY
REVQR
>DmelIR76b
MATGIELLVAAALCVACPPLNDSPPTNLIQMGENGTLSPVTELPMDVDASEAGFDADAPV
ETLETINRKKPKLREMLDWIGGKHLRIATLEDFPLSYTEVLENGTRVGHGVSFQIIDFLKKK
FNFTYEVVVPQDNIIGSPSDFDRSLIEMVNSSTVDLAAAFIPSLSDQRSFVYYSTTTLDEGE
WIMVMQRPRESASGSGLLAPFEFWVWILILVSLLAVGPIIYALIILRNRLTGDGQQTPYSLG
HCAWFVYGALMKQGSTLSPIADSTRLLFATWWIFITILTSFYTANLTAFLTLSKFTLPYNTV
NDILTKNKHFVSMRGGGVEYAIRTTNESLSMLNRMIQNNYAVFSDETNDTYNLQNYVEKN
GYVFVRDRPAINIMLYRDYLYRKTVSFSDEKVHCPFAMAKEPFLKKKRTFAYPIGSNLSQL
FDPELLHLVESGIVKHLSKRNLPSAEICPQDLGGTERQLRNGDLMMTYYIMLAGFATALAV
FSTELMFRYVNSRQEANKWARHGIGRTPNGQSVAPSRWLRGWRRLNSGHGQLLGASTHG
QNVTPPPPYQSIFNGGSHGDPLNRWRRPLANGNALGNGVLLGGDSEGGVRRLINGRDYM
VFRNPNGQSQLVPVRSPSAALFQYSYTE

>DmelIR75c
MTSWPLYRLIVFNLLEINLSNLMVFHCWSIKEAFPLVEMLNQNGIFSQYIDVQNPDNLANV
HKEYLDSDLVRLGVFLDLGCDKAELVTNQSSRARLYNQNLHWLLYDEAGNFTKLTQLFE
GANLSLNADVTYVSREDEERFILHDVYNKGSHLGGKLNITVDQTLQCNRSHCQVKEYLS
ELHLRPRLQHRMDLSSVTFRLAALVSVLPINSSEEELLEFLNSDRDSHMDSISRIGNRLIMH
TQEILGFKLHYIWCGTWSVQDAFGGAIGMLTNESAELCTTPFVPSWNRLHYLHPMTEQA
QFRAVCMFRTPHNAGIKAAVFLEPFMPSVWFAFAGLLIFAGVLLWMIFHLERHWMQRCLD
FIPSLLSSCLISFGAACIQGSYLMPKSAGGRLAFIAVMLTSFLMYNYYTSIVVSTLLGSPVRS
NIRTIQQLADSSLDVGFDTVPFTKTYLVSSPRPDIRSLYKQKVESKRDPNSVWLSPEEGVIR
VRDQPGFVYTSEASFMYHFVEKHYLPREISDLNEIILRPESAVYGMVHLNSTYRQLLTQLQ
VRMLETGITSKQSRFFSKTKLHTFSNSFVIQVGMEYAAPLFISLLVAYFLALLILILEICWARY
AKKKFSTIIPQNQ
>DmelIR75b
MLQLHNLILHNLIHMAKLSHVLILHCSLSHLALLAQSKNIFTQFQPLHSDIQLNDDFLNHNI
LKLGVFLDINCDKSGTVLDMASAKRFFSHRYHWLIYDRSMNFSVLESHFKEAQIFVDADV
TYVTHDPFSKNFLLYDVYNKGRQLGGELNITADREIFCNKTNCRVERYLSELYTRSALQHR
KSFTGLTMRATAVVTALPLNVSIKEIFDFMNSKYRIQLDTYARLGYQARQPLRDMLDCKFK
YIFRDRWSDGNATGGMIGDLILDKADLAIAPFIYSFDRALFLQPITKFSVFREICMFRNPRS
VSAGLSATEFLQPFSGGVWLTFALLLLLAGCLLWVTFILERRKQWKPSLLTSCLLSFGAGCI
QGAWLTPRSMGGRMAFFALMVTSYLMYNYYTSIVVSKLLGQPIKSNIRTLQQLADSNLD
VGIEPTVYTRIYVETSEEPDVRDLYRKKVLGSKRSPDKIWIPTEAGVLSVRDQEGFVYITGV
ATGYEFVRKHFLAHQICELNEIPLRDASHTHTVLAKRSPYAELIKLSELRMLETGVHFKHE
RSWMETKLHCYQHNHTVAVGLEYAAPLFIILLGAIILCMGILGLEVIWHRHCTLH
>DmelIR60d
MRLAIYVAFLSSIGNRSGFLSSLLMSLGKELHYKTILLVGGSSTCWSLEPFETGVPILNLRG
ENNAYPQDTFNSQMLALACLQTESEDAVKLLYRSLKDMRDTPTLLFASSEEHIHDTLFLGC
FRENMLNVLALTASSKEFIYSYQAFPTFRVIKRKLVEIHRYFEPQLKDLGGHIVSALPGNIM
PRTMCYRNAEGERQLAGYLNTFIRNYVESINGTLRISWGLVPEDDMRHLTISRLSKIQHVD
FPLGIIPLYNKTDKQHVYMEISSWFLMLPMETSVPRAHLFVKLGLERLLPIIVVVGAVLGN
AHRIEVGLGPSWRCYYLADKVLRGALAQPIVLPRRLSPKLMLIYSLLLLSGFFLSNYYMA
SLTTWLVHPPASDRILEWDQLRYLHLKVLTIPEEFKYMSLILGTDFMTAYGSIFQLTNSTDF
QRRRISMDPSYAYPVTTSLWPFLELSQVRLRRPLFRRSYDMVLQPFQVMSLPLPRNSIFHK
SLLRYAALTRETGLYYYWFRRSYYELVALGKISYKEEEGNPYCDLKWNDFRIVWLAFLGG
TIISCLALLLEVAHYRWHLGNSSL
>DmelIR94h
MLSNISFSSAPELVDLYGLVLKFLVSSETTLFYFNPTGQKCSWETLPRTILSNHPQIIWFREE
TYPGLYKRHSSNLFVMACLSSTSYDGQLQLLAESLTRYRSVRVLIEVQDKEGSFLASQILL
LCQQHSMLNVVLYFSRWTRTLNVFSYLAFPYFKLLKQRLSGSLRPKIFINQLKDLQGYKIR
VQPDLSPPNSFSYRDRHGECQVGGFLWRIVENFSKSLKGDTQVLYPTWAKAKVSAAEYMI
QFTRNGSSDIGVTTTMITFKHEERYRDYSYPMYDISWCTMLPVEKPLSVEILFSHVLSPGS
ALLLILAFILFFLIVPQLIKCLGITFRGRLIGMASRIFALVMLCSSSAQLLSLLMSPPLHTRIKS
FDDLLTSGLKIFGIRSELYFLDGGFRAKYASAFHLTENPNELYDNRNYFNTSWAYTITSVKW
NVIEAQQRHFAHPVFRYSTDLCFSSETPWGLLIAPESFYREPLQHFTLKINQAGLITQWMT
QSFHEMVRAGRMTIKDYSRTNLMKPLRIQDLRKCWVIFAVGLGTSTVVFTIELLLIYTNVF

LNSL
>DmelIR94g
MSTAVNSVHSKLVSLISRGQELTSIFFYAPAKEKCHLEDTISSATWGLPLVIWRTDRTVILNG
FIGEGLLVLACLPGFHWRALLGSLARSLKYLRQARILIELMQDRDEFLVSEVLQFCLSQDM
INVNAIFDDFPETENLSSFEAYPSFEVVNQTFTPDTQVSDLYPNKMLNLRGGVIRTMPDYSE
PNTILYQDKEGNKEILGYLWDLLEAYAHKHNAQLQVVNKYADDRPLNFIELLDAAQSGII
DVGASIQPMSMGSLSRMHEMSYPVNQASWCTMLPVERQLHVSELLTRVIPYPTLALLLLL
WIFYEVLRGRWRRHSRLQSIGWLVLATLVSSNYVGKLLNLFTDPPSLPPVNSLAALMESPV
RIISIRSEYSAIEFTQRTKYSAAFHLALHASILIGLRNAFNTSYGYTITSEKWKIYEEQQKRSS
KPVFRYSKDLCFYEMIPFGLVIPENSPHRAPLHSYTLLLRQAGLHDFWVNRGFSYMVKAG
KINFTAVGERYEAKTLTITDLRNVFIIYVSVLLISLILFTCELFVSWVNYWLGF
>DmelIR7a
MFHHLWLLMGLRSLAMGALHPPQPEAMTPLVAAALEILAEQVSPSQSTLAVMDLTQDAE
H
RDERQEQLMTIILRSVGSEMALRTFQKPPAEVPASFVVFLVNSAQAFNTLGFHFTDIHST
REFNFLILLTHRMSSRAERLQVLRDISRTCVRFHTSNVILLTEKRDGVVLVYAYRLLNMD
CDLSVNLELIDIYKNGLFRHGHEARSFNRVLSLSGCPLQVSWYPLPPFVSFIGNSSDPEE
RAQIWRLTGIDGELIKLLASIFDFRILLEEPCNKCLSPDIKDDCSGCFDQVIISNSSILI
GAMSGSHQHRSHFSFTSSYHQSSLVFIMHMSSQFGAVAQLAVPFTVIVWLALVVSSLLLV
LVLWMRNRLVCGRSDLASHALQVLTTLMGNPLEARSLPRSSRLRILYAGWLLLVLVLRVV
YQGKLFDSFRLPYHKPLPTEISELIRSNYTLINQEYLDYYPRELTVLTRNGSKDRFDYIQ
GLGKEGKFTTTSLIATMEYYNMMHWSTSRLTHIKEHIFLYQMVIYLRRHSLLKFAFDRKI
KQLLSAGIIGYFVREFDACQYRKPFEEDYEVTPIPLDSFCGLYYISLIWLSAAVVAFILE
LLSQRIVWLRRIFE
>DmelIR7d
MDIRCVVALLLGLCKVQAVVWPHQHLLEEQLASQISATLQKIFINGLAVYNFGVFISTSY
EEMDRDRVILVHQVLNRNLYPPNFPVAVVLASKMNRKITAQVFTQLLFVQNAEQAIAIAE
GVNRNGLCVIVLLTSQPERPIMTKIFTYFMQERYNINVVILVPRLHGVQAFNVRPYTPTS
CSSLEPVEIDIKDGDLWDVFPRRLKNLHGCPLSVIVWDIPPYMRINWKSSDPMDGLDGLD
GLLLRIVARKMNFTLKLIPNEPNGLIGGSSFMNGTFTGAYKMLRERRANITIGCAACTPE
RSTFLEATSPYSQMSYIIVLQARGGYSIYEVMLFPFEKYTWLLLSTILGLHWIVGSRWRM
PSPILAGWMLWIFVIRASYEASVFNFIQNSPVKPSPRTLDQALSGGFRFITDHASYRMTL
KIPSFQGKTLISAGQPVDVFDALLKAPWKTGAFTSRAFLADHLVRHRKHRNQLVILAEKI
VDNMLCMYFPHGSYFAWEINKLLFNMRSFGIFQHHSQILAWDNLPTTTDTDTPGKRIHSS
TESVATGFAESMSFVVAALNCLMGALCISIVVFGLELLSRRRHWTGLEWLFERV
>DmelIR7e
MNHINEFVARAVLHVVHHYILSVTPSLVLTLCCRSNHTCNFYNKMMSTLFREWGLAPLQI
VNVLRGVPWHPVPGRRHFNVIFTDSFAAFEEIRMEYYSREYNYNEHYFIFLQARDRLLQG
EMRLIFDYCWRYRLIHCSIQVQKSNGDILFYSYYPFGEHGCSDMEPQLINRYNGSMLVEP
DLFPRKLRNFFGCPLRCALWDVPPFLTLDEDQEEVLRVNGGYEGRLLLALAEKMNFTIAV
RKVHVNMRDEALEMLRRDEVDLTLGGIRQTVARGMVATSSHNYHQTREVFGVLASSYEL
S
SFDILFYPYRLQIWMGILGVVALSALIQLIVGRMLRERMGSRFWLNLELVFVGMPLLECP
RSHTARLYCVMLMMYTLIIRTIYQGLLYHLIRTHQLNRWPQTIESLVQKNFTVVLTPIVQ

EVLDEIPSVQHMRFRLLEANSELDPLYFLEANHQLRQHVTASALDIFIHFNRLSADKVHQ
RGEQGSGAHFEIVPEDIISMQLTMYLAKHSFLIDQLNEEIMWMRSVGLLSVWSRWELSES
YLRNEQSFQVLGTMELYAIFLMVLVGLIVGLLVFILELVSMRSIYLRKLFT
>DmelIR7f
MQGEDANLYVARALRLVIENVLAQLSTTLVVTISTRHLGTAHWFEYMMNILMDSWRMVA
V
QLLRIRPDLVVNPVPGRKRVSLLMVDSYQGLLDTNITASNANFDDPDYYFIFLQARDHLI
PKELQLILDHCLAHFWLHCNVMIQTAQVEVLVYTYYPYTADACQKAYPIPVNTFDGRKW
K
ASQMFPDKLSQMHGCPLTVLTWHQPPFVELVWDPKHNRSRGSGFEIQLVEHLARRMNFS
L
ELVNIALLRPNAYRLAEGSSEGPIEKLLQRNVNISMGYFRKTARRNQLLTTPMSYYSANL
VAVLQLERYRIGSLALLVFPFELSVWMLLLLALLIHLGIHLPSARRGNEEDGGGGLQVVA
LLLGAALARLPRSWRHRFIAAHWLWASIPLRISYQSLLFHLIRLQLYNTPSFSLDQLLAE
GFQGICTANTQRLLLEMPQLARDPDSIQSVDTPFDWDVLNVLTRNRNRKIFAVANQDVTL
SFLHSSAHPNAFHVVKQPVNVEYAGMYMPKHSFLYEKMDDDIRRLDASGFIHAWRRASF
A
SVHRKEQVHMTSRRYINHAKLSGIYMVMAGLYLLAGLLFAGEVLLRQRN
>DmelIR7g
MNVTSLLNFESMKYIGAQTQAASINHHVAQALRVFIEDFYQRIAPAFIVVLSCRRPSPMN
FYRNIMQLLYESVDTMIVQLVLVELGRPRRIAGPRTHNLLLVDSLDALLDIEIHTYTAQS
DTSEYYFIFLQQRDALIPHDMQGVFAYCWRHQLINCNVMTQSSGGQVLLHTYFPYAPGQC
NDSQPTRINMFLGESWKHRDYFPSKLHNLNGCPLIVLARKVSPFLDLDEGQRELRGLEGR
LLQELSRRMNFSIQFSGLQDQLKNRTTWTEKQLLQKLVQERIAHLAIGYVRKRIQYATNL
TPVFPHYSNRVVGCLLLNAHNLTSLEIWSFPFQALTWICLVAGDRLALVLAVYAASLGLP
IDPPERPSLQLLFASWLIFGLIVRSMYSALLFFILRYHLHQRLPGNLQDLTHGDYAAVMG
RTTLQDLREVPSLQDLLGLKSVIVTSEREEEVLRTLDRCTLREGAGSHPLFFGLISQDAL
LHLTQRGHRAGAYHIIPQDVLEQQLAIYLQKHSHLASHLDHLVMSIRSVGLVHHWAGQM
A
SERYFRSRFLYREKRIRQPDLWAVYILTAGLYLLSLVVFICELLASRRAGL
>DmelNmdar2
MMPSRVKLKRGTDGPTPTPTPMPTTMRKHTPIATLNTASCQHNSTTSRRKRILTPPSGPI
SLLLLTVLTLLILDTRSCQGLRLTNGGGSLSKGAAANKEQLNIGLIAPHTNFGKREYLRS
INNAVTGLTKTRGAKLTFLKDYSFEQKNIHFDMMSLTPSPTAILSTLCKEFLRVNVSAIL
YMMNNEQFGHSTASAQYFLQLAGYLGIPVISWNADNSGLERRASQSTLQLQLAPSIEHQS
AAMLSILERYKWHQFSVVTSQIAGHDDFVQAVRERVAEMQEHFKFTILNSIVVTRTSDLM
ELVNSEARVMLLYATQTEAITILRAAEEMKLTGENYVWVVSQSVIEKKDAHSQFPVGMLG
VHFDTSSAALMNEISNAIKIYSYGVEAYLTDPANRDRRLTTQSLSCEDEGRGRWDNGEIF
FKYLRNVSIEGDLNKPNIEFTADGDLRSAELKIMNLRPSANNKNLVWEEIGVWKSWETQK
LDIRDIAWPGNSHAPPQGVPEKFHLKITFLEEAPYINLSPADPVSGKCLMDRGVLCRVAA
DHEMAADIDVGQAHRNESFYQCCSGFCIDLLEKFAEELGFTYELVRVEDGKWGTLENGK
W
NGLIADLVNRKTDMVLTSLMINTEREAVVDFSEPFMETGIAIVVAKRTGIISPTAFLEPF
DTASWMLVGIVAIQAATFMIFLFEWLSPSGYDMKLYLQNTNVTPYRFSLFRTYWLVWAVL

FQAAVHVDSPRGFTSRFMTNVWALFAVVFLAIYTANLAAFMITREEFHEFSGLNDSRLVH
PFSHKPSFKFGTIPYSHTDSTIHKYFNVMHNYMRQYNKTSVADGVAAVLNGNLDSFIYDG
TVLDYLVAQDEDCRLMTVGSWYAMTGYGLAFSRNSKYVQMFNKRLLEFRANGDLERLR
RY
WMTGTCRPGKQEHKSSDPLALEQFLSAFLLLMAGILLAALLLLLEHVYFKYIRKRLAKKD
GGHCCALISLSMGKSLTFRGAVFEATEILKKHRCNDPICDTHLWKVKHELDMSRLRVRQL
EKVMDKHGIKAPQLRLASSSDLLNHHHLKERPPLLGNLSLAASAQDLYRWSYKTEIAEME
TVL
>DmelNmdar1
MAMAEFVFCRPLFGLAIVLLVAPIDAAQRHTASDNPSTYNIGGVLSNSDSEEHFSTTIKH
LNFDQQYVPRKVTYYDKTIRMDKNPIKTVFNVCDKLIENRVYAVVVSHEQTSGDLSPAAV
SYTSGFYSIPVIGISSRDAAFSDKNIHVSFLRTVPPYYHQADVWLEMLSHFAYTKVIIIH
SSDTDGRAILGRFQTTSQTYYDDVDVRATVELIVEFEPKLESFTEHLIDMKTAQSRVYLM
YASTEDAQVIFRDAGEYNMTGEGHVWIVTEQALFSNNTPDGVLGLQLEHAHSDKGHIRD
S
VYVLASAIKEMISNETIAEAPKDCGDSAVNWESGKRLFQYLKSRNITGETGQVAFDDNGD
RIYAGYDVINIREQQKKHVVGKFSYDSMRAKMRMRINDSEIIWPGKQRRKPEGIMIPTHL
RLLTIEEKPFVYVRRMGDDEFRCEPDERPCPLFNNSDATANEFCCRGYCIDLLIELSKRI
NFTYDLALSPDGQFGHYILRNNTGAMTLRKEWTGLIGELVNERADMIVAPLTINPERAEY
IEFSKPFKYQGITILEKKPSRSSTLVSFLQPFSNTLWILVMVSVHVVALVLYLLDRFSPF
GRFKLSHSDSNEEKALNLSSAVWFAWGVLLNSGIGEGTPRSFSARVLGMVWAGFAMIIVA
SYTANLAAFLVLERPKTKLSGINDARLRNTMENLTCATVKGSSVDMYFRRQVELSNMYRT
MEANNYATAEQAIQDVKKGKLMAFIWDSSRLEYEASKDCELVTAGELFGRSGYGIGLQK
G
SPWTDAVTLAILEFHESGFMEKLDKQWIFHGHVQQNCELFEKTPNTLGLKNMAGVFILVG
VGIAGGVGLIIIEVIYKKHQVKKQKRLDIARHAADKWRGTIEKRKTIRASLAMQRQYNVG
LNSTHAPGTISLAVDKRRYPRLGQRLGPERAWPGDAADVLRIRRPYELGNPGQSPKVMAA
NQPGMPMPMLGKTRPQQSVLPPRYSPGYTSDVSHLVV
>AmelIR218
MAYMNHTIRKTINGFEAEVYAMDNDLDWDGIEMRLFLIMAEKLNFTWTIRKPEGNYTYG
KRFNETYWIGGIIQMLVDQKVDIAFASIWMTLDQNKFVTLSMPWYDVYLHFLVPRPHRTT
SFWALKKPFSKKIWCLLLSALLLHSLYTYVRSWIDSKFPKRYRNFLITFIDLIGYLLSSSVPK
TAVPNRVQILLWQTVGWLIIAAYCSSLAARLATWEYESRIDTFKQFVEANLSWGKSGQPPP
FDDYFDLSDPHSAQLRNRYRQIENNTQLEKFIMEGNYAILGKIIETCFVPTDYITTESLKNY
RLMRESLGHFYASFAIQPRLLKPINKMILWLKESGIVIWHLRDVIRRRGNYNFREVFIERDR
YDGSVQVLGLTPLGAGFSLLLVGFFIATLVFCLELKHFVGKMCDLRILINRKHDS
>AmelIR75u
MLSAPLKWLLLRDRSSVDGDWTDERETLKGMAVYPDSEVIVARKRRNGVVEIRSVYRPS
PFHEAIEEDRGNWTIEHGVRMPNLYPSSQRRRDLRRTPLKSCLVMTDPDTINHLTDYENKH
IDPVTKANYPWIMHIANRMNATISFRVTDSWGYRSENGSWSGMTGMLQRREIDIGGTGTF
FIPQRIGVVDYIQLYTHTRACFIFRQPLLSTVSNIFTLPFQRSVWIAIAVFLLLLLVLLCFSTK
WEYRRGASANTARYWQQFNPAEQTVSDNLMVVLGAIAQQGYSYEPYRVPSRIVTLMLLI
AALNLYASYTANIVALLQSTTDSIKTPADLLHSPLKLGAQDVVYSRHFFKSFQDPIRRAIVD
QKIEPKGQNGSWMSVEEGARRVRNELFAFHAERGALYKIMQETYYEEEKCGIMEIDVMN

MLDPLLVIQTRSPYLEIVKNAALLLRETGLKVREDIRLYTNKPKCHERKSFVRIGFTECYFA
LVALGYGTLLSLVVLAIEVVW
>AmelIR68a
MLLVTLFLQFIVLASSKRVLYKLHQCENNEANLKSLAEEIVEEIIEQTNCIIFITDSTYQNLID
IKNIKGSSNVSKYETNLIRKSHKLKRYCETTSIILIAVFEHIPAVTEKSKLYYNKQPNNIIQGL
GIEFELIQIISKAMNFKPKYYIQQNIPLKQKDIEGSNQTDTGLISKVIEENAAFYLGDLHYTL
QNLNYLDLTIPYNIECLTFLTPESLTENSWKLLILPFKQFYTWIALILTLILGSIVFYFLSLSYK
KHISSYKSQNTSIKNETKGLYLFTEIGNSILYTYSMLFQVSLPHLPSPWAVRILIGWWWIYSI
LVAVAYRASMTATLANPVARVTIDTLAQLAKSSMEVGGLNEESKNFFLKSSDLSSQEIGNK
FIIIKHEDEAIEKVANGSFCYYENSYFLQYARVKRQIFEKEKKRNETANNRSSKHNLHIMEE
CIINMPIALGMEKNSPLKPKVDILIRRMIEIGLVKKWLNDVMEWPKIMEIRQEAESEKALV
NLHKLKGAFFAIIFGYLLAFMILIGEILYWKYIVLKDPKFDKYH
>AmelIR93a
MISVLLLVWWINYGSSYNNFPSLITSNATMAVIIDKGFFSNKDEYQNATKVIQDLITDAVKK
EMNLGSISIRVFRDMNVNFKDYTILLSVATCYLTWRLHEVAQKEELTHFAITDPDCPRIPDT
DGITVPSIVPGEELSQIFLDLRMTDILSWNVINILHDDTFGDKATSSNDNVTILLSNANTCSR
LVSDRDTISRVLKAISNKLPNKRMNLISRSIFSLRHGNTGSGRKSSVKKMLNDFHVEQLGH
CFLVIATVDMVADVMSVANSLNMVHPGSQWLYVITNSVSGNLINTSFINLLAEGGNVAFM
YNATNLDGFYKIKLKCYMKDLIEALAKALEYSLKNEIELFKRMNEDEFEMIRLTKSKKRA
ELLKNVRIHLSRNTSASNSVCEQCLLWRFFSSITWGNFFSHDRNMAHLLDIGTWTPIIGVN
LTDVIFPHIVHGFRGINLPIATYHNPPWQIISMSKTGKKLYEGLIFDAINYLSMKLNFTYTVI
MLETSQISRSWNTSQFAKLGEKIKEMTMSTTKKVPLEIIDLVRQKKVLLAACALTVNECG
NTTFNYTVPIFVQTYSFLTAKPSQLSRVLLFASPFTKETWACLAVSIIIMGPILYLIHKYSPYS
TKASGLNSSWQCVWYVYGALLQQGGMYLPQNDSARILIGMWWLVVMVLVATYSGSLVA
FLTFPRMDTSILSVEDLIAHKDSISWGFPNGSFLEMYLQNAEEPKYHVLFSRAERHNDTEE
ERLVERVKEGKHALIDWRSSLRFLMRKDFLLTGSCHFSLSMDEFLDEPIAMIIPYGSPYLSV
INAELHRMLESGLMNKWITEKMPMKDKCWEAPGSNQMVNKRKVNVTDMQGIFFVLFIG
ITLAFFFLFCEFYCHRRKIAKERKLIHPFVS
>AmelIR76b
MAINKDNEEGNSIPSQITVTSWNDMPFSGIVQKNGKWIGQGYAFYIFDLISSKLNFTYTIIPP
KEHILGNESSGILGLLYEKKVDIAVAFLPMLPEMRRYCSFSTLLDETKLTAVMKRPQESATS
SGLLAPFEKTVWLLVLTSLIFVGPIIYLFANMRAKLWHDPTSENFSLSSCFWFVYSSLLKQG
TNIIALTDSTRMLFATWWIFILILTSFYTANLTAFLTRPQFTLSISSLEDIVHKEYNWITYKGRI
VDFLLSQNQQNDLSLLNISKQQGKGIFKYYEPSRPILELVSTKRLFLEETHYLESLIFKDYV
NKTRDHLEHNLRCTYVIMPGNILVTSRAFGFSHGSTIEKHINKMLLRLRETGITQFKKKED
LPLAEICPVDLRSTERQLRNTDLLLTYKVVIGGYTIATIIFLFELIYACISYRLQNRKRQFING
RSYYVITNPVGDRKLIPIRTPSAFLFQYTT
>AmelIR2
MAARWLLLALLAAHAAALPDIIRIGGLFHPSDDKQEVAFRYAVEKINANRDILPKSRLSAQI
EQIKPQDSFHASKRVCHLLRSGVAAIFGPQSAHTASHVQSICDTMEIPHLETRWDYRLRRQ
SCLVNLYPHPTTLSKAYVDLVKAWGWKSFTIIYENNEGLVRLQELLKAHGPSEFPITVRQL
SEGSEYRPLLKQIKNSAESHIVLDCSTERIYDVLKQAQQIGMMSDYHSYLITSLDLHSVDL
EEFKHGGTNITAFRLVDPEKQDIQKVVQDWIYGEKQLLKSIDDTIVRERMTETSLMYDAV
HLFAKALHDLDTSQQIDIKPLSCESTDTWPHGYSLINYMKIVEMTGLTGTIKFDHQGFRSD

FILDIIELNNKEGLKKIGTWNSTKGINFTRSYKEEYTQIVENLQNKTFIVTTILSAPYCMRKV
SSNKLVGNAQFEGYSVDLIYEISKILGFNYTFRLVPDERYGSYNKKTKEWDGMMKELLDQ
KADLAIADLTITYDREQAVDFTMPFMNLGISILYRKPIKQPPNLFSFLSPLSLDVWIYMATA
YLGVSVLLFILARFTPYEWYNPHPCNKNPDHLENRFKLLNCMWFTIGSLMRQGSDILPKA
VSTRIVAGMWWFFTLIMISSYTANLAAFLTVERMDSPIESAEDLAKQTKIKYGALKGGSTA
AFFRDSNFSTYQRMWSFMDSAKPSVFTTSNVEGMERVIKGKGSYAFLMESTSIEYVIERNC
DLTQIGGLLDSKGYGIAMPPNSPYRTAISGAILKLQEEGKLHLLKTRWWKEKRGGGSCRD
DISKSSSTANELGLANVGGVFVVLMGGMGIACVIAVCEFVWKSRKVAIEERKQKSSGKPIC
EAFTDLH
>AmelIR25a
MTGAGFNEKYMSAPPRKLRYRVIINDEANKVANQSITTALETIKENYPNHLGNVWSVQVN
ESDINNTLDRVCNNWDSAVEKGGAEVPDLVIDTTTAGLAAKISNSFTAALGIPTLSAQYGQ
VGDLQYWRKLSADQQDYLIQVMPPTDLIPEVIRQLSIQLNITNAAILYDYNFVMDHKYKS
LLLNVPTRHVINETSQQIIEMKRQLLRLRDLDIVNYFILGNENTISIALEAADALNFTDKKY
GWFLLTPDINIWPRCECRDISVLFMKPEFDKRNNSDSVEFSLPKPILLSAFYYDMIRLAVLA
MKSALDDGEWPMEPRHITCDEYNNTNTPERKLNFFGKLKDAYKNITPTYAGIKWGSRNG
EHQAKFVMSVHLVTIKDGVVSNTVDSGSWNASISSPLQLTNNDVMNTTAVKSYRVVTVIH
PPFVMYNEEKNEYYGFCIDLLNEIKKTVGFQYEIRETDDKKYGSLNLDGSWDGMMRELIE
KRADIALGSLWVTAERERVVDFTVPYYDLVGLSIMMLKTKTTTSLFKFLTVLENEVWFCIL
AAYLFTSVLLWIFDRWSPYSYQNNREKYKNDDEKREFNLRECFWFCMTSLTPQGGGEAP
KNLSGRLVAATWWLFGFIIIASYTANLAAFLTVSRLEIPIETLEDLSKQYKIQYAPVINSSAYI
YFKRMANIEWKFYEIWKEMSLNDSLSDVERANLAVWDYPVSDKYTKMLQAMEEAGFPA
STEEALRRVRRLDSNNEFAYIEDSTTIKYLTMTNCDLIQVGEDFSRKPYAIAVQRGSPLKDQ
FNNAILILLNKRKLEKLKDKWWKKNPNKKDCDAENSQSDGISIHNIGGVFVVIFLGIIFACF
TLAFEYWYYRHRNAQDTASIWAAPTLSIPSGPLWPTDTTETIFTLSTSLYGYATDTPYQESI
VDDSPLIPWDIDLISIEDYADEYSNKNKSGDRSDM
>AmelIR8a
MSTTLVAVCALLLTFLGVSICQISMNMLIVIEEPDKSILNILNEALPQAEKNYGNDIISVHIST
IEVERSNTDASFKKVCAALFKGISIVLDMTWTGWDTLRNKANENGIIYKRGDSNINPYIQA
IDDLLMLKNATDVALIFEDERELNQSLYYLIGNSILRLIVIDEFTEKTVSKIKSMRPSPSYYAI
YASTAKMEDYFRTAVQGGLVKRNGIWKLIFTDNNYKDFKYINGDLQLNVSITVLWMKMD
VCCRLIGESLCNCPSNVKIFSNYFKRLVGLIVSLMSELQASGVSVEPKSVKCSSNANQSSN
VTIEAFNKNIVAKLGGNDTFEYWPEKGMITYKAEIELKILENGLLEPLATWTRNGKIKEAE
NKKILPAKRFFRIGITPVIKFLPWIIPKIDPATGKVMKNENGNDMWDGYCIDFVKKLSEEM
QFDYDLIIPEDRQFGKKLPNGQWNGLIGDLAKGETDIIVAALTMTSEREEVIDFVAPYFEQS
GLLIVMRKPVRKPSLFKFMTVLKVEVWLSIVGALTLTGIMIWILDKYSPYSARNNKQLYPY
PCREFTLKESFWFALTSFTPQGGGEAPKALSSRILVAAYWLFVVLMLATFTANLAAFLTVE
RMQSPVQSLEQLARQSRINYTVVANSSQHQYFINMKNAEDKLYTVWKEITLNNTSDEVEY
RVWDYPIKEQYGHILQAITQVGPVANSVEGFRKVIESENAEFAFIHDSSEIRYEVTKNCNLT
EVGEVFAEQPYAIAVQQGSHLQEEISRKILDLQKDRYFEMLASKYWNQTQKAQCLNSDDN
EGITLESLGGVFIATLFGLALAMITLAGEVFYYRKRNTETEKSTKDKKRKVKNKIIQNLTK
MSLQMKPAPINPFFEKTNNPPRVSHISVYPRNLPFKE
>AmelIR75f.1
NFKLAKTFNNMYTTYISMLNSTVELRLDVKKILRAEYHRLGIFLDSRCDRSRYRRILIDAT

KYSMYDEMHKWLILGSNLSHVLEILNDETFSVSTDVIIAVPSADNYILYDVYNPCKDRGGS
MNVTYFGMWNFKTGLNVNLNQSKFARRSNLHGMKLKVGIVVNFKPENMSLHDMMLQY
SMKSKYGRSKFLYILLQHMSDIFNFTMKIVQINAQRRFDNSGPIFAAFKKKLIDFSANPVA
MKVDRLHNGDIIRPIWPIRSCFMFRTISSTKVKPDQFLKPLSVKVWYVILAMIGVVTTILIIF
LKLENIGTPTEIYGLSVLLTIGALSQQGSAFIPTRCASRIALLQVLFFSLLILNYYSASVVSSR
LKNKGEKMNDSLINLAKSNLKVAVEPTPYIRSFLQVSDKEVKYFYDNCWTKIPELYKYLP
LEEGLNRVAEGSLAYHTMTDSAYPYIEHTFNYRSICELTEVHLFRAVLAFYARHHSPFTEL
MKVGLTKIHNVGIQKRELIRWTARKPFCPNNLLIAEPLSIHEAAPIFIFLCISIILSILICIIE
>AmelIR75f.2
YFLYKKVRNVVGFSCGDTIGDFNLLKTLSTTGIYTIIRESSVKIDFQRFMKSETWTVGVVID
LRCHNETAASSKYRMYDYSYNWLILGSNYNHSISLLNDTAYNIITDVALAISNRNGFHLYD
VFNHCKYRGGILNVTELGTWHIYSGLKIFLTQPLIIRRANMHGMRLKISGVVSLPKDMRLE
DYMQDINTRSLDSMHKFVHAMILHIGELFNFRHSVHGLIFEFLRSNYIDFASNPRIMVSERL
DYASLIGAAWPIKPCFMLLSTSTNKIKLEIFLKPFTRQTWYVFAAFGIFFIFIMKIIMNREDV
GKKEKYSGAIILSVGILAQQGANFLPKRLPSRIALFQITVHGWIMYNYYSASIVSARLSEPL
DMMEDSVTVLADSNLKIAAEAVPYLNYFLYNLNWESDYFRKKRWDPLPESKRYLPIEEGI
RQVGQGILAYHTDPNTAYPYVERMFDSNKICELTEIHLFKQSLMGMYASHNGQFIEIAKIG
LTKMFNTGLRNRQIKYWSSRKPECQLDTLSTRSITIYEIAPALILLAFGILVAGIICIMENIIYN
RFMKRKSETKES
>AmelIR75f.3
MVYFDIDDYQILKLLNEVGIGVSITQFTSIINIPQFLHTTYWNLGIFVDLECLVSDENITSTY
YMFDHLHQWLILEKNMTHILQLLNDNMFSIITDVTIAISKDNDYILYDVYNHCKNYGGLL
NITKLGTWTKNNGLQIILETNKFSRRWNYHRMKIKVAGLVIKRPKNQSLIDYLQEENLYEH
TDNWSKFGYAIMKHIKQLFNFTFELIELNHWEKNDSNGPLIAGLKNGIYDLGYFPSILTKE
RFNYADVILQVWPIRTCFMFLTVPSLKVDMDIIFRPFARNVWYMILILIVAIILGLWIIFKLEE
NDSAYGSTILIIIAALCQQGLPFFNNQFSSRIAFLQTMIFGLLVYNYYSAAIVSSRLNAPLDK
MNDSLYSLVNSRMKLAAYKDIYFNILLHVIQYFKKYWEKIPEKKRYLSIQDGLKKMTTAK
FAYHADPMNVYPFIERVFDKQMICQLTEVHLLRPSSLGLWSTRHSQFQEITKIGLIRISTSGI
RKREVIRWTYRKPYCDKDKHYVSSITIHETIPILLVLCFGIILSIVICFIENIIFHTIRKKQRQIK
E
>NvitIR68a
MYFLIVLIICLGTSLSVTDNRRLIYPASNKQLQTLVKLLIEEVAENSRCIVSMVDTYYRRKV
DISQIKANKFLPTYRVLIRENEEFSPPRRRLLRILKESKHLGCDVYLIMMANGLQVASLLRY
AEEERLMNVQGKFLFVYDFRIFHVEMLYLWNRIINVIFIRRYVEFKRRSSNRQLQKYEWY
DLNTIPFPARKKGLIVTRYIDTWYQNRFRYGINHFTAKTDDLRRQKLQVAVFEHVPAVTED
AQAYYKSQKDVGSNSKPLGIEFEMILIIANALNFKPYFYQPDNIQTERWGDSKNDTYTGLF
GEAKEGKAVFYLGDLHYTSRHIQILDLSWPYNTECLTFLTLESLTENSWKLLILPFRLNTWL
AVLFTLVFACATSFVFSRFYMRHVNVGENNDSDARKVFSKSKTMKVLEKRPVQAEEWKG
LYLFTDPQNSVLYTYSMLLQVSLPSLPRAWSLRVFIGWWWIFSILIAVTYRASMTATLANAI
DRVTIDTIPELGKSNVAVGSWNDETREFFINSSDPYLQKLSRRYVVTKDEQSALAAVANGT
LCYYENVYVLQRERVKRQILEDELQKNGSQGKHKFQDHNLHIMEECVVNMPISLGMDK
HSPLKHHVDKLVKRIIEAGFVEKWLSDITQQSKILELRGEGIADKALIDLDKLQGAVVALGI
GYLFSLLALAAETWHWRYIVMRHPNFNKY
>NvitIR75u.2

MSLVLLLLLCYLSTLLSAPATSSKTSDTGRLARLLEYAGPLYSGAFSMVTSFSCSDVDGRFE
LLRDLSRIQVGSNVLDLEKSVDRLSEIAWEKGYAGQNEIPSDAHRVLFTLDLDCPKIDDFIR
KADREYMFAAPFKWLLTQRSNDTDDLVSRFAEVGAYPDSEVTVWQKETGQLLSIYRTNG
NNDHLIEDRGSWMEEDRRMLVGDTNVTSRRRKNLGGIGLKSCLVITDPNTIHHLDDYHD
KHIDTITKCNYPWVLILMNMLNATVTFEPVGSWGYKGPNGSWDGMIGMLQRGEIDFGGT
GCFLTPERMGAIQYISLPTPTRNRFIFRRPPLSSVSNLFKLPFGNSVWFGSCALVIILITMLYP
AMKHEWMQFNEVEKAREPIPPNLSDDLLVIVGAVSQQGSWYEPRSVSTRIIVLFGLLAAL
NLYAAYTANIVALLQSTTGSIKTLQDLLDSPLMLAAHDNVYNRYYFKSFKDPIRRTIFEERI
EPRTSKKTNWLTIEDGIEKLRGGYFAFHVELGPAYKLIQERYEEDEKCGFQEIDYLNVFYA
HLVVRRRSPYLELLRVGAMRLYETGMRTKDINRLYTRKPECGGSSSRRFLSVGLAECYGA
FSTLGYGLALALGVLIAEVVSTKL
>NvitIR75u.1
MWRFCALIFISLGILVDCHEIDELISNLVAEASAGLFFPAAAVENAVEFSKILSKNHVLNNQL
NYDDFEENIVQYPVHQTTIFLDYDCPRASEALRKANNSGLFSAPIKWLILQDLRNAPNENC
TETDCILNIFHDYAMFPDSEILIFERTSKNYVKILSVYRPSPVRDMMIEDRGFWSKEDGLK
MKDAHVSSRRRRNLYMTPLKSSIVVTNPDTLNHLTDYRDEHVDTITKCNYVWLHHLVAA
MNATVTYNVVNSWGYRDKNGSWNGMMGMLSRKEIDIGGTSMFLVGDRWADAHYIPLS
TPTRAAFIFRQPPLSFVSNLFTLPFRPSVWIAIGILLIIIFLSLVISTKWEWVTKEVDSSGTPPP
SLSDNLLLVVGAIAQQGFGRNPRTVPSRIVLLMLLLAALNLYASYSANIVALLQSTTTSIQT
LRDLLDSPIKCGAQDIVYNRYYFNLEKDPVRRGIIDHKIEPKGGKSNWMSADDGIKNIRQG
FFAFFMETGPGYKIIQETFEEDEKCGFREMYFIEHFDPMFTIVKQSPYVEIMRVNSLKIEESG
LKSREMSKFYTKRPPCNGLSKFISVGLNECYFAFYTMGYGALIAFVVLFAEFAWMKRIHC
HREFTCKQETEPA
>NvitIR21a
MRFLVSWSFLVLLQLTVSLNQRHFRKLSKSHEARTESLTILLRTIANDYFSNCTLNVIYDSA
YELIYPINFSLYFKDNSLPIIQETVDFSSGGRVREKIVEKCTNYFVFLGDLQAIKYIVEQDTK
SKIVIISTETPWEVKDFLKSHLSKNYLNLLIMAYSASSRNDGSYLLYTHKLYAEGSGSTQPM
LLSSWINNNLTIRDVNLFPEKLTKGFMGHRLLVSAAENPPFTIKKSLWGSDDSNWDGIEIRL
LKLSAKYLNFTIEFTDARTSAYSPIDAVKRDVLLGLTSAAVGGIYLTQELSESFDATLPHAE
DCAAFISLASTAIPKYRAILGPFQLSVWLLLCAAYFVLIIPLSFNSNYTILSLFKHPSGLNNM
FWFVFSTYTNSFVVENPLLNYGIAKNSITILLGIYWIFTIIVTACYTGSIVAFITLPVYPSAIES
AAEFLAYRYRIGTLDHNGWENWFNVNTTDDPLLQSLFRKMEYLPTLLEGVHNASRAYFW
PYAFLASKTSLEYIIQTDFAPTWTTKRTLMHVSDECFVRYNVVQLFPAKSLYTKSMNGFVA
RVTETGLLDKIINDIEWEMQRVAMMTKKQITKSISKKIKVEDRELTVEDTQGMFLILGSGF
LMAMLALALESLYSYFKKRNIKKDSVSEMEGTDWMTESQVMGSWIYGKDAFPGMRQRP
ARGSV
>NvitIR93a
MLLALLVLLAGWIEIGTGYNDFPSLMTANATMAVIVEKGFFKSADNYRHTLDEISDVANA
VIRKNMEISGIALHVFGDADVNLARDYTVLLSVASCQTTWHLFKRAQKEKLVYLAVTDPD
CPRLPEDAGISLPLTNPGEELPQIFLDLRTTGSLSWPKVNLIHDDTFARDTISRVVKALSLEL
PDKRVSLSAQALFSTRFEKNENAMRQRVHRILSNYHVDQLGSCFMVVVTVDMVSIVMEV
AKSLRLVHPGSQWLYVISDAAGREAKVTSFAELLAEGENVAFVHNATKHVANCNMGLMC
HVKELVRALAISLENSLLNELELYDRVTEEEFEVVRLSKAERKQEIVKSVNRELSYARAHT
SSCGKCVNWRFSSAITWGTSFASSEEKQRRESGEKRRRENSKRHSEDDLGEKSLGLGELL

DAGTWSPGPGVNMSEPLFPHVEHGFRGRSLPVSTFHNPPWQIIKYSNTGAQEYGGLIFDV
LNYLSLKLNFTYTVRLASSPAAEAPTRLPSAGDSSKSMDLAAMSVAQKVPQEVVELVRSK
QVFIAASAFTVGKNSGGLNFTAAIVMQNYALLSAKPKPLSRALLFTAPYTNETWACLTSVL
IVIGPILYLTVKLSPRPRDIDNSLSLSTTWQCSWYVYGALLQQGGMSLPKADSARLVIGTW
WLVVMIVVATYSGNLIAFLTFPRIDAPIDNVDDLLARSDAFHWSFPNGSALESYLIAAVND
DPKYKQLLDGAERQDPSKPKQILDRVKAGNQVLIDWRISLAFLMREDLIDTGGCHFHVSA
EDFMHENMAMIISGDSPYLPLINDAIERMHESGLMKKWITEKMPMKDKCWEIAKTNQEA
TNHKVDMGDMQGIFFVLAIGFVIAAIAIGVEFAWHKRKEAFERSLIRPFVS
>NvitIR76b
MGSQDEPPPFPSHIKITTYNDWPFSRYREENGTIIGEGYAFELLKLLIRKFNFTYTIVPPREDI
IGNEERGMLQQIYDGEVDMAVAFIPILSSFRNICDYSAPLDEMDTTFLLKRPGTSATGSGLT
APFSTRVWYLILISLLVVGPTIYLIIYLRGKFARDEKAEKFTFLTCMWFVYGALLKQGTTAS
PMGDSTRLVFATWWIFITILTSFYTANLTAFLTLSRFTLGVNSLDELVYGRYSWVIVNGRSIH
TLLPIESDDTRMLVKSKSWGYGYVTREYNMSYNSILKKVKDGRVFILERTLAQMAIFEDY
RNKTRDAMDEERKCTYVISESNVLARPRGFAFPVGSTIKQHVNSEMIPAVEGGLVKHFKLE
KLPQAQICPLNLKSKERRLKVSDLMMTYKVVFAGMGVGVIIFLMELFTIFIRWVARHEAF
RNCCRRNEVKGPSEPEDPWDYPKKTDTNFWIRTPPPVYQMNEEVSHKTYSINGRDYYIVK
EETGDQRLIPIRTPSAYLFQYIH
>NvitNMDAR1
YFIFLAIIIRQYAKGKHSQTFNNPGYFKIGGMLSDGDSEAFFNETIDNLNFASQYVNKGVTY
XHVVIAMDSNPIRTALNVCKFLIAQKVYAIIVSHPPVGELSPAAVSYTSGFYHIPVIGISSRDS
AFSDKNIHVSFLRTVPPYSHQADVWVELLKYFNYMKVIFIHSSDTDGRSLLGRFQSTSQIL
EDDMEVKIQVENVIEFEPGLSNFKSELLLLKSAQSRVYLMYASAEDAEIIFKNAAELNMTG
AGYVWLVTEQALNSNNVPEGVLGLKLINVTNEKSHIKDSLVSLQLERKYRDSQCPRSQFE
ALSNQSCLESSFSIGRLFEYVRQQTLENGATGRVAFDDNGDRIYAEYDVVNSRLSNKFVSV
GQYYYSSVSIDIFMGAAAVNSGLDASSQQNVATTSDRPLNRGYGEDERCSAQYAQSYEVS
AQNSIIQNDMLKINCCRGYCVDLLKELSKTINFTYSLSLSPDGQFGNYIYKNNSTAKKEWT
GLIGEIVSERADMIVAPLTINPERAEFIEFSKPFKYQGITILEKKDQAFKISLSAPFRFADISSC
LCLELLVWSCVFAFRSSFDPSNNLCGVHSGTPRSFSARVLGMVWAGFAMIIVASYTANLAA
FLVLERPKTKLTGINDARLRNTMENLTCATVKGSAVDMYFRRQVELSNMYRTMEANNYN
TAEEAIRDVKIGKLMAFIWDSSRLEFEAAQDCELVTAGELFGRSGYGIGMQKRSPWVDAV
TLAVLDFHESTGFLVSNLHLSIIHLSKRFKFVIDRLSYIIIESEWYRYVFINVHCPTEDKEEEA
KDLYYETLEQMRKTMRTSDPRPIHSNGITDAGTVSLKVDTVQKTSKINRSPGRAWPGDND
LQQMCDYYENQTQIPTDSKYYVEASNLFV
>NvitIR8a
MMLKNTTDAALIFQNEKELNQTLYYLIGNSIIRLVVIDYLSPQTVERIGNMRPLPSYYATYS
DTRHMEELFKTAIDGGLVKRDGVWYLVFTDYNYAEFSYFKEASALNVTVNVLTMKEDVC
CHLMYTNPPCSCPPNFQIFDHYFRRLINLIVETLSELQSANQLQEPRSGQCTAKNSSGSAAG
NATLSDFDKKILAKIQKNETFEYVEKLTLITYRAAAELKVLSNGELVANATWSRDTGIVPLP
NRTIQAARRYFRIGTTEAIPWTIKKRDPQTNEPLRKPDGSLVWEGYCVELIQKLADMMDF
DYDLVLPEDGEFGQKVNGLWNGLVGDLAKGQTDIAVAALTMTSEREEVIDFVAPYFEQSG
ILIGKAIYQAKPVRKASLFKFMTVLRLEVWLSIVGALTLTGIMIWILDKYSPYSARNNKHM
YPYPCREFTLKESFWFALTSFTPQGGGEAPKALSSRTLVAAYWLFVVLMLATFTANLAAFL
TVERMQSPVQSLEQLARQSRINYTVLDNSTIHQYFKNMKMAEEKLYQVWKEITLNSTSD

QVEYRVWDYPIKEQYGHILQAITQVGTVKSSEEGFQKVIDSENAEFAFIHDSSEIKYKVTQ
NCNLTEVGEVFAEQPYAIAVQQGSHLQEEISRRILDLQKDRYFESLSSKYWNQSLKGSCPN
SDDNEGITLESLGGVFIATLFGLALAMVTLAGEVIYYRRRNAQDEQNQQKHRESIGSTSKR
KTRATADEAEPYDYVPETRPLYDKLLGVSLPAYMITKSAQEAEFDEYVEKAKF
>NvitIR25a
MYRRGCSKGLSLLLIGKLVLLVGGQQTTDTAANRPVNVFVINDANNDVANKSVTNSLKA
LKEKSPDKLGQVYVAQINVSDSDQSLDAICSLWQSSIRENEADAPDFVLDTTTYGIGAESV
NRFTALLGIPTLSAQFGQEGDLLGWRDISEEQKRYLVQVMNPADLMPEVIRQQCSNFNISN
AAILFDENFVMDHKYKSLLLNVPTRHVIVPAEPAGAPLQKQISKLRDLDIVNFFILGSESTIS
SALIEANNLNFTGHKYGWFGITLNEEFQAQCQDCRNISLLLFKPKAESSQQLSELTSKGSLP
KPVISSAFYYDLTKLGVLAMKSALMSGEWHRPRFITCDEYNENATLPARNLNLRQRLEQV
ANSSGFTRTYASFAWGRNGVSRAKFGVNGLLIRIRDSKLISSDPVETWEAGVDSQLKVLDE
NKAGNHTAVTSYRVVTVIKPPFVMYDNETGNWTGYCIDLLDEIREHVKFEYEIREVDDKE
YGNMDEDGNWNGMVRELKEKKADIALGALAVMAERENVIDYTVPYYDLVGISILRKKP
KTATSLFKFLTVLESDVWLCILGAYFFTSLLMWIFDKFSPYSYQNNMEKYKDDDEKRLFT
MKECLWFCMTSLTPQGGGEAPKNLSGRLVAATWWLFGFIIIASYTANLAAFLTVSRLDAPV
ESLEDLSKQYKIQYAPILNSSEYRYFERMANIEKKFYEIWKDMSLNDSLSDVERAKLAVW
DYPVSDKYTKMFQTMQDAGFPNDMDEALRRVREGKPTEFAFIGDATDIKYLTMTDCTFM
QIGEEFSRKPYAIAVQQGSPLKDQFNNAILMMLNRRKLEKLKDTWWNKNPKRKRCNKAE
DQSDGISIQNIGGVFIVIFVGIGLACVTLIFEYFYYRRRPQIKKRHQESRTDKTKSVQSVKS
MKFNLRPAPTQSLENTNYRSRF
>NvitIR25b
MNATAKPTYRVITIPKPPFVIYDPDSNWYGGFLVDLLNEIARRLNFRYEIEMQNESEYGFM
DDQGNWNGLMRDLKEGKADIGLAAVSVMSERMKVVDFTEPIYKPTGISVLMQKPIPKTD
FYRFLTVLELDVWLCIIGAYIFTSLLLWIFDTWSPYSYRNCKAKYKDDTEKRIFGCKESLW
FCLTSLTPQGGGEAPKNLSGRLVAATWWLFGFIIIASYTANLAAFLTISKFEKTIETFDDLISQ
YKYSYTCIQNSSTNRYFQRMNDIEYVCYEKWKDMTLNDSLSPYERAQLAVWEYPLSDKF
IKIYSAISHHGMVASLQDGLDKFNSTDSRFALITEASDVQYQAMIDCSVKEIGPEFSKKPYA
IVLQKNSPLTKQFNRIIYNMKNDNWLEALTDKWWKYNPLRQRCHDKDEMTNGIIFENIGG
VFVLIGVGILSAFSTLVYEYFYFKCLQDKFERIFEHKLKSIFRRQKNFARSISVKP
>NvitIR64a
LSINAELTLATTATGGAHRYELYDVYNPSYRHGGKLNFTRMGYWDRDEGLRIHMTQYKY
KRRGDLRGMVLNFSIVVDFPPANVDMATYLTTPIQKHLDTMHRYNYALTLQLRDYYNFT
MNLMRGGTWGYLINGTFNGIVGDMIKGIVDVGATPFQFKPERMDVMDYTVQAYMARAA
VIFRHPKRQTLNNSFLEPFSKQVWWLTAVVGLINWILLYLTIKMEQRYVGNVQGSTLFTQP
ESETFLITSAAICQQVILRHPKKNDLSNQFFKPFSREIWMLTTVVAIVNWLLLYLTVKVEQH
YNKNYEKKNLGTLDTHPASETALITTAALCQQGLSDGPSLYSGRIVFISLFLWALMLYQFYS
ASVVGSLLAVPPRFITTPQALLESTMELGAEDIAYNYDFFAXYNDCLLIELTNIVGLLTQRFS
QQTHPDPIVLELNKKKLMVNKHRKKAPYFTPEEGLKKVQKGGFAFQVDIATAYKIIEDTFT
QAQICDLTEIELLPPRYVTIVTAKHSPFKKMVIYGLRQIVERGLTNRLFRIWHHRRPSCPESF
RSQPVAVELQEFSPAMFMIVGGLILATIVMAGEHLY

Legends of Supplemental Figures

Figure S1. Cluster of orthologous groups (COG) function classification of *A. gifuensis* antenna transcripts. In total, 11086 of the 29302 unigenes with nonredundant database hits were grouped into 25 COG classifications.

Figure S2. Heat-map of relative expression values for AgifORs. Estimation of abundance values determined by read mapping.

Figure S3. Heat-map of relative expression values for AgifGRs. Estimation of abundance values determined by read mapping.

Figure S4. Heat-map of relative expression values for AgifIRs. Estimation of abundance values determined by read mapping.

Figure S1

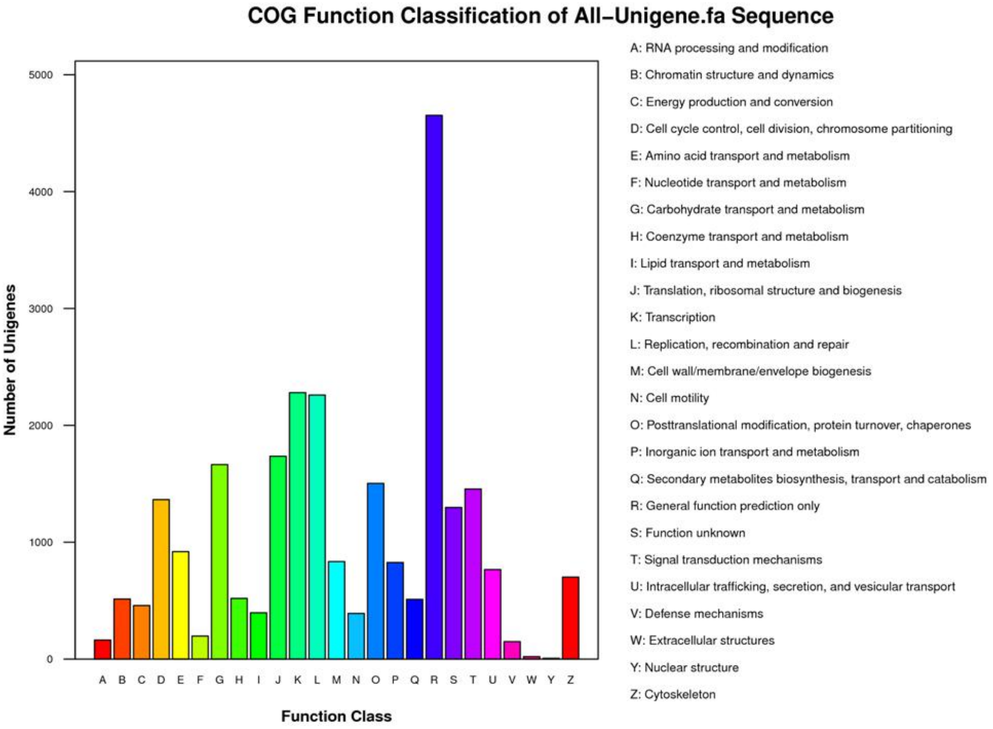
COG Function Classification of All−Unigene.fa Sequence
Number of Unigenes
Function Class
0
1000
2000
3000
4000
5000
A B C D E F G H I J K L M N O P Q R S T U V W Y Z
A: RNA processing and modification
B: Chromatin structure and dynamics
C: Energy production and conversion
D: Cell cycle control, cell division, chromosome partitioning
E: Amino acid transport and metabolism
F: Nucleotide transport and metabolism
G: Carbohydrate transport and metabolism
H: Coenzyme transport and metabolism
I: Lipid transport and metabolism
J: Translation, ribosomal structure and biogenesis
K: Transcription
L: Replication, recombination and repair
M: Cell wall/membrane/envelope biogenesis
N: Cell motility
O: Posttranslational modification, protein turnover, chaperones
P: Inorganic ion transport and metabolism
Q: Secondary metabolites biosynthesis, transport and catabolism
R: General function prediction only
S: Function unknown
T: Signal transduction mechanisms
U: Intracellular trafficking, secretion, and vesicular transport
V: Defense mechanisms
W: Extracellular structures
Y: Nuclear structure
Z: Cytoskeleton

Figure S2

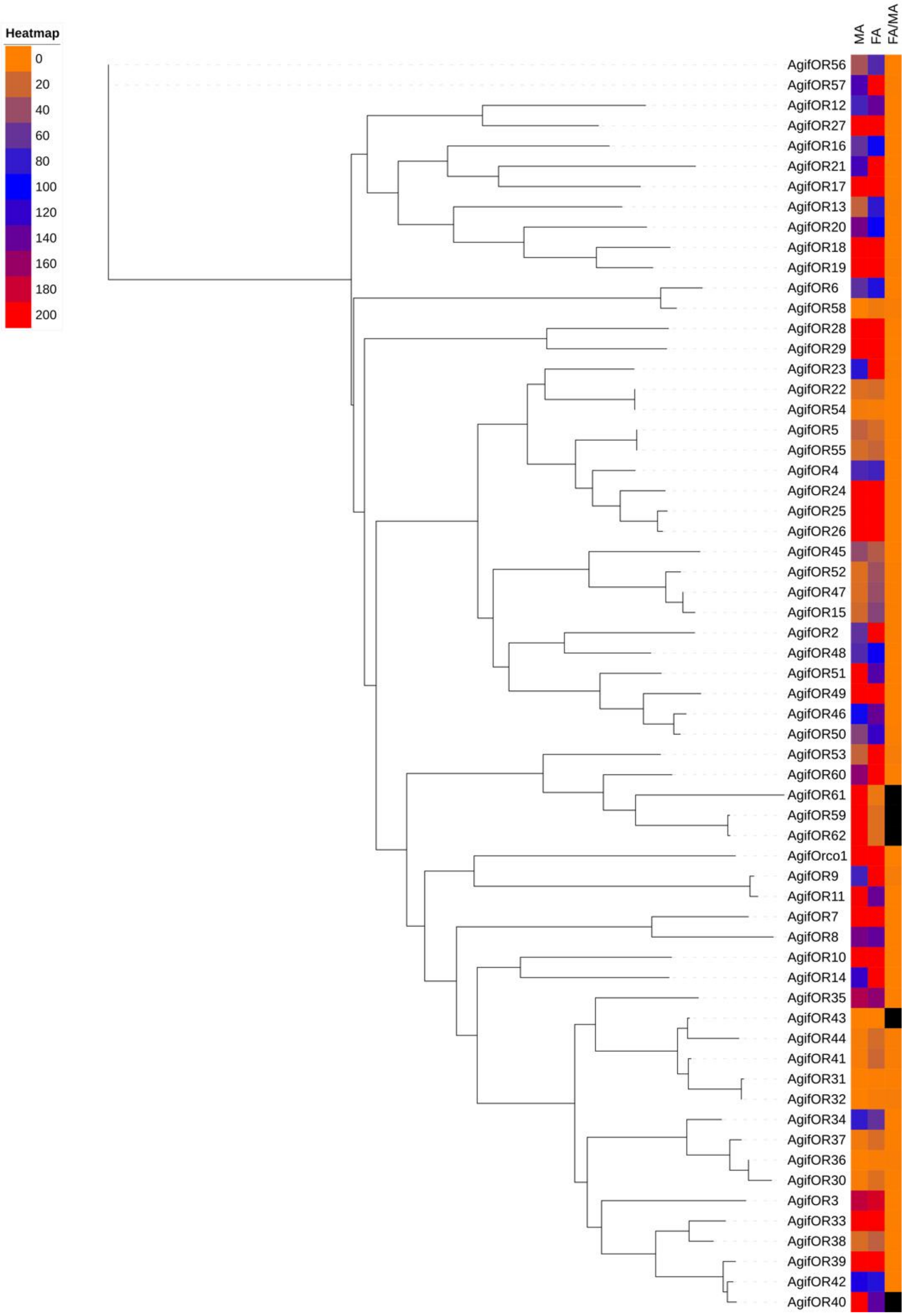
Heatmap
0
20
40
60
80
100
120
140
160
180
200
MA
FA
FA/MA
AgifOR56
AgifOR57
AgifOR12
AgifOR27
AgifOR16
AgifOR21
AgifOR17
AgifOR13
AgifOR20
AgifOR18
AgifOR19
AgifOR6
AgifOR58
AgifOR28
AgifOR29
AgifOR23
AgifOR22
AgifOR54
AgifOR5
AgifOR55
AgifOR4
AgifOR24
AgifOR25
AgifOR26
AgifOR45
AgifOR52
AgifOR47
AgifOR15
AgifOR2
AgifOR48
AgifOR51
AgifOR49
AgifOR46
AgifOR50
AgifOR53
AgifOR60
AgifOR61
AgifOR59
AgifOR62
AgifOrco1
AgifOR9
AgifOR11
AgifOR7
AgifOR8
AgifOR10
AgifOR14
AgifOR35
AgifOR43
AgifOR44
AgifOR41
AgifOR31
AgifOR32
AgifOR34
AgifOR37
AgifOR36
AgifOR30
AgifOR3
AgifOR33
AgifOR38
AgifOR39
AgifOR42
AgifOR40

5862

5863

Figure S3

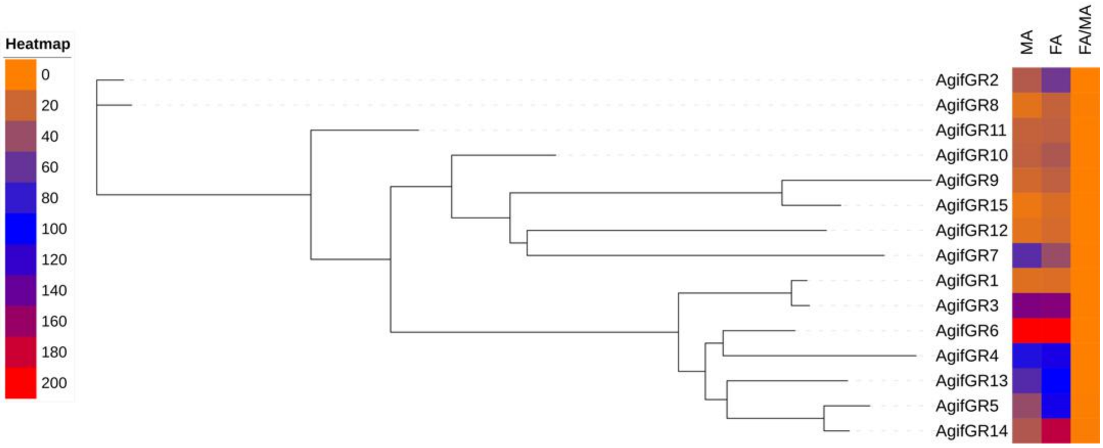
Heatmap
0
20
40
60
80
100
120
140
160
180
200
MA
FA
FA/MA
AgifGR2
AgifGR8
AgifGR11
AgifGR10
AgifGR9
AgifGR15
AgifGR12
AgifGR7
AgifGR1
AgifGR3
AgifGR6
AgifGR4
AgifGR13
AgifGR5
AgifGR14

Figure S4

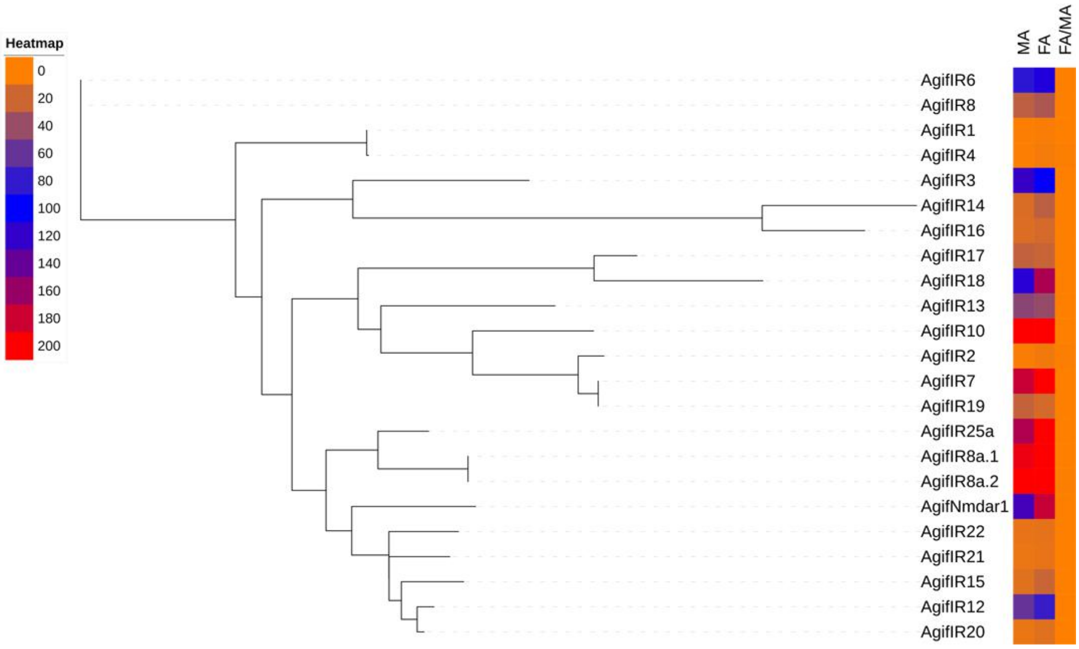
Heatmap
0
20
40
60
80
100
120
140
160
180
200
MA
FA
FA/MA
AgifIR6
AgifIR8
AgifIR1
AgifIR4
AgifIR3
AgifIR14
AgifIR16
AgifIR17
AgifIR18
AgifIR13
AgifIR10
AgifIR2
AgifIR7
AgifIR19
AgifIR25a
AgifIR8a.1
AgifIR8a.2
AgifNmdar1
AgifIR22
AgifIR21
AgifIR15
AgifIR12
AgifIR20
